# Metallothionein-3 is a multifunctional driver that modulates the development of sorafenib-resistant phenotype in hepatocellular carcinoma cells

**DOI:** 10.1186/s40364-024-00584-y

**Published:** 2024-04-09

**Authors:** Miguel Angel Merlos Rodrigo, Hana Michalkova, Ana Maria Jimenez Jimenez, Frantisek Petrlak, Tomas Do, Ladislav Sivak, Yazan Haddad, Petra Kubickova, Vivian de los Rios, J. Ignacio Casal, Marina Serrano-Macia, Teresa C. Delgado, Loreto Boix, Jordi Bruix, Maria L. Martinez Chantar, Vojtech Adam, Zbynek Heger

**Affiliations:** 1 Department of Chemistry and Biochemistry, Mendel University in Brno, Zemedelska 1, Brno, CZ-613 00 Czech Republic; 2 Department of Cellular and Molecular Medicine and Proteomic Facility, Centro de Investigaciones Biológicas (CIB-CSIC), Ramiro de Maeztu 9, Madrid, 280 40 Spain; 3 Liver Disease Laboratory, Center for Cooperative Research in Biosciences (CIC bioGUNE), Basque Research and Technology Alliance (BRTA), Bizkaia Technology Park, Building 801A, Derio, 48160 Spain; 4 Barcelona-Clínic Liver Cancer Group, Liver Unit, Institut d’Investigacions Biomèdiques August Pi I Sunyer, Barcelona, Catalonia Spain; 5 Centro de Investigación Biomédica en Red de Enfermedades Hepáticas y Digestivas (CIBERehd), Instituto de Salud Carlos III, Madrid, Spain

**Keywords:** Metallothionein-3, Resistance, Hepatocellular carcinoma, Sorafenib

## Abstract

**Background & aims:**

Metallothionein-3 (hMT3) is a structurally unique member of the metallothioneins family of low-mass cysteine-rich proteins. hMT3 has poorly characterized functions, and its importance for hepatocellular carcinoma (HCC) cells has not yet been elucidated. Therefore, we investigated the molecular mechanisms driven by hMT3 with a special emphasis on susceptibility to sorafenib.

**Methods:**

Intrinsically sorafenib-resistant (BCLC-3) and sensitive (Huh7) cells with or without up-regulated hMT3 were examined using cDNA microarray and methods aimed at mitochondrial flux, oxidative status, cell death, and cell cycle. In addition, *in ovo*/*ex ovo* chick chorioallantoic membrane (CAM) assays were conducted to determine a role of hMT3 in resistance to sorafenib and associated cancer hallmarks, such as angiogenesis and metastastic spread. Molecular aspects of hMT3-mediated induction of sorafenib-resistant phenotype were delineated using mass-spectrometry-based proteomics.

**Results:**

The phenotype of sensitive HCC cells can be remodeled into sorafenib-resistant one *via* up-regulation of hMT3. hMT3 has a profound effect on mitochondrial respiration, glycolysis, and redox homeostasis. Proteomic analyses revealed a number of hMT3-affected biological pathways, including exocytosis, glycolysis, apoptosis, angiogenesis, and cellular stress, which drive resistance to sorafenib.

**Conclusions:**

hMT3 acts as a multifunctional driver capable of inducing sorafenib-resistant phenotype of HCC cells. Our data suggest that hMT3 and related pathways could serve as possible druggable targets to improve therapeutic outcomes in patients with sorafenib-resistant HCC.

**Supplementary Information:**

The online version contains supplementary material available at 10.1186/s40364-024-00584-y.

## Introduction

 Liver cancer poses a significant global health hurdle, with a projected number of cases surpassing one million by 2025 [1]. Worldwide, hepatocellular carcinoma (HCC) ranks as the sixth leading cause of cancer-related deaths [2]. HCC prognosis remains unsatisfactory, particularly when the disease is diagnosed in an advanced stage [3]. The main risk factors for the onset of HCC are well known and include presence of cirrhosis, chronic hepatitis C, hepatitis B infections, and heavy alcohol consumption [4, 5]. Surgical interventions, such as resection and transplantation, are the most successful methods to treat early HCC. However, since HCC is difficult to diagnose early, most patients tend to be diagnosed in the advanced stages and cannot undergo surgery [6]. Then, sorafenib, a multikinase inhibitor, is a widely used first-line standard systemic anticancer agent [7]. However, only approximately 30% of patients can benefit from sorafenib therapy, and this cohort usually acquires resistance within the first 6 months of chemotherapy [8]. The molecular mechanisms responsible for this phenomenon are not well understood.

Therefore, the main objective of this study is to delineate the molecular mechanisms driving the resistance of HCC cells to sorafenib. As sorafenib is known to induce extensive oxidative stress damage [9], we hypothesized that resistance might be associated with up-regulation of metallothioneins (MTs) that are capable of scavenging reactive oxygen species (ROS) [10], and are known to cause resistance to a variety of anticancer agents [11]. Indeed, recently, Sun et al. suggested that the expression of MT1G is stimulated by sorafenib and could subsequently act as a regulator of sorafenib resistance [12].

MTs belong to a family of low-molecular weight cysteine-rich proteins. Mammalian MTs are classified into four major isoforms (MT1, MT2, MT3, and MT4). MT1 group contains specific sub/isoforms (MT1A, -1B, -1E, -1F, -1G, -1H, -1M and -1X) [13]. The expression of MTs has been suggested as a useful marker for pathogenesis of various types of cancers [14, 15]. Elevated levels of some MTs have also been suggested to mediate protection against apoptosis and promote cell proliferation, thus encouraging tumorigenesis [16, 17].

Recently, few studies have shown that MT1G, MT1H, or MT1M can act as tumor suppressors in HCC [18–21]. Furthermore, MT1G and MT2 have been shown to act as biomarkers of altered redox metabolism in HCC cells [12, 22, 23], underpinning their importance for HCC progression. In contrast to the frequently studied MT1 and MT2, relevant information on the role of MT3 in HCC resistance is lacking. Compared to other MT isoforms, MT3 possesses several unique structural features, such as conserved TCPCP motif in β-domain, EAAEAE hexapeptides insert in α-domain, and acid-basic catalysis motif in β-domain, all of which serve specific purposes (reviewed in [24]). This makes MT3 a unique member of MTs family. Recently, expression of MT3-encoding mRNA *MT3* has been associated with a worse prognosis of breast cancer and neuroblastoma patients, and it has also been shown that *MT3* expression tightly correlates with the poor outcomes of chemotherapy [25–27]. However, the role of MT3 in HCC remains unknown.

Herein, we performed a comprehensive investigation of human BCLC-3 HCC cells possessing intrinsic sorafenib resistant phenotype and sorafenib-sensitive Huh7 cells. For this purpose, an array of experiments focused on understanding the involvement of human MT3 (hMT3) in HCC carcinogenesis and susceptibility to sorafenib was carried out at in vitro and in vivo levels. We provide the first evidence that up-regulation of hMT3 induces the remodeling of sorafenib-sensitive Huh7 cells into sorafenib-resistant phenotype and stimulates intravasation and consequent extravasation of these cells to organs. Moreover, our proteomic survey revealed that transient up-regulation of hMT3 in Huh7 cells stimulates the expression of a number of proteins commonly up-regulated in resistant BCLC-3 cells.

## Materials and methods

### Chemicals

All chemicals and reagents were purchased from Sigma-Aldrich (St. Louis, MO, USA) in ACS purity, unless noted otherwise.

### Cell lines and cell culture

Human HCC cell lines Huh7 were purchased from the American Type Culture Collection (Manassas, VA, USA). Experiments were also performed using the human cell line BCLC-3, obtained and characterized as previously reported [28]. Mouse (C57BL/6) primary hepatocytes were isolated by perfusion with type IV collagenase (Worthington, Lakewood, NJ, USA) according to Barbier-Torres et al. [29]. Animal procedures were approved by CIC bioGUNE’s Animal Care and Use Committee and the competent authority (Diputación de Bizkaia, Spain). BCLC-3 cells were maintained in DMEM/F12, Huh7 cells were cultured in DMEM, and mouse primary hepatocytes were cultured in MEM or methionine/choline deficient medium (MCDM). All media were supplemented with 10% fetal bovine serum, penicillin (100 U/mL), and streptomycin (100 U/mL). Cells were maintained in a humidified CO_2_ incubator. For all experiments, viability was screened using trypan blue exclusion followed by quantification of live/dead cells by the Countess FL II Automated Cell Counter (Thermo Fisher Scientific, Waltham, MA, USA).

### Evaluation of cytotoxicity of sorafenib in 2D and 3D cultures of HCC cells

Susceptibility of HCC cells to sorafenib was investigated using MTT assay. Briefly, a suspension of ~ 2.5 × 10^4^ cells was added to each well of 48 well plates and the cells were cultured overnight. Then the medium was replaced with a new medium containing annotated concentrations of sorafenib. After incubation (24 h), the medium was replaced with a fresh medium containing 30 µL of 3-(4,5-dimethylthiazol-2-yl)-2,5-diphenyltetrazolium bromide (MTT) (5 mg/mL) and the cells were incubated for another 3 h at 37 °C. Subsequently, the medium was replaced with 500 µL of 99.9% dimethylsulfoxide, and the absorbance was recorded at 570 nm using Infinite 200PRO (Tecan, Maennedorf, Switzerland).

Additionally, spheroid experiments were carried out using 3D HCC cell cultures. Studying the differences between 2D and 3D cell cultures is crucial for understanding how the 3D culture environment influences cellular behavior. This comparison provides a better representation of physiological processes and enables more accurate investigation of a drug response [30]. Spheroids were generated using the hanging drop method [31]. ~ 1 × 10^4^ of BCLC-3^*WT*^ and Huh7^*WT*^ cells or ~ 5 × 10^3^ of Huh7^*mock*^ or Huh7^*hMT3*^ cells were suspended in medium hanging in the lid of a Petri dish for 4 d. Once spheroids formed, they were transferred to droplets containing annotated amounts of sorafenib in their corresponding culture medium. After that, spheroids were incubated hanging in the lid of a Petri dish for an additional 24 h. Before spheroids generation, BCLC-3^*WT*^ and Huh7^*WT*^ cells were pre-labeled using CellTracker Green (Invitrogen, Carlsbad, CA, USA) to allow their fluorescence visualization. HCC spheroids micrographs were taken 5 days after seeding using an EVOS FL microscope (Thermo Fisher Scientific, Waltham, MA, USA). The relative area of spheroids was measured using ImageJ software (NIH, Bethesda, MA, USA).

### Isolation of RNA and reverse transcription

To isolate total RNA from HCC cells, a high-purity total-RNA isolation kit (Roche, Basel, Switzerland) was used according to the manufacturer’s instructions. 500 ng of isolated RNA was reverse-transcribed using the Transcriptor First Strand cDNA Synthesis Kit (Roche) according to manufacturer’s instructions. The prepared cDNA was diluted with RNase-free water and used for subsequent experiments.

### Electrochemical cDNA microarray

To compare the transcriptomic landscapes of BCLC-3^*WT*^ and Huh7^*WT*^ cells, 3′-end of cDNA was biotinylated with the Biotin 3′ End DNA Labeling Kit (Thermo Fisher Scientific) according to the manufacturer’s instructions. Subsequent hybridization of cDNA on ElectraSense 4 × 2k array slides (Custom Array, Bothell, WA, USA) was carried out according to our previously published study [25]. After hybridization, the microarray chips were rinsed (3×) using biotin wash solution and 3,3′,5,5′-tetramethylbenzidine (TMB) rinsing solution, followed by incubation of chips with TMB. Readout was carried out using the ElectraSense Reader (Custom Array). The threshold values for up- and down-regulation were set to the median gene expression fold ratio of BCLC-3^*WT*^ cells ≥ 1.5 and ≤ 0.5 compared to Huh7^*WT*^ cells.

### Real-time quantitative reverse-transcription polymerase chain reaction

To validate proteomic data and determine the expression of the hMT3, a real-time quantitative reverse-transcription polymerase chain reaction (qRT-PCR) with the Luna Universal RT-qPCR kit was carried out using the qTOWER^3^ cycler (Analytik Jena, Jena, Germany). The specificity of qRT-PCR was validated through a melting curve analysis. Relative transcription levels were calculated using the 2^−ΔΔCT^ method. The primers used for qRT-PCR are shown in the Suppl. Table 1.

### Plasmid construction, transfection, and transfection validation

Vector construction was carried out according to our previously published study [32]. The constructed and validated (sequencing) vector was transfected into Huh7^*WT*^ cells using Lipofectamine 2000 (Thermo Fisher Scientific, Waltham, MA, USA) according to the manufacturer’s instructions to obtain Huh7^*hMT3*^ cells. In addition, an empty pcDNA3.1-GFP-TOPO (Thermo Fisher Scientific) was prepared and transfected to obtain control mock cells (Huh7^*mock*^). GFP expression was validated in 4% formaldehyde-fixed cells using a confocal laser-scanning microscope (CLSM) LSM 880 (Carl Zeiss, Jena, Germany).

### Real-time cell proliferation assay

Label-free analysis of the effect of hMT3 up-regulation on proliferation of Huh7 cells was performed using real-time electrical impedance measurement on the xCELLigence RTCA DP instrument (Roche Diagnostics GmbH, Basel, Switzerland) according to a previous study [33].

### hMT3 expression in HCC patients and Kaplan-Meier survival analysis

To determine the expression of *MT3* in HCC and healthy adjacent tissue, RNA-Seq data (containing paired tumor vs. adjacent normal tissue data) were extracted from TNMplot (www.tnmplot.com) [34]. Furthermore, the expression of *MT3* in HCC patients based on individual cancer stage, nodal metastasis status, and tumor grades vs. normal tissue data was determined using the UALCAN analysis platform (http://ualcan.path.uab.edu/) [35]. The cBio Cancer Genomics Portal (https://www.cbioportal.org/) was used to determine the expression of *MT3* in HCC patients based on the stage of HCC at diagnosis in sorafenib-treated and untreated cohorts [36]. Further, Kaplan-Meier plots that represent the overall survival of primary HCC cohorts stratified according to the expression of *MT3* were prepared using the KM plotter database (www.kmplot.com) [37] using pan-cancer – liver HCC and the RNA-SeqID:4504 cohorts. The high and low expression cohorts were classified according to the *MT3*-encoding mRNA expression above or below the median. The log-rank test was employed to evaluate the correlation between the *MT3* expression and survival outcomes. A *p* value ≤ 0.05 was considered statistically significant.

### Mitochondrial flux analyses

Cells were seeded at a density of ~ 1 × 10^5^ cells per well in 24-well Seahorse cell culture plates (Seahorse Biosciences, North Billerica, MA, USA) in DMEM without carbonate. After incubation (1 h at 37 °C without CO_2_), real-time oxygen consumption rate (OCR) and extracellular acidification rate (ECAR) analyses were performed using Seahorse Extracellular Flux Analyzer (Seahorse Biosciences) as previously described by Barbier-Torres and coworkers [29].

### Quantification of total intracellular ROS

For ROS analysis, cell suspension (~ 5 × 10^4^ per well) was seeded in flat bottom tissue culture plates and incubated overnight. Subsequently, cells were exposed to annotated amounts of sorafenib and incubated for another 24 h. The cells were then washed with phosphate-buffered saline (PBS, pH 7.4) and stained with CellROX Deep Red Reagent (Thermo Fisher Scientific) according to the manufacturer’s instructions. Finally, cells were harvested by trypsin, centrifuged (300×*g*, 5 min, 4°C), and fluorescence intensity was analyzed using the BD Accuri C6 Plus (BD Biosciences, Franklin Lakes, NJ, USA). Dead cells were gated out by 7-aminoactinomycin D (7-AAD) staining. For each experiment, at least 25,000 events/samples were recorded.

### Quantification of mitochondrial superoxide

Mitochondrial superoxide production was assessed using the MitoSOX Red Mitochondrial Superoxide Indicator (Thermo Fisher Scientific). The cells were loaded with 1.5 mM MitoSOX and incubated (10 min, 37 °C). The cells were then washed with hot PBS (3×). Fluorescence (λ_exc_ = 510 nm, λ_em_ = 560 nm) was analyzed using a SpectraMax M2 plate reader (Molecular Devices, CA, USA).

### Determination of superoxide dismutase activity

For assessment of superoxide dismutase (SOD) activity, cells were lysed, and SOD activity was determined using Superoxide Dismutase Activity Assay Kit (Abcam, Cambridge, UK) according to the manufacturer’s protocol. SOD activity was analyzed in 10 µg of total protein extract. Absorbance was determined at 450 nm using a SpectraMax M2 plate reader (Molecular Devices).

### Determination of apoptosis induction by sorafenib administration

Apoptotic cell death was determined using flow cytometry by simultaneous staining with Annexin V-Dyomics 647 and 7-AAD (Exbio, Prague, Czech Republic). Cells were seeded (~ 1 × 10^5^ per well) in 96-well flat bottom tissue culture plates. After incubation with or without sorafenib, the cells were transferred to 96-well tissue culture plates with conical bottom and washed with 200 µL of Annexin binding buffer (10 mM HEPES, 140 mM NaCl, 2 mM CaCl_2_, pH 7.4). The cells were then stained with 10 µL Annexin V-Dyomics 647 and incubated for 15 min at room temperature in the dark. Subsequently, Annexin binding buffer and 7-AAD were added to the final volume of 100 µL, and the samples were immediately analyzed using BD Accuri C6 Plus (BD Biosciences). At least 30,000 events were analyzed for each sample. Positioning of quadrants in Annexin V-Dyomics 647/7-AAD dot plots was performed to distinguish between living cells (Annexin V^−^/7-AAD^−^), early apoptotic cells (Annexin V^+^/7-AAD^−^), late apoptotic cells (Annexin V^+^/7-AAD^+^) and death cells/cell debris (Annexin V^−^/7-AAD^+^).

### Cell cycle analysis

Cell cycle distribution analyses were carried out by staining the DNA with propidium iodide (PI). HCC cells were seeded (~ 3 × 10^5^ per well) into 48-well flat bottom culture tissue plates and incubated with or without sorafenib for 24 h. After treatment, cells were harvested and washed (2×) with PBS. Resulting pellets were fixed with ice-cold 70% ethanol and collected by centrifugation (850×*g*, 10 min, 4 °C), rinsed with ice-cold PBS (2×), and resuspended in 300 µL of PI staining solution (0.1% Triton X-100, (*v*/*v*), 50 µg/mL of RNase A and 50 µg/mL of PI). Samples were incubated (60 min, 4 °C) in the dark and analyzed using BD Accuri C6 Plus (BD Biosciences). For each experiment, 50,000 events per sample were recorded.

### Chick chorioallantoic membrane (CAM) assay

To determine the susceptibility of HCC cells to sorafenib and their subsequent ability to form metastatic spreads, *ex ovo* and *in ovo* CAM assays were employed. In this study, we followed the conditions of CAM assay previously reported in our study [38]. The fertilized chicken eggs were obtained from Integra farm (Zabcice, Czech Republic). For the *ex ovo* CAM assay, fertilized chicken eggs were incubated with rotation at 37.5 °C and 65% humidity for 3 d. Before xenografting, HCC cells were pre-labeled with CellTracker Green (Invitrogen) and implanted on the CAM at an initial seeding density of ~ 5 × 10^4^. After incubation (3 d), 100 µM sorafenib was added to each microtumor, and *ex ovo* cultures were further incubated at 37.5 °C for 24 h. For fluorescent angiography, 50 µL of 10 µg/mL of rhodamine-labeled *Lens culinaris* agglutinin (LCA) (Vector Laboratories, Burlingame, CA, USA) was injected into the peripheral veins of the viable CAM using a 30G hypodermic needle attached to a 1 mL syringe. Microtumors-infested CAM areas were cut with a 3/4 cm margin around them and fixed in 4% paraformaldehyde (Sigma Aldrich) in PBS. For subsequent fluorescent imaging, EVOS FL Auto Cell Imaging System (Thermo Fisher Scientific) was used. The relative area (%) of the tumors, intravasation, and vascular density were quantified using ImageJ software (NIH). To confirm the development of 3D tumors, micrographs of cross-sections of untreated tumors were obtained using LSM 880 (Carl Zeiss) in 3D mode.

For the *in ovo* CAM assay, fertilized chicken eggs were incubated with intermittent rotation (37.5 °C, 65% humidity, 10 d). After this period, a square window (~ 1 cm^2^) was drilled into eggshell close to the bifurcation of the allantoid vein. The eggs were then inoculated with ~ 1 × 10^6^ of Huh7^*WT*^ and BCLC-3^*WT*^, or ~ 5 × 10^5^ Huh7^*mock*^ and Huh7^*hMT3*^ cells grafted directly on the CAM, followed by incubation (37.5 °C, 6 d). Then 100 µM sorafenib was added topically on the upper CAM directly to the developed tumors, and the eggs were kept (30 min) without movement to allow absorption of sorafenib to the tumors and avoid its runoff. After 24 h, CAM containing tumors, and lungs were harvested, weighed and stained with hematoxylin-eosin, or used for extraction of DNA followed by determination of tumor burden through quantification of human-specific *alu* sequences using *alu-*qPCR according to [39]. In EU countries, CAM assay is not declared as an animal experiment by law and therefore does not require ethical approval.

### Matrix-assisted laser desorption/ionization time-of-flight mass spectrometry

Tumors excised from the CAM assay were further processed to determine the post-transfection stability of hMT3 expression and validate up-regulation of hMT3 at the experimental end-point. For this purpose, tumors were embedded in 2% carboxymethylcellulose and 10% gelatin and sliced using a Leica CM 1850 cryostat (Leica, Wetzlar, Germany). 10-µm cryosections were then mounted onto cold indium-tin-oxide coated glass and stored at -80 °C. Matrix-assisted laser desorption/ionization mass spectrometry imaging (MALDI-MSI) was carried out using a Bruker ultrafleXtreme MALDI tandem time-of-flight mass (MALDI-TOF/TOF) spectrometer (Bruker Daltonik GmbH, Bremen, Germany) according to our previously published study [40]. An external calibration was performed using a commercial protein calibration mixture (Bruker Daltonik GmbH) in mass range of 2–20 kDa.

### Mass spectrometry-based proteomics

After extraction with RIPA buffer, proteins were reduced, alkylated and digested with trypsin (37 °C, overnight). The obtained peptides were desalted and separated on an Easy-nLC 1000 (Thermo Fisher Scientific). For analyses, samples were loaded into an Acclaim PepMap 100 precolumn (Thermo Fisher Scientific) and eluted in a RSLC PepMap C18 with inner diameter of 75 μm and particle size of 2 μm (Thermo Fisher Scientific). The mobile phase flow rate was 300 nL/min using 0.1% formic acid in water and 0.1% formic acid and 100% acetonitrile. MS analysis was performed using a Q-Exactive mass spectrometer (Thermo Fisher Scientific). For the ionization, 2,000 V of liquid junction voltage and 270 °C capillary temperature were used. The full scan method employed an *m/z* 400–1,500 mass selection, a resolution of 70,000 (at *m*/*z* 200), a target automatic gain control (AGC) value of 3e6, and maximum injection times of 100 ms. After the survey scan, the 15 most intense precursor ions were selected for MS/MS fragmentation. Fragmentation was performed with a normalized collision energy of 27 eV and MS/MS scans were acquired with a starting mass of *m*/*z* 100, AGC target was 2e5, resolution of 17,500 (at *m*/*z* 200), intensity threshold of 8e3, isolation window of 2 *m*/*z* units. Charge-state screening was enabled to reject unassigned, singly charged, and equal or more than seven protonated ions. A dynamic exclusion time of 20 s was used to discriminate against previously selected ions. Mass spectra *.raw files were searched against SwissProt_2016_10.fasta, *Homo sapiens* (human) database (20,121 sequences protein entries) using Mascot search engine (version 2.6, Matrix Science). The precursor and fragment mass tolerance was set at 10 ppm and 0.02 Da, respectively, allowing for 2 missed cleavages, carbamidomethylation of cysteines as a fixed modification, methionine oxidation and acetylation *N*-terminal as a variable modification [41]. Identified peptides were filtered using the Percolator algorithm with a q-value threshold of 0.01.

### BODIPY staining of lipid bodies in primary murine hepatocytes

Hepatocytes were obtained as described by Serrano-Macia et al. [42]. For analyses of lipid bodies formation, WT, mock-transfected and hMT3-overexpressing mouse primary hepatocytes were cultured (48 h) in MCDM or MEM as negative control after transfection using jetPRIME reagent (Polyplus, Berkeley, CA, USA). Cells were further incubated with BODIPY 493/503 (Molecular Probes, Eugene, OR, USA) at a concentration of 1 mg/mL for 30 min followed by fixation with 4% paraformaldehyde. After staining, cells were observed under a fluorescence microscope (Axiomager D1, Carl Zeiss). Quantification of lipid bodies was performed using the Frida Software (FRamework for Image Dataset Analysis).

### Bioinformatical tools

For statistical evaluation of the results, the data were expressed as mean ± SEM. Differences between groups were analyzed using Student’s test (two-sided) and ANOVA. The MALDI-MSI molecular maps were generated and visualized using SCiLS Lab 2014b software (Bruker Daltonik GmbH). Furthermore, using SCiLS Lab, MSI data were processed by segmentation pipeline and statistical analysis (Anderson-Darling normality test and the Kruskal-Wallis test). The final images were edited in GIMP 2.8 (www.gimp.org). The flow cytometry data were analyzed using FlowJo software (Tree Star, Inc., Ashland, OR, USA). Proteomic data were analyzed with Proteome Discoverer v.1.4.1.14 (Thermo Fisher Scientific) using standardized workflows. The list of processes and/or pathways driven by regulated genes and proteins was created by Protein-Protein Interaction Networks Functional Enrichment Analysis (STRING), and METASCAPE analysis tool (metascape.org).

## Results

### Evaluation of susceptibility of 2D and 3D cultures of wild-type HCC cells to sorafenib

**Fig. 1:**
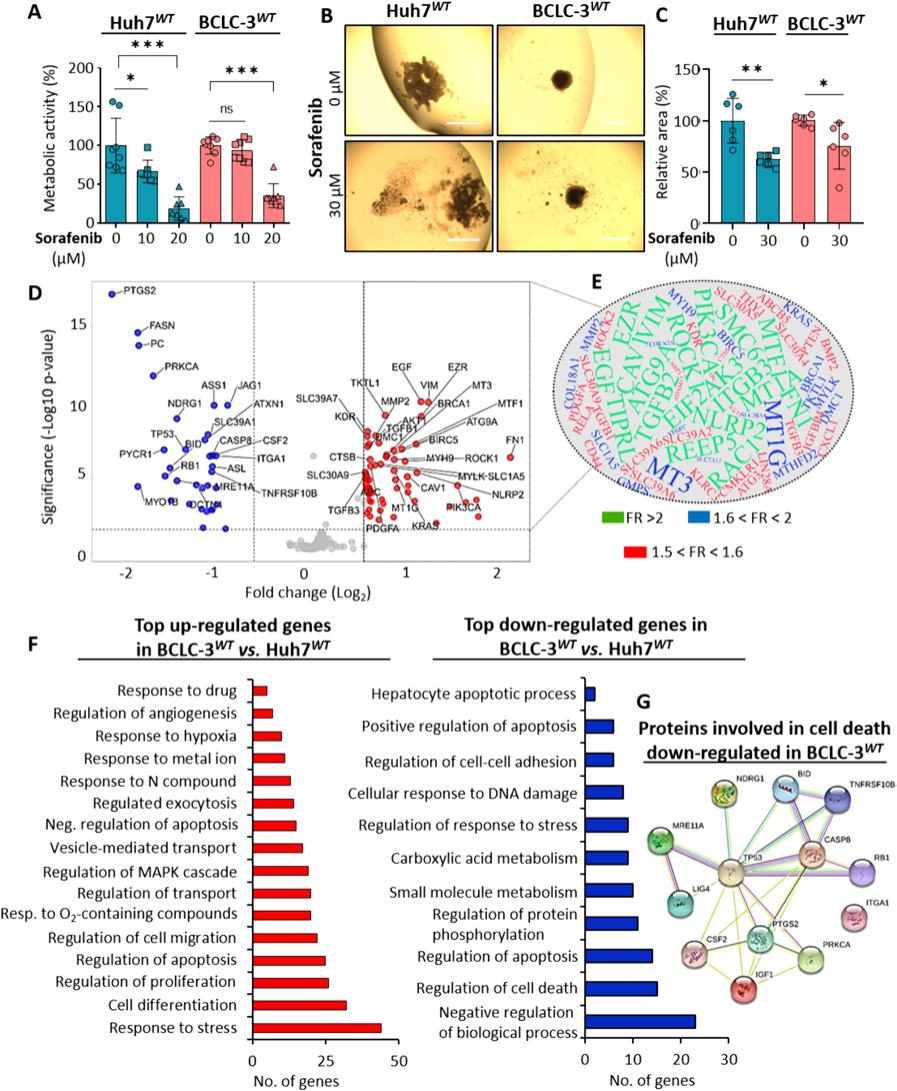
Comparative transcriptomics analysis of HCC cell lines intrinsically sensitive (Huh7^***WT***^) or resistant (BCLC-3^***WT***^) to sorafenib. **A** Dose-response metabolic activity of HCC cells administered with sorafenib. **B** HCC spheroid cultures demonstrating differential response to sorafenib. Scale bars, 200 μm. **C** Quantification of the relative area (%) of HCC spheroids carried out using ImageJ software. Data are expressed as mean ± SEM *p ≤ 0.05, **p ≤ 0.01, ***p ≤ 0.001 (Student’s test, two-sided) related to control non-treated cells. **D** Comparative transcriptomics analysis of HCC cell lines expressed as Volcano plot of log_2_ ratio *versus* – log_10_ *p* value of DEGs. **E** Tag cloud network of 64 enriched genes identified consistently across the transcriptomic profile of sorafenib-resistant BCLC-3^*WT*^ cells compared to sorafenib-susceptible Huh7^*WT*^ cell line. The font size and colors (green > blue > red) change proportionally according to the average FR. **F** Classification of cellular functions and canonical pathways of genes differentially up- and down-regulated in resistant BCLC-3^*WT*^ (vs. susceptible Huh7^*WT*^) cells as predicted by String software. **G** Interactome network showing the proteins encoded by mRNAs, which were found down-regulated in BCLC-3^*WT*^ cells compared to Huh7^*WT*^ cell line and are involved in regulation of cell death process. The color of the line provides evidence of the different interactions among proteins

We first revalidated the susceptibility of BCLC-3^*WT*^ and Huh7^*WT*^ cells to sorafenib (Fig. 1A). BCLC-3^*WT*^ cells exhibited higher resistance to sorafenib, which is in line with previously published studies [43, 44]. In the next set of experiments, additional parameters of 3D spheroid cultures including morphology and solidity were monitored (Suppl. Figure 1A). The data revealed that BCLC-3^*WT*^ cells formed relatively tightly compacted spheroids with pronounced quiescent and proliferating zones, while Huh7^*WT*^ cells formed into rather loosely compacted aggregates. Furthermore, the efficacy of sorafenib in 3D HCC cultures was determined. As shown in Fig. 1B and C, sorafenib administration resulted in a significant reduction of Huh7^*WT*^ spheroid area (by 37.7 ± 6.02%), while a significantly lower area reduction was found for BCLC-3^*WT*^ cells (24.4 ± 20.7%), which is consistent with their sorafenib resistant phenotype. It is worth noting that morphologically, in Huh7^*WT*^ cells, sorafenib triggered additional spheroids loosening, while BCLC-3^*WT*^ spheroids were mostly affected only within the superficial proliferating layer (Suppl. Figure 1B).

### Comparative transcriptomic analysis of BCLC-3^*WT*^ and Huh7^*WT*^ cells

To delineate the molecular aspects associated with the resistance of BCLC-3^*WT*^ cells, we investigated their transcriptomic patterns and compared them with transcriptome of Huh7^*WT*^ cells. The data revealed highly differentiated transcriptomic profiles as obvious from volcano plot of differentially expressed genes (DEGs) (Fig. 1D). The full list of up- (*n* = 64) and down-regulated (*n* = 33) genes is shown in Suppl. Table 2. Expression of selected mRNAs was then cross-validated with MS/MS data (Suppl. Figure 1C). A closer look at the microarray dataset revealed that among the DEGs up-regulated in BCLC-3^*WT*^ cells, three members of the MT family (MT3, MT1A and MT1G) were identified (Fig. 1D and E). This suggests their importance for the resistance of BCLC-3^*WT*^ cells. To further evaluate the biological relevance of DEGs, we performed GO enrichment analysis. As shown in Fig. 1F, the set of up-regulated genes was involved in response to stress factors, regulation of proliferation, migration, exocytosis, angiogenesis, and response to drug processes. On the contrary, the set of down-regulated genes mostly affects pathways related to apoptosis, DNA damage, and repair and metabolic processes. The interactome network in Fig. 1G shows an interconnected network of proteins down-regulated in BCLC-3^*WT*^ cells involved in a cell death regulation. Overall, the presented data confirm that BCLC-3^*WT*^ cells utilize a complex molecular machinery capable of alleviating the cytotoxicity of sorafenib and inhibiting induction of apoptosis. Importantly, based on the data, MTs seemed to be a part of this machinery.

### hMT3 expression positively correlates with resistance of HCC to Sorafenib

**Fig. 2:**
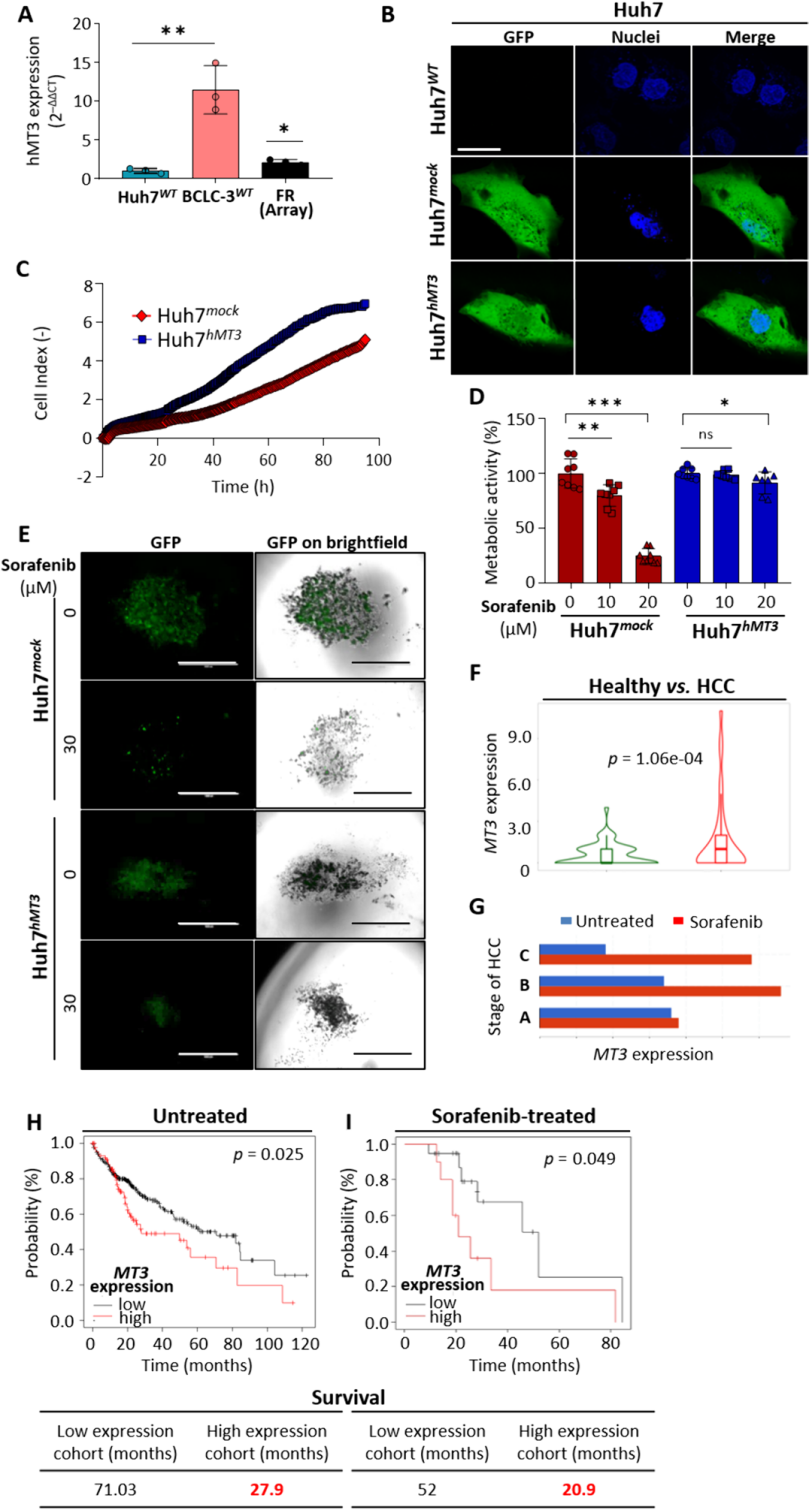
Transiently induced expression of hMT3 increases the resistance of intrinsically susceptible Huh7 cells to sorafenib. **A** Analysis of a baseline expression of hMT3 in Huh7^*WT*^ and BCLC-3^*WT*^ cells, accompanied by FR value obtained by cDNA microarray. *p ≤ 0.05, **p ≤ 0.01 (Student’s test, two-sided). **B** Representative micrographs of Huh7^*WT*^ cells and the same cells transfected either with pcDNA3.1-GFP-TOPO (Huh7^*mock*^ transfection) or pcDNA3.1-GFP-hMT3-TOPO (Huh7^*hMT3*^). Scale bar, 10 μm. **C** Effect of hMT3 up-regulation on proliferation rate of Huh7 cells analyzed using real-time electrical impedance measurement. **D** Dose-response metabolic activity of Huh7^*mock*^ and Huh7^*hMT3*^ cells exposed to sorafenib (24 h). Data are expressed as mean ± SEM (*n* = 3). *p ≤ 0.05, **p ≤ 0.005, ***p ≤ 0.0001. **E** Representative micrographs of spheroids formed by Huh7^*mock*^ and Huh7^*hMT3*^ cells exposed to sorafenib (24 h). Scale bars, 200 μm. **F** *MT3* expression in normal liver (*n* = 50) and HCC (*n* = 50) tissues. **G** *MT3* expression in various stages of HCC (A, early stage; B, intermediate stage; C, advanced stage) in patients either treated (red) or untreated (blue) with sorafenib. The data were gathered and analyzed using the cBioPortal for Cancer Genomics. **H** The Kaplan-Meier curves indicating that in the studied HCC cohort (*n* = 364, RNA-seqID: 4504), subjects with high expression of *MT3* exhibited worse prognosis than patients with low expression of *MT3*. (**I**) The Kaplan-Meier curves showing that HCC patients with high expression of *MT3* treated with sorafenib (*n* = 30) exhibited significantly lower survivability than sorafenib-treated subjects with low *MT3* expression. Data were derived from Kaplan-Meier plotter database. *p* ≤ 0.05 was considered statistically significant

Based on the transcriptomic data, our next steps led us to examine a baseline expression of *MT3* in tested HCC cells. The obtained data corroborated significantly higher (**p ≤ 0.01) baseline expression of *MT3* in BCLC-3^*WT*^ compared to Huh7^*WT*^ cells and supported our original hypothesis about the link between hMT3 and sorafenib resistance (Fig. 2A).

With the idea that up-regulation of hMT3 in sorafenib-sensitive cells should remodel their phenotype into resistant one, we constructed a plasmid encoding full-length hMT3. As shown in Fig. 2B, after transfection, Huh7^*mock*^ and Huh7^*hMT3*^ cells exhibited normal morphology and homogeneous cytoplasmic spread of fluorescent reporter with a transfection efficiency > 70%. As determined by RT-qPCR, the transfection with hMT3-encoding vector resulted in ~ 12-fold higher relative expression of *MT3* compared to Huh7^*WT*^ and Huh7^*mock*^ cells (Suppl. Fig. 1D). In addition, real-time impedance measurements revealed a marked stimulatory effect of hMT3 up-regulation on cellular proliferation of Huh7^*hMT3*^ cells (Fig. 2C). It should also be noted that the cytotoxicity screenings revealed that in 2D culture, Huh7^*hMT3*^ cells gained substantial resistance to sorafenib not observable for Huh7^*mock*^ cells (Fig. 2D). Revalidation of these findings using 3D spheroids led to a confirmation that Huh7^*mock*^ spheroids exhibited pronouncedly higher susceptibility to sorafenib compared Huh7^*hMT3*^ spheroids (Fig. 2E and Suppl. Figure 1E).

By analyzing available RNA-Seq libraries containing paired tumor vs. adjacent healthy tissue data, we found that HCC cells express a considerably higher amount of *MT3* (Fig. 2F). Based on the RNA-Seq data of HCC-diagnosed subjects, *MT3* expression positively correlates with HCC stage; nodal metastasis status and tumor grade (Suppl. Figure 1F). Further data mining revealed that while in untreated patients, *MT3* expression decreased from early to the advanced stage of HCC, sorafenib-treated patients exhibited substantially higher *MT3* expression in the intermediate and advanced stages of the disease (Fig. 2G), suggesting stimulatory activity of sorafenib on *MT3* expression. Subsequent survival analyses revealed that both analyzed cohorts (untreated vs. sorafenib-treated) exhibited significantly lower survival associated with high expression of *MT3* (Fig. 2H and I). In addition, survival of both high-expression cohorts did not markedly differ further indicating importance of *MT3* for sorafenib-resistance. These data suggest that *MT3* expression could be a clinically relevant biomarker to predict aggressiveness of HCC.

### hMT3 affects mitochondrial flux and ROS generated by sorafenib exposure

**Fig. 3:**
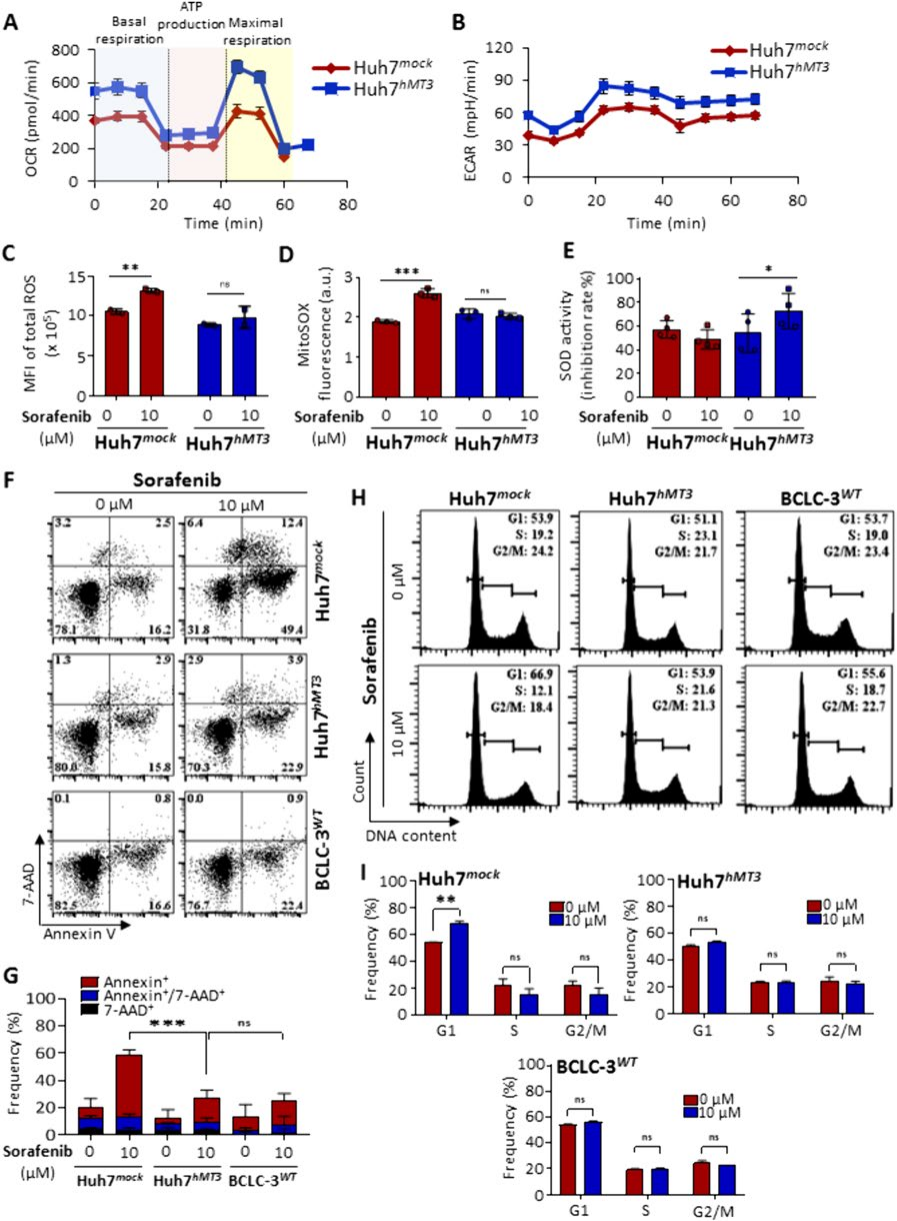
In Huh7 cells, hMT3 affects mitochondrial flux, oxidative stress, apoptosis, and cell cycle. Metabolic analysis of Huh7^*mock*^ and Huh7^*hMT3*^ cells expressed as (**A**) OCR and (**B**) ECAR values (*n* = 6). **C** Sorafenib-mediated induction of total ROS, (**D**) mitochondrial superoxide and (**E**) SOD activity in Huh7^*mock*^ and Huh7^*hMT3*^ cells. *p ≤ 0.05, **p ≤ 0.001, ***p ≤ 0.0005 (Student’s test, two-sided). **F** Scatter plots of double-stained (Annexin V-Dy647/7-AAD) cells showing markedly lower ability of sorafenib to trigger apoptosis in Huh7^*hMT3*^ and BCLC-3^*WT*^ compared to Huh7^*mock*^ cells. **G** Quantification of flow cytometry analysis of apoptosis induction by sorafenib. Data are expressed as mean ± SEM. ***p ≤ 0.0005 (Student’s test, two-sided). **H** Cell cycle histograms of PI-stained cells cultured with or without sorafenib. **I** Bar graphs showing the quantification of the frequencies of cells in individual cell cycle phases. Data are expressed as mean ± SEM. **p ≤ 0.001 (Student’s test, two-sided)

We further performed experiments to delineate the role of hMT3 in metabolic reprogramming and verify the ability of hMT3 to alleviate ROS-induced damage in HCC cells exposed to sorafenib. Huh7^*hMT3*^ cells were found to exhibit substantially higher mitochondrial oxidative phosphorylation (Fig. 3A), indicating an improvement in the efficiency of mitochondrial respiration due to hMT3 up-regulation. Moreover, hMT3 up-regulation also resulted in markedly higher ECAR values (Fig. 3B), suggesting a stimulatory role of hMT3 in reprogramming of energetic metabolism towards glycolysis, which belongs among hallmarks of HCC oncogenesis [45]. Since the increased respiration could lead to augmented levels of mitochondrial ROS levels, we next assessed the levels of total ROS, mitochondrial superoxide, and SOD activity in Huh7^*mock*^ and Huh7^*hMT3*^ cells. Indeed, our data confirmed that in contrast to Huh7^*mock*^ cells, in Huh7^*hMT3*^ cells, sorafenib administration did not lead to a significantly increased amount of total ROS (Fig. 3C) or mitochondrial superoxide (Fig. 3D). Furthermore, Huh7^*hMT3*^ cells exposed to sorafenib exhibited significantly (*p* < 0.05) higher SOD activity than Huh7^*mock*^ cells (Fig. 3E). This is plausibly caused by ROS-triggered release of Zn^2+^ ions from metal-thiolate clusters of hMT3 and their consequent utilization as cofactors at the active center of SOD, resulting in enhancement in SOD activity [46]. The obtained data underpin the importance of hMT3 for mitochondrial fluxes and confirm its ability to protect HCC cells against ROS.

### Effect of up-regulated hMT3 on sorafenib-induced apoptosis and cell cycle arrest

Our next aim was to determine effect of hMT3 on sorafenib-triggered apoptosis induction. Scatter plots shown in Fig. 3F clearly demonstrate the intrinsic resistance of BCLC-3^*WT*^ and sensitivity of Huh7^*mock*^ cells. After up-regulation of hMT3, Huh7^*hMT3*^ underwent a switch to a sorafenib-resistant phenotype comparable to intrinsically resistant BCLC-3^*WT*^ cells (Fig. 3G). The obtained results are in line with the ability of hMT3 to efficiently scavenge sorafenib-induced ROS and subsequently alleviate its cytotoxicity. Next, we investigated the effect of hMT3 up-regulation on cell cycle distribution. In susceptible Huh7^*mock*^ cells, sorafenib induced a cell cycle arrest at the G1 phase (Fig. 3H). It is worth noting that either in BCLC-3^*WT*^ or Huh7^*hMT3*^ cells, G1 arrest was not identified (Fig. 3I), confirming the ability of hMT3 to inhibit activity of sorafenib.

### hMT3 up-regulation induces sorafenib-resistant phenotype of HCC cells in vivo

**Fig. 4:**
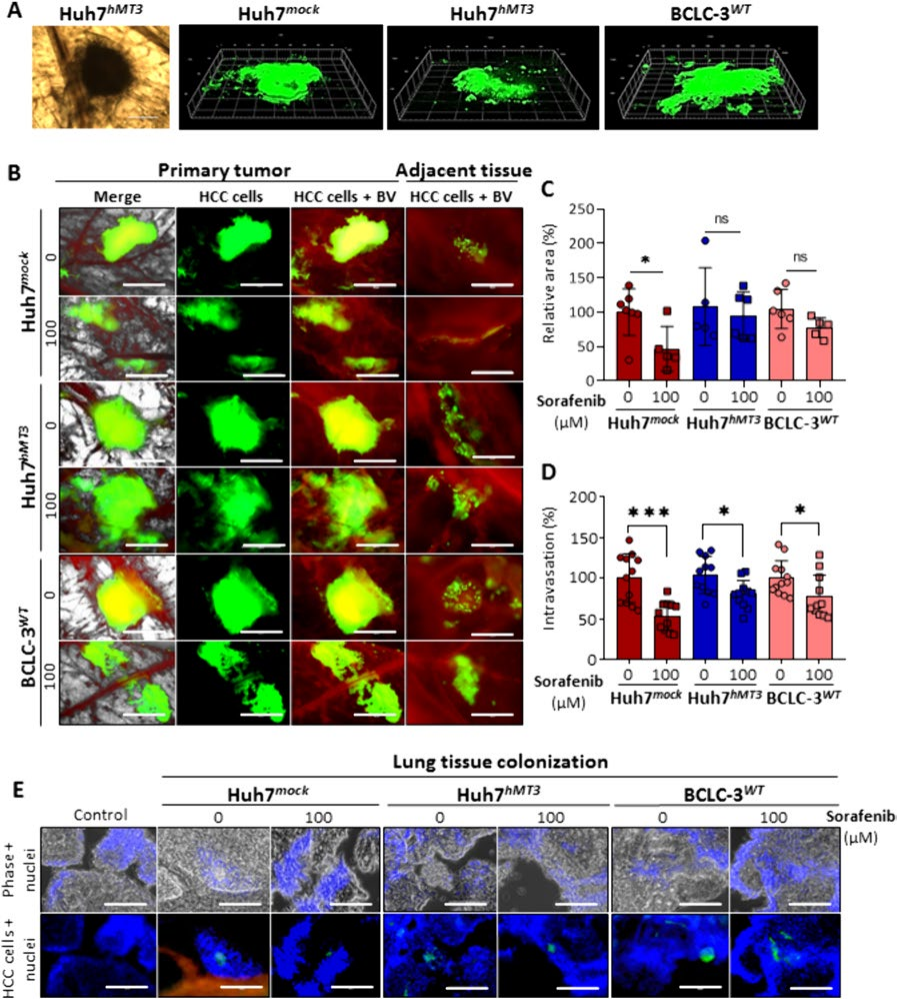
Up-regulation of hMT3 induces the remodeling of sorafenib-sensitive HCC tumors into sorafenib-resistant phenotype. **A** Representative 3D CLSM micrographs of untreated microtumors formed by Huh7^*mock*^, Huh7^*hMT3*^, and BCLC-3^*WT*^ cells. **B** Fluorescence micrographs of microtumors on the area of implantation of HCC cells on CAM either non-treated or treated with sorafenib (24 h). HCC cells are green (labeled with CellTracker Green), angiogenic blood vessels (BV) are red (labeled with rhodamine-LCA). Scale bars, 1 mm. In a zoomed micrographs, individual HCC cells that detached from the main microtumor could be detected intravasating into the endothelial cells in the adjacent tissues. Scale bars, 200 μm. **C** Quantification of the relative area (%) of the HCC microtumors and (**D**) invasive vasculotropic (intravasation, %) HCC cells that escaped from the primary tumor. Data are expressed as mean ± SEM. *p ≤ 0.05, **p ≤ 0.001. **E** Fluorescence micrographs of lungs excised from CAM assay. Metastasizing HCC cells are green, BV are red and nuclei blue (Hoechst 33258). Scale bars, 100 μm

In the next steps, we determined the role of hMT3 in sorafenib resistance, intravasation, extravasation, and metastatic spread of HCC cells in vivo. First, the *ex ovo* CAM assay was conducted to study the inhibitory effects of sorafenib on the growth of HCC xenograft. Figure 4A shows that HCC xenografts were properly established on the upper CAM as 3D tissue structures resembling solid tumors (Suppl. Figure 2A). Subsequently, analysis of relative tumor areas confirmed that while Huh7^*mock*^ tumors were largely inhibited by sorafenib administration, Huh7^*hMT3*^ and BCLC-3^*WT*^ tumors displayed only a limited insignificant response to treatment (Fig. 4B and C). We found that sorafenib inhibited intravasation in all analyzed cohorts, but the highest inhibition of intravasation was again determined in Huh7^*mock*^ cells (approx. 46% inhibition) (Fig. 4D). Fluorescence angiography revealed that compared to the rest of groups Huh7^*hMT3*^ tumors developed a substantially higher amount of blood vessels supplying tumor mass suggesting involvement of hMT3 in angiogenesis (Suppl. Figure 2B), which is consistent with analysis of BCLC-3^*WT*^ cells DEGs by cDNA microarray. Subsequently, we analyzed lung tissues for possible dissemination of HCC cells from primary tumors. As shown in Fig. 4E, in contrast to Huh7^*mock*^ cells, sorafenib had only limited effect on dissemination of Huh7^*hMT3*^ and BCLC-3^*WT*^ cells, again confirming intrinsic/induced resistance of these tumors.

**Fig. 5:**
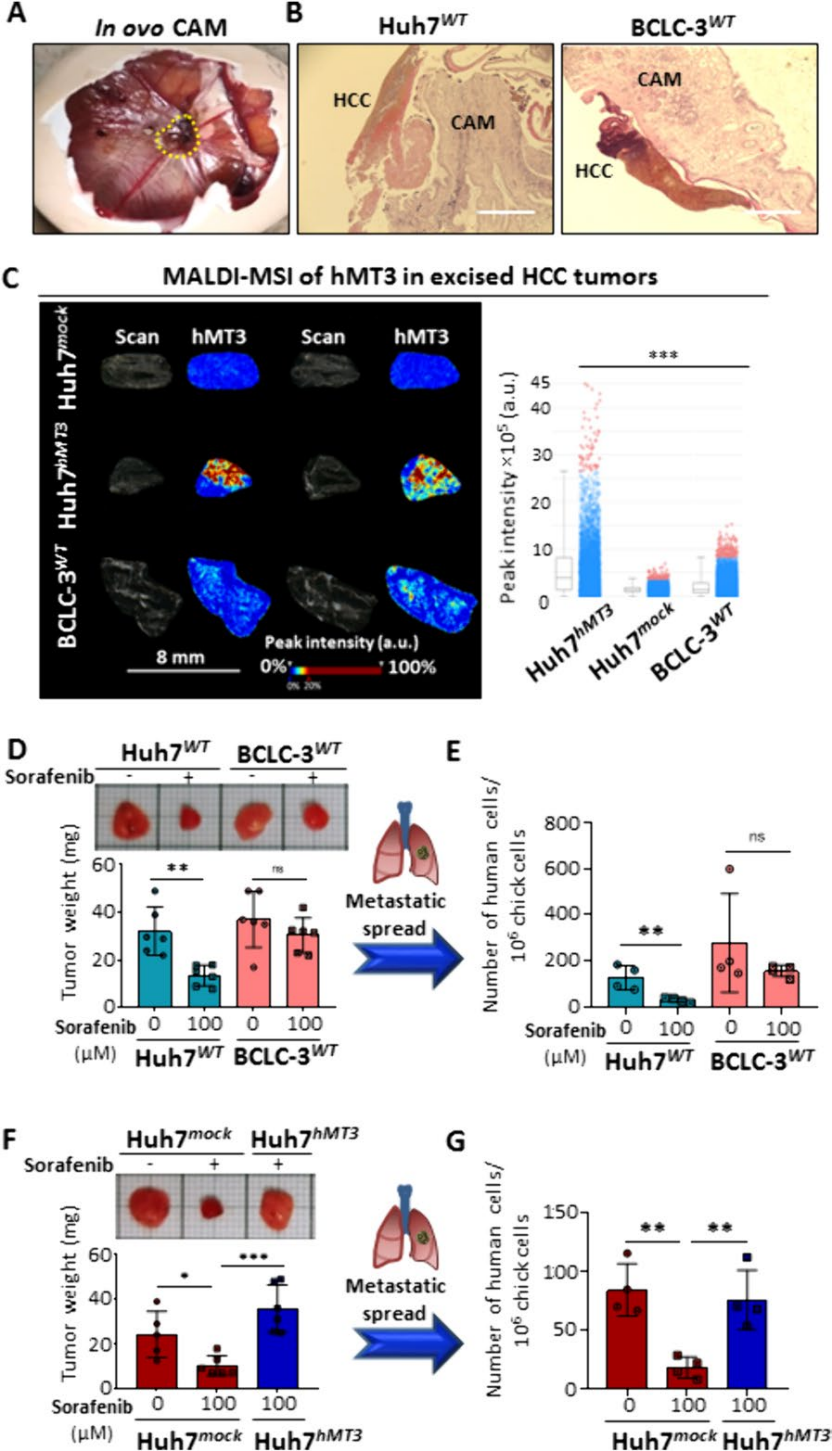
Evaluation of efficiency of sorafenib to inhibit HCC growth and metastatic spread of HCC. **A** Representative photograph of *in ovo* CAM assay with the xenografted human HCC (yellow dashed line) formed on CAM 7th day after induction. **B** H&E staining of HCC excised from *in ovo* CAM assay at the beginning of the experiment. Scale bars 400 μm. **C** MALDI MSI of untreated primary HCC tissue cryosections excised from CAM at the experimental end-point validating up-regulation of hMT3 in Huh7^*hMT3*^ and increased expression of hMT3 in BCLC-3^*WT*^ cells. Bar graph shows peak intensity plots of hMT3 expression in HCC tissues. Data are expressed as mean ± SEM. ***p ≤ 0.001 (Kruskal-Wallis test). **D** Representative photographs and weights of Huh7^*WT*^ and BCLC-3^*WT*^ tumors (*n* = 6) either non-treated or treated with sorafenib. **E** Metastatic spread of Huh7^*WT*^ and BCLC-3^*WT*^ cells to lung of sorafenib-treated and untreated embryos (*n* = 4). **F** Representative photographs and weights of Huh7^*mock*^ and Huh7^*hMT3*^ HCC tumors (*n* ≥ 5) either non-treated or treated with sorafenib. **H** Metastatic spread of Huh7^*mock*^ and Huh7^*hMT3*^ cells to lung of sorafenib-treated and untreated embryos (*n* = 4). Data shows mean ± SEM. *p ≤ 0.05, **p ≤ 0.005, ***p ≤ 0.001 (Student’s test, two-sided)

In follow-up experiments, *in ovo* CAM assay was carried out by applying a higher initial amount of the HCC cells. By this, we promoted the growth of a macroscopically visible tumor, and enhanced intravasation and migration in circulatory system, and extravasation into lung tissue. As shown in Fig. 5A and B, after induction, bounded HCC tumors, formed from densely populated HCC cells developed. To validate that up-regulation of hMT3 in Huh7^*hMT3*^ cells remains stable throughout the experiment, at the end-point, we excised the tumors and examined the spatial expression of hMT3. We confirmed high expression of hMT3 in Huh7^*hMT3*^ tumors. We also observed locally elevated peak intensities of hMT3 in BCLC-3^*WT*^, and practically no hMT3 expression in Huh7^*mock*^ cells (Fig. 5C). In subsequent experiments, we compared the behavior of Huh7^*WT*^ and BCLC-3^*WT*^ cells exposed to sorafenib. As expected, BCLC-3^*WT*^ tumors exhibited higher resistance compared to Huh7^*WT*^ cells (Fig. 5D). It was also found that BCLC-3^*WT*^ cells also possessed a higher efficiency of spreading to lungs (Fig. 5E). Experiments with mock- and hMT3-transfected cells confirmed induction of resistance due to hMT3 up-regulation. Although sorafenib efficiently decreased weight of Huh7^*mock*^ tumors, it exhibited literally no inhibitory effects on the weight of Huh7^*hMT3*^ tumors (Fig. 5F). In addition, hMT3-mediated sorafenib-resistance was reflected in the amounts of Huh7^*hMT3*^ cells colonizing lung tissue (Fig. 5G), which is in agreement with *ex ovo* data. These experiments provide the first evidence of the importance of hMT3 for metastatic processes of HCC cells.

### Proteome alterations due to hMT3 up-regulation

**Fig. 6:**
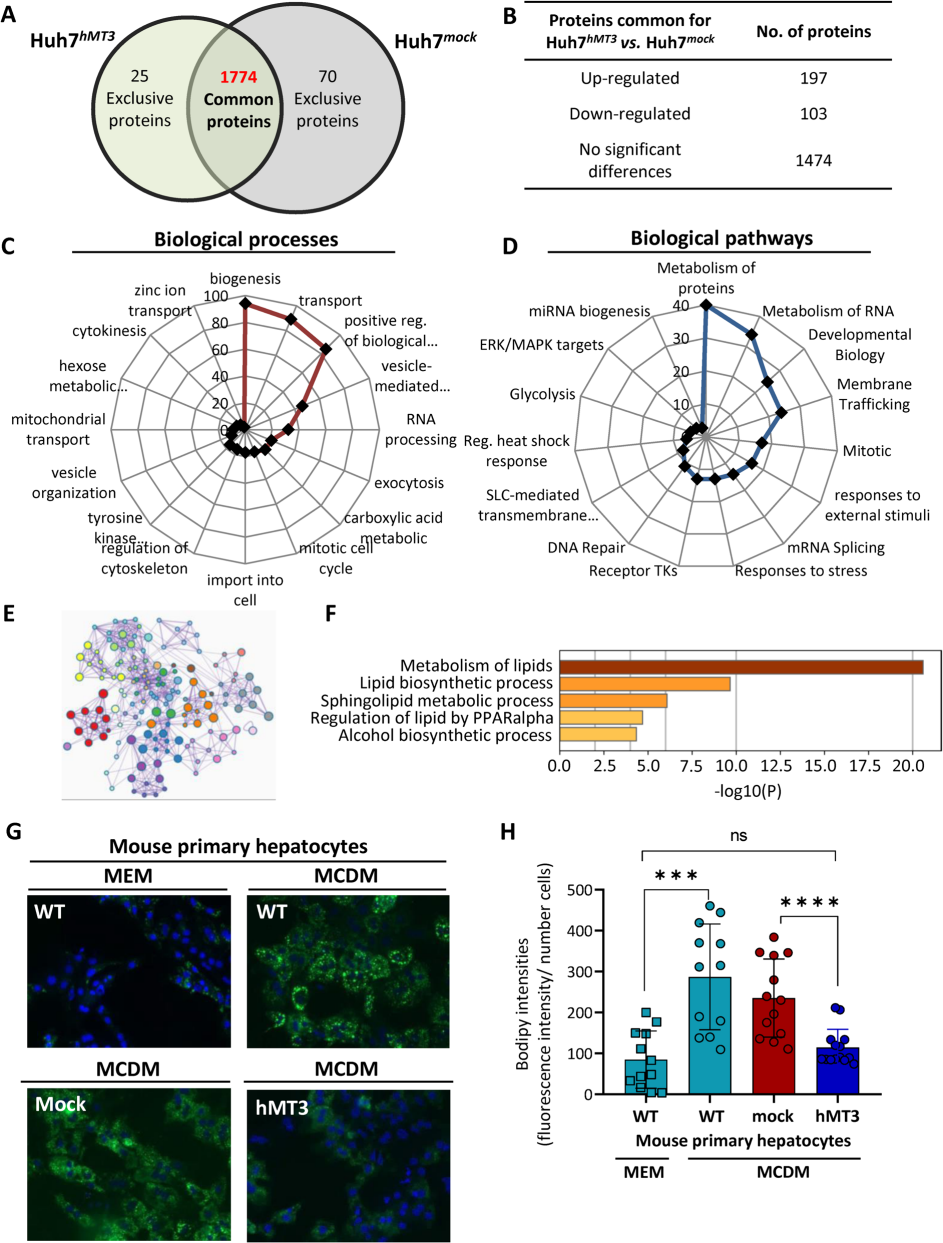
Proteomic landscape analyses of molecular mechanisms associated with up-regulation of hMT3 in Huh7 cells.** A** Venn diagram showing exclusiveness and commonness of proteins identified in HuH7^*mock*^ and Huh7^*hMT3*^ cells. **B** Numbers of proteins commonly expressed in Huh7^*mock*^ and Huh7^*hMT3*^ cells and the distribution of their deregulation. Up-regulation, fold ratio ≥ 1.5; down-regulation, fold ratio ≤ 0.5. Classification of top up-regulated proteins (including exclusively expressed proteins in Huh7^*hMT3*^) according to their (**C**) biological processes and (**D**) biological pathways. **E** Interactome showing relations of biological processes driven by down-regulated proteins in Huh7^*hMT3*^vs. Huh7^*mock*^, metabolism of lipids (orange nodes), metabolism of amino acids (blue nodes), metabolism of nucleobase-containing small molecules (red nodes), cellular response to stress (green nodes), positive regulation of lamellipodium (yellow nodes) and translation (purple nodes). **F** Top 5 clusters of enriched terms within the metabolism of lipids in Huh7^*hMT3*^. **G** Representative micrographs of BODIPY-stained lipid bodies’ content in mouse primary hepatocytes and (**H**) their relative quantification (lipid bodies/cell ± SEM, *n* ≥ 12). ***p ≤ 0.005 ****p ≤ 0.001

To investigate the quantitative differences in the proteome resulting from hMT3 up-regulation, the comparative proteomic landscapes of Huh7^*mock*^ and Huh7^*hMT3*^ were analyzed. Out of total of 1,869 identified proteins, 1,774 were commonly expressed in Huh7^*mock*^ and Huh7^*hMT3*^ cells with 25 proteins exclusively expressed in Huh7^*hMT3*^ (Fig. 6A). The full list of identified proteins as well as relevant MS data are available in Suppl. Table 3. From the dataset of commonly expressed proteins, 197, 103, and 1,474 proteins were quantitatively up-regulated (fold ratio ≥ 1.5), down-regulated (fold ratio < 0.5), or exhibited no significant deregulation (fold ratio 0.5 − 1.5, Fig. 6B, Suppl. Table 4). The up-regulated dataset contained a long list of proteins driving cell proliferation (PAFAH1B1, MAPK3, SMARCA5, POLR2H, PSME3, DCTN1, CHMP4B, TPR, RFC3, MAPK1, GSK3B, RAD9A, MAPK14, etc.), vesicles transport and membrane trafficking (CTR1A, ACTR2, ACTR3, ARPC3, CHMP4B, CHMP7, COPS3, COPS5, DCTN1, KLC4, MAN2A1, MYH9, MYO6, PAFAH1B1, PAFAH1B3, SNAP23, etc.) and metabolic shift to glycolysis (HK2, NUP205, NUP50, PGK1, SLC16A3, and TPR), which is in agreement with our previously discussed data.

Further, we focused on a dataset (*n* = 25) of proteins exclusively expressed in Huh7^*hMT3*^ cells (full list of proteins is shown in Suppl. Table 5). By classification of biological processes of proteins exclusively and up-regulated expressed in Huh7^*hMT3*^ cells, we revealed that the top scoring included transport, positive regulation of biological processes, vesicle-mediated transport, or exocytosis (Fig. 6C) indicating plausible involvement of hMT3 in lysosomal sequestration and autophagy as previously described by Ullio et al. [47]. Analysis of canonical pathways revealed that the most significantly affected pathways in Huh7^*hMT3*^ cells consist of metabolism of proteins and RNA, membrane trafficking, mitosis, and response to external stimuli/stress and glycolysis (Fig. 6D), which is in line with induced resistant phenotype of Huh7^*hMT3*^ cells. Moreover, a plethora of proteins exclusively expressed in Huh7^*hMT3*^ cells was found to be involved in VEGFA-VEGFR2 signaling (Suppl. Figure 2C), which supports the critical role of hMT3 in angiogenesis of HCC.

Interactome analysis of proteins down-regulated in Huh7^*hMT3*^ cells revealed extensive inter-connected involvement of hMT3 in the metabolism of amino acids, nucleobase-containing small molecules, and lipids (Fig. 6E). Noteworthy, the top cluster within the metabolism of lipids included lipid biosynthesis (Fig. 6F). Thus, we further examined how hMT3 up-regulation affects intracellular lipid accumulation in murine primary hepatocytes (Fig. 6G). Indeed, we confirmed that compared to *mock* transfected cells (235 ± 91) cultured in MCDM, *hMT3* overexpressing cells contained markedly lower amount of intracellular lipid droplets (114 ± 42) (Fig. 6H), therefore validating the involvement of hMT3 in the lipid metabolism of liver cells.

To delineate the possible clinical relevance of the obtained data, we further constructed the Kaplan-Meier curves for mRNA encoding proteins with expression deregulated in Huh7^*hMT3*^ cells (namely HK2 and SLC16A3 involved in glycolysis, MAPK3 involved in proliferation, PSM3 involved in proteasome activity, ACSF2 involved in lipid biosynthesis, ACAT1 involved in mitochondrial beta-oxidation, and FGA and FGB involved in formation of fibrinogen complex). Suppl. Figure 3 demonstrates that the expression of analyzed targets is associated with the prognosis of HCC-diagnosed patients, suggesting a clinical relevance of the obtained findings.

### Proteome of Huh7^hMT3^ cells shares high similarity with proteome of intrinsically sorafenib-resistant BCLC-3^WT^ cells

**Fig. 7:**
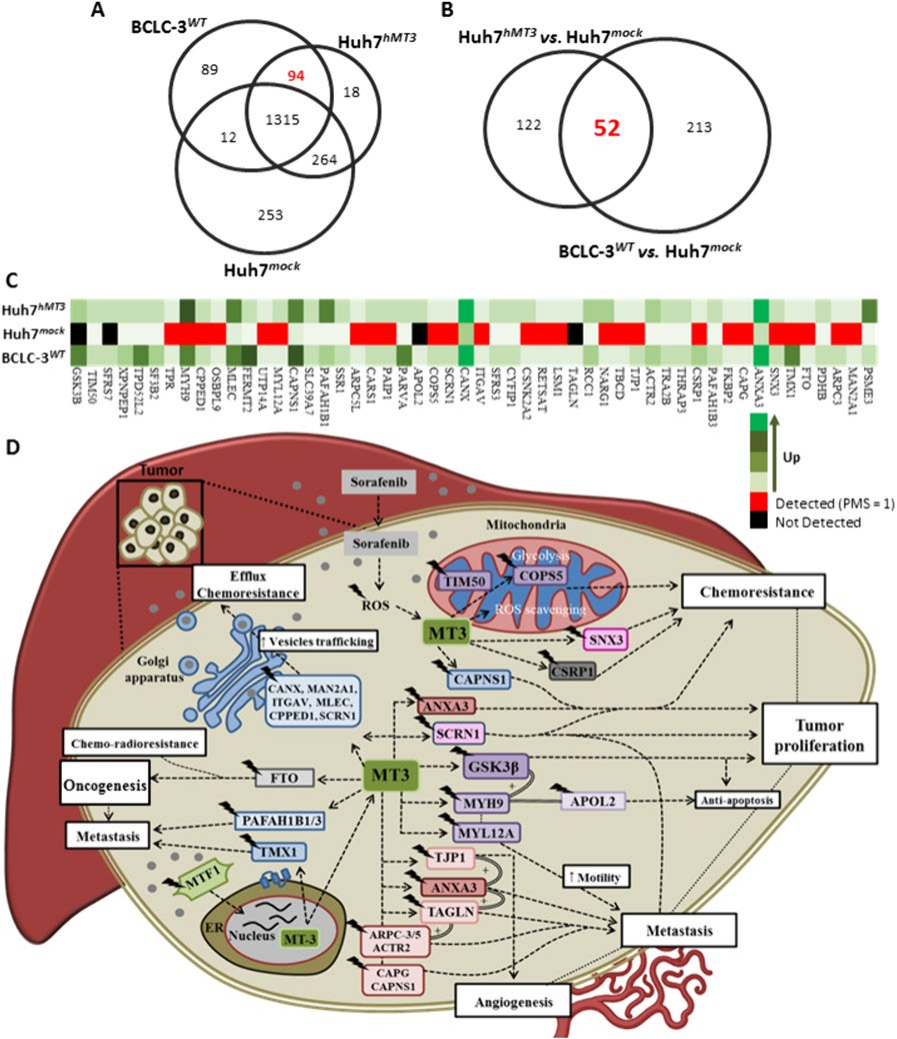
Comparative proteomic analysis of Huh7 and BCLC-3 cells. **A** Venn diagram showing exclusiveness and commonness of proteins identified in proteomes of Huh7^*mock*^, Huh7^*hMT3*^, and BCLC-3^*WT*^ cells. The red number highlights exclusive proteins shared between BCLC-3^*WT*^ and Huh7^*hMT3*^. **B** Venn diagrams showing overlap of proteins commonly expressed (*n* = 52) in HCC cells with intrinsic (BCLC-3^*WT*^) and induced (Huh7^*hMT3*^) resistance to sorafenib. **C** Protein expression-based heatmap of 52 proteins deregulated in Huh7^*hMT3*^ cells compared to Huh7^*mock*^ and BCLC-3^*WT*^. **D** Proposed mechanism of interaction and co-regulation of proteins that have been found deregulated in Huh7^*hMT3*^ and BCLC-3^*WT*^

Finally, we focused on a comparative examination of proteome landscapes of Huh7^*hMT3*^ and intrinsically sorafenib-resistant BCLC-3^*WT*^ cells (Suppl. Table 6). Datasets analyses revealed relatively high amount (*n* = 94) of overlapping up-regulated proteins shared for Huh7^*hMT3*^ and BCLC-3^*WT*^ cells but not for Huh7^*mock*^, indicating that hMT3 up-regulation is plausibly solely responsible for a proteome shift of Huh7^*hMT3*^ cells towards similarity with BCLC-3^*WT*^ cells proteome (Fig. 7A). Further, we re-analyzed the entire datasets and performed an interactive comparative analysis of protein expression patterns of HCC cells with intrinsic (BCLC-3^*WT*^vs. Huh7^*mock*^) or induced (Huh7^*hMT3*^vs. Huh7^*mock*^) resistance to sorafenib. The obtained Venn diagram highlighted 52 up- or down-regulated proteins, whose expression was common for BCLC-3^*WT*^ and Huh7^*hMT3*^ cells (Fig. 7B). These proteins were further employed to construct an expression-based heatmap depicted in Fig. 7C, which demonstrates a high similarity of both sorafenib-resistant cell lines, thus providing a list of proteins plausibly linked to a high expression of hMT3 and subsequent progression of HCC cells into a resistant phenotype. These proteins were utilized to construct a generalized scheme suggesting a multifactorial molecular mechanism responsible for sorafenib resistance induced by hMT3 up-regulation (Fig. 7D). The scheme clearly shows the involvement of hMT3 in a plethora of processes crucial for oncogenesis and resistance including vesicle trafficking and subsequent efflux of therapeutic compounds, quenching of ROS and disruption the mitochondrial metabolism, stimulation of tumor proliferation and anti-apoptotic processes and possible stimulation of angiogenesis and metastatic activity.

## Discussion

Among the main challenges of HCC therapy remains the timely suppression of metastases and development of resistance to chemotherapeutic agents [48]. The mechanisms conferring resistance to sorafenib seem to be complex, highly multifactorial, and specific for various types of cells [8, 49]. In addition to resistance, sorafenib-treated patients frequently exhibit adverse side effects that complicate therapeutic intervention [50]. Thus, a detailed understanding of sorafenib resistance is of utmost interest to identify subjects that would more likely benefit from sorafenib administration.

Therefore, we first aimed on identification of genes associated with sorafenib resistance in BCLC-3^*WT*^ cells. The transcriptomic data suggest that resistance in BCLC-3^*WT*^ cells results from complex changes at molecular and cellular levels including the ability of cells to inhibit the intracellular activity of sorafenib by either its efflux or scavenging of the ROS produced during sorafenib bioaccumulation and metabolic processing. BCLC-3^*WT*^ cells were found to possess an extensive ability to inhibit apoptotic processes to enhance its resistant phenotype, which are features frequently observed in a variety of resistant cancers [51]. To briefly comment on a few observed phenomena, it was found that BCLC-3^*WT*^ cells exhibit extensive up-regulation of SLC and ABC transporters that play a crucial role in drug absorption, distribution, elimination, accumulation, and finally, resistance. It is worth noting that several up-regulated SLC/ABC transporters have previously been associated with impaired overall survival and the development of aggressive phenotype of HCC [52–54]. Furthermore, sorafenib resistance has previously been linked to up-regulation of MT1G [12]. In line with this study, we identified that BCLC-3^*WT*^ cells exhibit pronounced up-regulation of not only MT1G but also hMT3, which is a structurally unique member of this family of proteins [24]. Subsequent analyses of HCC-diagnosed cohorts revealed that *MT3* can be found to be up-regulated in HCC tissues, and more importantly, its high expression is closely associated with poor therapeutic outcomes of HCC subjects treated with sorafenib. Thus, the obtained data suggest that *MT3*/hMT3 expression could serve a promising biomarker enabling stratification of patients before sorafenib treatment to enhance personalization of HCC therapy.

We further paid our attention to describing the behavior of HCC cells with up-regulated hMT3, and unraveling possible molecular pathways deregulated by hMT3. To the best of our knowledge, the present study is the first to document that up-regulation of hMT3 in HCC cells results in the induction of sorafenib-resistant phenotype. Among the main complications associated with HCC is a frequent progression to extrahepatic metastatic disease with primary metastatic sites in lungs, adrenal glands, bones, and brain [55, 56]. Indeed, in a CAM HCC model, we demonstrated that higher intrinsic resistance of BCLC-3^*WT*^ cells to sorafenib is associated with their higher metastatic potential to the lungs. Importantly, we have shown that hMT3 is an important driver of the extravasation, cellular migration, and intravasation of HCC cells, and unlike to Huh7^*mock*^ cells, the metastatic spread of Huh7^*hMT3*^ cells is only negligibly inhibited by sorafenib, which is consistent with the regulatory role of MTs in cancer metastasis and resistance previously reported in squamous cell carcinoma of esophagus or colorectal cancers and synchronous liver metastases [57, 58].

MTs are well-known drug binders and ROS scavengers [11]. Thus, we further analyzed the effect of up-regulation of hMT3 on cellular redox homeostasis and mitochondrial flux. HCC cells have been shown to be profoundly dependent on glycolysis for efficient ATP production [59]. Moreover, an increase in aerobic glycolysis has also been involved in the development of sorafenib resistance [60]. In fact, our data indicate that hMT3 up-regulation led to increased mitochondrial respiration and glycolysis, highlighting its importance for HCC oncogenesis. In addition, our data clearly show that hMT3 is a crucial factor influencing intracellular redox homeostasis and its up-regulation led to a protective effect against ROS-induced oxidative stress and apoptosis. This finding is in agreement with a generally accepted function of MTs in regulating baseline oxidative stress in cells [16].

To understand the molecular mechanisms affected by the up-regulation of hMT3 and subsequent progression to sorafenib-resistant phenotype, we performed extensive proteomic comparative studies revealing several interesting molecular phenomena. In the following discussion, we briefly highlight the role of up-regulated hMT3 in sorafenib resistance by interpretation of the obtained proteomic landscape.

First, we found that up-regulation of hMT3 results in up-regulation of a wide range of proteins involved in vesicle trafficking, including exocytosis which is frequently implicated in resistance of a variety of tumors to various anticancer agents [61]. It should be noted that we identified hMT3-induced up-regulation of a number of proteins from charged multivesicular body family, vacuolar ATPases, vesicle-mediated transport proteins, and others that have previously been associated with oncogenesis and resistance of HCC [62–64]. Moreover, hMT3 up-regulation exhibited stimulatory activity on the expression of proteins mediating endothelial exocytosis, exportins, and importin, which all have been identified as proteins that play a pivotal role in the resistance of HCC cells [65–67]. Thus, the obtained data clearly suggest the involvement of hMT3 in the regulation of endolysosomal functions. This is in line with a previously published study indicating that when highly expressed, MTs can be redirected to the lysosomal compartments by autophagic fluxes, where they can chelate intralysosomal redox-active metal ions and affect lysosomal membrane permeabilization to stimulate exocytic processes [47].

Chen and coworkers have shown that aerobic glycolysis is frequently increased in HCC cells, and inhibition of glycolysis metabolism in resistant cells may restore the sensitivity of cells to chemotherapy [68]. Noteworthy, up-regulated hepatic MTs have previously been associated with stimulatory effects on hepatocytes glycolysis [69]. Indeed, our proteome analyses revealed high expression of proteins within central carbon metabolism and glycolysis stimulated by hMT3 up-regulation. Among them, HK2 and GSK3B were identified in HCC cells as important factors responsible for the efficient production of energy to support accelerated growth and escape from mitochondria-associated cell death [70, 71]. In addition, GSK3B has been shown to be aberrantly activated in HCC, and its high expression is closely associated with a poor prognosis of HCC patients [72], which is in agreement with our data showing a similar phenomenon for hMT3 in particular for subjects treated with sorafenib.

It is clinically established that zinc is virtually always decreased in the HCC cells compared to normal hepatocytes [73]. Here, we demonstrate that a plethora of various zinc transporter proteins were up-regulated due to hMT3 up-regulation. We hypothesize that during oncogenesis, HCC cells develop a machinery that is able to remove intracellular zinc and inhibit its utilization as a cofactor for proteins that could potentially act as tumor suppressors (p53, ZNF471, CASZ1, and many others) [74]. This hypothesis is supported by the study by Meplan et al. who showed that up-regulation of MT2A efficiently modulates the transcriptional activity of p53 and regulates its DNA binding activity [75].

It is important to point out that current studies on HCC cells demonstrate that down-regulation of MTs may contribute to liver tumorigenesis by increasing cellular NF-κB activity [19] or through regulating Wnt/β-catenin signaling pathway [18, 23]. In contrast, there are also reports showing that up-regulation of MTs are associated with carcinogenesis, proliferation, migration, and resistance of HCC cells [11, 76]. Such contradictions clearly highlight the complexity of MTs and indicate that MTs are involved in various aspects of cellular pathophysiology, while the resulting effect is most likely sub/isoform or cell-specific. In this study, we show that hMT3 up-regulation does not negatively affect the growth of HCC cells.

Dysregulation of fatty acid metabolism, in which activity of lipid-metabolizing enzymes is an emerging hallmark of cancer cells, has previously been tightly linked to the development and progression of HCC [77]. Our proteomic analyses revealed a down-regulation of a plethora of proteins involved in metabolic pathways related to lipid biosynthesis. Interestingly, we have also functionally validated that up-regulation of hMT3 in mouse primary hepatocytes resulted in a profound inhibitory effect on the accumulation of intracellular bodies, which is in agreement with the study by Sato and coworkers who demonstrated possible protective effects of MTs against a high-fat diet, lipid accumulation and subsequent development of obesity [78]. Thus, we confirmed that besides HCC, hMT3 could be an interesting druggable target for the management of obesity and related diseases. We are eager to further work on this hypothesis.

Ferroptosis, a non-apoptotic cell death frequently occurring in HCC is characterized by the iron-dependent accumulation of lipid peroxide [79]. It has been shown that the lipid metabolic pathway and ferroptosis are highly interconnected and that FIN56 promotes ferroptosis by binding and activation with FDFT1, which is also involved in cholesterol synthesis [80]. In addition, ACBD3 has been reported to be involved in iron homeostasis and is also involved in apoptosis induction through interaction with golgin-160 caspase cleavage fragments [81]. With respect to our data, it is obvious that up-regulation of hMT3 results in the protection of HCC cells against the accumulation of lipids and induction of ferroptosis through negative regulation of expression FDFT1, ACSF2 and ACBD3, which is an obvious and important factor affecting resistance of HCC cells to sorafenib.

Another interesting finding is an exclusive expression of TAGLN triggered by hMT3 up-regulation. TAGLN was originally identified as a tumor suppressor [82]. However, recently published studies have provided contradictory results and indicate its pro-tumorigenic role in HCC cells [83]. Noteworthy, Kim and coworkers demonstrated that in HCC cells, TAGLN could regulate the expression of MTs [84]. In contrast, we show that hMT3 is able to trigger the expression of TAGLN suggesting a bidirectional regulatory feedback loop that could be used by HCC cells to confer resistance to cytostatics.

In contrast to Huh7^*mock*^ cells, BCLC-3^*WT*^ and Huh7^*hMT3*^ cell lines both exhibited a high expression of MYH9 and MYL12A, which have previously been associated with a poor prognosis of HCC patients [85]. In addition, Huh7^*hMT3*^ cells also expressed high levels of MYO6, which have been found highly expressed in human HCC specimens [86]. Together with myosins, in BCLC-3^*WT*^ and Huh7^*hMT3*^ cells, we also identified a shared up-regulation of a number of actin related proteins (namely ACTR2, ARPC3, and ARPC5L) whose expression also associates with a low survival of HCC patients [87]. Unfortunately, none of these studies examined expression patterns of MTs; however, our data provide clear evidence that hMT3 is involved in the regulation of expression of myosin and actin-related proteins, which is in line with a study by Summermatter et al. on MT1 and MT2 [88]. Thus, based on the clinical relevance of above-mentioned proteins, hMT3 could be useful as an additional biomarker for a precise prediction of the prognosis of HCC patients.

Last but not least, we also identified extensive hMT3-driven deregulation of proteins involved in proteasome activity (PSME3, SCRN1, SF3B2, and others). These proteins have been recently associated with chemo- and radioresistance and possess oncogenic activities [89, 90]. Taken together, our data provide the first evidence of the multifactorial importance of hMT3 in sorafenib-resistant phenotype of HCC cells. The provided data thoroughly confirm the hMT3-induced phenotype in Huh7^*hMT3*^ cells and demonstrate that intrinsically resistant BCLC-3^*WT*^ cells exhibit a multitude of shared molecular signatures induced by hMT3 up-regulation in Huh7^*hMT3*^ cells.

## Conclusions

The present study is the first to delineate the molecular aspects underlying sorafenib-resistance in HCC cells with high expression of hMT3. We provide clear evidence that the up-regulation of hMT3 results in increased metastatic spread from primary tumors. In addition, we demonstrated a complex dataset of proteins that are deregulated due to hMT3 up-regulation and show exceptional similarities in the proteomic landscape of Huh7^hMT3^ and intrinsically sorafenib-resistant BCLC-3^*WT*^ cells. The presented proteins and associated pathways might be further functionally validated in follow-up studies, and we believe that such analyses could result in the identification of novel druggable targets for the therapy of resistant HCC and validation of novel biomarkers that might be useful to further enhance the precision of prognostic biomarkers of HCC.

### Supplementary Information

**Additional file 1:** **Supplementary Table 1. **List of primers and their sequences used in current study. **Supplementary Figure 1. **Analysis of HCC spheroids, validation of transcriptomic data and UALCAN cohort analysis. **Supplementary Figure 2.** Fluorescence imaging of ex ovo CAM and interactome analysis. **Supplementary Figure 3.** Prognostic values of mRNA encoding proteins deregulated in Huh7^*hMT3*^ cells. **Supplementary Table 2.** Full list of up- and down-regulated mRNAs. **Supplementary Table 3.** Full list of proteins identified in Huh7^*mock*^and Huh7^*hMT3*^cells. **Supplementary Table 4.** Full list of proteins commonly expressed in Huh7^*mock*^ and Huh7^*hMT3*^ cells. **Supplementary Table 5.** Full list of proteins exclusively expressed in Huh7^*hMT3*^ cells. **Supplementary Table 6.** Full list of proteins detected in comparative proteomic analysis of BCLC-3^*WT*^ and Huh7^*hMT3*^ cells.

## Data Availability

The data that support this study are available from the corresponding authors upon reasonable request.

## Abbreviations

hMT3: Human metallothionein-3
HCC: Hepatocellular carcinoma
CAM: Chick chorioallantoic membrane
ROS: Reactive oxygen species
MCDM: Methionine/choline deficient medium
MTT: 3-(4,5-dimethylthiazol-2-yl)-2,5-diphenyltetrazolium bromide
TMB: 3,3′,5,5′-tetramethylbenzidine
qRT-PCR: Real-time quantitative reverse-transcription polymerase chain reaction
OCR: Oxygen consumption rate
ECAR: Extracellular acidification rate
7-AAD: 7-aminoactinomycin
SOD: Superoxide dismutase
CLSM: Confocal laser scanning microscopy
MALDI-MSI: Matrix-assisted laser desorption/ionization mass spectrometry imaging
MALDI-TOF/TOF: Matrix-assisted laser desorption/ionization mass spectrometry tandem time-of-flight
AGC: Automatic gain control
DEGs: Differentially expressed genes
