# Supplementary material for: Metallothionein-3 is a multifunctional driver that modulates the development of sorafenib-resistant phenotype in hepatocellular carcinoma cells
Source: Biomark Res. 2024 Apr 9;12:38. doi: 10.1186/s40364-024-00584-y (PMC11003176; doi:10.1186/s40364-024-00584-y)
Supplement: Supplementary file 1 — Additional file 1: Supplementary Table 1. List of primers and their sequences used in current study. Supplementary Figure 1. Analysis of HCC spheroids, validation of transcriptomic data and UALCAN cohort analysis. Supplementary Figure 2. Fluorescence imaging of ex ovo CAM and interactome analysis. Supplementary Figure 3. Prognostic values of mRNA encoding proteins deregulated in Huh7hMT3 cells. Supplementary Table 2. Full list of up- and down-regulated mRNAs. Supplementary Table 3. Full list of proteins identified in Huh7mockand Huh7hMT3cells. Supplementary Table 4. Full list of proteins commonly expressed in Huh7mock and Huh7hMT3 cells. Supplementary Table 5. Full list of proteins exclusively expressed in Huh7hMT3 cells. Supplementary Table 6. Full list of proteins detected in comparative proteomic analysis of BCLC-3WT and Huh7hMT3 cells. [file 40364_2024_584_MOESM1_ESM.docx]

**Metallothionein-3 is a multifunctional driver modulating development of sorafenib-resistant phenotype in hepatocellular carcinoma cells**

Miguel Angel Merlos Rodrigo^1*^, Hana Michalkova^1^, Ana Maria Jimenez Jimenez^1^, Frantisek Petrlak^1^, Tomas Do^1^, Ladislav Sivak^1^, Yazan Haddad^1^, Petra Kubickova^1^, Vivian de los Rios^2^, J. Ignacio Casal^2^, Marina Serrano-Macia^3^, Teresa C. Delgado^3^, Loreto Boix^4,5^, Jordi Bruix^4,5^, Maria L. Martinez Chantar^3,5^, Vojtech Adam^1^, Zbynek Heger^1†^

*^1^Department of Chemistry and Biochemistry, Mendel University in Brno, Zemedelska 1, CZ-613 00 Brno, Czech Republic*

*^2^Department of Cellular and Molecular Medicine and Proteomic Facility, Centro de Investigaciones Biológicas (CIB-CSIC), Ramiro de Maeztu 9, Madrid 280 40, Spain*

*^3^Liver Disease Laboratory, Center for Cooperative Research in Biosciences (CIC bioGUNE), Basque Research and Technology Alliance (BRTA), Bizkaia Technology Park, Building 801A, 48160 Derio, Spain*

*^4^Barcelona-Clínic Liver Cancer Group, Liver Unit, Institut d'Investigacions Biomèdiques August Pi I Sunyer, Barcelona, Catalonia, Spain.*

*^5^Centro de Investigación Biomédica en Red de Enfermedades Hepáticas y Digestivas (CIBERehd), Instituto de Salud Carlos III, Madrid, Spain*

***Corresponding authors**

^†^Zbynek Heger, Department of Chemistry and Biochemistry, Mendel University in Brno, Zemedelska 1, CZ-613 00 Brno, Czech Republic; E-mail: [zbynek.heger@mendelu.cz](mailto:zbynek.heger@mendelu.cz); phone: +420-5-4513-3350

^*^Miguel Angel Merlos Rodrigo, Department of Chemistry and Biochemistry, Mendel University in Brno, Zemedelska 1, CZ-613 00 Brno, Czech Republic; E-mail: [miguel.rodrigo@mendelu.cz](mailto:miguel.rodrigo@mendelu.cz); phone: +420-5-4513-3350

**Supplementary Table 1.** List of primers employed for validation of *hMT3* expression and selected proteomic results by qRT-PCR. The primer was designed by Primer3web (version 4.1.0) and validated by Primer-BLAST (NCBI).

| **Primers for qRT-PCR of hMT3 and** **validation of proteomic** **data** | | | |
| --- | --- | --- | --- |
| **Gene** | **Symbol** | **Primer pair**  **(5´-3´)*** | **T*_m_*** |
| Metallothionein 3 | *MT3* | TCGACATGGACCCTGAGACC  CACACTTCTCACACTCCGCA | 60.0 |
| Acyl-CoA synthetase family member 2 | *ACSF2* | CTGTCTACGTCGGGATGCTG  CTCTGGAACTGAGGAAGCGG | 60.0 |
| Annexin A3 | *ANXA3* | CTTCGCTCGCAGTTTGTTCG  CTCGGTGTCCAACCCAGATA | 59.9 |
| Glycogen synthase kinase 3 beta | *GSK3B* | TCCAGGGGATAGTGGTGTGG  GGGGTCGGAAGACCTTAGTCC | 60.0 |
| Hepatocyte nuclear factor 1-beta | *HNF1B* | AACACAACATCCCCCAGAGG  CTGGACTGTCTGGTTGAATTGTCG | 60.1 |
| Myosin Heavy Chain 9 | *MYH9* | TCGACCAGATCAACACCGAC  ACTTGGACTTGACAGTGCCC | 60.0 |
| Transgelin 2 | *TAGLN2* | TCCCAACTGGTTCCCTAAGAAAT  GATCAGAGGATCTGGCGTGG | 60.0 |
| Glyceraldehyde-3-Phosphate Dehydrogenase | *GAPDH* | GCCGTCTAGAAAAACCTGCC  AGTGGTCGTTGAGGGCAATG | 59.9 |

*Upper and lower sequences represent forward and reverse primers, respectively; T*_m_* is the melting temperature of a specific product.

**
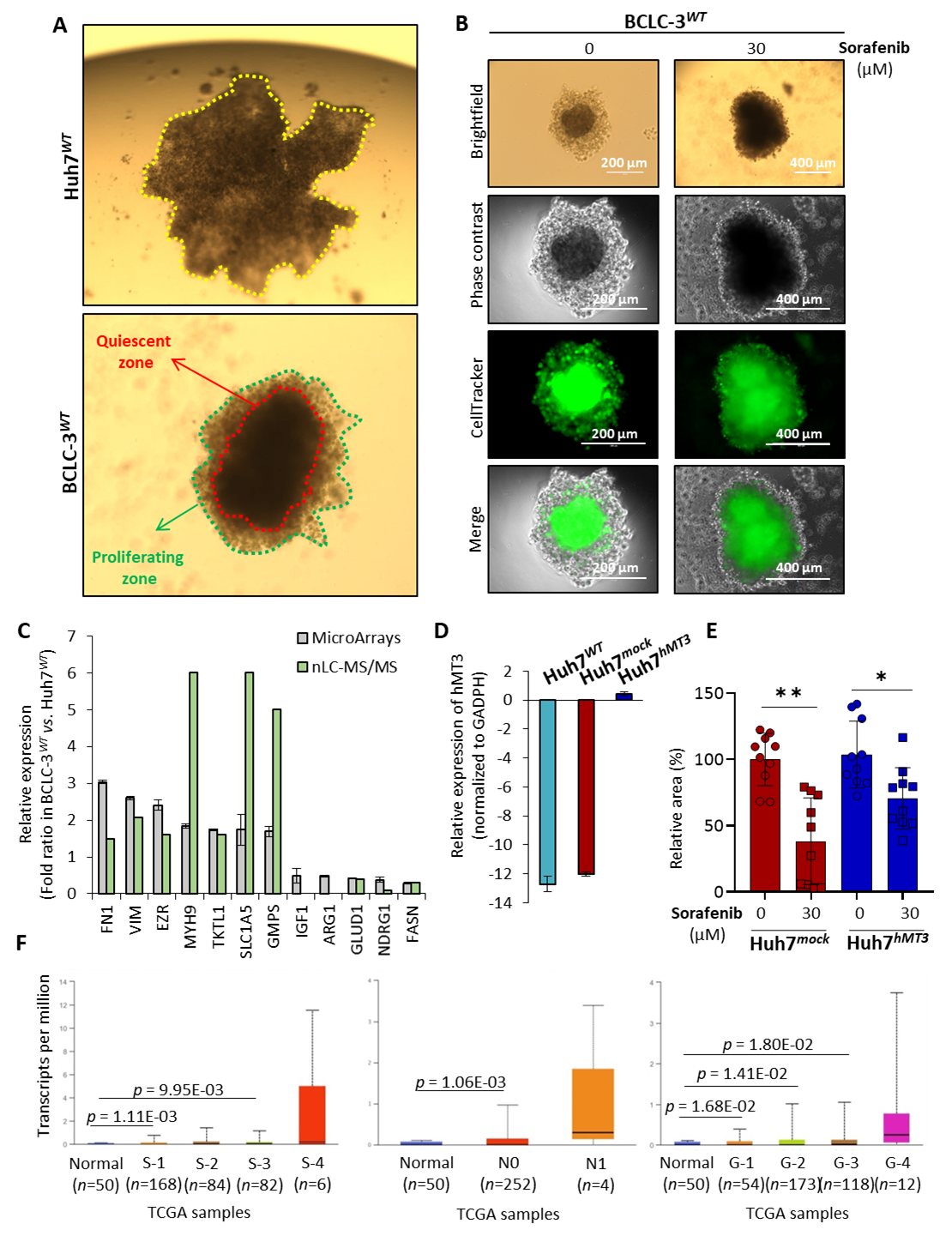
**

**Supplementary Figure 1:** (**A**) Huh7*^WT^* and BCLC-3*^WT^* spheroids morphology and (**B**) the anticancer efficacy of sorafenib against BCLC-3*^WT^* spheroid. Before spheroids formation by the hanging drop method, cells were pre-labeled using CellTracker Green. (**C**) Cross-validation of expression of selected deregulated proteins as determined by proteomics and cDNA microarray. Threshold values for up- and down-regulation were set to BCLC-3*^WT^* cells median protein expression fold ratio (PMS) ≥1.5 and ≤0.5, respectively compared to Huh7*^WT^* cells. (**D**) Baseline expression of hMT3 in Huh7*^WT^*, Huh7*^mock^*, and Huh7*^hMT3^* cells normalized to GADPH. (**E**) Quantification of relative area (%) of the spheroids (*n* = 10) either treated or non-treated with sorafenib. Data show mean ± SEM. **p* ≤ 0.01, ***p* ≤ 0.001 (Student’s test, two-sided). (**F**) Expression of hMT3 in HCC patients stratified by different stages (from S-1 to S-4), nodal metastasis status (N0 and N1) and tumor grades (from G-1 to G-4) compared to hMT3 expression found in normal liver tissue. Data were gathered and analyzed using UALCAN analysis platform (<http://ualcan.path.uab.edu/>).

**
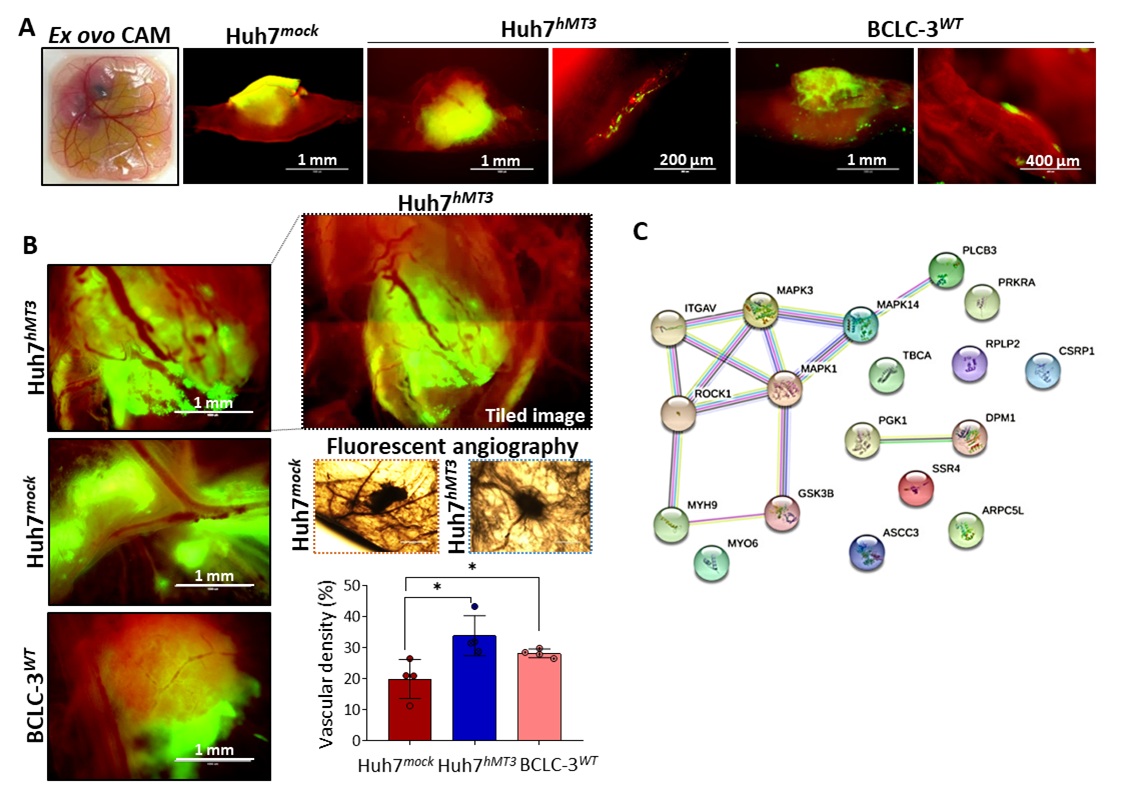
**

**Supplementary Figure 2.** (**A**) Representative fluorescence micrographs of the cross-section (transversal view) of the microtumors developed in *ex ovo* CAM assay. (**B**) Representative digital photographs of HCC tumors and adjacent angiogenesis produced in *ex ovo* CAM assay. Tiled image, 4 micrographs (×4 - 1 mm). For fluorescence imaging, the cells were pre-labeled with CellTracker Green while blood vessels were stained using rhodamine-labeled LCA. Vascular density (%) was quantified by ImageJ software (Vessel Analysis plugin). Data are expressed as mean ± SEM (*n* = 3). **p* ≤ 0.05. (**C**) STRING interactome network showing the interactions of proteins, which were found exclusively expressed and up-regulated in Huh7*^hMT3^* cells compared to Huh7*^mock^*cells and are involved in VEGFA-VEGFR2 signaling pathway involved in agiogenesis.

**
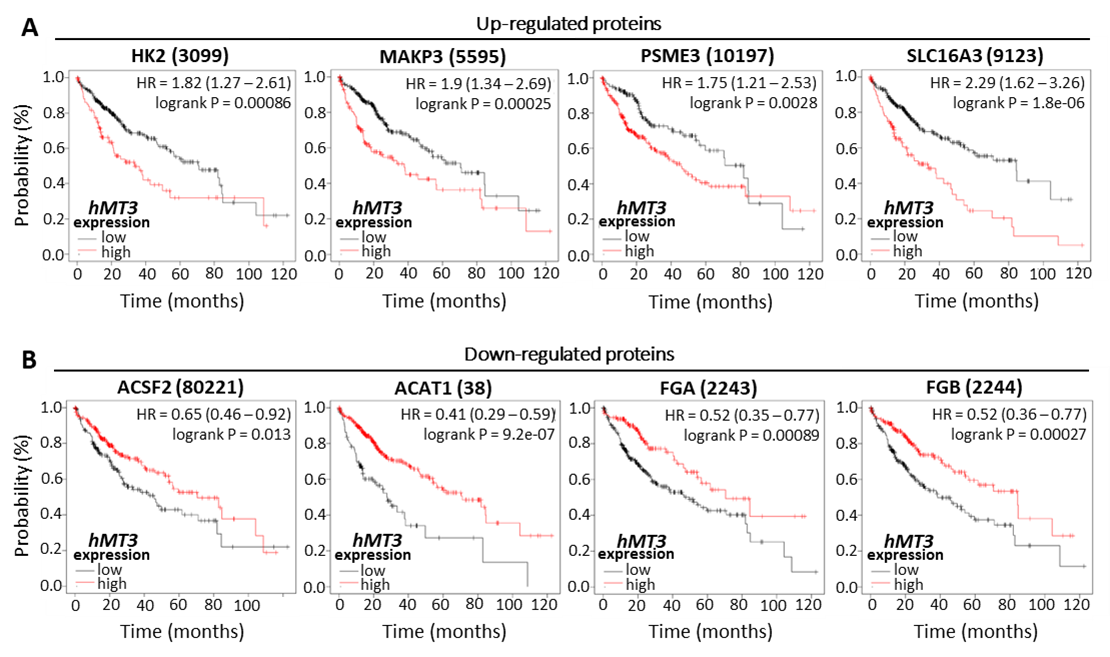
**

**Supplementary Figure 3:** Prognostic values of mRNA encoding proteins with expression deregulated due to hMT3 up-regulation (dataset RNA-SeqID: 1633, *n* = 364 subjects with HCC). The survival curves revealed that in patients suffering from HCC, (**A**) high expression of HK2, PSME3, MAPK3, and SLC16A3, and (**B**) low expression of ACSF2, ACAT1, FGA, and FGB indicate a worse prognosis.

**Supplementary Table 2**: The full list of mRNAs that were found up- or down-regulated in BCLC-3*^WT^* cells transcriptome compared with transcriptome of Huh7*^WT^* cells. *FR, fold ratio.

| **Up-regulated** | | | | | | |
| --- | --- | --- | --- | --- | --- | --- |
| Gene name | Symbol | NCBI | | FR* | p-value | |
| Fibronectin 1 | *FN1* | NM_212476 | | 3.04 | 4.216E-07 | |
| Meiotic recombination protein DMC1/LIM15 homolog | *DMC1* | NM_007068 | | 3.13 | 4.525E-08 | |
| Eukaryotic translation initiation factor 2-alpha kinase 3 | *EIF2AK3* | NM_004836 | | 3.11 | 0.0007 | |
| Phosphatidylinositol 4,5-bisphosphate 3-kinase catalytic subunit alpha isoform | *PIK3CA* | NM_006218 | | 3.06 | 2.997E-05 | |
| Structural maintenance of chromosomes protein 6 | *SMC6* | NM_001142286 | | 2.90 | 0.00027 | |
| Transforming growth factor beta-2 | *TGFB2* | NM_003238 | | 2.69 | 0.0035 | |
| TOR Signaling Pathway Regulator | *TIPRL* | NM_152902 | | 2.68 | 0.0004 | |
| Vimentin | *VIM* | NM_003380 | | 2.64 | 9.184E-11 | |
| Integrin beta-3 | *ITGB3* | NM_000212 | | 2.61 | 0.0095 | |
| Autophagy-related protein 9A | *ATG9A* | NM_001077198 | | 2.50 | 5.52E-08 | |
| Caveolin-1 | *CAV1* | NM_001172896 | | 2.45 | 8.023E-06 | |
| Receptor expression-enhancing protein 5 | *REEP5* | NM_005669 | | 2.43 | 3.164E-05 | |
| Rho-associated protein kinase 1 | *ROCK1* | NM_005406 | | 2.40 | 7.513E-07 | |
| Cystine/glutamate transporter | *SLC7A11* | NM_014331 | | 2.36 | 0.0038 | |
| Ezrin | *EZR* | NM_003379 | | 2.33 | 8.05E-10 | |
| Myosin-9 | *MYH9* | NM_002473 | | 2.29 | 5.813E-07 | |
| Pro-epidermal growth factor | *EGF* | NM_001178131 | | 2.28 | 8.482E-11 | |
| NACHT, LRR and PYD domains-containing protein 2 | *NLRP2* | NM_001174082 | | 2.24 | 4.3E-06 | |
| Ras-related C3 botulinum toxin substrate 1 | *RAC1* | NM_006908 | | 2.21 | 0.00033 | |
| Bifunctional methylenetetrahydrofolate dehydrogenase/cyclohydrolase | *MTHFD2* | NM_006636 | | 2.18 | 4.623E-05 | |
| Baculoviral IAP repeat containing 5 | *BIRC5* | NM_001168 | | 2.17 | 3.86E-07 | |
| Metallothionein-3 | *MT3* | NM_005954 | | 2.16 | 1.422E-07 | |
| Vascular endothelial growth factor C | *VEGFC* | NM_005429 | | 2.12 | 0.0004 | |
| Breast cancer type 1 susceptibility protein | *BRCA1* | NM_007298 | | 2.11 | 2.873E-07 | |
| Metallothionein-1A | *MT1A* | NM_005946 | | 2.10 | 8.697E-05 | |
| Sodium-Dependent Neutral Amino Acid Transporter Type 2 | *SLC1A5* | NM_001145145 | | 2.07 | 1.183E-06 | |
| Cyclin-dependent kinase inhibitor 2A | *CDKN2A* | NM_000077 | | 2.06 | 0.00026 | |
| Zinc transporter 1 | *SLC30A1* | NM_021194 | | 2.04 | 0.0014 | |
| Metal regulatory transcription factor 1 | *MTF1* | NM_005955 | | 2.01 | 1.17E-07 | |
| Insulin-like growth factor-binding protein 4 | *IGFBP4* | NM_001552 | | 2.00 | 7.374E-05 | |
| Metallothionein-1G | *MT1G* | NM_001301267 | | 1.92 | 1.286E-05 | |
| Transketolase-like protein 1 | *TKTL1* | NM_001145934 | | 1.90 | 6.765E-10 | |
| GTPase KRAS | *KRAS* | NM_033360 | | 1.90 | 3.15E-06 | |
| Transcription factor p65 | *RELA* | NM_021975 | | 1.80 | 4.309E-05 | |
| Zinc transporter ZIP2 | *SLC39A2* | NM_001256588 | | 1.79 | 0.00036 | |
| Neuropilin-1 | *NRP1* | NM_001024628 | | 1.78 | 9.844E-05 | |
| Killer cell lectin-like receptor subfamily C, member 3 | *KLRC3* | NM_002261 | | 1.77 | 1.313E-05 | |
| Matrix metallopeptidase 2 | *MMP2* | NM_001127891 | | 1.76 | 1.5409E-08 | |
| Collagen alpha-1(XVIII) chain | *COL18A1* | NM_130445 | | 1.75 | 0.00044 | |
| ATP-binding cassette sub-family B member 5 | *ABCB5* | NM_178559 | | 1.73 | 0.00208 | |
| CD44 antigen | *CD44* | NM_001001390 | | 1.72 | 5.661E-05 | |
| Phosphatase and tensin homolog | *PTEN* | NM_000314 | | 1.72 | 3.848E-05 | |
| Rho-associated protein kinase 2 | *ROCK2* | NM_004850 | | 1.69 | 3.39E-08 | |
| Bone morphogenetic protein 2 | *BMP2* | NM_001200 | | 1.69 | 1.044E-05 | |
| Cathepsin B | *CTSB* | NM_147781 | | 1.68 | 5.836E-07 | |
| Zinc transporter 5 | *SLC30A5* | NM_024055 | | 1.67 | 0.0012 | |
| Myosin light chain kinase | *MYLK* | NM_053031 | | 1.64 | 1.332E-06 | |
| GMP synthase [glutamine-hydrolyzing] | *GMPS* | NM_003875 | | 1.62 | 0.0023 | |
| Transforming growth factor beta-3 | *TGFB3* | NM_003239 | | 1.61 | 5.687E-06 | |
| Platelet-derived growth factor subunit A | *PDGFA* | NM_033023 | | 1.59 | 1.628E-06 | |
| Chemokine-like receptor 1 | *CMKLR1* | NM_001142344 | | 1.58 | 0.00128 | |
| Disintegrin and metalloproteinase domain-containing protein 17 | *ADAM17* | NM_003183 | | 1.58 | 4.159E-05 | |
| Zinc transporter 4 | *SLC30A4* | NM_013309 | | 1.57 | 9.929E-05 | |
| Zinc transporter ZIP6 | *SLC39A6* | NM_001099406 | | 1.57 | 0.000302 | |
| RAC-alpha serine/threonine-protein kinase | *AKT1* | NM_001014431 | | 1.57 | 5.495E-08 | |
| Protein lin-28 homolog A | *LIN28A* | NM_024674 | | 1.57 | 5.36E-05 | |
| Kinase insert domain receptor | *KDR* | NM_002253 | | 1.55 | 8.013E-09 | |
| Transforming growth factor beta-1 | *TGFB1* | NM_000660 | | 1.55 | 7.969E-08 | |
| Ubiquitin-like-conjugating enzyme ATG3 | *ATG3* | NM_022488 | | 1.55 | 1.344E-05 | |
| Zinc transporter SLC39A7 | *SLC39A7* | NM_001077516 | | 1.55 | 1.688E-08 | |
| Adenomatous polyposis coli protein | *APC* | NM_001127510 | | 1.54 | 8.358E-06 | |
| Thy-1 membrane glycoprotein | *THY1* | NM_006288 | | 1.53 | 2.243E-05 | |
| Zinc transporter 9 | *SLC30A9* | NM_006345 | | 1.53 | 4.031E-06 | |
| C-X-C Motif Chemokine Ligand 1 | *CXCL1* | NM_001511 | | 1.51 | 0.00041 | |
| **Down-regulated** | | | | | | |
| Gene name | Symbol | NCBI | FR* | | | p-value |
| Protein jagged-1 | *JAG1* | NM_000214 | 0.54 | | | 1.447E-10 |
| Neuropilin-2 | *NRP2* | NM_201264 | 0.53 | | | 0.0235 |
| Caspase-8 | *CASP8* | NM_033356 | 0.51 | | | 3.44E-07 |
| Arginase-1 | *ARG1* | NM_001244438 | 0.51 | | | 4.42E-05 |
| DNA ligase 4 | *LIG4* | NM_001098268 | 0.50 | | | 0.00113 |
| Myosin-10 | *MYH10* | NM_001256095 | 0.50 | | | 0.00055 |
| Argininosuccinate lyase | *ASL* | NM_000048 | 0.50 | | | 1.701E-06 |
| Integrin alpha-1 | *ITGA1* | NM_181501 | 0.50 | | | 3.277E-07 |
| Tumor necrosis factor receptor superfamily member 10B | *TNFRSF10B* | NM_147187 | 0.49 | | | 3.72E-06 |
| Argininosuccinate synthase | *ASS1* | NM_000050 | 0.49 | | | 1.468E-10 |
| Granulocyte-macrophage colony-stimulating factor | *CSF2* | NM_000758 | 0.48 | | | 3.132E-07 |
| Cadherin-2 | *CDH2* | NM_001792 | 0.47 | | | 0.00128 |
| Ataxin-1 | *ATXN1* | NM_001128164 | 0.47 | | | 2.863E-08 |
| Insulin-like growth factor I | *IGF1* | NM_001111285 | 0.46 | | | 0.00161 |
| Dynactin subunit 4 | *DCTN4* | NM_001135644 | 0.46 | | | 2.807E-05 |
| Mediator of DNA damage checkpoint protein 1 | *MDC1* | NM_014641 | 0.46 | | | 4.67E-05 |
| Zinc transporter ZIP1 | *SLC39A1* | NM_001271959 | 0.45 | | | 1.31E-08 |
| Vascular endothelial growth factor A | *VEGFA* | NM_001025369 | 0.45 | | | 3.08E-05 |
| Deoxycytidine kinase | *DCK* | NM_000788 | 0.45 | | | 0.0012 |
| Replication protein A 70 kDa DNA-binding subunit | *RPA1* | NM_002945 | 0.41 | | | 0.0052 |
| Cellular tumor antigen p53 | *TP53* | NM_000546 | 0.39 | | | 1.233E-07 |
| Double-strand break repair protein MRE11 | *MRE11A* | NM_005591 | 0.37 | | | 1.481E-05 |
| Protein NDRG1 | *NDRG1* | NM_001258433 | 0.37 | | | 1.129E-09 |
| Glutamate dehydrogenase 1 | *GLUD1* | NM_001318905 | 0.37 | | | 0.0196 |
| Tyrosyl-DNA phosphodiesterase 2 | *TDP2* | NM_016614 | 0.35 | | | 0.000307 |
| BH3-interacting domain death agonist | *BID* | NM_001196 | 0.35 | | | 2.159E-06 |
| Retinoblastoma-associated protein | *RB1* | NM_000321 | 0.34 | | | 7.198E-06 |
| Pyrroline-5-carboxylate reductase 1 | *PYCR1* | NM_006907 | 0.34 | | | 1.313E-07 |
| Protein kinase C alpha type | *PRKCA* | NM_002737 | 0.31 | | | 1.611E-12 |
| Pyruvate carboxylase | *PC* | NM_022172 | 0.28 | | | 1.578E-14 |
| Fatty acid synthase | *FASN* | NM_004104 | 0.28 | | | 2.21E-15 |
| Unconventional myosin-Ib | *MYO1B* | NM_001161819 | 0.28 | | | 3.606E-05 |
| Prostaglandin G/H synthase 2 | *PTGS2* | NM_000963 | 0.23 | | | 6.23E-18 |
| **References Genes** | | | | | | |
| Gene name | Symbol | NCBI | FR* | | | p-value |
| Actin Beta | *ACTB* | NM_00110 | 1.07 | | | 0.348 |
| Proteasome 20S Subunit Beta 2 | *PSMB2* | NM_002794 | 1.05 | | | 0.123 |
| Small Nuclear Ribonucleoprotein D3 Polypeptide | *SNRPD3* | NM_004175 | 1.05 | | | 0.414 |
| Valosin Containing Protein | *VCP* | NM_007126 | 1.06 | | | 0.276 |
| ER Membrane Protein Complex Subunit 7 | *EMC7* | NM_020154 | 1.02 | | | 0.394 |
| Actin Beta | *ACTB* | NM_00110 | 1.07 | | | 0.348 |
| Proteasome 20S Subunit Beta 2 | *PSMB2* | NM_002794 | 1.05 | | | 0.123 |
| Small Nuclear Ribonucleoprotein D3 Polypeptide | *SNRPD3* | NM_004175 | 1.05 | | | 0.414 |
| Valosin Containing Protein | *VCP* | NM_007126 | 1.06 | | | 0.276 |

**Supplementary Table 3:** The full list of proteins identified in Huh7*^mock^* and Huh7*^hMT3^* cells. For each protein, the following information is given: accession number (Swiss-Prot), theoretical molecular weight, pI, amino acids amount, and number of peptides used for identification and percentage of sequence coverage. The universally accepted requirement is to have identified the proteins with the least two peptides (Σ Unique Peptides).

| **Accession** | **Description** | **Unique Peptides** | **PSM**  **Huh7**  ***^mock^*** | **PSM**  **Huh7**  ***^hMT3^*** | **AA** | **MW [kDa]** | **pI** |
| --- | --- | --- | --- | --- | --- | --- | --- |
| Q13813 | Spectrin alpha chain, brain OS=Homo sapiens GN=SPTAN1 PE=1 SV=3 - [SPTA2_HUMAN] | 76 | 84 | 80 | 2472 | 284.4 | 5.35 |
| Q00610 | Clathrin heavy chain 1 OS=Homo sapiens GN=CLTC PE=1 SV=5 - [CLH1_HUMAN] | 58 | 69 | 79 | 1675 | 191.5 | 5.69 |
| Q01082 | Spectrin beta chain, brain 1 OS=Homo sapiens GN=SPTBN1 PE=1 SV=2 - [SPTB2_HUMAN] | 58 | 58 | 67 | 2364 | 274.4 | 5.57 |
| P49327 | Fatty acid synthase OS=Homo sapiens GN=FASN PE=1 SV=3 - [FAS_HUMAN] | 53 | 64 | 66 | 2511 | 273.3 | 6.44 |
| Q9Y490 | Talin-1 OS=Homo sapiens GN=TLN1 PE=1 SV=3 - [TLN1_HUMAN] | 50 | 53 | 60 | 2541 | 269.6 | 6.07 |
| P13639 | Elongation factor 2 OS=Homo sapiens GN=EEF2 PE=1 SV=4 - [EF2_HUMAN] | 40 | 83 | 86 | 858 | 95.3 | 6.83 |
| Q14697 | Neutral alpha-glucosidase AB OS=Homo sapiens GN=GANAB PE=1 SV=3 - [GANAB_HUMAN] | 35 | 49 | 51 | 944 | 106.8 | 6.14 |
| P10809 | 60 kDa heat shock protein, mitochondrial OS=Homo sapiens GN=HSPD1 PE=1 SV=2 - [CH60_HUMAN] | 34 | 79 | 98 | 573 | 61.0 | 5.87 |
| P21333 | Filamin-A OS=Homo sapiens GN=FLNA PE=1 SV=4 - [FLNA_HUMAN] | 34 | 30 | 33 | 2647 | 280.6 | 6.06 |
| P42704 | Leucine-rich PPR motif-containing protein, mitochondrial OS=Homo sapiens GN=LRPPRC PE=1 SV=3 - [LPPRC_HUMAN] | 33 | 29 | 36 | 1394 | 157.8 | 6.13 |
| P55072 | Transitional endoplasmic reticulum ATPase OS=Homo sapiens GN=VCP PE=1 SV=4 - [TERA_HUMAN] | 32 | 53 | 59 | 806 | 89.3 | 5.26 |
| P14625 | Endoplasmin OS=Homo sapiens GN=HSP90B1 PE=1 SV=1 - [ENPL_HUMAN] | 32 | 42 | 46 | 803 | 92.4 | 4.84 |
| P11021 | 78 kDa glucose-regulated protein OS=Homo sapiens GN=HSPA5 PE=1 SV=2 - [GRP78_HUMAN] | 31 | 45 | 51 | 654 | 72.3 | 5.16 |
| P55060 | Exportin-2 OS=Homo sapiens GN=CSE1L PE=1 SV=3 - [XPO2_HUMAN] | 31 | 37 | 43 | 971 | 110.3 | 5.77 |
| P55157 | Microsomal triglyceride transfer protein large subunit OS=Homo sapiens GN=MTTP PE=1 SV=1 - [MTP_HUMAN] | 31 | 34 | 37 | 894 | 99.3 | 8.41 |
| P14618 | Pyruvate kinase isozymes M1/M2 OS=Homo sapiens GN=PKM2 PE=1 SV=4 - [KPYM_HUMAN] | 30 | 58 | 63 | 531 | 57.9 | 7.84 |
| O43707 | Alpha-actinin-4 OS=Homo sapiens GN=ACTN4 PE=1 SV=2 - [ACTN4_HUMAN] | 30 | 63 | 62 | 911 | 104.8 | 5.44 |
| Q86VP6 | Cullin-associated NEDD8-dissociated protein 1 OS=Homo sapiens GN=CAND1 PE=1 SV=2 - [CAND1_HUMAN] | 30 | 38 | 37 | 1230 | 136.3 | 5.78 |
| O75369 | Filamin-B OS=Homo sapiens GN=FLNB PE=1 SV=2 - [FLNB_HUMAN] | 30 | 33 | 35 | 2602 | 278.0 | 5.73 |
| P06733 | Alpha-enolase OS=Homo sapiens GN=ENO1 PE=1 SV=2 - [ENOA_HUMAN] | 29 | 81 | 82 | 434 | 47.1 | 7.39 |
| P11142 | Heat shock cognate 71 kDa protein OS=Homo sapiens GN=HSPA8 PE=1 SV=1 - [HSP7C_HUMAN] | 29 | 52 | 60 | 646 | 70.9 | 5.52 |
| P11498 | Pyruvate carboxylase. mitochondrial OS=Homo sapiens GN=PC PE=1 SV=2 - [PYC_HUMAN] | 29 | 26 | 37 | 1178 | 129.6 | 6.84 |
| Q7KZF4 | Staphylococcal nuclease domain-containing protein 1 OS=Homo sapiens GN=SND1 PE=1 SV=1 - [SND1_HUMAN] | 28 | 30 | 27 | 910 | 101.9 | 7.17 |
| P07237 | Protein disulfide-isomerase OS=Homo sapiens GN=P4HB PE=1 SV=3 - [PDIA1_HUMAN] | 27 | 47 | 44 | 508 | 57.1 | 4.87 |
| P53621 | Coatomer subunit alpha OS=Homo sapiens GN=COPA PE=1 SV=2 - [COPA_HUMAN] | 27 | 22 | 27 | 1224 | 138.3 | 7.66 |
| P00352 | Retinal dehydrogenase 1 OS=Homo sapiens GN=ALDH1A1 PE=1 SV=2 - [AL1A1_HUMAN] | 26 | 50 | 57 | 501 | 54.8 | 6.73 |
| P08670 | Vimentin OS=Homo sapiens GN=VIM PE=1 SV=4 - [VIME_HUMAN] | 26 | 39 | 39 | 466 | 53.6 | 5.12 |
| P13010 | ATP-dependent DNA helicase 2 subunit 2 OS=Homo sapiens GN=XRCC5 PE=1 SV=3 - [KU86_HUMAN] | 25 | 29 | 34 | 732 | 82.7 | 5.81 |
| P02545 | Lamin-A/C OS=Homo sapiens GN=LMNA PE=1 SV=1 - [LMNA_HUMAN] | 25 | 28 | 24 | 664 | 74.1 | 7.02 |
| Q08211 | ATP-dependent RNA helicase A OS=Homo sapiens GN=DHX9 PE=1 SV=4 - [DHX9_HUMAN] | 25 | 29 | 30 | 1270 | 140.9 | 6.84 |
| P00338 | L-lactate dehydrogenase A chain OS=Homo sapiens GN=LDHA PE=1 SV=2 - [LDHA_HUMAN] | 24 | 52 | 44 | 332 | 36.7 | 8.27 |
| O60701 | UDP-glucose 6-dehydrogenase OS=Homo sapiens GN=UGDH PE=1 SV=1 - [UGDH_HUMAN] | 24 | 35 | 35 | 494 | 55.0 | 7.12 |
| P31939 | Bifunctional purine biosynthesis protein PURH OS=Homo sapiens GN=ATIC PE=1 SV=3 - [PUR9_HUMAN] | 24 | 21 | 25 | 592 | 64.6 | 6.71 |
| P38646 | Stress-70 protein. mitochondrial OS=Homo sapiens GN=HSPA9 PE=1 SV=2 - [GRP75_HUMAN] | 24 | 27 | 38 | 679 | 73.6 | 6.16 |
| P22314 | Ubiquitin-like modifier-activating enzyme 1 OS=Homo sapiens GN=UBA1 PE=1 SV=3 - [UBA1_HUMAN] | 24 | 36 | 30 | 1058 | 117.8 | 5.76 |
| P49588 | Alanyl-tRNA synthetase. cytoplasmic OS=Homo sapiens GN=AARS PE=1 SV=2 - [SYAC_HUMAN] | 24 | 30 | 28 | 968 | 106.7 | 5.53 |
| P05091 | Aldehyde dehydrogenase. mitochondrial OS=Homo sapiens GN=ALDH2 PE=1 SV=2 - [ALDH2_HUMAN] | 23 | 27 | 32 | 517 | 56.3 | 7.05 |
| P06576 | ATP synthase subunit beta. mitochondrial OS=Homo sapiens GN=ATP5B PE=1 SV=3 - [ATPB_HUMAN] | 23 | 38 | 48 | 529 | 56.5 | 5.40 |
| P08238 | Heat shock protein HSP 90-beta OS=Homo sapiens GN=HSP90AB1 PE=1 SV=4 - [HS90B_HUMAN] | 23 | 65 | 78 | 724 | 83.2 | 5.03 |
| P12956 | ATP-dependent DNA helicase 2 subunit 1 OS=Homo sapiens GN=XRCC6 PE=1 SV=2 - [KU70_HUMAN] | 23 | 18 | 28 | 609 | 69.8 | 6.64 |
| Q07065 | Cytoskeleton-associated protein 4 OS=Homo sapiens GN=CKAP4 PE=1 SV=2 - [CKAP4_HUMAN] | 23 | 26 | 28 | 602 | 66.0 | 5.92 |
| Q9NY33 | Dipeptidyl peptidase 3 OS=Homo sapiens GN=DPP3 PE=1 SV=2 - [DPP3_HUMAN] | 23 | 28 | 35 | 737 | 82.5 | 5.10 |
| P51659 | Peroxisomal multifunctional enzyme type 2 OS=Homo sapiens GN=HSD17B4 PE=1 SV=3 - [DHB4_HUMAN] | 23 | 19 | 21 | 736 | 79.6 | 8.84 |
| P25705 | ATP synthase subunit alpha. mitochondrial OS=Homo sapiens GN=ATP5A1 PE=1 SV=1 - [ATPA_HUMAN] | 23 | 29 | 36 | 553 | 59.7 | 9.13 |
| O75533 | Splicing factor 3B subunit 1 OS=Homo sapiens GN=SF3B1 PE=1 SV=3 - [SF3B1_HUMAN] | 23 | 20 | 29 | 1304 | 145.7 | 7.09 |
| P46940 | Ras GTPase-activating-like protein IQGAP1 OS=Homo sapiens GN=IQGAP1 PE=1 SV=1 - [IQGA1_HUMAN] | 23 | 24 | 27 | 1657 | 189.1 | 6.48 |
| Q13576 | Ras GTPase-activating-like protein IQGAP2 OS=Homo sapiens GN=IQGAP2 PE=1 SV=4 - [IQGA2_HUMAN] | 23 | 21 | 25 | 1575 | 180.5 | 5.64 |
| P07355 | Annexin A2 OS=Homo sapiens GN=ANXA2 PE=1 SV=2 - [ANXA2_HUMAN] | 22 | 39 | 35 | 339 | 38.6 | 7.75 |
| P16435 | NADPH--cytochrome P450 reductase OS=Homo sapiens GN=POR PE=1 SV=2 - [NCPR_HUMAN] | 22 | 22 | 24 | 677 | 76.6 | 5.58 |
| Q14974 | Importin subunit beta-1 OS=Homo sapiens GN=KPNB1 PE=1 SV=2 - [IMB1_HUMAN] | 22 | 33 | 38 | 876 | 97.1 | 4.78 |
| P35221 | Catenin alpha-1 OS=Homo sapiens GN=CTNNA1 PE=1 SV=1 - [CTNA1_HUMAN] | 22 | 22 | 20 | 906 | 100.0 | 6.29 |
| Q00839 | Heterogeneous nuclear ribonucleoprotein U OS=Homo sapiens GN=HNRNPU PE=1 SV=6 - [HNRPU_HUMAN] | 22 | 22 | 23 | 825 | 90.5 | 6.00 |
| O75643 | U5 small nuclear ribonucleoprotein 200 kDa helicase OS=Homo sapiens GN=SNRNP200 PE=1 SV=2 - [U520_HUMAN] | 22 | 18 | 22 | 2136 | 244.4 | 6.06 |
| Q99832 | T-complex protein 1 subunit eta OS=Homo sapiens GN=CCT7 PE=1 SV=2 - [TCPH_HUMAN] | 21 | 23 | 24 | 543 | 59.3 | 7.65 |
| P30101 | Protein disulfide-isomerase A3 OS=Homo sapiens GN=PDIA3 PE=1 SV=4 - [PDIA3_HUMAN] | 21 | 33 | 35 | 505 | 56.7 | 6.35 |
| P54886 | Delta-1-pyrroline-5-carboxylate synthetase OS=Homo sapiens GN=ALDH18A1 PE=1 SV=2 - [P5CS_HUMAN] | 21 | 27 | 25 | 795 | 87.2 | 7.12 |
| P13667 | Protein disulfide-isomerase A4 OS=Homo sapiens GN=PDIA4 PE=1 SV=2 - [PDIA4_HUMAN] | 21 | 20 | 22 | 645 | 72.9 | 5.07 |
| P19338 | Nucleolin OS=Homo sapiens GN=NCL PE=1 SV=3 - [NUCL_HUMAN] | 21 | 29 | 29 | 710 | 76.6 | 4.70 |
| Q9Y4L1 | Hypoxia up-regulated protein 1 OS=Homo sapiens GN=HYOU1 PE=1 SV=1 - [HYOU1_HUMAN] | 21 | 18 | 21 | 999 | 111.3 | 5.22 |
| P12277 | Creatine kinase B-type OS=Homo sapiens GN=CKB PE=1 SV=1 - [KCRB_HUMAN] | 20 | 90 | 92 | 381 | 42.6 | 5.59 |
| P26641 | Elongation factor 1-gamma OS=Homo sapiens GN=EEF1G PE=1 SV=3 - [EF1G_HUMAN] | 20 | 30 | 35 | 437 | 50.1 | 6.67 |
| P09525 | Annexin A4 OS=Homo sapiens GN=ANXA4 PE=1 SV=4 - [ANXA4_HUMAN] | 20 | 26 | 28 | 319 | 35.9 | 6.13 |
| P04844 | Dolichyl-diphosphooligosaccharide--protein glycosyltransferase subunit 2 OS=Homo sapiens GN=RPN2 PE=1 SV=3 - [RPN2_HUMAN] | 20 | 21 | 26 | 631 | 69.2 | 5.69 |
| P36871 | Phosphoglucomutase-1 OS=Homo sapiens GN=PGM1 PE=1 SV=3 - [PGM1_HUMAN] | 20 | 24 | 24 | 562 | 61.4 | 6.76 |
| P07900 | Heat shock protein HSP 90-alpha OS=Homo sapiens GN=HSP90AA1 PE=1 SV=5 - [HS90A_HUMAN] | 20 | 77 | 75 | 732 | 84.6 | 5.02 |
| P23141 | Liver carboxylesterase 1 OS=Homo sapiens GN=CES1 PE=1 SV=2 - [EST1_HUMAN] | 20 | 27 | 23 | 567 | 62.5 | 6.60 |
| P04843 | Dolichyl-diphosphooligosaccharide--protein glycosyltransferase subunit 1 OS=Homo sapiens GN=RPN1 PE=1 SV=1 - [RPN1_HUMAN] | 20 | 24 | 23 | 607 | 68.5 | 6.38 |
| P49748 | Very long-chain specific acyl-CoA dehydrogenase. mitochondrial OS=Homo sapiens GN=ACADVL PE=1 SV=1 - [ACADV_HUMAN] | 20 | 19 | 18 | 655 | 70.3 | 8.75 |
| Q9Y678 | Coatomer subunit gamma OS=Homo sapiens GN=COPG PE=1 SV=1 - [COPG_HUMAN] | 20 | 30 | 30 | 874 | 97.7 | 5.47 |
| P53618 | Coatomer subunit beta OS=Homo sapiens GN=COPB1 PE=1 SV=3 - [COPB_HUMAN] | 20 | 19 | 23 | 953 | 107.1 | 6.05 |
| O14980 | Exportin-1 OS=Homo sapiens GN=XPO1 PE=1 SV=1 - [XPO1_HUMAN] | 20 | 19 | 20 | 1071 | 123.3 | 6.06 |
| P18206 | Vinculin OS=Homo sapiens GN=VCL PE=1 SV=4 - [VINC_HUMAN] | 20 | 24 | 19 | 1134 | 123.7 | 5.66 |
| P60842 | Eukaryotic initiation factor 4A-I OS=Homo sapiens GN=EIF4A1 PE=1 SV=1 - [IF4A1_HUMAN] | 19 | 53 | 42 | 406 | 46.1 | 5.48 |
| P02771 | Alpha-fetoprotein OS=Homo sapiens GN=AFP PE=1 SV=1 - [FETA_HUMAN] | 19 | 21 | 23 | 609 | 68.6 | 5.68 |
| P13674 | Prolyl 4-hydroxylase subunit alpha-1 OS=Homo sapiens GN=P4HA1 PE=1 SV=2 - [P4HA1_HUMAN] | 19 | 19 | 21 | 534 | 61.0 | 6.01 |
| Q06210 | Glucosamine--fructose-6-phosphate aminotransferase [isomerizing] 1 OS=Homo sapiens GN=GFPT1 PE=1 SV=3 - [GFPT1_HUMAN] | 19 | 18 | 14 | 699 | 78.8 | 7.11 |
| P09327 | Villin-1 OS=Homo sapiens GN=VIL1 PE=1 SV=4 - [VILI_HUMAN] | 19 | 19 | 16 | 827 | 92.6 | 6.39 |
| P02786 | Transferrin receptor protein 1 OS=Homo sapiens GN=TFRC PE=1 SV=2 - [TFR1_HUMAN] | 19 | 20 | 17 | 760 | 84.8 | 6.61 |
| Q92900 | Regulator of nonsense transcripts 1 OS=Homo sapiens GN=UPF1 PE=1 SV=2 - [RENT1_HUMAN] | 19 | 10 | 19 | 1129 | 124.3 | 6.61 |
| P07814 | Bifunctional aminoacyl-tRNA synthetase OS=Homo sapiens GN=EPRS PE=1 SV=5 - [SYEP_HUMAN] | 19 | 17 | 16 | 1512 | 170.5 | 7.33 |
| P04075 | Fructose-bisphosphate aldolase A OS=Homo sapiens GN=ALDOA PE=1 SV=2 - [ALDOA_HUMAN] | 18 | 28 | 29 | 364 | 39.4 | 8.09 |
| P08758 | Annexin A5 OS=Homo sapiens GN=ANXA5 PE=1 SV=2 - [ANXA5_HUMAN] | 18 | 28 | 29 | 320 | 35.9 | 5.05 |
| P17987 | T-complex protein 1 subunit alpha OS=Homo sapiens GN=TCP1 PE=1 SV=1 - [TCPA_HUMAN] | 18 | 24 | 23 | 556 | 60.3 | 6.11 |
| P78371 | T-complex protein 1 subunit beta OS=Homo sapiens GN=CCT2 PE=1 SV=4 - [TCPB_HUMAN] | 18 | 22 | 24 | 535 | 57.5 | 6.46 |
| O75083 | WD repeat-containing protein 1 OS=Homo sapiens GN=WDR1 PE=1 SV=4 - [WDR1_HUMAN] | 18 | 15 | 18 | 606 | 66.2 | 6.65 |
| Q9UJS0 | Calcium-binding mitochondrial carrier protein Aralar2 OS=Homo sapiens GN=SLC25A13 PE=1 SV=2 - [CMC2_HUMAN] | 18 | 14 | 19 | 675 | 74.1 | 8.62 |
| P02768 | Serum albumin OS=Homo sapiens GN=ALB PE=1 SV=2 - [ALBU_HUMAN] | 18 | 19 | 24 | 609 | 69.3 | 6.28 |
| Q8WUM4 | Programmed cell death 6-interacting protein OS=Homo sapiens GN=PDCD6IP PE=1 SV=1 - [PDC6I_HUMAN] | 18 | 20 | 17 | 868 | 96.0 | 6.52 |
| P02787 | Serotransferrin OS=Homo sapiens GN=TF PE=1 SV=3 - [TRFE_HUMAN] | 18 | 13 | 16 | 698 | 77.0 | 7.12 |
| Q15393 | Splicing factor 3B subunit 3 OS=Homo sapiens GN=SF3B3 PE=1 SV=4 - [SF3B3_HUMAN] | 18 | 10 | 17 | 1217 | 135.5 | 5.26 |
| Q16531 | DNA damage-binding protein 1 OS=Homo sapiens GN=DDB1 PE=1 SV=1 - [DDB1_HUMAN] | 18 | 16 | 21 | 1140 | 126.9 | 5.26 |
| Q14152 | Eukaryotic translation initiation factor 3 subunit A OS=Homo sapiens GN=EIF3A PE=1 SV=1 - [EIF3A_HUMAN] | 18 | 15 | 20 | 1382 | 166.5 | 6.79 |
| Q6P2Q9 | Pre-mRNA-processing-splicing factor 8 OS=Homo sapiens GN=PRPF8 PE=1 SV=2 - [PRP8_HUMAN] | 18 | 16 | 15 | 2335 | 273.4 | 8.84 |
| P04406 | Glyceraldehyde-3-phosphate dehydrogenase OS=Homo sapiens GN=GAPDH PE=1 SV=3 - [G3P_HUMAN] | 17 | 63 | 63 | 335 | 36.0 | 8.46 |
| P60174 | Triosephosphate isomerase OS=Homo sapiens GN=TPI1 PE=1 SV=3 - [TPIS_HUMAN] | 17 | 34 | 37 | 286 | 30.8 | 5.92 |
| O75874 | Isocitrate dehydrogenase [NADP] cytoplasmic OS=Homo sapiens GN=IDH1 PE=1 SV=2 - [IDHC_HUMAN] | 17 | 27 | 31 | 414 | 46.6 | 7.01 |
| P22234 | Multifunctional protein ADE2 OS=Homo sapiens GN=PAICS PE=1 SV=3 - [PUR6_HUMAN] | 17 | 15 | 22 | 425 | 47.0 | 7.23 |
| P49411 | Elongation factor Tu. mitochondrial OS=Homo sapiens GN=TUFM PE=1 SV=2 - [EFTU_HUMAN] | 17 | 16 | 20 | 452 | 49.5 | 7.61 |
| Q9NZI8 | Insulin-like growth factor 2 mRNA-binding protein 1 OS=Homo sapiens GN=IGF2BP1 PE=1 SV=2 - [IF2B1_HUMAN] | 17 | 20 | 23 | 577 | 63.4 | 9.20 |
| P00367 | Glutamate dehydrogenase 1. mitochondrial OS=Homo sapiens GN=GLUD1 PE=1 SV=2 - [DHE3_HUMAN] | 17 | 15 | 15 | 558 | 61.4 | 7.80 |
| Q96QK1 | Vacuolar protein sorting-associated protein 35 OS=Homo sapiens GN=VPS35 PE=1 SV=2 - [VPS35_HUMAN] | 17 | 18 | 20 | 796 | 91.6 | 5.49 |
| Q9BSJ8 | Extended synaptotagmin-1 OS=Homo sapiens GN=ESYT1 PE=1 SV=1 - [ESYT1_HUMAN] | 17 | 19 | 15 | 1104 | 122.8 | 5.83 |
| P43243 | Matrin-3 OS=Homo sapiens GN=MATR3 PE=1 SV=2 - [MATR3_HUMAN] | 17 | 18 | 20 | 847 | 94.6 | 6.25 |
| O94979 | Protein transport protein Sec31A OS=Homo sapiens GN=SEC31A PE=1 SV=3 - [SC31A_HUMAN] | 17 | 10 | 16 | 1220 | 132.9 | 6.89 |
| P50395 | Rab GDP dissociation inhibitor beta OS=Homo sapiens GN=GDI2 PE=1 SV=2 - [GDIB_HUMAN] | 16 | 28 | 31 | 445 | 50.6 | 6.47 |
| P61978 | Heterogeneous nuclear ribonucleoprotein K OS=Homo sapiens GN=HNRNPK PE=1 SV=1 - [HNRPK_HUMAN] | 16 | 38 | 38 | 463 | 50.9 | 5.54 |
| P22626 | Heterogeneous nuclear ribonucleoproteins A2/B1 OS=Homo sapiens GN=HNRNPA2B1 PE=1 SV=2 - [ROA2_HUMAN] | 16 | 29 | 32 | 353 | 37.4 | 8.95 |
| P48735 | Isocitrate dehydrogenase [NADP]. mitochondrial OS=Homo sapiens GN=IDH2 PE=1 SV=2 - [IDHP_HUMAN] | 16 | 20 | 16 | 452 | 50.9 | 8.69 |
| Q01518 | Adenylyl cyclase-associated protein 1 OS=Homo sapiens GN=CAP1 PE=1 SV=5 - [CAP1_HUMAN] | 16 | 33 | 30 | 475 | 51.9 | 8.06 |
| P14868 | Aspartyl-tRNA synthetase. cytoplasmic OS=Homo sapiens GN=DARS PE=1 SV=2 - [SYDC_HUMAN] | 16 | 11 | 13 | 501 | 57.1 | 6.55 |
| P04040 | Catalase OS=Homo sapiens GN=CAT PE=1 SV=3 - [CATA_HUMAN] | 16 | 13 | 15 | 527 | 59.7 | 7.39 |
| P33176 | Kinesin-1 heavy chain OS=Homo sapiens GN=KIF5B PE=1 SV=1 - [KINH_HUMAN] | 16 | 22 | 21 | 963 | 109.6 | 6.51 |
| P52272 | Heterogeneous nuclear ribonucleoprotein M OS=Homo sapiens GN=HNRNPM PE=1 SV=3 - [HNRPM_HUMAN] | 16 | 11 | 16 | 730 | 77.5 | 8.70 |
| P47897 | Glutaminyl-tRNA synthetase OS=Homo sapiens GN=QARS PE=1 SV=1 - [SYQ_HUMAN] | 16 | 15 | 18 | 775 | 87.7 | 7.15 |
| P08133 | Annexin A6 OS=Homo sapiens GN=ANXA6 PE=1 SV=3 - [ANXA6_HUMAN] | 16 | 12 | 14 | 673 | 75.8 | 5.60 |
| Q92945 | Far upstream element-binding protein 2 OS=Homo sapiens GN=KHSRP PE=1 SV=4 - [FUBP2_HUMAN] | 16 | 13 | 17 | 711 | 73.1 | 7.30 |
| Q14566 | DNA replication licensing factor MCM6 OS=Homo sapiens GN=MCM6 PE=1 SV=1 - [MCM6_HUMAN] | 16 | 14 | 16 | 821 | 92.8 | 5.41 |
| Q00341 | Vigilin OS=Homo sapiens GN=HDLBP PE=1 SV=2 - [VIGLN_HUMAN] | 16 | 10 | 15 | 1268 | 141.4 | 6.87 |
| P00558 | Phosphoglycerate kinase 1 OS=Homo sapiens GN=PGK1 PE=1 SV=3 - [PGK1_HUMAN] | 15 | 21 | 13 | 417 | 44.6 | 8.10 |
| P62424 | 60S ribosomal protein L7a OS=Homo sapiens GN=RPL7A PE=1 SV=2 - [RL7A_HUMAN] | 15 | 16 | 14 | 266 | 30.0 | 10.61 |
| Q01581 | Hydroxymethylglutaryl-CoA synthase. cytoplasmic OS=Homo sapiens GN=HMGCS1 PE=1 SV=2 - [HMCS1_HUMAN] | 15 | 16 | 15 | 520 | 57.3 | 5.41 |
| O43175 | D-3-phosphoglycerate dehydrogenase OS=Homo sapiens GN=PHGDH PE=1 SV=4 - [SERA_HUMAN] | 15 | 12 | 21 | 533 | 56.6 | 6.71 |
| P06744 | Glucose-6-phosphate isomerase OS=Homo sapiens GN=GPI PE=1 SV=4 - [G6PI_HUMAN] | 15 | 18 | 19 | 558 | 63.1 | 8.32 |
| P23526 | Adenosylhomocysteinase OS=Homo sapiens GN=AHCY PE=1 SV=4 - [SAHH_HUMAN] | 15 | 16 | 23 | 432 | 47.7 | 6.34 |
| P49368 | T-complex protein 1 subunit gamma OS=Homo sapiens GN=CCT3 PE=1 SV=4 - [TCPG_HUMAN] | 15 | 12 | 15 | 545 | 60.5 | 6.49 |
| P08107 | Heat shock 70 kDa protein 1A/1B OS=Homo sapiens GN=HSPA1A PE=1 SV=5 - [HSP71_HUMAN] | 15 | 18 | 22 | 641 | 70.0 | 5.66 |
| P28838 | Cytosol aminopeptidase OS=Homo sapiens GN=LAP3 PE=1 SV=3 - [AMPL_HUMAN] | 15 | 13 | 14 | 519 | 56.1 | 7.93 |
| P48643 | T-complex protein 1 subunit epsilon OS=Homo sapiens GN=CCT5 PE=1 SV=1 - [TCPE_HUMAN] | 15 | 12 | 15 | 541 | 59.6 | 5.66 |
| P40227 | T-complex protein 1 subunit zeta OS=Homo sapiens GN=CCT6A PE=1 SV=3 - [TCPZ_HUMAN] | 15 | 27 | 24 | 531 | 58.0 | 6.68 |
| P05023 | Sodium/potassium-transporting ATPase subunit alpha-1 OS=Homo sapiens GN=ATP1A1 PE=1 SV=1 - [AT1A1_HUMAN] | 15 | 23 | 23 | 1023 | 112.8 | 5.49 |
| P55884 | Eukaryotic translation initiation factor 3 subunit B OS=Homo sapiens GN=EIF3B PE=1 SV=3 - [EIF3B_HUMAN] | 15 | 14 | 12 | 814 | 92.4 | 5.00 |
| Q13200 | 26S proteasome non-ATPase regulatory subunit 2 OS=Homo sapiens GN=PSMD2 PE=1 SV=3 - [PSMD2_HUMAN] | 15 | 11 | 17 | 908 | 100.1 | 5.20 |
| O95373 | Importin-7 OS=Homo sapiens GN=IPO7 PE=1 SV=1 - [IPO7_HUMAN] | 15 | 14 | 17 | 1038 | 119.4 | 4.82 |
| P55786 | Puromycin-sensitive aminopeptidase OS=Homo sapiens GN=NPEPPS PE=1 SV=2 - [PSA_HUMAN] | 15 | 8 | 15 | 919 | 103.2 | 5.72 |
| P53396 | ATP-citrate synthase OS=Homo sapiens GN=ACLY PE=1 SV=3 - [ACLY_HUMAN] | 15 | 17 | 18 | 1101 | 120.8 | 7.33 |
| P08183 | Multidrug resistance protein 1 OS=Homo sapiens GN=ABCB1 PE=1 SV=3 - [MDR1_HUMAN] | 15 | 19 | 24 | 1280 | 141.4 | 9.00 |
| P20020 | Plasma membrane calcium-transporting ATPase 1 OS=Homo sapiens OX=9606 GN=ATP2B1 PE=1 SV=4 - [AT2B1_HUMAN] | 15 | 14 | 14 | 1220 | 134.6 | 5.91 |
| P35232 | Prohibitin OS=Homo sapiens GN=PHB PE=1 SV=1 - [PHB_HUMAN] | 14 | 16 | 19 | 272 | 29.8 | 5.76 |
| P63244 | Guanine nucleotide-binding protein subunit beta-2-like 1 OS=Homo sapiens GN=GNB2L1 PE=1 SV=3 - [GBLP_HUMAN] | 14 | 14 | 17 | 317 | 35.1 | 7.69 |
| P30041 | Peroxiredoxin-6 OS=Homo sapiens GN=PRDX6 PE=1 SV=3 - [PRDX6_HUMAN] | 14 | 23 | 23 | 224 | 25.0 | 6.38 |
| P23396 | 40S ribosomal protein S3 OS=Homo sapiens GN=RPS3 PE=1 SV=2 - [RS3_HUMAN] | 14 | 13 | 20 | 243 | 26.7 | 9.66 |
| P27797 | Calreticulin OS=Homo sapiens GN=CALR PE=1 SV=1 - [CALR_HUMAN] | 14 | 22 | 25 | 417 | 48.1 | 4.44 |
| P62258 | 14-3-3 protein epsilon OS=Homo sapiens GN=YWHAE PE=1 SV=1 - [1433E_HUMAN] | 14 | 28 | 22 | 255 | 29.2 | 4.74 |
| P05455 | Lupus La protein OS=Homo sapiens GN=SSB PE=1 SV=2 - [LA_HUMAN] | 14 | 14 | 15 | 408 | 46.8 | 7.12 |
| Q16555 | Dihydropyrimidinase-related protein 2 OS=Homo sapiens GN=DPYSL2 PE=1 SV=1 - [DPYL2_HUMAN] | 14 | 11 | 12 | 572 | 62.3 | 6.38 |
| P29401 | Transketolase OS=Homo sapiens GN=TKT PE=1 SV=3 - [TKT_HUMAN] | 14 | 22 | 21 | 623 | 67.8 | 7.66 |
| Q6DD88 | Atlastin-3 OS=Homo sapiens GN=ATL3 PE=1 SV=1 - [ATLA3_HUMAN] | 14 | 13 | 14 | 541 | 60.5 | 5.66 |
| P37837 | Transaldolase OS=Homo sapiens GN=TALDO1 PE=1 SV=2 - [TALDO_HUMAN] | 14 | 12 | 14 | 337 | 37.5 | 6.81 |
| P52209 | 6-phosphogluconate dehydrogenase. decarboxylating OS=Homo sapiens GN=PGD PE=1 SV=3 - [6PGD_HUMAN] | 14 | 20 | 18 | 483 | 53.1 | 7.23 |
| P36578 | 60S ribosomal protein L4 OS=Homo sapiens GN=RPL4 PE=1 SV=5 - [RL4_HUMAN] | 14 | 11 | 14 | 427 | 47.7 | 11.06 |
| O60488 | Long-chain-fatty-acid--CoA ligase 4 OS=Homo sapiens GN=ACSL4 PE=1 SV=2 - [ACSL4_HUMAN] | 14 | 12 | 17 | 711 | 79.1 | 8.38 |
| Q9Y262 | Eukaryotic translation initiation factor 3 subunit L OS=Homo sapiens GN=EIF3L PE=1 SV=1 - [EIF3L_HUMAN] | 14 | 15 | 16 | 564 | 66.7 | 6.34 |
| P50990 | T-complex protein 1 subunit theta OS=Homo sapiens GN=CCT8 PE=1 SV=4 - [TCPQ_HUMAN] | 14 | 15 | 12 | 548 | 59.6 | 5.60 |
| P40939 | Trifunctional enzyme subunit alpha. mitochondrial OS=Homo sapiens GN=HADHA PE=1 SV=2 - [ECHA_HUMAN] | 14 | 13 | 16 | 763 | 82.9 | 9.04 |
| P33993 | DNA replication licensing factor MCM7 OS=Homo sapiens GN=MCM7 PE=1 SV=4 - [MCM7_HUMAN] | 14 | 13 | 14 | 719 | 81.3 | 6.46 |
| P22059 | Oxysterol-binding protein 1 OS=Homo sapiens GN=OSBP PE=1 SV=1 - [OSBP1_HUMAN] | 14 | 10 | 14 | 807 | 89.4 | 7.30 |
| Q9NYU2 | UDP-glucose:glycoprotein glucosyltransferase 1 OS=Homo sapiens GN=UGGT1 PE=1 SV=3 - [UGGG1_HUMAN] | 14 | 12 | 15 | 1555 | 177.1 | 5.63 |
| Q9BQG0 | Myb-binding protein 1A OS=Homo sapiens GN=MYBBP1A PE=1 SV=2 - [MBB1A_HUMAN] | 14 | 14 | 13 | 1328 | 148.8 | 9.28 |
| Q04637 | Eukaryotic translation initiation factor 4 gamma 1 OS=Homo sapiens GN=EIF4G1 PE=1 SV=4 - [IF4G1_HUMAN] | 14 | 12 | 13 | 1599 | 175.4 | 5.33 |
| P09211 | Glutathione S-transferase P OS=Homo sapiens GN=GSTP1 PE=1 SV=2 - [GSTP1_HUMAN] | 13 | 38 | 41 | 210 | 23.3 | 5.64 |
| P21796 | Voltage-dependent anion-selective channel protein 1 OS=Homo sapiens GN=VDAC1 PE=1 SV=2 - [VDAC1_HUMAN] | 13 | 17 | 21 | 283 | 30.8 | 8.54 |
| P27695 | DNA-(apurinic or apyrimidinic site) lyase OS=Homo sapiens GN=APEX1 PE=1 SV=2 - [APEX1_HUMAN] | 13 | 13 | 15 | 318 | 35.5 | 8.12 |
| P78417 | Glutathione S-transferase omega-1 OS=Homo sapiens GN=GSTO1 PE=1 SV=2 - [GSTO1_HUMAN] | 13 | 15 | 18 | 241 | 27.5 | 6.60 |
| Q12905 | Interleukin enhancer-binding factor 2 OS=Homo sapiens GN=ILF2 PE=1 SV=2 - [ILF2_HUMAN] | 13 | 18 | 15 | 390 | 43.0 | 5.26 |
| P39656 | Dolichyl-diphosphooligosaccharide--protein glycosyltransferase 48 kDa subunit OS=Homo sapiens GN=DDOST PE=1 SV=4 - [OST48_HUMAN] | 13 | 13 | 18 | 456 | 50.8 | 6.55 |
| P40926 | Malate dehydrogenase. mitochondrial OS=Homo sapiens GN=MDH2 PE=1 SV=3 - [MDHM_HUMAN] | 13 | 19 | 22 | 338 | 35.5 | 8.68 |
| P30740 | Leukocyte elastase inhibitor OS=Homo sapiens GN=SERPINB1 PE=1 SV=1 - [ILEU_HUMAN] | 13 | 13 | 11 | 379 | 42.7 | 6.28 |
| Q9UQ80 | Proliferation-associated protein 2G4 OS=Homo sapiens GN=PA2G4 PE=1 SV=3 - [PA2G4_HUMAN] | 13 | 16 | 13 | 394 | 43.8 | 6.55 |
| Q99541 | Perilipin-2 OS=Homo sapiens GN=PLIN2 PE=1 SV=2 - [PLIN2_HUMAN] | 13 | 15 | 18 | 437 | 48.0 | 6.80 |
| P50454 | Serpin H1 OS=Homo sapiens GN=SERPINH1 PE=1 SV=2 - [SERPH_HUMAN] | 13 | 16 | 18 | 418 | 46.4 | 8.69 |
| P04424 | Argininosuccinate lyase OS=Homo sapiens GN=ASL PE=1 SV=4 - [ARLY_HUMAN] | 13 | 11 | 17 | 464 | 51.6 | 6.48 |
| Q02878 | 60S ribosomal protein L6 OS=Homo sapiens GN=RPL6 PE=1 SV=3 - [RL6_HUMAN] | 13 | 18 | 17 | 288 | 32.7 | 10.58 |
| P14866 | Heterogeneous nuclear ribonucleoprotein L OS=Homo sapiens GN=HNRNPL PE=1 SV=2 - [HNRPL_HUMAN] | 13 | 11 | 14 | 589 | 64.1 | 8.22 |
| Q99873 | Protein arginine N-methyltransferase 1 OS=Homo sapiens OX=9606 GN=PRMT1 PE=1 SV=3 - [ANM1_HUMAN] | 13 | 12 | 13 | 371 | 42.4 | 5.35 |
| P50991 | T-complex protein 1 subunit delta OS=Homo sapiens GN=CCT4 PE=1 SV=4 - [TCPD_HUMAN] | 13 | 16 | 19 | 539 | 57.9 | 7.83 |
| P12268 | Inosine-5'-monophosphate dehydrogenase 2 OS=Homo sapiens GN=IMPDH2 PE=1 SV=2 - [IMDH2_HUMAN] | 13 | 14 | 13 | 514 | 55.8 | 6.90 |
| Q16851 | UTP--glucose-1-phosphate uridylyltransferase OS=Homo sapiens GN=UGP2 PE=1 SV=5 - [UGPA_HUMAN] | 13 | 13 | 16 | 508 | 56.9 | 8.15 |
| Q15436 | Protein transport protein Sec23A OS=Homo sapiens GN=SEC23A PE=1 SV=2 - [SC23A_HUMAN] | 13 | 12 | 19 | 765 | 86.1 | 7.08 |
| P07384 | Calpain-1 catalytic subunit OS=Homo sapiens GN=CAPN1 PE=1 SV=1 - [CAN1_HUMAN] | 13 | 10 | 15 | 714 | 81.8 | 5.67 |
| P17844 | Probable ATP-dependent RNA helicase DDX5 OS=Homo sapiens GN=DDX5 PE=1 SV=1 - [DDX5_HUMAN] | 13 | 16 | 16 | 614 | 69.1 | 8.92 |
| Q12906 | Interleukin enhancer-binding factor 3 OS=Homo sapiens GN=ILF3 PE=1 SV=3 - [ILF3_HUMAN] | 13 | 12 | 16 | 894 | 95.3 | 8.76 |
| O60763 | General vesicular transport factor p115 OS=Homo sapiens GN=USO1 PE=1 SV=2 - [USO1_HUMAN] | 13 | 13 | 11 | 962 | 107.8 | 4.91 |
| O60716 | Catenin delta-1 OS=Homo sapiens GN=CTNND1 PE=1 SV=1 - [CTND1_HUMAN] | 13 | 9 | 12 | 968 | 108.1 | 6.23 |
| O00410 | Importin-5 OS=Homo sapiens GN=IPO5 PE=1 SV=4 - [IPO5_HUMAN] | 13 | 11 | 13 | 1097 | 123.5 | 4.94 |
| Q05682 | Caldesmon OS=Homo sapiens GN=CALD1 PE=1 SV=3 - [CALD1_HUMAN] | 13 | 11 | 13 | 793 | 93.2 | 5.66 |
| Q06830 | Peroxiredoxin-1 OS=Homo sapiens GN=PRDX1 PE=1 SV=1 - [PRDX1_HUMAN] | 12 | 16 | 29 | 199 | 22.1 | 8.13 |
| O00299 | Chloride intracellular channel protein 1 OS=Homo sapiens GN=CLIC1 PE=1 SV=4 - [CLIC1_HUMAN] | 12 | 12 | 11 | 241 | 26.9 | 5.17 |
| Q06323 | Proteasome activator complex subunit 1 OS=Homo sapiens GN=PSME1 PE=1 SV=1 - [PSME1_HUMAN] | 12 | 16 | 17 | 249 | 28.7 | 6.02 |
| P08865 | 40S ribosomal protein SA OS=Homo sapiens GN=RPSA PE=1 SV=4 - [RSSA_HUMAN] | 12 | 15 | 15 | 295 | 32.8 | 4.87 |
| P13804 | Electron transfer flavoprotein subunit alpha. mitochondrial OS=Homo sapiens GN=ETFA PE=1 SV=1 - [ETFA_HUMAN] | 12 | 19 | 18 | 333 | 35.1 | 8.38 |
| Q99623 | Prohibitin-2 OS=Homo sapiens GN=PHB2 PE=1 SV=2 - [PHB2_HUMAN] | 12 | 16 | 15 | 299 | 33.3 | 9.83 |
| P63104 | 14-3-3 protein zeta/delta OS=Homo sapiens GN=YWHAZ PE=1 SV=1 - [1433Z_HUMAN] | 12 | 21 | 21 | 245 | 27.7 | 4.79 |
| O60664 | Perilipin-3 OS=Homo sapiens GN=PLIN3 PE=1 SV=3 - [PLIN3_HUMAN] | 12 | 7 | 11 | 434 | 47.0 | 5.44 |
| Q04917 | 14-3-3 protein eta OS=Homo sapiens GN=YWHAH PE=1 SV=4 - [1433F_HUMAN] | 12 | 15 | 14 | 246 | 28.2 | 4.84 |
| P43686 | 26S protease regulatory subunit 6B OS=Homo sapiens GN=PSMC4 PE=1 SV=2 - [PRS6B_HUMAN] | 12 | 9 | 13 | 418 | 47.3 | 5.21 |
| P09651 | Heterogeneous nuclear ribonucleoprotein A1 OS=Homo sapiens GN=HNRNPA1 PE=1 SV=5 - [ROA1_HUMAN] | 12 | 28 | 33 | 372 | 38.7 | 9.13 |
| Q9UNZ2 | NSFL1 cofactor p47 OS=Homo sapiens GN=NSFL1C PE=1 SV=2 - [NSF1C_HUMAN] | 12 | 12 | 13 | 370 | 40.5 | 5.10 |
| P26599 | Polypyrimidine tract-binding protein 1 OS=Homo sapiens GN=PTBP1 PE=1 SV=1 - [PTBP1_HUMAN] | 12 | 26 | 25 | 531 | 57.2 | 9.17 |
| P54578 | Ubiquitin carboxyl-terminal hydrolase 14 OS=Homo sapiens GN=USP14 PE=1 SV=3 - [UBP14_HUMAN] | 12 | 11 | 14 | 494 | 56.0 | 5.30 |
| P34897 | Serine hydroxymethyltransferase. mitochondrial OS=Homo sapiens GN=SHMT2 PE=1 SV=3 - [GLYM_HUMAN] | 12 | 12 | 12 | 504 | 56.0 | 8.53 |
| Q02790 | Peptidyl-prolyl cis-trans isomerase FKBP4 OS=Homo sapiens GN=FKBP4 PE=1 SV=3 - [FKBP4_HUMAN] | 12 | 10 | 13 | 459 | 51.8 | 5.43 |
| Q8WVM8 | Sec1 family domain-containing protein 1 OS=Homo sapiens GN=SCFD1 PE=1 SV=4 - [SCFD1_HUMAN] | 12 | 16 | 12 | 642 | 72.3 | 6.27 |
| Q9Y230 | RuvB-like 2 OS=Homo sapiens GN=RUVBL2 PE=1 SV=3 - [RUVB2_HUMAN] | 12 | 9 | 9 | 463 | 51.1 | 5.64 |
| Q15067 | Peroxisomal acyl-coenzyme A oxidase 1 OS=Homo sapiens GN=ACOX1 PE=1 SV=3 - [ACOX1_HUMAN] | 12 | 7 | 11 | 660 | 74.4 | 8.16 |
| Q99829 | Copine-1 OS=Homo sapiens GN=CPNE1 PE=1 SV=1 - [CPNE1_HUMAN] | 12 | 13 | 14 | 537 | 59.0 | 5.83 |
| Q10567 | AP-1 complex subunit beta-1 OS=Homo sapiens GN=AP1B1 PE=1 SV=2 - [AP1B1_HUMAN] | 12 | 20 | 21 | 949 | 104.6 | 5.06 |
| O00571 | ATP-dependent RNA helicase DDX3X OS=Homo sapiens GN=DDX3X PE=1 SV=3 - [DDX3X_HUMAN] | 12 | 16 | 19 | 662 | 73.2 | 7.18 |
| O00116 | Alkyldihydroxyacetonephosphate synthase. peroxisomal OS=Homo sapiens GN=AGPS PE=1 SV=1 - [ADAS_HUMAN] | 12 | 8 | 6 | 658 | 72.9 | 7.34 |
| P21980 | Protein-glutamine gamma-glutamyltransferase 2 OS=Homo sapiens GN=TGM2 PE=1 SV=2 - [TGM2_HUMAN] | 12 | 10 | 12 | 687 | 77.3 | 5.22 |
| P27824 | Calnexin OS=Homo sapiens GN=CANX PE=1 SV=2 - [CALX_HUMAN] | 12 | 12 | 8 | 592 | 67.5 | 4.60 |
| P23246 | Splicing factor. proline- and glutamine-rich OS=Homo sapiens GN=SFPQ PE=1 SV=2 - [SFPQ_HUMAN] | 12 | 12 | 13 | 707 | 76.1 | 9.44 |
| O43776 | Asparaginyl-tRNA synthetase. cytoplasmic OS=Homo sapiens GN=NARS PE=1 SV=1 - [SYNC_HUMAN] | 12 | 9 | 11 | 548 | 62.9 | 6.25 |
| Q12864 | Cadherin-17 OS=Homo sapiens GN=CDH17 PE=2 SV=3 - [CAD17_HUMAN] | 12 | 9 | 9 | 832 | 92.2 | 5.14 |
| Q15046 | Lysyl-tRNA synthetase OS=Homo sapiens GN=KARS PE=1 SV=3 - [SYK_HUMAN] | 12 | 9 | 10 | 597 | 68.0 | 6.35 |
| P34932 | Heat shock 70 kDa protein 4 OS=Homo sapiens GN=HSPA4 PE=1 SV=4 - [HSP74_HUMAN] | 12 | 9 | 15 | 840 | 94.3 | 5.19 |
| Q99798 | Aconitate hydratase. mitochondrial OS=Homo sapiens GN=ACO2 PE=1 SV=2 - [ACON_HUMAN] | 12 | 9 | 12 | 780 | 85.4 | 7.61 |
| O94874 | E3 UFM1-protein ligase 1 OS=Homo sapiens GN=KIAA0776 PE=1 SV=2 - [UFL1_HUMAN] | 12 | 6 | 11 | 794 | 89.5 | 6.79 |
| P54136 | Arginyl-tRNA synthetase. cytoplasmic OS=Homo sapiens GN=RARS PE=1 SV=2 - [SYRC_HUMAN] | 12 | 13 | 12 | 660 | 75.3 | 6.68 |
| P35606 | Coatomer subunit beta' OS=Homo sapiens GN=COPB2 PE=1 SV=2 - [COPB2_HUMAN] | 12 | 12 | 14 | 906 | 102.4 | 5.27 |
| P33991 | DNA replication licensing factor MCM4 OS=Homo sapiens GN=MCM4 PE=1 SV=5 - [MCM4_HUMAN] | 12 | 8 | 11 | 863 | 96.5 | 6.74 |
| Q9Y4C2 | Protein FAM115A OS=Homo sapiens GN=FAM115A PE=1 SV=3 - [F115A_HUMAN] | 12 | 12 | 10 | 921 | 102.1 | 6.54 |
| P21399 | Cytoplasmic aconitate hydratase OS=Homo sapiens GN=ACO1 PE=1 SV=3 - [ACOC_HUMAN] | 12 | 11 | 16 | 889 | 98.3 | 6.68 |
| P27487 | Dipeptidyl peptidase 4 OS=Homo sapiens GN=DPP4 PE=1 SV=2 - [DPP4_HUMAN] | 12 | 12 | 9 | 766 | 88.2 | 6.04 |
| P25205 | DNA replication licensing factor MCM3 OS=Homo sapiens GN=MCM3 PE=1 SV=3 - [MCM3_HUMAN] | 12 | 8 | 14 | 808 | 90.9 | 5.77 |
| P06737 | Glycogen phosphorylase. liver form OS=Homo sapiens GN=PYGL PE=1 SV=4 - [PYGL_HUMAN] | 12 | 13 | 15 | 847 | 97.1 | 7.17 |
| P09874 | Poly [ADP-ribose] polymerase 1 OS=Homo sapiens GN=PARP1 PE=1 SV=4 - [PARP1_HUMAN] | 12 | 11 | 11 | 1014 | 113.0 | 8.88 |
| P16615 | Sarcoplasmic/endoplasmic reticulum calcium ATPase 2 OS=Homo sapiens GN=ATP2A2 PE=1 SV=1 - [AT2A2_HUMAN] | 12 | 7 | 9 | 1042 | 114.7 | 5.34 |
| P07737 | Profilin-1 OS=Homo sapiens GN=PFN1 PE=1 SV=2 - [PROF1_HUMAN] | 11 | 16 | 19 | 140 | 15.0 | 8.27 |
| P30086 | Phosphatidylethanolamine-binding protein 1 OS=Homo sapiens GN=PEBP1 PE=1 SV=3 - [PEBP1_HUMAN] | 11 | 12 | 12 | 187 | 21.0 | 7.53 |
| P09936 | Ubiquitin carboxyl-terminal hydrolase isozyme L1 OS=Homo sapiens GN=UCHL1 PE=1 SV=2 - [UCHL1_HUMAN] | 11 | 15 | 11 | 223 | 24.8 | 5.48 |
| P18669 | Phosphoglycerate mutase 1 OS=Homo sapiens GN=PGAM1 PE=1 SV=2 - [PGAM1_HUMAN] | 11 | 16 | 17 | 254 | 28.8 | 7.18 |
| P04792 | Heat shock protein beta-1 OS=Homo sapiens GN=HSPB1 PE=1 SV=2 - [HSPB1_HUMAN] | 11 | 12 | 12 | 205 | 22.8 | 6.40 |
| Q9UJZ1 | Stomatin-like protein 2 OS=Homo sapiens GN=STOML2 PE=1 SV=1 - [STML2_HUMAN] | 11 | 13 | 15 | 356 | 38.5 | 7.39 |
| Q08257 | Quinone oxidoreductase OS=Homo sapiens GN=CRYZ PE=1 SV=1 - [QOR_HUMAN] | 11 | 10 | 13 | 329 | 35.2 | 8.44 |
| P63241 | Eukaryotic translation initiation factor 5A-1 OS=Homo sapiens GN=EIF5A PE=1 SV=2 - [IF5A1_HUMAN] | 11 | 17 | 15 | 154 | 16.8 | 5.24 |
| P23284 | Peptidyl-prolyl cis-trans isomerase B OS=Homo sapiens GN=PPIB PE=1 SV=2 - [PPIB_HUMAN] | 11 | 9 | 14 | 216 | 23.7 | 9.41 |
| P12429 | Annexin A3 OS=Homo sapiens GN=ANXA3 PE=1 SV=3 - [ANXA3_HUMAN] | 11 | 13 | 5 | 323 | 36.4 | 5.92 |
| P18124 | 60S ribosomal protein L7 OS=Homo sapiens GN=RPL7 PE=1 SV=1 - [RL7_HUMAN] | 11 | 7 | 12 | 248 | 29.2 | 10.65 |
| P31943 | Heterogeneous nuclear ribonucleoprotein H OS=Homo sapiens GN=HNRNPH1 PE=1 SV=4 - [HNRH1_HUMAN] | 11 | 15 | 17 | 449 | 49.2 | 6.30 |
| P15880 | 40S ribosomal protein S2 OS=Homo sapiens GN=RPS2 PE=1 SV=2 - [RS2_HUMAN] | 11 | 10 | 12 | 293 | 31.3 | 10.24 |
| P38117 | Electron transfer flavoprotein subunit beta OS=Homo sapiens GN=ETFB PE=1 SV=3 - [ETFB_HUMAN] | 11 | 9 | 14 | 255 | 27.8 | 8.10 |
| Q9Y617 | Phosphoserine aminotransferase OS=Homo sapiens GN=PSAT1 PE=1 SV=2 - [SERC_HUMAN] | 11 | 11 | 12 | 370 | 40.4 | 7.66 |
| Q15084 | Protein disulfide-isomerase A6 OS=Homo sapiens GN=PDIA6 PE=1 SV=1 - [PDIA6_HUMAN] | 11 | 19 | 20 | 440 | 48.1 | 5.08 |
| P51991 | Heterogeneous nuclear ribonucleoprotein A3 OS=Homo sapiens GN=HNRNPA3 PE=1 SV=2 - [ROA3_HUMAN] | 11 | 14 | 17 | 378 | 39.6 | 9.01 |
| P11940 | Polyadenylate-binding protein 1 OS=Homo sapiens GN=PABPC1 PE=1 SV=2 - [PABP1_HUMAN] | 11 | 16 | 23 | 636 | 70.6 | 9.50 |
| P13797 | Plastin-3 OS=Homo sapiens GN=PLS3 PE=1 SV=4 - [PLST_HUMAN] | 11 | 15 | 15 | 630 | 70.8 | 5.60 |
| O75390 | Citrate synthase. mitochondrial OS=Homo sapiens GN=CS PE=1 SV=2 - [CISY_HUMAN] | 11 | 15 | 11 | 466 | 51.7 | 8.32 |
| Q96KP4 | Cytosolic non-specific dipeptidase OS=Homo sapiens GN=CNDP2 PE=1 SV=2 - [CNDP2_HUMAN] | 11 | 7 | 11 | 475 | 52.8 | 5.97 |
| P09972 | Fructose-bisphosphate aldolase C OS=Homo sapiens GN=ALDOC PE=1 SV=2 - [ALDOC_HUMAN] | 11 | 13 | 12 | 364 | 39.4 | 6.87 |
| Q9UMS4 | Pre-mRNA-processing factor 19 OS=Homo sapiens GN=PRPF19 PE=1 SV=1 - [PRP19_HUMAN] | 11 | 7 | 13 | 504 | 55.1 | 6.61 |
| O60506 | Heterogeneous nuclear ribonucleoprotein Q OS=Homo sapiens GN=SYNCRIP PE=1 SV=2 - [HNRPQ_HUMAN] | 11 | 13 | 15 | 623 | 69.6 | 8.59 |
| P20700 | Lamin-B1 OS=Homo sapiens GN=LMNB1 PE=1 SV=2 - [LMNB1_HUMAN] | 11 | 15 | 14 | 586 | 66.4 | 5.16 |
| O00231 | 26S proteasome non-ATPase regulatory subunit 11 OS=Homo sapiens GN=PSMD11 PE=1 SV=3 - [PSD11_HUMAN] | 11 | 9 | 9 | 422 | 47.4 | 6.48 |
| O95394 | Phosphoacetylglucosamine mutase OS=Homo sapiens GN=PGM3 PE=1 SV=1 - [AGM1_HUMAN] | 11 | 14 | 10 | 542 | 59.8 | 6.25 |
| P35611 | Alpha-adducin OS=Homo sapiens GN=ADD1 PE=1 SV=2 - [ADDA_HUMAN] | 11 | 7 | 10 | 737 | 80.9 | 5.83 |
| O43390 | Heterogeneous nuclear ribonucleoprotein R OS=Homo sapiens GN=HNRNPR PE=1 SV=1 - [HNRPR_HUMAN] | 11 | 8 | 14 | 633 | 70.9 | 8.13 |
| Q9H4A4 | Aminopeptidase B OS=Homo sapiens GN=RNPEP PE=1 SV=2 - [AMPB_HUMAN] | 11 | 10 | 8 | 650 | 72.5 | 5.74 |
| Q16891 | Mitochondrial inner membrane protein OS=Homo sapiens GN=IMMT PE=1 SV=1 - [IMMT_HUMAN] | 11 | 7 | 11 | 758 | 83.6 | 6.48 |
| P56192 | Methionyl-tRNA synthetase. cytoplasmic OS=Homo sapiens GN=MARS PE=1 SV=2 - [SYMC_HUMAN] | 11 | 6 | 11 | 900 | 101.1 | 6.16 |
| P31948 | Stress-induced-phosphoprotein 1 OS=Homo sapiens GN=STIP1 PE=1 SV=1 - [STIP1_HUMAN] | 11 | 10 | 11 | 543 | 62.6 | 6.80 |
| Q14247 | Src substrate cortactin OS=Homo sapiens GN=CTTN PE=1 SV=2 - [SRC8_HUMAN] | 11 | 6 | 10 | 550 | 61.5 | 5.40 |
| P31040 | Succinate dehydrogenase [ubiquinone] flavoprotein subunit. mitochondrial OS=Homo sapiens GN=SDHA PE=1 SV=2 - [DHSA_HUMAN] | 11 | 9 | 11 | 664 | 72.6 | 7.39 |
| O00469 | Procollagen-lysine.2-oxoglutarate 5-dioxygenase 2 OS=Homo sapiens GN=PLOD2 PE=1 SV=2 - [PLOD2_HUMAN] | 11 | 10 | 7 | 737 | 84.6 | 6.71 |
| Q13263 | Transcription intermediary factor 1-beta OS=Homo sapiens GN=TRIM28 PE=1 SV=5 - [TIF1B_HUMAN] | 11 | 8 | 10 | 835 | 88.5 | 5.77 |
| P78347 | General transcription factor II-I OS=Homo sapiens GN=GTF2I PE=1 SV=2 - [GTF2I_HUMAN] | 11 | 9 | 10 | 998 | 112.3 | 6.39 |
| Q8TEX9 | Importin-4 OS=Homo sapiens GN=IPO4 PE=1 SV=2 - [IPO4_HUMAN] | 11 | 11 | 14 | 1081 | 118.6 | 4.96 |
| P36776 | Lon protease homolog. mitochondrial OS=Homo sapiens GN=LONP1 PE=1 SV=2 - [LONM_HUMAN] | 11 | 9 | 8 | 959 | 106.4 | 6.39 |
| Q9NSE4 | Isoleucyl-tRNA synthetase. mitochondrial OS=Homo sapiens GN=IARS2 PE=1 SV=2 - [SYIM_HUMAN] | 11 | 9 | 10 | 1012 | 113.7 | 7.20 |
| Q99613 | Eukaryotic translation initiation factor 3 subunit C OS=Homo sapiens GN=EIF3C PE=1 SV=1 - [EIF3C_HUMAN] | 11 | 13 | 10 | 913 | 105.3 | 5.68 |
| P27816 | Microtubule-associated protein 4 OS=Homo sapiens GN=MAP4 PE=1 SV=3 - [MAP4_HUMAN] | 11 | 10 | 14 | 1152 | 120.9 | 5.43 |
| P35580 | Myosin-10 OS=Homo sapiens GN=MYH10 PE=1 SV=3 - [MYH10_HUMAN] | 11 | 14 | 3 | 1976 | 228.9 | 5.54 |
| P48681 | Nestin OS=Homo sapiens GN=NES PE=1 SV=2 - [NEST_HUMAN] | 11 | 9 | 7 | 1621 | 177.3 | 4.36 |
| Q99497 | Protein DJ-1 OS=Homo sapiens GN=PARK7 PE=1 SV=2 - [PARK7_HUMAN] | 10 | 14 | 15 | 189 | 19.9 | 6.79 |
| P04179 | Superoxide dismutase [Mn]. mitochondrial OS=Homo sapiens OX=9606 GN=SOD2 PE=1 SV=3 - [SODM_HUMAN] | 10 | 15 | 16 | 222 | 24.7 | 8.25 |
| P62081 | 40S ribosomal protein S7 OS=Homo sapiens GN=RPS7 PE=1 SV=1 - [RS7_HUMAN] | 10 | 15 | 17 | 194 | 22.1 | 10.10 |
| P29692 | Elongation factor 1-delta OS=Homo sapiens GN=EEF1D PE=1 SV=5 - [EF1D_HUMAN] | 10 | 8 | 10 | 281 | 31.1 | 5.01 |
| O14818 | Proteasome subunit alpha type-7 OS=Homo sapiens GN=PSMA7 PE=1 SV=1 - [PSA7_HUMAN] | 10 | 12 | 11 | 248 | 27.9 | 8.46 |
| P61019 | Ras-related protein Rab-2A OS=Homo sapiens GN=RAB2A PE=1 SV=1 - [RAB2A_HUMAN] | 10 | 10 | 8 | 212 | 23.5 | 6.54 |
| P05388 | 60S acidic ribosomal protein P0 OS=Homo sapiens GN=RPLP0 PE=1 SV=1 - [RLA0_HUMAN] | 10 | 9 | 14 | 317 | 34.3 | 5.97 |
| Q15417 | Calponin-3 OS=Homo sapiens GN=CNN3 PE=1 SV=1 - [CNN3_HUMAN] | 10 | 11 | 11 | 329 | 36.4 | 6.05 |
| P22760 | Arylacetamide deacetylase OS=Homo sapiens GN=AADAC PE=1 SV=5 - [AAAD_HUMAN] | 10 | 12 | 9 | 399 | 45.7 | 8.69 |
| P62826 | GTP-binding nuclear protein Ran OS=Homo sapiens GN=RAN PE=1 SV=3 - [RAN_HUMAN] | 10 | 15 | 15 | 216 | 24.4 | 7.49 |
| P40925 | Malate dehydrogenase. cytoplasmic OS=Homo sapiens GN=MDH1 PE=1 SV=4 - [MDHC_HUMAN] | 10 | 13 | 15 | 334 | 36.4 | 7.36 |
| P62701 | 40S ribosomal protein S4. X isoform OS=Homo sapiens GN=RPS4X PE=1 SV=2 - [RS4X_HUMAN] | 10 | 11 | 15 | 263 | 29.6 | 10.15 |
| P17174 | Aspartate aminotransferase. cytoplasmic OS=Homo sapiens GN=GOT1 PE=1 SV=3 - [AATC_HUMAN] | 10 | 9 | 11 | 413 | 46.2 | 7.01 |
| P00390 | Glutathione reductase. mitochondrial OS=Homo sapiens GN=GSR PE=1 SV=2 - [GSHR_HUMAN] | 10 | 16 | 14 | 522 | 56.2 | 8.50 |
| Q9BR76 | Coronin-1B OS=Homo sapiens GN=CORO1B PE=1 SV=1 - [COR1B_HUMAN] | 10 | 10 | 11 | 489 | 54.2 | 5.88 |
| P12814 | Alpha-actinin-1 OS=Homo sapiens GN=ACTN1 PE=1 SV=2 - [ACTN1_HUMAN] | 10 | 35 | 34 | 892 | 103.0 | 5.41 |
| P02649 | Apolipoprotein E OS=Homo sapiens GN=APOE PE=1 SV=1 - [APOE_HUMAN] | 10 | 11 | 9 | 317 | 36.1 | 5.73 |
| P43490 | Nicotinamide phosphoribosyltransferase OS=Homo sapiens GN=NAMPT PE=1 SV=1 - [NAMPT_HUMAN] | 10 | 7 | 7 | 491 | 55.5 | 7.15 |
| P30153 | Serine/threonine-protein phosphatase 2A 65 kDa regulatory subunit A alpha isoform OS=Homo sapiens GN=PPP2R1A PE=1 SV=4 - [2AAA_HUMAN] | 10 | 22 | 20 | 589 | 65.3 | 5.11 |
| P41091 | Eukaryotic translation initiation factor 2 subunit 3 OS=Homo sapiens GN=EIF2S3 PE=1 SV=3 - [IF2G_HUMAN] | 10 | 9 | 8 | 472 | 51.1 | 8.40 |
| P52292 | Importin subunit alpha-2 OS=Homo sapiens GN=KPNA2 PE=1 SV=1 - [IMA2_HUMAN] | 10 | 11 | 15 | 529 | 57.8 | 5.40 |
| P07910 | Heterogeneous nuclear ribonucleoproteins C1/C2 OS=Homo sapiens GN=HNRNPC PE=1 SV=4 - [HNRPC_HUMAN] | 10 | 13 | 16 | 306 | 33.6 | 5.08 |
| P46781 | 40S ribosomal protein S9 OS=Homo sapiens GN=RPS9 PE=1 SV=3 - [RS9_HUMAN] | 10 | 6 | 9 | 194 | 22.6 | 10.65 |
| Q16401 | 26S proteasome non-ATPase regulatory subunit 5 OS=Homo sapiens GN=PSMD5 PE=1 SV=3 - [PSMD5_HUMAN] | 10 | 10 | 9 | 504 | 56.2 | 5.48 |
| P12955 | Xaa-Pro dipeptidase OS=Homo sapiens GN=PEPD PE=1 SV=3 - [PEPD_HUMAN] | 10 | 10 | 11 | 493 | 54.5 | 6.00 |
| P15311 | Ezrin OS=Homo sapiens GN=EZR PE=1 SV=4 - [EZRI_HUMAN] | 10 | 17 | 15 | 586 | 69.4 | 6.27 |
| P60228 | Eukaryotic translation initiation factor 3 subunit E OS=Homo sapiens GN=EIF3E PE=1 SV=1 - [EIF3E_HUMAN] | 10 | 9 | 12 | 445 | 52.2 | 6.04 |
| O95831 | Apoptosis-inducing factor 1. mitochondrial OS=Homo sapiens GN=AIFM1 PE=1 SV=1 - [AIFM1_HUMAN] | 10 | 12 | 9 | 613 | 66.9 | 8.95 |
| P35222 | Catenin beta-1 OS=Homo sapiens GN=CTNNB1 PE=1 SV=1 - [CTNB1_HUMAN] | 10 | 16 | 13 | 781 | 85.4 | 5.86 |
| P48637 | Glutathione synthetase OS=Homo sapiens GN=GSS PE=1 SV=1 - [GSHB_HUMAN] | 10 | 5 | 9 | 474 | 52.4 | 5.92 |
| Q9P258 | Protein RCC2 OS=Homo sapiens GN=RCC2 PE=1 SV=2 - [RCC2_HUMAN] | 10 | 5 | 7 | 522 | 56.0 | 8.78 |
| Q16822 | Phosphoenolpyruvate carboxykinase [GTP]. mitochondrial OS=Homo sapiens OX=9606 GN=PCK2 PE=1 SV=4 - [PCKGM_HUMAN] | 10 | 8 | 9 | 640 | 70.7 | 7.62 |
| P48444 | Coatomer subunit delta OS=Homo sapiens GN=ARCN1 PE=1 SV=1 - [COPD_HUMAN] | 10 | 7 | 10 | 511 | 57.2 | 6.21 |
| P41250 | Glycine--tRNA ligase OS=Homo sapiens GN=GARS PE=1 SV=3 - [SYG_HUMAN] | 10 | 6 | 9 | 739 | 83.1 | 7.03 |
| Q8N163 | Protein KIAA1967 OS=Homo sapiens GN=KIAA1967 PE=1 SV=2 - [K1967_HUMAN] | 10 | 10 | 8 | 923 | 102.8 | 5.22 |
| O43747 | AP-1 complex subunit gamma-1 OS=Homo sapiens GN=AP1G1 PE=1 SV=5 - [AP1G1_HUMAN] | 10 | 10 | 9 | 822 | 91.3 | 6.80 |
| P26640 | Valyl-tRNA synthetase OS=Homo sapiens GN=VARS PE=1 SV=4 - [SYVC_HUMAN] | 10 | 10 | 12 | 1264 | 140.4 | 7.59 |
| O43491 | Band 4.1-like protein 2 OS=Homo sapiens GN=EPB41L2 PE=1 SV=1 - [E41L2_HUMAN] | 10 | 5 | 8 | 1005 | 112.5 | 5.44 |
| P49736 | DNA replication licensing factor MCM2 OS=Homo sapiens GN=MCM2 PE=1 SV=4 - [MCM2_HUMAN] | 10 | 9 | 7 | 904 | 101.8 | 5.52 |
| P41252 | Isoleucyl-tRNA synthetase. cytoplasmic OS=Homo sapiens GN=IARS PE=1 SV=2 - [SYIC_HUMAN] | 10 | 5 | 8 | 1262 | 144.4 | 6.15 |
| P55196 | Afadin OS=Homo sapiens GN=MLLT4 PE=1 SV=3 - [AFAD_HUMAN] | 10 | 6 | 10 | 1824 | 206.7 | 6.47 |
| P68104 | Elongation factor 1-alpha 1 OS=Homo sapiens GN=EEF1A1 PE=1 SV=1 - [EF1A1_HUMAN] | 9 | 60 | 61 | 462 | 50.1 | 9.01 |
| Q99714 | 3-hydroxyacyl-CoA dehydrogenase type-2 OS=Homo sapiens GN=HSD17B10 PE=1 SV=3 - [HCD2_HUMAN] | 9 | 8 | 12 | 261 | 26.9 | 7.78 |
| P37802 | Transgelin-2 OS=Homo sapiens GN=TAGLN2 PE=1 SV=3 - [TAGL2_HUMAN] | 9 | 13 | 15 | 199 | 22.4 | 8.25 |
| Q15165 | Serum paraoxonase/arylesterase 2 OS=Homo sapiens OX=9606 GN=PON2 PE=1 SV=4 - [PON2_HUMAN] | 9 | 10 | 9 | 354 | 39.4 | 5.60 |
| P62937 | Peptidyl-prolyl cis-trans isomerase A OS=Homo sapiens GN=PPIA PE=1 SV=2 - [PPIA_HUMAN] | 9 | 17 | 22 | 165 | 18.0 | 7.81 |
| Q9UL46 | Proteasome activator complex subunit 2 OS=Homo sapiens GN=PSME2 PE=1 SV=4 - [PSME2_HUMAN] | 9 | 14 | 17 | 239 | 27.4 | 5.73 |
| P51858 | Hepatoma-derived growth factor OS=Homo sapiens GN=HDGF PE=1 SV=1 - [HDGF_HUMAN] | 9 | 7 | 8 | 240 | 26.8 | 4.73 |
| P31946 | 14-3-3 protein beta/alpha OS=Homo sapiens GN=YWHAB PE=1 SV=3 - [1433B_HUMAN] | 9 | 20 | 18 | 246 | 28.1 | 4.83 |
| P32119 | Peroxiredoxin-2 OS=Homo sapiens GN=PRDX2 PE=1 SV=5 - [PRDX2_HUMAN] | 9 | 15 | 15 | 198 | 21.9 | 5.97 |
| P27348 | 14-3-3 protein theta OS=Homo sapiens GN=YWHAQ PE=1 SV=1 - [1433T_HUMAN] | 9 | 14 | 15 | 245 | 27.7 | 4.78 |
| P30048 | Thioredoxin-dependent peroxide reductase. mitochondrial OS=Homo sapiens GN=PRDX3 PE=1 SV=3 - [PRDX3_HUMAN] | 9 | 10 | 10 | 256 | 27.7 | 7.78 |
| Q9BPW8 | Protein NipSnap homolog 1 OS=Homo sapiens GN=NIPSNAP1 PE=1 SV=1 - [NIPS1_HUMAN] | 9 | 11 | 13 | 284 | 33.3 | 9.31 |
| Q96FW1 | Ubiquitin thioesterase OTUB1 OS=Homo sapiens GN=OTUB1 PE=1 SV=2 - [OTUB1_HUMAN] | 9 | 9 | 7 | 271 | 31.3 | 4.94 |
| Q14914 | Prostaglandin reductase 1 OS=Homo sapiens GN=PTGR1 PE=1 SV=2 - [PTGR1_HUMAN] | 9 | 9 | 15 | 329 | 35.8 | 8.29 |
| P25786 | Proteasome subunit alpha type-1 OS=Homo sapiens GN=PSMA1 PE=1 SV=1 - [PSA1_HUMAN] | 9 | 9 | 10 | 263 | 29.5 | 6.61 |
| O76003 | Glutaredoxin-3 OS=Homo sapiens GN=GLRX3 PE=1 SV=2 - [GLRX3_HUMAN] | 9 | 9 | 12 | 335 | 37.4 | 5.39 |
| Q96AG4 | Leucine-rich repeat-containing protein 59 OS=Homo sapiens GN=LRRC59 PE=1 SV=1 - [LRC59_HUMAN] | 9 | 10 | 11 | 307 | 34.9 | 9.57 |
| P09661 | U2 small nuclear ribonucleoprotein A' OS=Homo sapiens GN=SNRPA1 PE=1 SV=2 - [RU2A_HUMAN] | 9 | 7 | 8 | 255 | 28.4 | 8.62 |
| P30085 | UMP-CMP kinase OS=Homo sapiens GN=CMPK1 PE=1 SV=3 - [KCY_HUMAN] | 9 | 11 | 12 | 196 | 22.2 | 5.57 |
| P30044 | Peroxiredoxin-5. mitochondrial OS=Homo sapiens GN=PRDX5 PE=1 SV=4 - [PRDX5_HUMAN] | 9 | 9 | 9 | 214 | 22.1 | 8.70 |
| P61247 | 40S ribosomal protein S3a OS=Homo sapiens GN=RPS3A PE=1 SV=2 - [RS3A_HUMAN] | 9 | 9 | 11 | 264 | 29.9 | 9.73 |
| Q9UNM6 | 26S proteasome non-ATPase regulatory subunit 13 OS=Homo sapiens GN=PSMD13 PE=1 SV=2 - [PSD13_HUMAN] | 9 | 11 | 10 | 376 | 42.9 | 5.81 |
| P28074 | Proteasome subunit beta type-5 OS=Homo sapiens GN=PSMB5 PE=1 SV=3 - [PSB5_HUMAN] | 9 | 10 | 10 | 263 | 28.5 | 6.92 |
| P13489 | Ribonuclease inhibitor OS=Homo sapiens GN=RNH1 PE=1 SV=2 - [RINI_HUMAN] | 9 | 10 | 7 | 461 | 49.9 | 4.82 |
| P68400 | Casein kinase II subunit alpha OS=Homo sapiens GN=CSNK2A1 PE=1 SV=1 - [CSK21_HUMAN] | 9 | 8 | 7 | 391 | 45.1 | 7.74 |
| Q9H0B6 | Kinesin light chain 2 OS=Homo sapiens GN=KLC2 PE=1 SV=1 - [KLC2_HUMAN] | 9 | 9 | 14 | 622 | 68.9 | 7.15 |
| P62495 | Eukaryotic peptide chain release factor subunit 1 OS=Homo sapiens GN=ETF1 PE=1 SV=3 - [ERF1_HUMAN] | 9 | 8 | 10 | 437 | 49.0 | 5.71 |
| P35241 | Radixin OS=Homo sapiens GN=RDX PE=1 SV=1 - [RADI_HUMAN] | 9 | 17 | 17 | 583 | 68.5 | 6.37 |
| P35998 | 26S protease regulatory subunit 7 OS=Homo sapiens GN=PSMC2 PE=1 SV=3 - [PRS7_HUMAN] | 9 | 7 | 7 | 433 | 48.6 | 5.95 |
| P42765 | 3-ketoacyl-CoA thiolase. mitochondrial OS=Homo sapiens GN=ACAA2 PE=1 SV=2 - [THIM_HUMAN] | 9 | 4 | 9 | 397 | 41.9 | 8.09 |
| O15460 | Prolyl 4-hydroxylase subunit alpha-2 OS=Homo sapiens GN=P4HA2 PE=1 SV=1 - [P4HA2_HUMAN] | 9 | 8 | 9 | 535 | 60.9 | 5.71 |
| P61221 | ATP-binding cassette sub-family E member 1 OS=Homo sapiens GN=ABCE1 PE=1 SV=1 - [ABCE1_HUMAN] | 9 | 6 | 8 | 599 | 67.3 | 8.34 |
| P09622 | Dihydrolipoyl dehydrogenase. mitochondrial OS=Homo sapiens GN=DLD PE=1 SV=2 - [DLDH_HUMAN] | 9 | 6 | 12 | 509 | 54.1 | 7.85 |
| P54577 | Tyrosyl-tRNA synthetase. cytoplasmic OS=Homo sapiens GN=YARS PE=1 SV=4 - [SYYC_HUMAN] | 9 | 4 | 9 | 528 | 59.1 | 7.05 |
| Q6P996 | Pyridoxal-dependent decarboxylase domain-containing protein 1 OS=Homo sapiens GN=PDXDC1 PE=1 SV=2 - [PDXD1_HUMAN] | 9 | 6 | 7 | 788 | 86.7 | 5.38 |
| P17858 | 6-phosphofructokinase. liver type OS=Homo sapiens GN=PFKL PE=1 SV=6 - [K6PL_HUMAN] | 9 | 7 | 8 | 780 | 85.0 | 7.50 |
| Q9UHD8 | Septin-9 OS=Homo sapiens GN=SEPT9 PE=1 SV=2 - [SEPT9_HUMAN] | 9 | 6 | 7 | 586 | 65.4 | 8.97 |
| Q92973 | Transportin-1 OS=Homo sapiens GN=TNPO1 PE=1 SV=2 - [TNPO1_HUMAN] | 9 | 10 | 10 | 898 | 102.3 | 4.98 |
| Q03252 | Lamin-B2 OS=Homo sapiens GN=LMNB2 PE=1 SV=4 - [LMNB2_HUMAN] | 9 | 11 | 11 | 620 | 69.9 | 5.59 |
| Q8TCJ2 | Dolichyl-diphosphooligosaccharide--protein glycosyltransferase subunit STT3B OS=Homo sapiens GN=STT3B PE=1 SV=1 - [STT3B_HUMAN] | 9 | 6 | 9 | 826 | 93.6 | 8.91 |
| P14314 | Glucosidase 2 subunit beta OS=Homo sapiens GN=PRKCSH PE=1 SV=2 - [GLU2B_HUMAN] | 9 | 9 | 12 | 528 | 59.4 | 4.41 |
| Q92499 | ATP-dependent RNA helicase DDX1 OS=Homo sapiens GN=DDX1 PE=1 SV=2 - [DDX1_HUMAN] | 9 | 9 | 12 | 740 | 82.4 | 7.23 |
| P15144 | Aminopeptidase N OS=Homo sapiens GN=ANPEP PE=1 SV=4 - [AMPN_HUMAN] | 9 | 7 | 8 | 967 | 109.5 | 5.48 |
| P45974 | Ubiquitin carboxyl-terminal hydrolase 5 OS=Homo sapiens GN=USP5 PE=1 SV=2 - [UBP5_HUMAN] | 9 | 6 | 9 | 858 | 95.7 | 5.03 |
| O43143 | Putative pre-mRNA-splicing factor ATP-dependent RNA helicase DHX15 OS=Homo sapiens GN=DHX15 PE=1 SV=2 - [DHX15_HUMAN] | 9 | 12 | 11 | 795 | 90.9 | 7.46 |
| O15067 | Phosphoribosylformylglycinamidine synthase OS=Homo sapiens GN=PFAS PE=1 SV=4 - [PUR4_HUMAN] | 9 | 6 | 8 | 1338 | 144.6 | 5.76 |
| Q9HCE1 | Putative helicase MOV-10 OS=Homo sapiens GN=MOV10 PE=1 SV=2 - [MOV10_HUMAN] | 9 | 4 | 9 | 1003 | 113.6 | 8.82 |
| Q92616 | eIF-2-alpha kinase activator GCN1 OS=Homo sapiens GN=GCN1 PE=1 SV=6 - [GCN1_HUMAN] | 9 | 10 | 9 | 2671 | 292.6 | 7.47 |
| P23528 | Cofilin-1 OS=Homo sapiens GN=CFL1 PE=1 SV=3 - [COF1_HUMAN] | 8 | 24 | 25 | 166 | 18.5 | 8.09 |
| P07148 | Fatty acid-binding protein. liver OS=Homo sapiens GN=FABP1 PE=1 SV=1 - [FABPL_HUMAN] | 8 | 15 | 19 | 127 | 14.2 | 7.18 |
| O95865 | N(G).N(G)-dimethylarginine dimethylaminohydrolase 2 OS=Homo sapiens GN=DDAH2 PE=1 SV=1 - [DDAH2_HUMAN] | 8 | 8 | 5 | 285 | 29.6 | 6.01 |
| P46782 | 40S ribosomal protein S5 OS=Homo sapiens GN=RPS5 PE=1 SV=4 - [RS5_HUMAN] | 8 | 13 | 10 | 204 | 22.9 | 9.72 |
| Q15365 | Poly(rC)-binding protein 1 OS=Homo sapiens GN=PCBP1 PE=1 SV=2 - [PCBP1_HUMAN] | 8 | 11 | 11 | 356 | 37.5 | 7.09 |
| P51149 | Ras-related protein Rab-7a OS=Homo sapiens GN=RAB7A PE=1 SV=1 - [RAB7A_HUMAN] | 8 | 7 | 8 | 207 | 23.5 | 6.70 |
| P48047 | ATP synthase subunit O. mitochondrial OS=Homo sapiens GN=ATP5O PE=1 SV=1 - [ATPO_HUMAN] | 8 | 9 | 8 | 213 | 23.3 | 9.96 |
| Q15691 | Microtubule-associated protein RP/EB family member 1 OS=Homo sapiens GN=MAPRE1 PE=1 SV=3 - [MARE1_HUMAN] | 8 | 7 | 7 | 268 | 30.0 | 5.14 |
| P54819 | Adenylate kinase 2. mitochondrial OS=Homo sapiens GN=AK2 PE=1 SV=2 - [KAD2_HUMAN] | 8 | 5 | 10 | 239 | 26.5 | 7.81 |
| Q07020 | 60S ribosomal protein L18 OS=Homo sapiens GN=RPL18 PE=1 SV=2 - [RL18_HUMAN] | 8 | 7 | 8 | 188 | 21.6 | 11.72 |
| P62249 | 40S ribosomal protein S16 OS=Homo sapiens GN=RPS16 PE=1 SV=2 - [RS16_HUMAN] | 8 | 7 | 10 | 146 | 16.4 | 10.21 |
| Q9UBQ7 | Glyoxylate reductase/hydroxypyruvate reductase OS=Homo sapiens GN=GRHPR PE=1 SV=1 - [GRHPR_HUMAN] | 8 | 8 | 9 | 328 | 35.6 | 7.39 |
| P20618 | Proteasome subunit beta type-1 OS=Homo sapiens GN=PSMB1 PE=1 SV=2 - [PSB1_HUMAN] | 8 | 9 | 12 | 241 | 26.5 | 8.13 |
| P62269 | 40S ribosomal protein S18 OS=Homo sapiens GN=RPS18 PE=1 SV=3 - [RS18_HUMAN] | 8 | 10 | 11 | 152 | 17.7 | 10.99 |
| Q9H9B4 | Sideroflexin-1 OS=Homo sapiens GN=SFXN1 PE=1 SV=4 - [SFXN1_HUMAN] | 8 | 8 | 7 | 322 | 35.6 | 9.07 |
| P24539 | ATP synthase subunit b. mitochondrial OS=Homo sapiens GN=ATP5F1 PE=1 SV=2 - [AT5F1_HUMAN] | 8 | 11 | 9 | 256 | 28.9 | 9.36 |
| Q9Y6C9 | Mitochondrial carrier homolog 2 OS=Homo sapiens GN=MTCH2 PE=1 SV=1 - [MTCH2_HUMAN] | 8 | 8 | 11 | 303 | 33.3 | 7.97 |
| P25789 | Proteasome subunit alpha type-4 OS=Homo sapiens GN=PSMA4 PE=1 SV=1 - [PSA4_HUMAN] | 8 | 9 | 6 | 261 | 29.5 | 7.72 |
| P22695 | Cytochrome b-c1 complex subunit 2. mitochondrial OS=Homo sapiens GN=UQCRC2 PE=1 SV=3 - [QCR2_HUMAN] | 8 | 8 | 5 | 453 | 48.4 | 8.63 |
| Q9Y320 | Thioredoxin-related transmembrane protein 2 OS=Homo sapiens GN=TMX2 PE=1 SV=1 - [TMX2_HUMAN] | 8 | 9 | 9 | 296 | 34.0 | 8.69 |
| Q12792 | Twinfilin-1 OS=Homo sapiens GN=TWF1 PE=1 SV=3 - [TWF1_HUMAN] | 8 | 10 | 12 | 350 | 40.3 | 6.96 |
| P47756 | F-actin-capping protein subunit beta OS=Homo sapiens GN=CAPZB PE=1 SV=4 - [CAPZB_HUMAN] | 8 | 11 | 8 | 277 | 31.3 | 5.59 |
| P45880 | Voltage-dependent anion-selective channel protein 2 OS=Homo sapiens GN=VDAC2 PE=1 SV=2 - [VDAC2_HUMAN] | 8 | 6 | 7 | 294 | 31.5 | 7.56 |
| Q13347 | Eukaryotic translation initiation factor 3 subunit I OS=Homo sapiens GN=EIF3I PE=1 SV=1 - [EIF3I_HUMAN] | 8 | 7 | 8 | 325 | 36.5 | 5.64 |
| Q00325 | Phosphate carrier protein. mitochondrial OS=Homo sapiens GN=SLC25A3 PE=1 SV=2 - [MPCP_HUMAN] | 8 | 7 | 6 | 362 | 40.1 | 9.38 |
| P61158 | Actin-related protein 3 OS=Homo sapiens GN=ACTR3 PE=1 SV=3 - [ARP3_HUMAN] | 8 | 9 | 6 | 418 | 47.3 | 5.88 |
| Q9NVA2 | Septin-11 OS=Homo sapiens GN=SEPT11 PE=1 SV=3 - [SEP11_HUMAN] | 8 | 8 | 10 | 429 | 49.4 | 6.81 |
| Q02978 | Mitochondrial 2-oxoglutarate/malate carrier protein OS=Homo sapiens GN=SLC25A11 PE=1 SV=3 - [M2OM_HUMAN] | 8 | 6 | 11 | 314 | 34.0 | 9.91 |
| P38919 | Eukaryotic initiation factor 4A-III OS=Homo sapiens GN=EIF4A3 PE=1 SV=4 - [IF4A3_HUMAN] | 8 | 13 | 13 | 411 | 46.8 | 6.73 |
| Q93099 | Homogentisate 1.2-dioxygenase OS=Homo sapiens GN=HGD PE=1 SV=2 - [HGD_HUMAN] | 8 | 4 | 7 | 445 | 49.9 | 6.96 |
| Q03154 | Aminoacylase-1 OS=Homo sapiens GN=ACY1 PE=1 SV=1 - [ACY1_HUMAN] | 8 | 7 | 9 | 408 | 45.9 | 6.18 |
| O00232 | 26S proteasome non-ATPase regulatory subunit 12 OS=Homo sapiens GN=PSMD12 PE=1 SV=3 - [PSD12_HUMAN] | 8 | 8 | 10 | 456 | 52.9 | 7.65 |
| Q9Y265 | RuvB-like 1 OS=Homo sapiens GN=RUVBL1 PE=1 SV=1 - [RUVB1_HUMAN] | 8 | 9 | 10 | 456 | 50.2 | 6.42 |
| Q9ULV4 | Coronin-1C OS=Homo sapiens GN=CORO1C PE=1 SV=1 - [COR1C_HUMAN] | 8 | 3 | 7 | 474 | 53.2 | 7.08 |
| P00505 | Aspartate aminotransferase. mitochondrial OS=Homo sapiens GN=GOT2 PE=1 SV=3 - [AATM_HUMAN] | 8 | 9 | 8 | 430 | 47.5 | 9.01 |
| P07339 | Cathepsin D OS=Homo sapiens GN=CTSD PE=1 SV=1 - [CATD_HUMAN] | 8 | 7 | 9 | 412 | 44.5 | 6.54 |
| P38159 | Heterogeneous nuclear ribonucleoprotein G OS=Homo sapiens GN=RBMX PE=1 SV=3 - [HNRPG_HUMAN] | 8 | 8 | 10 | 391 | 42.3 | 10.05 |
| Q16850 | Lanosterol 14-alpha demethylase OS=Homo sapiens GN=CYP51A1 PE=1 SV=3 - [CP51A_HUMAN] | 8 | 10 | 11 | 503 | 56.8 | 8.53 |
| P38606 | V-type proton ATPase catalytic subunit A OS=Homo sapiens GN=ATP6V1A PE=1 SV=2 - [VATA_HUMAN] | 8 | 8 | 10 | 617 | 68.3 | 5.52 |
| P08195 | 4F2 cell-surface antigen heavy chain OS=Homo sapiens GN=SLC3A2 PE=1 SV=3 - [4F2_HUMAN] | 8 | 9 | 8 | 630 | 68.0 | 5.01 |
| P46060 | Ran GTPase-activating protein 1 OS=Homo sapiens GN=RANGAP1 PE=1 SV=1 - [RAGP1_HUMAN] | 8 | 5 | 8 | 587 | 63.5 | 4.68 |
| Q9BZZ5 | Apoptosis inhibitor 5 OS=Homo sapiens GN=API5 PE=1 SV=3 - [API5_HUMAN] | 8 | 8 | 8 | 524 | 59.0 | 7.34 |
| P11413 | Glucose-6-phosphate 1-dehydrogenase OS=Homo sapiens GN=G6PD PE=1 SV=4 - [G6PD_HUMAN] | 8 | 7 | 6 | 515 | 59.2 | 6.84 |
| O43242 | 26S proteasome non-ATPase regulatory subunit 3 OS=Homo sapiens GN=PSMD3 PE=1 SV=2 - [PSMD3_HUMAN] | 8 | 7 | 8 | 534 | 60.9 | 8.44 |
| Q96G03 | Phosphoglucomutase-2 OS=Homo sapiens GN=PGM2 PE=1 SV=4 - [PGM2_HUMAN] | 8 | 9 | 9 | 612 | 68.2 | 6.73 |
| O00429 | Dynamin-1-like protein OS=Homo sapiens GN=DNM1L PE=1 SV=2 - [DNM1L_HUMAN] | 8 | 6 | 8 | 736 | 81.8 | 6.81 |
| Q12931 | Heat shock protein 75 kDa. mitochondrial OS=Homo sapiens GN=TRAP1 PE=1 SV=3 - [TRAP1_HUMAN] | 8 | 8 | 11 | 704 | 80.1 | 8.21 |
| Q96PK6 | RNA-binding protein 14 OS=Homo sapiens GN=RBM14 PE=1 SV=2 - [RBM14_HUMAN] | 8 | 7 | 8 | 669 | 69.4 | 9.67 |
| P33992 | DNA replication licensing factor MCM5 OS=Homo sapiens GN=MCM5 PE=1 SV=5 - [MCM5_HUMAN] | 8 | 4 | 8 | 734 | 82.2 | 8.37 |
| Q15459 | Splicing factor 3A subunit 1 OS=Homo sapiens GN=SF3A1 PE=1 SV=1 - [SF3A1_HUMAN] | 8 | 5 | 6 | 793 | 88.8 | 5.22 |
| Q9NR30 | Nucleolar RNA helicase 2 OS=Homo sapiens GN=DDX21 PE=1 SV=5 - [DDX21_HUMAN] | 8 | 9 | 9 | 783 | 87.3 | 9.28 |
| Q13616 | Cullin-1 OS=Homo sapiens GN=CUL1 PE=1 SV=2 - [CUL1_HUMAN] | 8 | 6 | 7 | 776 | 89.6 | 8.00 |
| P48147 | Prolyl endopeptidase OS=Homo sapiens GN=PREP PE=1 SV=2 - [PPCE_HUMAN] | 8 | 6 | 8 | 710 | 80.6 | 5.86 |
| Q9Y5L0 | Transportin-3 OS=Homo sapiens GN=TNPO3 PE=1 SV=3 - [TNPO3_HUMAN] | 8 | 6 | 7 | 923 | 104.1 | 5.57 |
| Q15029 | 116 kDa U5 small nuclear ribonucleoprotein component OS=Homo sapiens GN=EFTUD2 PE=1 SV=1 - [U5S1_HUMAN] | 8 | 9 | 10 | 972 | 109.4 | 5.00 |
| P26639 | Threonyl-tRNA synthetase. cytoplasmic OS=Homo sapiens GN=TARS PE=1 SV=3 - [SYTC_HUMAN] | 8 | 3 | 7 | 723 | 83.4 | 6.67 |
| Q9H0D6 | 5'-3' exoribonuclease 2 OS=Homo sapiens GN=XRN2 PE=1 SV=1 - [XRN2_HUMAN] | 8 | 7 | 7 | 950 | 108.5 | 7.47 |
| P46977 | Dolichyl-diphosphooligosaccharide--protein glycosyltransferase subunit STT3A OS=Homo sapiens GN=STT3A PE=1 SV=2 - [STT3A_HUMAN] | 8 | 9 | 8 | 705 | 80.5 | 8.07 |
| P22102 | Trifunctional purine biosynthetic protein adenosine-3 OS=Homo sapiens GN=GART PE=1 SV=1 - [PUR2_HUMAN] | 8 | 7 | 7 | 1010 | 107.7 | 6.70 |
| P78344 | Eukaryotic translation initiation factor 4 gamma 2 OS=Homo sapiens GN=EIF4G2 PE=1 SV=1 - [IF4G2_HUMAN] | 8 | 6 | 6 | 907 | 102.3 | 7.14 |
| Q99460 | 26S proteasome non-ATPase regulatory subunit 1 OS=Homo sapiens GN=PSMD1 PE=1 SV=2 - [PSMD1_HUMAN] | 8 | 9 | 10 | 953 | 105.8 | 5.39 |
| Q9UDY2 | Tight junction protein ZO-2 OS=Homo sapiens GN=TJP2 PE=1 SV=2 - [ZO2_HUMAN] | 8 | 4 | 10 | 1190 | 133.9 | 7.40 |
| O60841 | Eukaryotic translation initiation factor 5B OS=Homo sapiens GN=EIF5B PE=1 SV=4 - [IF2P_HUMAN] | 8 | 5 | 8 | 1220 | 138.7 | 5.49 |
| P53992 | Protein transport protein Sec24C OS=Homo sapiens GN=SEC24C PE=1 SV=3 - [SC24C_HUMAN] | 8 | 9 | 9 | 1094 | 118.2 | 7.06 |
| P55265 | Double-stranded RNA-specific adenosine deaminase OS=Homo sapiens GN=ADAR PE=1 SV=4 - [DSRAD_HUMAN] | 8 | 6 | 7 | 1226 | 136.0 | 8.65 |
| P00533 | Epidermal growth factor receptor OS=Homo sapiens GN=EGFR PE=1 SV=2 - [EGFR_HUMAN] | 8 | 9 | 9 | 1210 | 134.2 | 6.68 |
| O95486 | Protein transport protein Sec24A OS=Homo sapiens GN=SEC24A PE=1 SV=2 - [SC24A_HUMAN] | 8 | 9 | 7 | 1093 | 119.7 | 7.66 |
| O75976 | Carboxypeptidase D OS=Homo sapiens GN=CPD PE=1 SV=2 - [CBPD_HUMAN] | 8 | 6 | 6 | 1380 | 152.8 | 6.05 |
| P61088 | Ubiquitin-conjugating enzyme E2 N OS=Homo sapiens GN=UBE2N PE=1 SV=1 - [UBE2N_HUMAN] | 7 | 8 | 10 | 152 | 17.1 | 6.57 |
| P07741 | Adenine phosphoribosyltransferase OS=Homo sapiens GN=APRT PE=1 SV=2 - [APT_HUMAN] | 7 | 6 | 8 | 180 | 19.6 | 6.02 |
| P56537 | Eukaryotic translation initiation factor 6 OS=Homo sapiens GN=EIF6 PE=1 SV=1 - [IF6_HUMAN] | 7 | 8 | 7 | 245 | 26.6 | 4.68 |
| P27144 | Adenylate kinase isoenzyme 4. mitochondrial OS=Homo sapiens GN=AK3L1 PE=1 SV=1 - [KAD4_HUMAN] | 7 | 8 | 7 | 223 | 25.3 | 8.40 |
| P67809 | Nuclease-sensitive element-binding protein 1 OS=Homo sapiens GN=YBX1 PE=1 SV=3 - [YBOX1_HUMAN] | 7 | 13 | 12 | 324 | 35.9 | 9.88 |
| P67936 | Tropomyosin alpha-4 chain OS=Homo sapiens GN=TPM4 PE=1 SV=3 - [TPM4_HUMAN] | 7 | 12 | 10 | 248 | 28.5 | 4.69 |
| P32969 | 60S ribosomal protein L9 OS=Homo sapiens GN=RPL9 PE=1 SV=1 - [RL9_HUMAN] | 7 | 11 | 10 | 192 | 21.8 | 9.95 |
| Q15366 | Poly(rC)-binding protein 2 OS=Homo sapiens GN=PCBP2 PE=1 SV=1 - [PCBP2_HUMAN] | 7 | 12 | 10 | 365 | 38.6 | 6.79 |
| O75347 | Tubulin-specific chaperone A OS=Homo sapiens GN=TBCA PE=1 SV=3 - [TBCA_HUMAN] | 7 | 9 | 6 | 108 | 12.8 | 5.29 |
| Q04760 | Lactoylglutathione lyase OS=Homo sapiens GN=GLO1 PE=1 SV=4 - [LGUL_HUMAN] | 7 | 5 | 8 | 184 | 20.8 | 5.31 |
| Q13162 | Peroxiredoxin-4 OS=Homo sapiens GN=PRDX4 PE=1 SV=1 - [PRDX4_HUMAN] | 7 | 11 | 12 | 271 | 30.5 | 6.29 |
| P61981 | 14-3-3 protein gamma OS=Homo sapiens GN=YWHAG PE=1 SV=2 - [1433G_HUMAN] | 7 | 17 | 14 | 247 | 28.3 | 4.89 |
| O43809 | Cleavage and polyadenylation specificity factor subunit 5 OS=Homo sapiens GN=NUDT21 PE=1 SV=1 - [CPSF5_HUMAN] | 7 | 9 | 9 | 227 | 26.2 | 8.82 |
| Q9NVJ2 | ADP-ribosylation factor-like protein 8B OS=Homo sapiens GN=ARL8B PE=1 SV=1 - [ARL8B_HUMAN] | 7 | 4 | 9 | 186 | 21.5 | 8.43 |
| P27105 | Erythrocyte band 7 integral membrane protein OS=Homo sapiens GN=STOM PE=1 SV=3 - [STOM_HUMAN] | 7 | 7 | 8 | 288 | 31.7 | 7.88 |
| P29966 | Myristoylated alanine-rich C-kinase substrate OS=Homo sapiens GN=MARCKS PE=1 SV=4 - [MARCS_HUMAN] | 7 | 8 | 8 | 332 | 31.5 | 4.45 |
| Q9Y5M8 | Signal recognition particle receptor subunit beta OS=Homo sapiens GN=SRPRB PE=1 SV=3 - [SRPRB_HUMAN] | 7 | 4 | 6 | 271 | 29.7 | 9.04 |
| Q15907 | Ras-related protein Rab-11B OS=Homo sapiens GN=RAB11B PE=1 SV=4 - [RB11B_HUMAN] | 7 | 7 | 7 | 218 | 24.5 | 5.94 |
| P62241 | 40S ribosomal protein S8 OS=Homo sapiens GN=RPS8 PE=1 SV=2 - [RS8_HUMAN] | 7 | 6 | 8 | 208 | 24.2 | 10.32 |
| O95861 | 3'(2').5'-bisphosphate nucleotidase 1 OS=Homo sapiens GN=BPNT1 PE=1 SV=1 - [BPNT1_HUMAN] | 7 | 6 | 6 | 308 | 33.4 | 5.69 |
| P62277 | 40S ribosomal protein S13 OS=Homo sapiens GN=RPS13 PE=1 SV=2 - [RS13_HUMAN] | 7 | 6 | 6 | 151 | 17.2 | 10.54 |
| P61313 | 60S ribosomal protein L15 OS=Homo sapiens GN=RPL15 PE=1 SV=2 - [RL15_HUMAN] | 7 | 5 | 9 | 204 | 24.1 | 11.62 |
| Q15274 | Nicotinate-nucleotide pyrophosphorylase [carboxylating] OS=Homo sapiens GN=QPRT PE=1 SV=3 - [NADC_HUMAN] | 7 | 9 | 8 | 297 | 30.8 | 6.21 |
| Q92597 | Protein NDRG1 OS=Homo sapiens GN=NDRG1 PE=1 SV=1 - [NDRG1_HUMAN] | 7 | 6 | 9 | 394 | 42.8 | 5.82 |
| Q53H82 | Beta-lactamase-like protein 2 OS=Homo sapiens GN=LACTB2 PE=1 SV=2 - [LACB2_HUMAN] | 7 | 6 | 5 | 288 | 32.8 | 6.80 |
| P25787 | Proteasome subunit alpha type-2 OS=Homo sapiens GN=PSMA2 PE=1 SV=2 - [PSA2_HUMAN] | 7 | 12 | 13 | 234 | 25.9 | 7.43 |
| P36542 | ATP synthase subunit gamma. mitochondrial OS=Homo sapiens GN=ATP5C1 PE=1 SV=1 - [ATPG_HUMAN] | 7 | 8 | 11 | 298 | 33.0 | 9.22 |
| O00151 | PDZ and LIM domain protein 1 OS=Homo sapiens GN=PDLIM1 PE=1 SV=4 - [PDLI1_HUMAN] | 7 | 8 | 5 | 329 | 36.0 | 7.02 |
| P00387 | NADH-cytochrome b5 reductase 3 OS=Homo sapiens GN=CYB5R3 PE=1 SV=3 - [NB5R3_HUMAN] | 7 | 8 | 9 | 301 | 34.2 | 7.59 |
| Q07960 | Rho GTPase-activating protein 1 OS=Homo sapiens GN=ARHGAP1 PE=1 SV=1 - [RHG01_HUMAN] | 7 | 6 | 6 | 439 | 50.4 | 6.29 |
| Q13011 | Delta(3.5)-Delta(2.4)-dienoyl-CoA isomerase. mitochondrial OS=Homo sapiens GN=ECH1 PE=1 SV=2 - [ECH1_HUMAN] | 7 | 8 | 9 | 328 | 35.8 | 8.00 |
| O14745 | Na(+)/H(+) exchange regulatory cofactor NHE-RF1 OS=Homo sapiens GN=SLC9A3R1 PE=1 SV=4 - [NHRF1_HUMAN] | 7 | 6 | 7 | 358 | 38.8 | 5.77 |
| Q01105 | Protein SET OS=Homo sapiens GN=SET PE=1 SV=3 - [SET_HUMAN] | 7 | 7 | 7 | 290 | 33.5 | 4.32 |
| Q92820 | Gamma-glutamyl hydrolase OS=Homo sapiens GN=GGH PE=1 SV=2 - [GGH_HUMAN] | 7 | 6 | 9 | 318 | 35.9 | 7.11 |
| P63092 | Guanine nucleotide-binding protein G(s) subunit alpha isoforms short OS=Homo sapiens GN=GNAS PE=1 SV=1 - [GNAS2_HUMAN] | 7 | 8 | 9 | 394 | 45.6 | 5.82 |
| P60900 | Proteasome subunit alpha type-6 OS=Homo sapiens GN=PSMA6 PE=1 SV=1 - [PSA6_HUMAN] | 7 | 10 | 7 | 246 | 27.4 | 6.76 |
| P25325 | 3-mercaptopyruvate sulfurtransferase OS=Homo sapiens GN=MPST PE=1 SV=3 - [THTM_HUMAN] | 7 | 8 | 8 | 297 | 33.2 | 6.60 |
| P30154 | Serine/threonine-protein phosphatase 2A 65 kDa regulatory subunit A beta isoform OS=Homo sapiens GN=PPP2R1B PE=1 SV=3 - [2AAB_HUMAN] | 7 | 15 | 15 | 601 | 66.2 | 4.94 |
| P14550 | Alcohol dehydrogenase [NADP+] OS=Homo sapiens GN=AKR1A1 PE=1 SV=3 - [AK1A1_HUMAN] | 7 | 6 | 7 | 325 | 36.5 | 6.79 |
| Q9Y3F4 | Serine-threonine kinase receptor-associated protein OS=Homo sapiens GN=STRAP PE=1 SV=1 - [STRAP_HUMAN] | 7 | 5 | 8 | 350 | 38.4 | 5.12 |
| P24752 | Acetyl-CoA acetyltransferase. mitochondrial OS=Homo sapiens GN=ACAT1 PE=1 SV=1 - [THIL_HUMAN] | 7 | 8 | 9 | 427 | 45.2 | 8.85 |
| P17980 | 26S protease regulatory subunit 6A OS=Homo sapiens GN=PSMC3 PE=1 SV=3 - [PRS6A_HUMAN] | 7 | 7 | 7 | 439 | 49.2 | 5.24 |
| P62195 | 26S protease regulatory subunit 8 OS=Homo sapiens GN=PSMC5 PE=1 SV=1 - [PRS8_HUMAN] | 7 | 4 | 9 | 406 | 45.6 | 7.55 |
| Q99956 | Dual specificity protein phosphatase 9 OS=Homo sapiens GN=DUSP9 PE=1 SV=1 - [DUS9_HUMAN] | 7 | 7 | 8 | 384 | 41.8 | 6.11 |
| P30566 | Adenylosuccinate lyase OS=Homo sapiens GN=ADSL PE=1 SV=2 - [PUR8_HUMAN] | 7 | 7 | 8 | 484 | 54.9 | 7.11 |
| Q15392 | 24-dehydrocholesterol reductase OS=Homo sapiens GN=DHCR24 PE=1 SV=2 - [DHC24_HUMAN] | 7 | 5 | 6 | 516 | 60.1 | 8.16 |
| Q9BXS5 | AP-1 complex subunit mu-1 OS=Homo sapiens GN=AP1M1 PE=1 SV=3 - [AP1M1_HUMAN] | 7 | 7 | 9 | 423 | 48.6 | 7.30 |
| P07099 | Epoxide hydrolase 1 OS=Homo sapiens GN=EPHX1 PE=1 SV=1 - [HYEP_HUMAN] | 7 | 5 | 5 | 455 | 52.9 | 7.25 |
| Q13561 | Dynactin subunit 2 OS=Homo sapiens GN=DCTN2 PE=1 SV=4 - [DCTN2_HUMAN] | 7 | 7 | 7 | 401 | 44.2 | 5.21 |
| Q8NBS9 | Thioredoxin domain-containing protein 5 OS=Homo sapiens GN=TXNDC5 PE=1 SV=2 - [TXND5_HUMAN] | 7 | 6 | 7 | 432 | 47.6 | 5.97 |
| Q3LXA3 | Triokinase/FMN cyclase OS=Homo sapiens GN=TKFC PE=1 SV=2 - [TKFC_HUMAN] | 7 | 7 | 4 | 575 | 58.9 | 7.49 |
| Q7Z434 | Mitochondrial antiviral-signaling protein OS=Homo sapiens GN=MAVS PE=1 SV=2 - [MAVS_HUMAN] | 7 | 6 | 6 | 540 | 56.5 | 5.52 |
| Q99856 | AT-rich interactive domain-containing protein 3A OS=Homo sapiens GN=ARID3A PE=1 SV=2 - [ARI3A_HUMAN] | 7 | 5 | 8 | 593 | 62.9 | 4.91 |
| P23588 | Eukaryotic translation initiation factor 4B OS=Homo sapiens GN=EIF4B PE=1 SV=2 - [IF4B_HUMAN] | 7 | 6 | 5 | 611 | 69.1 | 5.73 |
| Q9NTK5 | Obg-like ATPase 1 OS=Homo sapiens GN=OLA1 PE=1 SV=2 - [OLA1_HUMAN] | 7 | 7 | 7 | 396 | 44.7 | 7.81 |
| P05362 | Intercellular adhesion molecule 1 OS=Homo sapiens GN=ICAM1 PE=1 SV=2 - [ICAM1_HUMAN] | 7 | 8 | 6 | 532 | 57.8 | 7.99 |
| P51812 | Ribosomal protein S6 kinase alpha-3 OS=Homo sapiens GN=RPS6KA3 PE=1 SV=1 - [KS6A3_HUMAN] | 7 | 7 | 11 | 740 | 83.7 | 6.89 |
| Q13057 | Bifunctional coenzyme A synthase OS=Homo sapiens GN=COASY PE=1 SV=4 - [COASY_HUMAN] | 7 | 6 | 6 | 564 | 62.3 | 6.99 |
| Q8N1G4 | Leucine-rich repeat-containing protein 47 OS=Homo sapiens GN=LRRC47 PE=1 SV=1 - [LRC47_HUMAN] | 7 | 4 | 7 | 583 | 63.4 | 8.28 |
| O43615 | Mitochondrial import inner membrane translocase subunit TIM44 OS=Homo sapiens GN=TIMM44 PE=1 SV=2 - [TIM44_HUMAN] | 7 | 6 | 7 | 452 | 51.3 | 8.32 |
| O76094 | Signal recognition particle 72 kDa protein OS=Homo sapiens GN=SRP72 PE=1 SV=3 - [SRP72_HUMAN] | 7 | 7 | 5 | 671 | 74.6 | 9.26 |
| Q9UGI8 | Testin OS=Homo sapiens GN=TES PE=1 SV=1 - [TES_HUMAN] | 7 | 5 | 5 | 421 | 48.0 | 7.68 |
| P28288 | ATP-binding cassette sub-family D member 3 OS=Homo sapiens GN=ABCD3 PE=1 SV=1 - [ABCD3_HUMAN] | 7 | 8 | 8 | 659 | 75.4 | 9.36 |
| P43304 | Glycerol-3-phosphate dehydrogenase. mitochondrial OS=Homo sapiens GN=GPD2 PE=1 SV=3 - [GPDM_HUMAN] | 7 | 4 | 6 | 727 | 80.8 | 7.69 |
| Q96AE4 | Far upstream element-binding protein 1 OS=Homo sapiens GN=FUBP1 PE=1 SV=3 - [FUBP1_HUMAN] | 7 | 3 | 10 | 644 | 67.5 | 7.61 |
| O95202 | LETM1 and EF-hand domain-containing protein 1. mitochondrial OS=Homo sapiens GN=LETM1 PE=1 SV=1 - [LETM1_HUMAN] | 7 | 5 | 6 | 739 | 83.3 | 6.70 |
| Q14694 | Ubiquitin carboxyl-terminal hydrolase 10 OS=Homo sapiens GN=USP10 PE=1 SV=2 - [UBP10_HUMAN] | 7 | 4 | 7 | 798 | 87.1 | 5.31 |
| P42224 | Signal transducer and activator of transcription 1-alpha/beta OS=Homo sapiens GN=STAT1 PE=1 SV=2 - [STAT1_HUMAN] | 7 | 6 | 7 | 750 | 87.3 | 6.05 |
| O95793 | Double-stranded RNA-binding protein Staufen homolog 1 OS=Homo sapiens GN=STAU1 PE=1 SV=2 - [STAU1_HUMAN] | 7 | 4 | 7 | 577 | 63.1 | 9.44 |
| Q9BXP5 | Serrate RNA effector molecule homolog OS=Homo sapiens GN=SRRT PE=1 SV=1 - [SRRT_HUMAN] | 7 | 7 | 5 | 876 | 100.6 | 5.96 |
| P50570 | Dynamin-2 OS=Homo sapiens GN=DNM2 PE=1 SV=2 - [DYN2_HUMAN] | 7 | 3 | 9 | 870 | 98.0 | 7.44 |
| Q92598 | Heat shock protein 105 kDa OS=Homo sapiens GN=HSPH1 PE=1 SV=1 - [HS105_HUMAN] | 7 | 4 | 9 | 858 | 96.8 | 5.39 |
| Q9NVP1 | ATP-dependent RNA helicase DDX18 OS=Homo sapiens GN=DDX18 PE=1 SV=2 - [DDX18_HUMAN] | 7 | 6 | 6 | 670 | 75.4 | 9.50 |
| Q13492 | Phosphatidylinositol-binding clathrin assembly protein OS=Homo sapiens GN=PICALM PE=1 SV=2 - [PICAL_HUMAN] | 7 | 5 | 4 | 652 | 70.7 | 7.90 |
| P17812 | CTP synthase 1 OS=Homo sapiens GN=CTPS PE=1 SV=2 - [PYRG1_HUMAN] | 7 | 6 | 9 | 591 | 66.6 | 6.46 |
| Q96P70 | Importin-9 OS=Homo sapiens GN=IPO9 PE=1 SV=3 - [IPO9_HUMAN] | 7 | 6 | 11 | 1041 | 115.9 | 4.81 |
| Q9NSD9 | Phenylalanine--tRNA ligase beta subunit OS=Homo sapiens GN=FARSB PE=1 SV=3 - [SYFB_HUMAN] | 7 | 5 | 7 | 589 | 66.1 | 6.84 |
| P43155 | Carnitine O-acetyltransferase OS=Homo sapiens GN=CRAT PE=1 SV=5 - [CACP_HUMAN] | 7 | 3 | 6 | 626 | 70.8 | 8.44 |
| P46379 | Large proline-rich protein BAT3 OS=Homo sapiens GN=BAT3 PE=1 SV=2 - [BAT3_HUMAN] | 7 | 7 | 6 | 1132 | 119.3 | 5.60 |
| Q8N766 | Uncharacterized protein KIAA0090 OS=Homo sapiens GN=KIAA0090 PE=1 SV=1 - [K0090_HUMAN] | 7 | 2 | 7 | 993 | 111.7 | 7.66 |
| P49321 | Nuclear autoantigenic sperm protein OS=Homo sapiens GN=NASP PE=1 SV=2 - [NASP_HUMAN] | 7 | 5 | 10 | 788 | 85.2 | 4.30 |
| Q99959 | Plakophilin-2 OS=Homo sapiens GN=PKP2 PE=1 SV=2 - [PKP2_HUMAN] | 7 | 4 | 6 | 881 | 97.4 | 9.33 |
| Q29RF7 | Sister chromatid cohesion protein PDS5 homolog A OS=Homo sapiens GN=PDS5A PE=1 SV=1 - [PDS5A_HUMAN] | 7 | 9 | 9 | 1337 | 150.7 | 7.91 |
| Q27J81 | Inverted formin-2 OS=Homo sapiens GN=INF2 PE=1 SV=2 - [INF2_HUMAN] | 7 | 5 | 7 | 1249 | 135.5 | 5.38 |
| Q15020 | Squamous cell carcinoma antigen recognized by T-cells 3 OS=Homo sapiens GN=SART3 PE=1 SV=1 - [SART3_HUMAN] | 7 | 4 | 5 | 963 | 109.9 | 5.57 |
| Q9UIA9 | Exportin-7 OS=Homo sapiens GN=XPO7 PE=1 SV=3 - [XPO7_HUMAN] | 7 | 4 | 7 | 1087 | 123.8 | 6.32 |
| O60271 | C-Jun-amino-terminal kinase-interacting protein 4 OS=Homo sapiens GN=SPAG9 PE=1 SV=4 - [JIP4_HUMAN] | 7 | 7 | 6 | 1321 | 146.1 | 5.15 |
| A0AVT1 | Ubiquitin-like modifier-activating enzyme 6 OS=Homo sapiens GN=UBA6 PE=1 SV=1 - [UBA6_HUMAN] | 7 | 5 | 4 | 1052 | 117.9 | 6.14 |
| O75694 | Nuclear pore complex protein Nup155 OS=Homo sapiens GN=NUP155 PE=1 SV=1 - [NU155_HUMAN] | 7 | 4 | 7 | 1391 | 155.1 | 6.16 |
| Q8TF05 | Serine/threonine-protein phosphatase 4 regulatory subunit 1 OS=Homo sapiens GN=PPP4R1 PE=1 SV=1 - [PP4R1_HUMAN] | 7 | 5 | 6 | 950 | 106.9 | 4.77 |
| Q7L2E3 | Putative ATP-dependent RNA helicase DHX30 OS=Homo sapiens GN=DHX30 PE=1 SV=1 - [DHX30_HUMAN] | 7 | 7 | 7 | 1194 | 133.9 | 8.78 |
| Q15155 | Nodal modulator 1 OS=Homo sapiens GN=NOMO1 PE=1 SV=5 - [NOMO1_HUMAN] | 7 | 6 | 6 | 1222 | 134.2 | 5.81 |
| Q5VYK3 | Proteasome-associated protein ECM29 homolog OS=Homo sapiens GN=ECM29 PE=1 SV=2 - [ECM29_HUMAN] | 7 | 7 | 6 | 1845 | 204.2 | 7.12 |
| P84077 | ADP-ribosylation factor 1 OS=Homo sapiens GN=ARF1 PE=1 SV=2 - [ARF1_HUMAN] | 6 | 14 | 14 | 181 | 20.7 | 6.80 |
| Q9NYL4 | Peptidyl-prolyl cis-trans isomerase FKBP11 OS=Homo sapiens GN=FKBP11 PE=1 SV=1 - [FKB11_HUMAN] | 6 | 6 | 11 | 201 | 22.2 | 9.39 |
| P62316 | Small nuclear ribonucleoprotein Sm D2 OS=Homo sapiens GN=SNRPD2 PE=1 SV=1 - [SMD2_HUMAN] | 6 | 3 | 6 | 118 | 13.5 | 9.91 |
| P61604 | 10 kDa heat shock protein. mitochondrial OS=Homo sapiens GN=HSPE1 PE=1 SV=2 - [CH10_HUMAN] | 6 | 7 | 7 | 102 | 10.9 | 8.92 |
| P30050 | 60S ribosomal protein L12 OS=Homo sapiens GN=RPL12 PE=1 SV=1 - [RL12_HUMAN] | 6 | 6 | 8 | 165 | 17.8 | 9.42 |
| O14880 | Microsomal glutathione S-transferase 3 OS=Homo sapiens GN=MGST3 PE=1 SV=1 - [MGST3_HUMAN] | 6 | 9 | 10 | 152 | 16.5 | 9.38 |
| P62805 | Histone H4 OS=Homo sapiens GN=HIST1H4A PE=1 SV=2 - [H4_HUMAN] | 6 | 9 | 8 | 103 | 11.4 | 11.36 |
| P62263 | 40S ribosomal protein S14 OS=Homo sapiens GN=RPS14 PE=1 SV=3 - [RS14_HUMAN] | 6 | 7 | 6 | 151 | 16.3 | 10.05 |
| O75947 | ATP synthase subunit d. mitochondrial OS=Homo sapiens GN=ATP5H PE=1 SV=3 - [ATP5H_HUMAN] | 6 | 4 | 4 | 161 | 18.5 | 5.30 |
| P60953 | Cell division control protein 42 homolog OS=Homo sapiens GN=CDC42 PE=1 SV=2 - [CDC42_HUMAN] | 6 | 6 | 10 | 191 | 21.2 | 6.55 |
| P49721 | Proteasome subunit beta type-2 OS=Homo sapiens GN=PSMB2 PE=1 SV=1 - [PSB2_HUMAN] | 6 | 3 | 6 | 201 | 22.8 | 7.02 |
| Q6ZN17 | Protein lin-28 homolog B OS=Homo sapiens GN=LIN28B PE=1 SV=1 - [LN28B_HUMAN] | 6 | 3 | 5 | 250 | 27.1 | 8.91 |
| Q9BUF5 | Tubulin beta-6 chain OS=Homo sapiens GN=TUBB6 PE=1 SV=1 - [TBB6_HUMAN] | 6 | 27 | 29 | 446 | 49.8 | 4.88 |
| P47755 | F-actin-capping protein subunit alpha-2 OS=Homo sapiens GN=CAPZA2 PE=1 SV=3 - [CAZA2_HUMAN] | 6 | 4 | 8 | 286 | 32.9 | 5.85 |
| P62280 | 40S ribosomal protein S11 OS=Homo sapiens GN=RPS11 PE=1 SV=3 - [RS11_HUMAN] | 6 | 4 | 7 | 158 | 18.4 | 10.30 |
| Q99685 | Monoglyceride lipase OS=Homo sapiens GN=MGLL PE=1 SV=2 - [MGLL_HUMAN] | 6 | 5 | 7 | 303 | 33.2 | 6.99 |
| P06493 | Cyclin-dependent kinase 1 OS=Homo sapiens GN=CDK1 PE=1 SV=3 - [CDK1_HUMAN] | 6 | 9 | 8 | 297 | 34.1 | 8.40 |
| Q9NQR4 | Omega-amidase NIT2 OS=Homo sapiens GN=NIT2 PE=1 SV=1 - [NIT2_HUMAN] | 6 | 5 | 7 | 276 | 30.6 | 7.21 |
| P30084 | Enoyl-CoA hydratase. mitochondrial OS=Homo sapiens GN=ECHS1 PE=1 SV=4 - [ECHM_HUMAN] | 6 | 7 | 10 | 290 | 31.4 | 8.07 |
| P18621 | 60S ribosomal protein L17 OS=Homo sapiens GN=RPL17 PE=1 SV=3 - [RL17_HUMAN] | 6 | 6 | 6 | 184 | 21.4 | 10.17 |
| Q9Y277 | Voltage-dependent anion-selective channel protein 3 OS=Homo sapiens GN=VDAC3 PE=1 SV=1 - [VDAC3_HUMAN] | 6 | 8 | 10 | 283 | 30.6 | 8.66 |
| Q9Y224 | UPF0568 protein C14orf166 OS=Homo sapiens GN=C14orf166 PE=1 SV=1 - [CN166_HUMAN] | 6 | 8 | 7 | 244 | 28.1 | 6.65 |
| Q9UBQ0 | Vacuolar protein sorting-associated protein 29 OS=Homo sapiens GN=VPS29 PE=1 SV=1 - [VPS29_HUMAN] | 6 | 4 | 5 | 182 | 20.5 | 6.79 |
| P00491 | Purine nucleoside phosphorylase OS=Homo sapiens GN=NP PE=1 SV=2 - [PNPH_HUMAN] | 6 | 8 | 7 | 289 | 32.1 | 6.95 |
| O43396 | Thioredoxin-like protein 1 OS=Homo sapiens GN=TXNL1 PE=1 SV=3 - [TXNL1_HUMAN] | 6 | 6 | 6 | 289 | 32.2 | 4.96 |
| P00918 | Carbonic anhydrase 2 OS=Homo sapiens GN=CA2 PE=1 SV=2 - [CAH2_HUMAN] | 6 | 5 | 5 | 260 | 29.2 | 7.40 |
| Q99536 | Synaptic vesicle membrane protein VAT-1 homolog OS=Homo sapiens GN=VAT1 PE=1 SV=2 - [VAT1_HUMAN] | 6 | 3 | 7 | 393 | 41.9 | 6.29 |
| P62753 | 40S ribosomal protein S6 OS=Homo sapiens GN=RPS6 PE=1 SV=1 - [RS6_HUMAN] | 6 | 9 | 10 | 249 | 28.7 | 10.84 |
| P05198 | Eukaryotic translation initiation factor 2 subunit 1 OS=Homo sapiens GN=EIF2S1 PE=1 SV=3 - [IF2A_HUMAN] | 6 | 7 | 6 | 315 | 36.1 | 5.08 |
| P06748 | Nucleophosmin OS=Homo sapiens GN=NPM1 PE=1 SV=2 - [NPM_HUMAN] | 6 | 9 | 8 | 294 | 32.6 | 4.78 |
| P51665 | 26S proteasome non-ATPase regulatory subunit 7 OS=Homo sapiens GN=PSMD7 PE=1 SV=2 - [PSD7_HUMAN] | 6 | 9 | 10 | 324 | 37.0 | 6.77 |
| P17931 | Galectin-3 OS=Homo sapiens GN=LGALS3 PE=1 SV=5 - [LEG3_HUMAN] | 6 | 11 | 13 | 250 | 26.1 | 8.56 |
| P22087 | rRNA 2'-O-methyltransferase fibrillarin OS=Homo sapiens GN=FBL PE=1 SV=2 - [FBRL_HUMAN] | 6 | 5 | 4 | 321 | 33.8 | 10.18 |
| Q9BWD1 | Acetyl-CoA acetyltransferase. cytosolic OS=Homo sapiens GN=ACAT2 PE=1 SV=2 - [THIC_HUMAN] | 6 | 9 | 10 | 397 | 41.3 | 6.92 |
| P24941 | Cell division protein kinase 2 OS=Homo sapiens GN=CDK2 PE=1 SV=2 - [CDK2_HUMAN] | 6 | 3 | 8 | 298 | 33.9 | 8.68 |
| Q15019 | Septin-2 OS=Homo sapiens GN=SEPT2 PE=1 SV=1 - [SEPT2_HUMAN] | 6 | 6 | 7 | 361 | 41.5 | 6.60 |
| Q8NBQ5 | Estradiol 17-beta-dehydrogenase 11 OS=Homo sapiens GN=HSD17B11 PE=1 SV=3 - [DHB11_HUMAN] | 6 | 5 | 7 | 300 | 32.9 | 9.07 |
| P30040 | Endoplasmic reticulum resident protein 29 OS=Homo sapiens GN=ERP29 PE=1 SV=4 - [ERP29_HUMAN] | 6 | 3 | 7 | 261 | 29.0 | 7.31 |
| O15372 | Eukaryotic translation initiation factor 3 subunit H OS=Homo sapiens GN=EIF3H PE=1 SV=1 - [EIF3H_HUMAN] | 6 | 5 | 5 | 352 | 39.9 | 6.54 |
| Q9Y3A5 | Ribosome maturation protein SBDS OS=Homo sapiens GN=SBDS PE=1 SV=4 - [SBDS_HUMAN] | 6 | 5 | 3 | 250 | 28.7 | 8.75 |
| Q9Y266 | Nuclear migration protein nudC OS=Homo sapiens GN=NUDC PE=1 SV=1 - [NUDC_HUMAN] | 6 | 5 | 6 | 331 | 38.2 | 5.38 |
| P12004 | Proliferating cell nuclear antigen OS=Homo sapiens GN=PCNA PE=1 SV=1 - [PCNA_HUMAN] | 6 | 7 | 5 | 261 | 28.8 | 4.69 |
| Q969X5 | Endoplasmic reticulum-Golgi intermediate compartment protein 1 OS=Homo sapiens GN=ERGIC1 PE=1 SV=1 - [ERGI1_HUMAN] | 6 | 5 | 5 | 290 | 32.6 | 7.06 |
| Q7L2H7 | Eukaryotic translation initiation factor 3 subunit M OS=Homo sapiens GN=EIF3M PE=1 SV=1 - [EIF3M_HUMAN] | 6 | 5 | 6 | 374 | 42.5 | 5.63 |
| P05026 | Sodium/potassium-transporting ATPase subunit beta-1 OS=Homo sapiens GN=ATP1B1 PE=1 SV=1 - [AT1B1_HUMAN] | 6 | 5 | 4 | 303 | 35.0 | 8.53 |
| O00303 | Eukaryotic translation initiation factor 3 subunit F OS=Homo sapiens GN=EIF3F PE=1 SV=1 - [EIF3F_HUMAN] | 6 | 11 | 9 | 357 | 37.5 | 5.45 |
| Q07955 | Splicing factor. arginine/serine-rich 1 OS=Homo sapiens GN=SFRS1 PE=1 SV=2 - [SFRS1_HUMAN] | 6 | 7 | 8 | 248 | 27.7 | 10.36 |
| O60884 | DnaJ homolog subfamily A member 2 OS=Homo sapiens GN=DNAJA2 PE=1 SV=1 - [DNJA2_HUMAN] | 6 | 5 | 6 | 412 | 45.7 | 6.48 |
| P50453 | Serpin B9 OS=Homo sapiens GN=SERPINB9 PE=1 SV=1 - [SPB9_HUMAN] | 6 | 6 | 7 | 376 | 42.4 | 5.86 |
| P31689 | DnaJ homolog subfamily A member 1 OS=Homo sapiens GN=DNAJA1 PE=1 SV=2 - [DNJA1_HUMAN] | 6 | 5 | 7 | 397 | 44.8 | 7.08 |
| P54727 | UV excision repair protein RAD23 homolog B OS=Homo sapiens GN=RAD23B PE=1 SV=1 - [RD23B_HUMAN] | 6 | 7 | 7 | 409 | 43.1 | 4.84 |
| Q99733 | Nucleosome assembly protein 1-like 4 OS=Homo sapiens GN=NAP1L4 PE=1 SV=1 - [NP1L4_HUMAN] | 6 | 4 | 5 | 375 | 42.8 | 4.69 |
| P39023 | 60S ribosomal protein L3 OS=Homo sapiens GN=RPL3 PE=1 SV=2 - [RL3_HUMAN] | 6 | 3 | 7 | 403 | 46.1 | 10.18 |
| P27338 | Amine oxidase [flavin-containing] B OS=Homo sapiens GN=MAOB PE=1 SV=3 - [AOFB_HUMAN] | 6 | 8 | 8 | 520 | 58.7 | 7.50 |
| Q9BS26 | Endoplasmic reticulum resident protein 44 OS=Homo sapiens GN=ERP44 PE=1 SV=1 - [ERP44_HUMAN] | 6 | 3 | 7 | 406 | 46.9 | 5.26 |
| P12081 | Histidyl-tRNA synthetase. cytoplasmic OS=Homo sapiens GN=HARS PE=1 SV=2 - [SYHC_HUMAN] | 6 | 4 | 8 | 509 | 57.4 | 5.88 |
| Q16181 | Septin-7 OS=Homo sapiens GN=SEPT7 PE=1 SV=2 - [SEPT7_HUMAN] | 6 | 6 | 8 | 437 | 50.6 | 8.63 |
| P35237 | Serpin B6 OS=Homo sapiens GN=SERPINB6 PE=1 SV=3 - [SPB6_HUMAN] | 6 | 6 | 5 | 376 | 42.6 | 5.27 |
| P11310 | Medium-chain specific acyl-CoA dehydrogenase. mitochondrial OS=Homo sapiens GN=ACADM PE=1 SV=1 - [ACADM_HUMAN] | 6 | 5 | 8 | 421 | 46.6 | 8.37 |
| P26196 | Probable ATP-dependent RNA helicase DDX6 OS=Homo sapiens GN=DDX6 PE=1 SV=2 - [DDX6_HUMAN] | 6 | 8 | 7 | 483 | 54.4 | 8.66 |
| P11172 | Uridine 5'-monophosphate synthase OS=Homo sapiens GN=UMPS PE=1 SV=1 - [UMPS_HUMAN] | 6 | 4 | 6 | 480 | 52.2 | 7.24 |
| Q9Y394 | Dehydrogenase/reductase SDR family member 7 OS=Homo sapiens GN=DHRS7 PE=1 SV=1 - [DHRS7_HUMAN] | 6 | 8 | 9 | 339 | 38.3 | 8.32 |
| P35520 | Cystathionine beta-synthase OS=Homo sapiens GN=CBS PE=1 SV=2 - [CBS_HUMAN] | 6 | 3 | 6 | 551 | 60.5 | 6.65 |
| P48163 | NADP-dependent malic enzyme OS=Homo sapiens GN=ME1 PE=1 SV=1 - [MAOX_HUMAN] | 6 | 6 | 7 | 572 | 64.1 | 6.13 |
| P13861 | cAMP-dependent protein kinase type II-alpha regulatory subunit OS=Homo sapiens GN=PRKAR2A PE=1 SV=2 - [KAP2_HUMAN] | 6 | 4 | 8 | 404 | 45.5 | 5.07 |
| O00425 | Insulin-like growth factor 2 mRNA-binding protein 3 OS=Homo sapiens GN=IGF2BP3 PE=1 SV=2 - [IF2B3_HUMAN] | 6 | 6 | 8 | 579 | 63.7 | 8.87 |
| P45954 | Short/branched chain specific acyl-CoA dehydrogenase. mitochondrial OS=Homo sapiens GN=ACADSB PE=1 SV=1 - [ACDSB_HUMAN] | 6 | 7 | 9 | 432 | 47.5 | 6.99 |
| Q6YN16 | Hydroxysteroid dehydrogenase-like protein 2 OS=Homo sapiens GN=HSDL2 PE=1 SV=1 - [HSDL2_HUMAN] | 6 | 3 | 6 | 418 | 45.4 | 7.99 |
| P30419 | Glycylpeptide N-tetradecanoyltransferase 1 OS=Homo sapiens GN=NMT1 PE=1 SV=2 - [NMT1_HUMAN] | 6 | 6 | 5 | 496 | 56.8 | 7.80 |
| P49821 | NADH dehydrogenase [ubiquinone] flavoprotein 1. mitochondrial OS=Homo sapiens GN=NDUFV1 PE=1 SV=4 - [NDUV1_HUMAN] | 6 | 2 | 6 | 464 | 50.8 | 8.21 |
| P07954 | Fumarate hydratase. mitochondrial OS=Homo sapiens GN=FH PE=1 SV=3 - [FUMH_HUMAN] | 6 | 5 | 6 | 510 | 54.6 | 8.76 |
| Q9UKM9 | RNA-binding protein Raly OS=Homo sapiens GN=RALY PE=1 SV=1 - [RALY_HUMAN] | 6 | 4 | 5 | 306 | 32.4 | 9.17 |
| Q16881 | Thioredoxin reductase 1. cytoplasmic OS=Homo sapiens GN=TXNRD1 PE=1 SV=3 - [TRXR1_HUMAN] | 6 | 3 | 7 | 649 | 70.9 | 7.39 |
| Q15233 | Non-POU domain-containing octamer-binding protein OS=Homo sapiens GN=NONO PE=1 SV=4 - [NONO_HUMAN] | 6 | 12 | 12 | 471 | 54.2 | 8.95 |
| Q12874 | Splicing factor 3A subunit 3 OS=Homo sapiens GN=SF3A3 PE=1 SV=1 - [SF3A3_HUMAN] | 6 | 3 | 6 | 501 | 58.8 | 5.38 |
| Q96CS3 | FAS-associated factor 2 OS=Homo sapiens GN=FAF2 PE=1 SV=2 - [FAF2_HUMAN] | 6 | 6 | 5 | 445 | 52.6 | 5.62 |
| Q13409 | Cytoplasmic dynein 1 intermediate chain 2 OS=Homo sapiens GN=DYNC1I2 PE=1 SV=3 - [DC1I2_HUMAN] | 6 | 5 | 8 | 638 | 71.4 | 5.20 |
| Q92841 | Probable ATP-dependent RNA helicase DDX17 OS=Homo sapiens GN=DDX17 PE=1 SV=2 - [DDX17_HUMAN] | 6 | 8 | 10 | 729 | 80.2 | 8.27 |
| Q9Y285 | Phenylalanyl-tRNA synthetase alpha chain OS=Homo sapiens GN=FARSA PE=1 SV=3 - [SYFA_HUMAN] | 6 | 5 | 8 | 508 | 57.5 | 7.80 |
| Q8N684 | Cleavage and polyadenylation specificity factor subunit 7 OS=Homo sapiens GN=CPSF7 PE=1 SV=1 - [CPSF7_HUMAN] | 6 | 7 | 7 | 471 | 52.0 | 8.00 |
| P09110 | 3-ketoacyl-CoA thiolase. peroxisomal OS=Homo sapiens GN=ACAA1 PE=1 SV=2 - [THIK_HUMAN] | 6 | 4 | 5 | 424 | 44.3 | 8.44 |
| Q8WW52 | Protein FAM151A OS=Homo sapiens GN=FAM151A PE=2 SV=2 - [F151A_HUMAN] | 6 | 6 | 6 | 585 | 64.0 | 6.67 |
| Q9HDC9 | Adipocyte plasma membrane-associated protein OS=Homo sapiens GN=APMAP PE=1 SV=2 - [APMAP_HUMAN] | 6 | 7 | 4 | 416 | 46.5 | 6.16 |
| P27694 | Replication protein A 70 kDa DNA-binding subunit OS=Homo sapiens GN=RPA1 PE=1 SV=2 - [RFA1_HUMAN] | 6 | 3 | 6 | 616 | 68.1 | 7.21 |
| P36551 | Coproporphyrinogen-III oxidase. mitochondrial OS=Homo sapiens GN=CPOX PE=1 SV=3 - [HEM6_HUMAN] | 6 | 6 | 5 | 454 | 50.1 | 8.25 |
| P51648 | Fatty aldehyde dehydrogenase OS=Homo sapiens GN=ALDH3A2 PE=1 SV=1 - [AL3A2_HUMAN] | 6 | 3 | 6 | 485 | 54.8 | 7.88 |
| Q9Y6M1 | Insulin-like growth factor 2 mRNA-binding protein 2 OS=Homo sapiens GN=IGF2BP2 PE=1 SV=2 - [IF2B2_HUMAN] | 6 | 5 | 6 | 599 | 66.1 | 8.46 |
| P50552 | Vasodilator-stimulated phosphoprotein OS=Homo sapiens GN=VASP PE=1 SV=3 - [VASP_HUMAN] | 6 | 6 | 5 | 380 | 39.8 | 8.94 |
| P01009 | Alpha-1-antitrypsin OS=Homo sapiens GN=SERPINA1 PE=1 SV=3 - [A1AT_HUMAN] | 6 | 6 | 6 | 418 | 46.7 | 5.59 |
| P55084 | Trifunctional enzyme subunit beta. mitochondrial OS=Homo sapiens GN=HADHB PE=1 SV=3 - [ECHB_HUMAN] | 6 | 6 | 5 | 474 | 51.3 | 9.41 |
| P00966 | Argininosuccinate synthase OS=Homo sapiens GN=ASS1 PE=1 SV=2 - [ASSY_HUMAN] | 6 | 3 | 6 | 412 | 46.5 | 8.02 |
| Q15942 | Zyxin OS=Homo sapiens GN=ZYX PE=1 SV=1 - [ZYX_HUMAN] | 6 | 6 | 7 | 572 | 61.2 | 6.67 |
| P40763 | Signal transducer and activator of transcription 3 OS=Homo sapiens GN=STAT3 PE=1 SV=2 - [STAT3_HUMAN] | 6 | 6 | 5 | 770 | 88.0 | 6.30 |
| O95747 | Serine/threonine-protein kinase OSR1 OS=Homo sapiens GN=OXSR1 PE=1 SV=1 - [OXSR1_HUMAN] | 6 | 5 | 6 | 527 | 58.0 | 6.43 |
| P08621 | U1 small nuclear ribonucleoprotein 70 kDa OS=Homo sapiens GN=SNRNP70 PE=1 SV=2 - [RU17_HUMAN] | 6 | 5 | 6 | 437 | 51.5 | 9.94 |
| O00567 | Nucleolar protein 56 OS=Homo sapiens GN=NOP56 PE=1 SV=4 - [NOP56_HUMAN] | 6 | 4 | 6 | 594 | 66.0 | 9.19 |
| P50895 | Basal cell adhesion molecule OS=Homo sapiens GN=BCAM PE=1 SV=2 - [BCAM_HUMAN] | 6 | 4 | 6 | 628 | 67.4 | 5.81 |
| Q9UHX1 | Poly(U)-binding-splicing factor PUF60 OS=Homo sapiens GN=PUF60 PE=1 SV=1 - [PUF60_HUMAN] | 6 | 5 | 6 | 559 | 59.8 | 5.29 |
| Q08945 | FACT complex subunit SSRP1 OS=Homo sapiens GN=SSRP1 PE=1 SV=1 - [SSRP1_HUMAN] | 6 | 3 | 4 | 709 | 81.0 | 6.87 |
| Q13724 | Mannosyl-oligosaccharide glucosidase OS=Homo sapiens GN=MOGS PE=1 SV=5 - [MOGS_HUMAN] | 6 | 4 | 5 | 837 | 91.9 | 8.90 |
| Q9H845 | Acyl-CoA dehydrogenase family member 9. mitochondrial OS=Homo sapiens GN=ACAD9 PE=1 SV=1 - [ACAD9_HUMAN] | 6 | 4 | 5 | 621 | 68.7 | 7.96 |
| Q9Y2L1 | Exosome complex exonuclease RRP44 OS=Homo sapiens GN=DIS3 PE=1 SV=2 - [RRP44_HUMAN] | 6 | 4 | 4 | 958 | 108.9 | 7.14 |
| O94776 | Metastasis-associated protein MTA2 OS=Homo sapiens GN=MTA2 PE=1 SV=1 - [MTA2_HUMAN] | 6 | 5 | 6 | 668 | 75.0 | 9.66 |
| Q96T76 | MMS19 nucleotide excision repair protein homolog OS=Homo sapiens GN=MMS19 PE=1 SV=2 - [MMS19_HUMAN] | 6 | 7 | 7 | 1030 | 113.2 | 6.35 |
| Q9NVI7 | ATPase family AAA domain-containing protein 3A OS=Homo sapiens GN=ATAD3A PE=1 SV=2 - [ATD3A_HUMAN] | 6 | 4 | 6 | 634 | 71.3 | 8.98 |
| Q8NE71 | ATP-binding cassette sub-family F member 1 OS=Homo sapiens GN=ABCF1 PE=1 SV=2 - [ABCF1_HUMAN] | 6 | 1 | 5 | 845 | 95.9 | 6.80 |
| P46459 | Vesicle-fusing ATPase OS=Homo sapiens GN=NSF PE=1 SV=3 - [NSF_HUMAN] | 6 | 5 | 6 | 744 | 82.5 | 6.95 |
| P14735 | Insulin-degrading enzyme OS=Homo sapiens GN=IDE PE=1 SV=4 - [IDE_HUMAN] | 6 | 3 | 4 | 1019 | 117.9 | 6.61 |
| P11586 | C-1-tetrahydrofolate synthase. cytoplasmic OS=Homo sapiens GN=MTHFD1 PE=1 SV=3 - [C1TC_HUMAN] | 6 | 4 | 6 | 935 | 101.5 | 7.30 |
| P42285 | Superkiller viralicidic activity 2-like 2 OS=Homo sapiens GN=SKIV2L2 PE=1 SV=3 - [SK2L2_HUMAN] | 6 | 4 | 8 | 1042 | 117.7 | 6.52 |
| O43156 | Uncharacterized protein KIAA0406 OS=Homo sapiens GN=KIAA0406 PE=1 SV=3 - [K0406_HUMAN] | 6 | 4 | 7 | 1089 | 122.0 | 5.97 |
| Q14CX7 | N-terminal acetyltransferase B complex subunit MDM20 OS=Homo sapiens GN=MDM20 PE=1 SV=1 - [MDM20_HUMAN] | 6 | 3 | 6 | 972 | 112.2 | 6.64 |
| Q5H9R7 | Serine/threonine-protein phosphatase 6 regulatory subunit 3 OS=Homo sapiens GN=SAPS3 PE=1 SV=2 - [SAPS3_HUMAN] | 6 | 5 | 8 | 873 | 97.6 | 4.60 |
| Q9UHB6 | LIM domain and actin-binding protein 1 OS=Homo sapiens GN=LIMA1 PE=1 SV=1 - [LIMA1_HUMAN] | 6 | 6 | 5 | 759 | 85.2 | 6.84 |
| Q9Y4E8 | Ubiquitin carboxyl-terminal hydrolase 15 OS=Homo sapiens GN=USP15 PE=1 SV=3 - [UBP15_HUMAN] | 6 | 3 | 6 | 981 | 112.3 | 5.22 |
| O43592 | Exportin-T OS=Homo sapiens GN=XPOT PE=1 SV=2 - [XPOT_HUMAN] | 6 | 5 | 3 | 962 | 109.9 | 5.39 |
| Q9P2J5 | Leucyl-tRNA synthetase. cytoplasmic OS=Homo sapiens GN=LARS PE=1 SV=2 - [SYLC_HUMAN] | 6 | 4 | 5 | 1176 | 134.4 | 7.30 |
| Q9NZ08 | Endoplasmic reticulum aminopeptidase 1 OS=Homo sapiens GN=ERAP1 PE=1 SV=3 - [ERAP1_HUMAN] | 6 | 4 | 5 | 941 | 107.2 | 6.46 |
| Q7L014 | Probable ATP-dependent RNA helicase DDX46 OS=Homo sapiens GN=DDX46 PE=1 SV=2 - [DDX46_HUMAN] | 6 | 3 | 6 | 1031 | 117.3 | 9.29 |
| Q14157 | Ubiquitin-associated protein 2-like OS=Homo sapiens GN=UBAP2L PE=1 SV=2 - [UBP2L_HUMAN] | 6 | 4 | 5 | 1087 | 114.5 | 7.11 |
| Q9Y5B9 | FACT complex subunit SPT16 OS=Homo sapiens GN=SUPT16H PE=1 SV=1 - [SP16H_HUMAN] | 6 | 3 | 5 | 1047 | 119.8 | 5.66 |
| O75153 | Protein KIAA0664 OS=Homo sapiens GN=KIAA0664 PE=1 SV=2 - [K0664_HUMAN] | 6 | 3 | 5 | 1309 | 146.6 | 6.13 |
| Q9Y4W6 | AFG3-like protein 2 OS=Homo sapiens GN=AFG3L2 PE=1 SV=2 - [AFG32_HUMAN] | 6 | 4 | 7 | 797 | 88.5 | 8.66 |
| O43847 | Nardilysin OS=Homo sapiens OX=9606 GN=NRDC PE=1 SV=3 - [NRDC_HUMAN] | 6 | 4 | 3 | 1151 | 131.6 | 4.98 |
| Q13017 | Rho GTPase-activating protein 5 OS=Homo sapiens GN=ARHGAP5 PE=1 SV=2 - [RHG05_HUMAN] | 6 | 3 | 8 | 1502 | 172.4 | 6.62 |
| Q86XA9 | HEAT repeat-containing protein 5A OS=Homo sapiens GN=HEATR5A PE=1 SV=2 - [HTR5A_HUMAN] | 6 | 5 | 5 | 2040 | 221.9 | 6.58 |
| P07437 | Tubulin beta chain OS=Homo sapiens GN=TUBB PE=1 SV=2 - [TBB5_HUMAN] | 5 | 76 | 79 | 444 | 49.6 | 4.89 |
| P0CG48 | Polyubiquitin-C OS=Homo sapiens GN=UBC PE=1 SV=3 - [UBC_HUMAN] | 5 | 28 | 31 | 685 | 77.0 | 7.66 |
| O75964 | ATP synthase subunit g. mitochondrial OS=Homo sapiens GN=ATP5L PE=1 SV=3 - [ATP5L_HUMAN] | 5 | 4 | 5 | 103 | 11.4 | 9.64 |
| Q96A72 | Protein mago nashi homolog 2 OS=Homo sapiens GN=MAGOHB PE=1 SV=1 - [MGN2_HUMAN] | 5 | 5 | 5 | 148 | 17.3 | 6.39 |
| Q01469 | Fatty acid-binding protein. epidermal OS=Homo sapiens GN=FABP5 PE=1 SV=3 - [FABP5_HUMAN] | 5 | 3 | 5 | 135 | 15.2 | 7.01 |
| P62829 | 60S ribosomal protein L23 OS=Homo sapiens GN=RPL23 PE=1 SV=1 - [RL23_HUMAN] | 5 | 4 | 7 | 140 | 14.9 | 10.51 |
| P61086 | Ubiquitin-conjugating enzyme E2 K OS=Homo sapiens GN=UBE2K PE=1 SV=3 - [UBE2K_HUMAN] | 5 | 4 | 7 | 200 | 22.4 | 5.44 |
| O14579 | Coatomer subunit epsilon OS=Homo sapiens GN=COPE PE=1 SV=3 - [COPE_HUMAN] | 5 | 3 | 7 | 308 | 34.5 | 5.12 |
| P39687 | Acidic leucine-rich nuclear phosphoprotein 32 family member A OS=Homo sapiens GN=ANP32A PE=1 SV=1 - [AN32A_HUMAN] | 5 | 6 | 7 | 249 | 28.6 | 4.09 |
| Q9BY32 | Inosine triphosphate pyrophosphatase OS=Homo sapiens GN=ITPA PE=1 SV=2 - [ITPA_HUMAN] | 5 | 3 | 6 | 194 | 21.4 | 5.66 |
| Q16718 | NADH dehydrogenase [ubiquinone] 1 alpha subcomplex subunit 5 OS=Homo sapiens GN=NDUFA5 PE=1 SV=3 - [NDUA5_HUMAN] | 5 | 4 | 4 | 116 | 13.5 | 5.99 |
| P49720 | Proteasome subunit beta type-3 OS=Homo sapiens GN=PSMB3 PE=1 SV=2 - [PSB3_HUMAN] | 5 | 6 | 6 | 205 | 22.9 | 6.55 |
| Q07021 | Complement component 1 Q subcomponent-binding protein. mitochondrial OS=Homo sapiens GN=C1QBP PE=1 SV=1 - [C1QBP_HUMAN] | 5 | 5 | 4 | 282 | 31.3 | 4.84 |
| P59998 | Actin-related protein 2/3 complex subunit 4 OS=Homo sapiens GN=ARPC4 PE=1 SV=3 - [ARPC4_HUMAN] | 5 | 5 | 4 | 168 | 19.7 | 8.43 |
| Q15185 | Prostaglandin E synthase 3 OS=Homo sapiens GN=PTGES3 PE=1 SV=1 - [TEBP_HUMAN] | 5 | 6 | 6 | 160 | 18.7 | 4.54 |
| P46783 | 40S ribosomal protein S10 OS=Homo sapiens GN=RPS10 PE=1 SV=1 - [RS10_HUMAN] | 5 | 9 | 9 | 165 | 18.9 | 10.15 |
| P62873 | Guanine nucleotide-binding protein G(I)/G(S)/G(T) subunit beta-1 OS=Homo sapiens GN=GNB1 PE=1 SV=3 - [GBB1_HUMAN] | 5 | 7 | 8 | 340 | 37.4 | 6.00 |
| Q9NQG5 | Regulation of nuclear pre-mRNA domain-containing protein 1B OS=Homo sapiens GN=RPRD1B PE=1 SV=1 - [RPR1B_HUMAN] | 5 | 4 | 4 | 326 | 36.9 | 5.97 |
| P30626 | Sorcin OS=Homo sapiens GN=SRI PE=1 SV=1 - [SORCN_HUMAN] | 5 | 4 | 4 | 198 | 21.7 | 5.59 |
| P62750 | 60S ribosomal protein L23a OS=Homo sapiens GN=RPL23A PE=1 SV=1 - [RL23A_HUMAN] | 5 | 4 | 7 | 156 | 17.7 | 10.45 |
| P61923 | Coatomer subunit zeta-1 OS=Homo sapiens GN=COPZ1 PE=1 SV=1 - [COPZ1_HUMAN] | 5 | 5 | 5 | 177 | 20.2 | 4.81 |
| P52907 | F-actin-capping protein subunit alpha-1 OS=Homo sapiens GN=CAPZA1 PE=1 SV=3 - [CAZA1_HUMAN] | 5 | 6 | 6 | 286 | 32.9 | 5.69 |
| Q15056 | Eukaryotic translation initiation factor 4H OS=Homo sapiens GN=EIF4H PE=1 SV=5 - [IF4H_HUMAN] | 5 | 5 | 4 | 248 | 27.4 | 7.23 |
| Q9H488 | GDP-fucose protein O-fucosyltransferase 1 OS=Homo sapiens GN=POFUT1 PE=1 SV=1 - [OFUT1_HUMAN] | 5 | 5 | 5 | 388 | 43.9 | 8.53 |
| P06132 | Uroporphyrinogen decarboxylase OS=Homo sapiens GN=UROD PE=1 SV=2 - [DCUP_HUMAN] | 5 | 4 | 5 | 367 | 40.8 | 6.14 |
| P52788 | Spermine synthase OS=Homo sapiens GN=SMS PE=1 SV=2 - [SPSY_HUMAN] | 5 | 2 | 3 | 366 | 41.2 | 5.02 |
| Q13155 | Aminoacyl tRNA synthetase complex-interacting multifunctional protein 2 OS=Homo sapiens GN=AIMP2 PE=1 SV=2 - [AIMP2_HUMAN] | 5 | 6 | 4 | 320 | 35.3 | 8.22 |
| O15144 | Actin-related protein 2/3 complex subunit 2 OS=Homo sapiens GN=ARPC2 PE=1 SV=1 - [ARPC2_HUMAN] | 5 | 4 | 5 | 300 | 34.3 | 7.36 |
| Q9BV20 | Methylthioribose-1-phosphate isomerase OS=Homo sapiens GN=MRI1 PE=1 SV=1 - [MTNA_HUMAN] | 5 | 4 | 4 | 369 | 39.1 | 6.30 |
| P09429 | High mobility group protein B1 OS=Homo sapiens GN=HMGB1 PE=1 SV=3 - [HMGB1_HUMAN] | 5 | 5 | 6 | 215 | 24.9 | 5.74 |
| P61619 | Protein transport protein Sec61 subunit alpha isoform 1 OS=Homo sapiens GN=SEC61A1 PE=1 SV=2 - [S61A1_HUMAN] | 5 | 10 | 9 | 476 | 52.2 | 8.06 |
| P25788 | Proteasome subunit alpha type-3 OS=Homo sapiens GN=PSMA3 PE=1 SV=2 - [PSA3_HUMAN] | 5 | 3 | 5 | 255 | 28.4 | 5.33 |
| Q7L1Q6 | Basic leucine zipper and W2 domain-containing protein 1 OS=Homo sapiens GN=BZW1 PE=1 SV=1 - [BZW1_HUMAN] | 5 | 3 | 7 | 419 | 48.0 | 5.92 |
| P06753 | Tropomyosin alpha-3 chain OS=Homo sapiens GN=TPM3 PE=1 SV=2 - [TPM3_HUMAN] | 5 | 10 | 11 | 285 | 32.9 | 4.72 |
| Q96HY6 | DDRGK domain-containing protein 1 OS=Homo sapiens GN=DDRGK1 PE=1 SV=2 - [DDRGK_HUMAN] | 5 | 6 | 7 | 314 | 35.6 | 5.12 |
| Q9UL25 | Ras-related protein Rab-21 OS=Homo sapiens GN=RAB21 PE=1 SV=3 - [RAB21_HUMAN] | 5 | 3 | 4 | 225 | 24.3 | 7.94 |
| Q9Y295 | Developmentally-regulated GTP-binding protein 1 OS=Homo sapiens GN=DRG1 PE=1 SV=1 - [DRG1_HUMAN] | 5 | 5 | 6 | 367 | 40.5 | 8.90 |
| P62906 | 60S ribosomal protein L10a OS=Homo sapiens GN=RPL10A PE=1 SV=2 - [RL10A_HUMAN] | 5 | 4 | 6 | 217 | 24.8 | 9.94 |
| Q6FI81 | Anamorsin OS=Homo sapiens GN=CIAPIN1 PE=1 SV=2 - [CPIN1_HUMAN] | 5 | 4 | 4 | 312 | 33.6 | 5.62 |
| P62917 | 60S ribosomal protein L8 OS=Homo sapiens GN=RPL8 PE=1 SV=2 - [RL8_HUMAN] | 5 | 3 | 6 | 257 | 28.0 | 11.03 |
| Q9UKK9 | ADP-sugar pyrophosphatase OS=Homo sapiens GN=NUDT5 PE=1 SV=1 - [NUDT5_HUMAN] | 5 | 3 | 4 | 219 | 24.3 | 4.94 |
| P31153 | S-adenosylmethionine synthetase isoform type-2 OS=Homo sapiens GN=MAT2A PE=1 SV=1 - [METK2_HUMAN] | 5 | 2 | 5 | 395 | 43.6 | 6.48 |
| O43852 | Calumenin OS=Homo sapiens GN=CALU PE=1 SV=2 - [CALU_HUMAN] | 5 | 4 | 4 | 315 | 37.1 | 4.64 |
| P20042 | Eukaryotic translation initiation factor 2 subunit 2 OS=Homo sapiens GN=EIF2S2 PE=1 SV=2 - [IF2B_HUMAN] | 5 | 3 | 5 | 333 | 38.4 | 5.80 |
| P13746 | HLA class I histocompatibility antigen. A-11 alpha chain OS=Homo sapiens GN=HLA-A PE=1 SV=1 - [1A11_HUMAN] | 5 | 4 | 4 | 365 | 40.9 | 6.15 |
| Q00796 | Sorbitol dehydrogenase OS=Homo sapiens GN=SORD PE=1 SV=4 - [DHSO_HUMAN] | 5 | 3 | 4 | 357 | 38.3 | 7.97 |
| P62191 | 26S protease regulatory subunit 4 OS=Homo sapiens GN=PSMC1 PE=1 SV=1 - [PRS4_HUMAN] | 5 | 5 | 8 | 440 | 49.2 | 6.21 |
| P46777 | 60S ribosomal protein L5 OS=Homo sapiens GN=RPL5 PE=1 SV=3 - [RL5_HUMAN] | 5 | 6 | 5 | 297 | 34.3 | 9.72 |
| Q9UBE0 | SUMO-activating enzyme subunit 1 OS=Homo sapiens GN=SAE1 PE=1 SV=1 - [SAE1_HUMAN] | 5 | 6 | 5 | 346 | 38.4 | 5.30 |
| P60891 | Ribose-phosphate pyrophosphokinase 1 OS=Homo sapiens GN=PRPS1 PE=1 SV=2 - [PRPS1_HUMAN] | 5 | 6 | 9 | 318 | 34.8 | 6.98 |
| O00170 | AH receptor-interacting protein OS=Homo sapiens GN=AIP PE=1 SV=2 - [AIP_HUMAN] | 5 | 4 | 5 | 330 | 37.6 | 6.29 |
| P31930 | Cytochrome b-c1 complex subunit 1. mitochondrial OS=Homo sapiens GN=UQCRC1 PE=1 SV=3 - [QCR1_HUMAN] | 5 | 5 | 4 | 480 | 52.6 | 6.37 |
| P42126 | 3.2-trans-enoyl-CoA isomerase. mitochondrial OS=Homo sapiens GN=DCI PE=1 SV=1 - [D3D2_HUMAN] | 5 | 5 | 4 | 302 | 32.8 | 8.54 |
| Q8NBX0 | Probable saccharopine dehydrogenase OS=Homo sapiens GN=SCCPDH PE=1 SV=1 - [SCPDH_HUMAN] | 5 | 3 | 4 | 429 | 47.1 | 9.14 |
| Q9Y2Z0 | Suppressor of G2 allele of SKP1 homolog OS=Homo sapiens GN=SUGT1 PE=1 SV=3 - [SUGT1_HUMAN] | 5 | 4 | 5 | 365 | 41.0 | 5.16 |
| O43765 | Small glutamine-rich tetratricopeptide repeat-containing protein alpha OS=Homo sapiens GN=SGTA PE=1 SV=1 - [SGTA_HUMAN] | 5 | 5 | 7 | 313 | 34.0 | 4.87 |
| P48556 | 26S proteasome non-ATPase regulatory subunit 8 OS=Homo sapiens GN=PSMD8 PE=1 SV=2 - [PSMD8_HUMAN] | 5 | 5 | 5 | 350 | 39.6 | 9.70 |
| Q16795 | NADH dehydrogenase [ubiquinone] 1 alpha subcomplex subunit 9. mitochondrial OS=Homo sapiens GN=NDUFA9 PE=1 SV=2 - [NDUA9_HUMAN] | 5 | 4 | 3 | 377 | 42.5 | 9.80 |
| O96008 | Mitochondrial import receptor subunit TOM40 homolog OS=Homo sapiens GN=TOMM40 PE=1 SV=1 - [TOM40_HUMAN] | 5 | 7 | 7 | 361 | 37.9 | 7.25 |
| Q96I99 | Succinyl-CoA ligase [GDP-forming] subunit beta. mitochondrial OS=Homo sapiens GN=SUCLG2 PE=1 SV=2 - [SUCB2_HUMAN] | 5 | 5 | 4 | 432 | 46.5 | 6.39 |
| P50440 | Glycine amidinotransferase. mitochondrial OS=Homo sapiens GN=GATM PE=1 SV=1 - [GATM_HUMAN] | 5 | 1 | 7 | 423 | 48.4 | 8.05 |
| P61289 | Proteasome activator complex subunit 3 OS=Homo sapiens GN=PSME3 PE=1 SV=1 - [PSME3_HUMAN] | 5 | 6 | 2 | 254 | 29.5 | 5.95 |
| P55209 | Nucleosome assembly protein 1-like 1 OS=Homo sapiens GN=NAP1L1 PE=1 SV=1 - [NP1L1_HUMAN] | 5 | 4 | 6 | 391 | 45.3 | 4.46 |
| Q86U38 | Pumilio domain-containing protein C14orf21 OS=Homo sapiens GN=C14orf21 PE=1 SV=1 - [CN021_HUMAN] | 5 | 3 | 3 | 636 | 69.4 | 7.28 |
| Q13148 | TAR DNA-binding protein 43 OS=Homo sapiens GN=TARDBP PE=1 SV=1 - [TADBP_HUMAN] | 5 | 6 | 4 | 414 | 44.7 | 6.19 |
| O43684 | Mitotic checkpoint protein BUB3 OS=Homo sapiens GN=BUB3 PE=1 SV=1 - [BUB3_HUMAN] | 5 | 3 | 5 | 328 | 37.1 | 6.84 |
| Q8N2K0 | Monoacylglycerol lipase ABHD12 OS=Homo sapiens GN=ABHD12 PE=2 SV=2 - [ABD12_HUMAN] | 5 | 4 | 3 | 398 | 45.1 | 8.65 |
| Q13247 | Splicing factor. arginine/serine-rich 6 OS=Homo sapiens GN=SFRS6 PE=1 SV=2 - [SFRS6_HUMAN] | 5 | 6 | 7 | 344 | 39.6 | 11.43 |
| P16930 | Fumarylacetoacetase OS=Homo sapiens GN=FAH PE=1 SV=2 - [FAAA_HUMAN] | 5 | 5 | 5 | 419 | 46.3 | 6.95 |
| P63010 | AP-2 complex subunit beta OS=Homo sapiens GN=AP2B1 PE=1 SV=1 - [AP2B1_HUMAN] | 5 | 14 | 13 | 937 | 104.5 | 5.38 |
| Q13363 | C-terminal-binding protein 1 OS=Homo sapiens GN=CTBP1 PE=1 SV=2 - [CTBP1_HUMAN] | 5 | 4 | 2 | 440 | 47.5 | 6.77 |
| P35637 | RNA-binding protein FUS OS=Homo sapiens GN=FUS PE=1 SV=1 - [FUS_HUMAN] | 5 | 4 | 3 | 526 | 53.4 | 9.36 |
| A1L0T0 | Acetolactate synthase-like protein OS=Homo sapiens GN=ILVBL PE=1 SV=2 - [ILVBL_HUMAN] | 5 | 4 | 3 | 632 | 67.8 | 8.15 |
| Q8IWE2 | Protein NOXP20 OS=Homo sapiens GN=FAM114A1 PE=1 SV=2 - [NXP20_HUMAN] | 5 | 4 | 6 | 563 | 60.7 | 4.68 |
| Q9HCC0 | Methylcrotonoyl-CoA carboxylase beta chain. mitochondrial OS=Homo sapiens GN=MCCC2 PE=1 SV=1 - [MCCB_HUMAN] | 5 | 4 | 3 | 563 | 61.3 | 7.68 |
| Q15813 | Tubulin-specific chaperone E OS=Homo sapiens GN=TBCE PE=1 SV=1 - [TBCE_HUMAN] | 5 | 5 | 4 | 527 | 59.3 | 6.76 |
| Q14847 | LIM and SH3 domain protein 1 OS=Homo sapiens GN=LASP1 PE=1 SV=2 - [LASP1_HUMAN] | 5 | 6 | 7 | 261 | 29.7 | 7.05 |
| P49257 | Protein ERGIC-53 OS=Homo sapiens GN=LMAN1 PE=1 SV=2 - [LMAN1_HUMAN] | 5 | 4 | 4 | 510 | 57.5 | 6.77 |
| Q96HE7 | ERO1-like protein alpha OS=Homo sapiens GN=ERO1L PE=1 SV=2 - [ERO1A_HUMAN] | 5 | 3 | 4 | 468 | 54.4 | 5.68 |
| O75439 | Mitochondrial-processing peptidase subunit beta OS=Homo sapiens GN=PMPCB PE=1 SV=2 - [MPPB_HUMAN] | 5 | 3 | 5 | 489 | 54.3 | 6.83 |
| P01019 | Angiotensinogen OS=Homo sapiens GN=AGT PE=1 SV=1 - [ANGT_HUMAN] | 5 | 4 | 8 | 485 | 53.1 | 6.32 |
| Q96I24 | Far upstream element-binding protein 3 OS=Homo sapiens GN=FUBP3 PE=1 SV=2 - [FUBP3_HUMAN] | 5 | 6 | 6 | 572 | 61.6 | 8.38 |
| Q02252 | Methylmalonate-semialdehyde dehydrogenase [acylating]. mitochondrial OS=Homo sapiens GN=ALDH6A1 PE=1 SV=2 - [MMSA_HUMAN] | 5 |  | 5 | 535 | 57.8 | 8.50 |
| Q9Y376 | Calcium-binding protein 39 OS=Homo sapiens GN=CAB39 PE=1 SV=1 - [CAB39_HUMAN] | 5 | 4 | 5 | 341 | 39.8 | 6.89 |
| Q16630 | Cleavage and polyadenylation specificity factor subunit 6 OS=Homo sapiens GN=CPSF6 PE=1 SV=2 - [CPSF6_HUMAN] | 5 | 6 | 7 | 551 | 59.2 | 7.15 |
| Q10713 | Mitochondrial-processing peptidase subunit alpha OS=Homo sapiens GN=PMPCA PE=1 SV=2 - [MPPA_HUMAN] | 5 | 7 | 7 | 525 | 58.2 | 6.92 |
| Q96TC7 | Regulator of microtubule dynamics protein 3 OS=Homo sapiens GN=FAM82A2 PE=1 SV=2 - [RMD3_HUMAN] | 5 | 5 | 4 | 470 | 52.1 | 5.10 |
| O95864 | Fatty acid desaturase 2 OS=Homo sapiens GN=FADS2 PE=1 SV=1 - [FADS2_HUMAN] | 5 | 4 | 3 | 444 | 52.2 | 8.82 |
| O94973 | AP-2 complex subunit alpha-2 OS=Homo sapiens GN=AP2A2 PE=1 SV=2 - [AP2A2_HUMAN] | 5 | 4 | 6 | 939 | 103.9 | 6.96 |
| Q9H3P7 | Golgi resident protein GCP60 OS=Homo sapiens GN=ACBD3 PE=1 SV=4 - [GCP60_HUMAN] | 5 | 2 | 5 | 528 | 60.6 | 5.06 |
| Q8WXF1 | Paraspeckle component 1 OS=Homo sapiens GN=PSPC1 PE=1 SV=1 - [PSPC1_HUMAN] | 5 | 5 | 6 | 523 | 58.7 | 6.67 |
| O15371 | Eukaryotic translation initiation factor 3 subunit D OS=Homo sapiens GN=EIF3D PE=1 SV=1 - [EIF3D_HUMAN] | 5 | 5 | 6 | 548 | 63.9 | 6.05 |
| Q9UNF1 | Melanoma-associated antigen D2 OS=Homo sapiens GN=MAGED2 PE=1 SV=2 - [MAGD2_HUMAN] | 5 | 2 | 5 | 606 | 64.9 | 9.32 |
| P49419 | Alpha-aminoadipic semialdehyde dehydrogenase OS=Homo sapiens GN=ALDH7A1 PE=1 SV=5 - [AL7A1_HUMAN] | 5 | 2 | 4 | 539 | 58.5 | 7.99 |
| Q9UBM7 | 7-dehydrocholesterol reductase OS=Homo sapiens GN=DHCR7 PE=1 SV=1 - [DHCR7_HUMAN] | 5 | 3 | 6 | 475 | 54.5 | 8.70 |
| O95573 | Long-chain-fatty-acid--CoA ligase 3 OS=Homo sapiens GN=ACSL3 PE=1 SV=3 - [ACSL3_HUMAN] | 5 | 6 | 6 | 720 | 80.4 | 8.38 |
| P23368 | NAD-dependent malic enzyme. mitochondrial OS=Homo sapiens GN=ME2 PE=1 SV=1 - [MAOM_HUMAN] | 5 | 3 | 4 | 584 | 65.4 | 7.61 |
| O76021 | Ribosomal L1 domain-containing protein 1 OS=Homo sapiens GN=RSL1D1 PE=1 SV=3 - [RL1D1_HUMAN] | 5 | 1 | 5 | 490 | 54.9 | 10.13 |
| P14923 | Junction plakoglobin OS=Homo sapiens GN=JUP PE=1 SV=3 - [PLAK_HUMAN] | 5 | 8 | 8 | 745 | 81.7 | 6.14 |
| Q9UBT2 | SUMO-activating enzyme subunit 2 OS=Homo sapiens GN=UBA2 PE=1 SV=2 - [SAE2_HUMAN] | 5 | 4 | 3 | 640 | 71.2 | 5.29 |
| P49902 | Cytosolic purine 5'-nucleotidase OS=Homo sapiens GN=NT5C2 PE=1 SV=1 - [5NTC_HUMAN] | 5 | 4 | 4 | 561 | 64.9 | 6.14 |
| Q08AM6 | Protein VAC14 homolog OS=Homo sapiens GN=VAC14 PE=1 SV=1 - [VAC14_HUMAN] | 5 | 4 | 4 | 782 | 87.9 | 6.13 |
| P34896 | Serine hydroxymethyltransferase. cytosolic OS=Homo sapiens GN=SHMT1 PE=1 SV=1 - [GLYC_HUMAN] | 5 | 4 | 4 | 483 | 53.0 | 7.71 |
| Q8N392 | Rho GTPase-activating protein 18 OS=Homo sapiens GN=ARHGAP18 PE=1 SV=3 - [RHG18_HUMAN] | 5 | 3 | 4 | 663 | 74.9 | 6.44 |
| O75821 | Eukaryotic translation initiation factor 3 subunit G OS=Homo sapiens GN=EIF3G PE=1 SV=2 - [EIF3G_HUMAN] | 5 | 5 | 5 | 320 | 35.6 | 6.13 |
| Q9UHB9 | Signal recognition particle 68 kDa protein OS=Homo sapiens GN=SRP68 PE=1 SV=2 - [SRP68_HUMAN] | 5 | 3 | 4 | 627 | 70.7 | 8.56 |
| P26368 | Splicing factor U2AF 65 kDa subunit OS=Homo sapiens GN=U2AF2 PE=1 SV=4 - [U2AF2_HUMAN] | 5 | 7 | 6 | 475 | 53.5 | 9.09 |
| Q16643 | Drebrin OS=Homo sapiens GN=DBN1 PE=1 SV=4 - [DREB_HUMAN] | 5 | 3 | 4 | 649 | 71.4 | 4.45 |
| O94925 | Glutaminase kidney isoform. mitochondrial OS=Homo sapiens GN=GLS PE=1 SV=1 - [GLSK_HUMAN] | 5 | 4 | 4 | 669 | 73.4 | 7.77 |
| Q9ULC5 | Long-chain-fatty-acid--CoA ligase 5 OS=Homo sapiens GN=ACSL5 PE=1 SV=1 - [ACSL5_HUMAN] | 5 | 4 | 6 | 683 | 75.9 | 6.92 |
| Q13557 | Calcium/calmodulin-dependent protein kinase type II subunit delta OS=Homo sapiens GN=CAMK2D PE=1 SV=3 - [KCC2D_HUMAN] | 5 | 3 | 4 | 499 | 56.3 | 7.25 |
| P28331 | NADH-ubiquinone oxidoreductase 75 kDa subunit. mitochondrial OS=Homo sapiens GN=NDUFS1 PE=1 SV=3 - [NDUS1_HUMAN] | 5 | 4 | 3 | 727 | 79.4 | 6.23 |
| P11216 | Glycogen phosphorylase. brain form OS=Homo sapiens GN=PYGB PE=1 SV=5 - [PYGB_HUMAN] | 5 | 6 | 6 | 843 | 96.6 | 6.86 |
| Q96JB5 | CDK5 regulatory subunit-associated protein 3 OS=Homo sapiens GN=CDK5RAP3 PE=1 SV=2 - [CK5P3_HUMAN] | 5 | 4 | 5 | 506 | 56.9 | 4.75 |
| Q14258 | E3 ubiquitin/ISG15 ligase TRIM25 OS=Homo sapiens GN=TRIM25 PE=1 SV=2 - [TRI25_HUMAN] | 5 | 3 | 6 | 630 | 70.9 | 8.09 |
| Q13618 | Cullin-3 OS=Homo sapiens GN=CUL3 PE=1 SV=2 - [CUL3_HUMAN] | 5 | 3 | 5 | 768 | 88.9 | 8.48 |
| Q9UEY8 | Gamma-adducin OS=Homo sapiens GN=ADD3 PE=1 SV=1 - [ADDG_HUMAN] | 5 | 3 | 5 | 706 | 79.1 | 6.32 |
| Q09161 | Nuclear cap-binding protein subunit 1 OS=Homo sapiens GN=NCBP1 PE=1 SV=1 - [NCBP1_HUMAN] | 5 | 5 | 5 | 790 | 91.8 | 6.43 |
| O43795 | Myosin-Ib OS=Homo sapiens GN=MYO1B PE=1 SV=3 - [MYO1B_HUMAN] | 5 | 3 | 7 | 1136 | 131.9 | 9.38 |
| P52789 | Hexokinase-2 OS=Homo sapiens GN=HK2 PE=1 SV=2 - [HXK2_HUMAN] | 5 | 5 | 2 | 917 | 102.3 | 6.05 |
| P06396 | Gelsolin OS=Homo sapiens GN=GSN PE=1 SV=1 - [GELS_HUMAN] | 5 | 3 | 4 | 782 | 85.6 | 6.28 |
| Q92667 | A kinase anchor protein 1. mitochondrial OS=Homo sapiens GN=AKAP1 PE=1 SV=1 - [AKAP1_HUMAN] | 5 | 4 | 5 | 903 | 97.3 | 4.94 |
| Q8N1F7 | Nuclear pore complex protein Nup93 OS=Homo sapiens GN=NUP93 PE=1 SV=2 - [NUP93_HUMAN] | 5 | 4 | 3 | 819 | 93.4 | 5.72 |
| Q9UQE7 | Structural maintenance of chromosomes protein 3 OS=Homo sapiens GN=SMC3 PE=1 SV=2 - [SMC3_HUMAN] | 5 | 3 | 4 | 1217 | 141.5 | 7.18 |
| O00203 | AP-3 complex subunit beta-1 OS=Homo sapiens GN=AP3B1 PE=1 SV=3 - [AP3B1_HUMAN] | 5 | 5 | 6 | 1094 | 121.2 | 6.04 |
| Q9BPX3 | Condensin complex subunit 3 OS=Homo sapiens GN=NCAPG PE=1 SV=1 - [CND3_HUMAN] | 5 | 1 | 4 | 1015 | 114.3 | 5.59 |
| Q9UBC2 | Epidermal growth factor receptor substrate 15-like 1 OS=Homo sapiens GN=EPS15L1 PE=1 SV=1 - [EP15R_HUMAN] | 5 | 3 | 4 | 864 | 94.2 | 5.11 |
| Q92797 | Symplekin OS=Homo sapiens GN=SYMPK PE=1 SV=2 - [SYMPK_HUMAN] | 5 | 2 | 4 | 1274 | 141.1 | 6.13 |
| O14976 | Cyclin-G-associated kinase OS=Homo sapiens GN=GAK PE=1 SV=2 - [GAK_HUMAN] | 5 | 3 | 4 | 1311 | 143.1 | 5.73 |
| P23229 | Integrin alpha-6 OS=Homo sapiens GN=ITGA6 PE=1 SV=5 - [ITA6_HUMAN] | 5 | 3 | 5 | 1130 | 126.5 | 6.61 |
| P35579 | Myosin-9 OS=Homo sapiens GN=MYH9 PE=1 SV=4 - [MYH9_HUMAN] | 5 | 7 | 1 | 1960 | 226.4 | 5.60 |
| Q9NVI1 | Fanconi anemia group I protein OS=Homo sapiens GN=FANCI PE=1 SV=4 - [FANCI_HUMAN] | 5 | 6 | 5 | 1328 | 149.2 | 6.74 |
| P01024 | Complement C3 OS=Homo sapiens GN=C3 PE=1 SV=2 - [CO3_HUMAN] | 5 | 5 | 5 | 1663 | 187.0 | 6.40 |
| Q86UP2 | Kinectin OS=Homo sapiens GN=KTN1 PE=1 SV=1 - [KTN1_HUMAN] | 5 | 1 | 5 | 1357 | 156.2 | 5.64 |
| P07942 | Laminin subunit beta-1 OS=Homo sapiens GN=LAMB1 PE=1 SV=2 - [LAMB1_HUMAN] | 5 | 2 | 3 | 1786 | 197.9 | 4.94 |
| Q92538 | Golgi-specific brefeldin A-resistance guanine nucleotide exchange factor 1 OS=Homo sapiens GN=GBF1 PE=1 SV=2 - [GBF1_HUMAN] | 5 | 2 | 4 | 1859 | 206.3 | 5.73 |
| Q92896 | Golgi apparatus protein 1 OS=Homo sapiens GN=GLG1 PE=1 SV=2 - [GSLG1_HUMAN] | 5 | 4 | 4 | 1179 | 134.5 | 6.90 |
| Q8WWI1 | LIM domain only protein 7 OS=Homo sapiens GN=LMO7 PE=1 SV=3 - [LMO7_HUMAN] | 5 | 3 | 5 | 1683 | 192.6 | 8.09 |
| Q14997 | Proteasome activator complex subunit 4 OS=Homo sapiens GN=PSME4 PE=1 SV=2 - [PSME4_HUMAN] | 5 | 4 | 3 | 1843 | 211.2 | 6.90 |
| Q14839 | Chromodomain-helicase-DNA-binding protein 4 OS=Homo sapiens GN=CHD4 PE=1 SV=2 - [CHD4_HUMAN] | 5 | 4 | 3 | 1912 | 217.9 | 5.86 |
| P10586 | Receptor-type tyrosine-protein phosphatase F OS=Homo sapiens GN=PTPRF PE=1 SV=2 - [PTPRF_HUMAN] | 5 | 2 | 4 | 1907 | 212.7 | 6.30 |
| P05387 | 60S acidic ribosomal protein P2 OS=Homo sapiens GN=RPLP2 PE=1 SV=1 - [RLA2_HUMAN] | 4 | 5 | 3 | 115 | 11.7 | 4.54 |
| P68363 | Tubulin alpha-1B chain OS=Homo sapiens GN=TUBA1B PE=1 SV=1 - [TBA1B_HUMAN] | 4 | 77 | 81 | 451 | 50.1 | 5.06 |
| P15531 | Nucleoside diphosphate kinase A OS=Homo sapiens GN=NME1 PE=1 SV=1 - [NDKA_HUMAN] | 4 | 10 | 15 | 152 | 17.1 | 6.19 |
| P62888 | 60S ribosomal protein L30 OS=Homo sapiens GN=RPL30 PE=1 SV=2 - [RL30_HUMAN] | 4 | 4 | 5 | 115 | 12.8 | 9.63 |
| Q9NRX4 | 14 kDa phosphohistidine phosphatase OS=Homo sapiens GN=PHPT1 PE=1 SV=1 - [PHP14_HUMAN] | 4 | 7 | 7 | 125 | 13.8 | 6.07 |
| P49773 | Histidine triad nucleotide-binding protein 1 OS=Homo sapiens GN=HINT1 PE=1 SV=2 - [HINT1_HUMAN] | 4 | 1 | 4 | 126 | 13.8 | 6.95 |
| O95336 | 6-phosphogluconolactonase OS=Homo sapiens GN=PGLS PE=1 SV=2 - [6PGL_HUMAN] | 4 | 2 | 4 | 258 | 27.5 | 6.05 |
| P62244 | 40S ribosomal protein S15a OS=Homo sapiens GN=RPS15A PE=1 SV=2 - [RS15A_HUMAN] | 4 | 2 | 4 | 130 | 14.8 | 10.13 |
| P00167 | Cytochrome b5 OS=Homo sapiens GN=CYB5A PE=1 SV=2 - [CYB5_HUMAN] | 4 | 3 | 3 | 134 | 15.3 | 4.96 |
| Q9BRA2 | Thioredoxin domain-containing protein 17 OS=Homo sapiens GN=TXNDC17 PE=1 SV=1 - [TXD17_HUMAN] | 4 | 4 | 4 | 123 | 13.9 | 5.52 |
| P62314 | Small nuclear ribonucleoprotein Sm D1 OS=Homo sapiens GN=SNRPD1 PE=1 SV=1 - [SMD1_HUMAN] | 4 | 8 | 9 | 119 | 13.3 | 11.56 |
| P61106 | Ras-related protein Rab-14 OS=Homo sapiens GN=RAB14 PE=1 SV=4 - [RAB14_HUMAN] | 4 | 7 | 7 | 215 | 23.9 | 6.21 |
| Q13185 | Chromobox protein homolog 3 OS=Homo sapiens GN=CBX3 PE=1 SV=4 - [CBX3_HUMAN] | 4 | 3 | 6 | 183 | 20.8 | 5.33 |
| P52565 | Rho GDP-dissociation inhibitor 1 OS=Homo sapiens GN=ARHGDIA PE=1 SV=3 - [GDIR1_HUMAN] | 4 | 6 | 6 | 204 | 23.2 | 5.11 |
| P61803 | Dolichyl-diphosphooligosaccharide--protein glycosyltransferase subunit DAD1 OS=Homo sapiens GN=DAD1 PE=1 SV=3 - [DAD1_HUMAN] | 4 | 3 | 3 | 113 | 12.5 | 7.08 |
| P60660 | Myosin light polypeptide 6 OS=Homo sapiens GN=MYL6 PE=1 SV=2 - [MYL6_HUMAN] | 4 | 4 | 4 | 151 | 16.9 | 4.65 |
| P62266 | 40S ribosomal protein S23 OS=Homo sapiens GN=RPS23 PE=1 SV=3 - [RS23_HUMAN] | 4 | 4 | 4 | 143 | 15.8 | 10.49 |
| P05141 | ADP/ATP translocase 2 OS=Homo sapiens GN=SLC25A5 PE=1 SV=7 - [ADT2_HUMAN] | 4 | 12 | 13 | 298 | 32.8 | 9.69 |
| P40616 | ADP-ribosylation factor-like protein 1 OS=Homo sapiens GN=ARL1 PE=1 SV=1 - [ARL1_HUMAN] | 4 | 5 | 6 | 181 | 20.4 | 5.72 |
| P28482 | Mitogen-activated protein kinase 1 OS=Homo sapiens GN=MAPK1 PE=1 SV=3 - [MK01_HUMAN] | 4 | 11 | 7 | 360 | 41.4 | 6.98 |
| Q02539 | Histone H1.1 OS=Homo sapiens GN=HIST1H1A PE=1 SV=3 - [H11_HUMAN] | 4 | 5 | 11 | 215 | 21.8 | 10.99 |
| O60869 | Endothelial differentiation-related factor 1 OS=Homo sapiens GN=EDF1 PE=1 SV=1 - [EDF1_HUMAN] | 4 | 1 | 4 | 148 | 16.4 | 9.95 |
| Q9HB71 | Calcyclin-binding protein OS=Homo sapiens GN=CACYBP PE=1 SV=2 - [CYBP_HUMAN] | 4 | 5 | 5 | 228 | 26.2 | 8.25 |
| Q09028 | Histone-binding protein RBBP4 OS=Homo sapiens GN=RBBP4 PE=1 SV=3 - [RBBP4_HUMAN] | 4 | 5 | 8 | 425 | 47.6 | 4.89 |
| Q13838 | Spliceosome RNA helicase BAT1 OS=Homo sapiens GN=BAT1 PE=1 SV=1 - [UAP56_HUMAN] | 4 | 14 | 15 | 428 | 49.0 | 5.67 |
| Q15631 | Translin OS=Homo sapiens GN=TSN PE=1 SV=1 - [TSN_HUMAN] | 4 | 7 | 5 | 228 | 26.2 | 6.44 |
| P28072 | Proteasome subunit beta type-6 OS=Homo sapiens GN=PSMB6 PE=1 SV=4 - [PSB6_HUMAN] | 4 | 4 | 4 | 239 | 25.3 | 4.92 |
| P31947 | 14-3-3 protein sigma OS=Homo sapiens GN=SFN PE=1 SV=1 - [1433S_HUMAN] | 4 | 9 | 8 | 248 | 27.8 | 4.74 |
| Q9Y3D6 | Mitochondrial fission 1 protein OS=Homo sapiens GN=FIS1 PE=1 SV=2 - [FIS1_HUMAN] | 4 | 6 | 5 | 152 | 16.9 | 8.79 |
| P29992 | Guanine nucleotide-binding protein subunit alpha-11 OS=Homo sapiens GN=GNA11 PE=1 SV=2 - [GNA11_HUMAN] | 4 | 5 | 7 | 359 | 42.1 | 5.69 |
| P61254 | 60S ribosomal protein L26 OS=Homo sapiens GN=RPL26 PE=1 SV=1 - [RL26_HUMAN] | 4 | 3 | 4 | 145 | 17.2 | 10.55 |
| P62913 | 60S ribosomal protein L11 OS=Homo sapiens GN=RPL11 PE=1 SV=2 - [RL11_HUMAN] | 4 | 5 | 5 | 178 | 20.2 | 9.60 |
| Q9NRV9 | Heme-binding protein 1 OS=Homo sapiens GN=HEBP1 PE=1 SV=1 - [HEBP1_HUMAN] | 4 | 3 | 4 | 189 | 21.1 | 5.80 |
| P35270 | Sepiapterin reductase OS=Homo sapiens GN=SPR PE=1 SV=1 - [SPRE_HUMAN] | 4 | 5 | 7 | 261 | 28.0 | 8.05 |
| Q9Y2Q3 | Glutathione S-transferase kappa 1 OS=Homo sapiens GN=GSTK1 PE=1 SV=3 - [GSTK1_HUMAN] | 4 | 4 | 6 | 226 | 25.5 | 8.41 |
| Q9Y2B0 | Protein canopy homolog 2 OS=Homo sapiens GN=CNPY2 PE=1 SV=1 - [CNPY2_HUMAN] | 4 | 5 | 2 | 182 | 20.6 | 4.92 |
| P52597 | Heterogeneous nuclear ribonucleoprotein F OS=Homo sapiens GN=HNRNPF PE=1 SV=3 - [HNRPF_HUMAN] | 4 | 8 | 9 | 415 | 45.6 | 5.58 |
| P13693 | Translationally-controlled tumor protein OS=Homo sapiens GN=TPT1 PE=1 SV=1 - [TCTP_HUMAN] | 4 | 3 | 3 | 172 | 19.6 | 4.93 |
| Q9GZS3 | WD repeat-containing protein 61 OS=Homo sapiens GN=WDR61 PE=1 SV=1 - [WDR61_HUMAN] | 4 | 5 | 4 | 305 | 33.6 | 5.47 |
| Q13310 | Polyadenylate-binding protein 4 OS=Homo sapiens GN=PABPC4 PE=1 SV=1 - [PABP4_HUMAN] | 4 | 10 | 13 | 644 | 70.7 | 9.26 |
| P09601 | Heme oxygenase 1 OS=Homo sapiens GN=HMOX1 PE=1 SV=1 - [HMOX1_HUMAN] | 4 | 4 | 5 | 288 | 32.8 | 8.25 |
| P15559 | NAD(P)H dehydrogenase [quinone] 1 OS=Homo sapiens GN=NQO1 PE=1 SV=1 - [NQO1_HUMAN] | 4 | 3 | 4 | 274 | 30.8 | 8.88 |
| Q14165 | Malectin OS=Homo sapiens GN=MLEC PE=1 SV=1 - [MLEC_HUMAN] | 4 | 5 | 3 | 292 | 32.2 | 5.41 |
| P62879 | Guanine nucleotide-binding protein G(I)/G(S)/G(T) subunit beta-2 OS=Homo sapiens GN=GNB2 PE=1 SV=3 - [GBB2_HUMAN] | 4 | 6 | 7 | 340 | 37.3 | 6.00 |
| Q9H4A6 | Golgi phosphoprotein 3 OS=Homo sapiens GN=GOLPH3 PE=1 SV=1 - [GOLP3_HUMAN] | 4 | 4 | 3 | 298 | 33.8 | 6.44 |
| Q02543 | 60S ribosomal protein L18a OS=Homo sapiens GN=RPL18A PE=1 SV=2 - [RL18A_HUMAN] | 4 | 3 | 4 | 176 | 20.7 | 10.71 |
| P36507 | Dual specificity mitogen-activated protein kinase kinase 2 OS=Homo sapiens GN=MAP2K2 PE=1 SV=1 - [MP2K2_HUMAN] | 4 | 5 | 4 | 400 | 44.4 | 6.55 |
| Q8IZP2 | Protein FAM10A4 OS=Homo sapiens GN=FAM10A4 PE=1 SV=1 - [F10A4_HUMAN] | 4 | 4 | 5 | 240 | 27.4 | 5.08 |
| Q9BTV4 | Transmembrane protein 43 OS=Homo sapiens GN=TMEM43 PE=1 SV=1 - [TMM43_HUMAN] | 4 | 4 | 4 | 400 | 44.8 | 8.13 |
| P46776 | 60S ribosomal protein L27a OS=Homo sapiens GN=RPL27A PE=1 SV=2 - [RL27A_HUMAN] | 4 | 4 | 4 | 148 | 16.6 | 11.00 |
| O00743 | Serine/threonine-protein phosphatase 6 catalytic subunit OS=Homo sapiens GN=PPP6C PE=1 SV=1 - [PPP6_HUMAN] | 4 | 3 | 4 | 305 | 35.1 | 5.69 |
| P83731 | 60S ribosomal protein L24 OS=Homo sapiens GN=RPL24 PE=1 SV=1 - [RL24_HUMAN] | 4 | 4 | 4 | 157 | 17.8 | 11.25 |
| P39019 | 40S ribosomal protein S19 OS=Homo sapiens GN=RPS19 PE=1 SV=2 - [RS19_HUMAN] | 4 | 4 | 4 | 145 | 16.1 | 10.32 |
| P02794 | Ferritin heavy chain OS=Homo sapiens GN=FTH1 PE=1 SV=2 - [FRIH_HUMAN] | 4 | 5 | 4 | 183 | 21.2 | 5.55 |
| Q86V81 | THO complex subunit 4 OS=Homo sapiens GN=THOC4 PE=1 SV=3 - [THOC4_HUMAN] | 4 | 5 | 4 | 257 | 26.9 | 11.15 |
| Q9P0L0 | Vesicle-associated membrane protein-associated protein A OS=Homo sapiens GN=VAPA PE=1 SV=3 - [VAPA_HUMAN] | 4 | 6 | 3 | 249 | 27.9 | 8.62 |
| P16401 | Histone H1.5 OS=Homo sapiens GN=HIST1H1B PE=1 SV=3 - [H15_HUMAN] | 4 | 3 | 5 | 226 | 22.6 | 10.92 |
| O75396 | Vesicle-trafficking protein SEC22b OS=Homo sapiens GN=SEC22B PE=1 SV=4 - [SC22B_HUMAN] | 4 | 2 | 4 | 215 | 24.6 | 6.92 |
| Q9Y696 | Chloride intracellular channel protein 4 OS=Homo sapiens GN=CLIC4 PE=1 SV=4 - [CLIC4_HUMAN] | 4 | 5 | 6 | 253 | 28.8 | 5.59 |
| P53007 | Tricarboxylate transport protein. mitochondrial OS=Homo sapiens GN=SLC25A1 PE=1 SV=2 - [TXTP_HUMAN] | 4 | 5 | 6 | 311 | 34.0 | 9.89 |
| P28066 | Proteasome subunit alpha type-5 OS=Homo sapiens GN=PSMA5 PE=1 SV=3 - [PSA5_HUMAN] | 4 | 3 | 3 | 241 | 26.4 | 4.79 |
| Q14103 | Heterogeneous nuclear ribonucleoprotein D0 OS=Homo sapiens GN=HNRNPD PE=1 SV=1 - [HNRPD_HUMAN] | 4 | 7 | 9 | 355 | 38.4 | 7.81 |
| P26373 | 60S ribosomal protein L13 OS=Homo sapiens GN=RPL13 PE=1 SV=4 - [RL13_HUMAN] | 4 | 5 | 5 | 211 | 24.2 | 11.65 |
| P16152 | Carbonyl reductase [NADPH] 1 OS=Homo sapiens GN=CBR1 PE=1 SV=3 - [CBR1_HUMAN] | 4 | 3 | 4 | 277 | 30.4 | 8.32 |
| Q9P287 | BRCA2 and CDKN1A-interacting protein OS=Homo sapiens GN=BCCIP PE=1 SV=1 - [BCCIP_HUMAN] | 4 | 4 | 3 | 314 | 36.0 | 4.61 |
| Q14376 | UDP-glucose 4-epimerase OS=Homo sapiens GN=GALE PE=1 SV=2 - [GALE_HUMAN] | 4 | 5 | 4 | 348 | 38.3 | 6.73 |
| Q9H7Z7 | Prostaglandin E synthase 2 OS=Homo sapiens GN=PTGES2 PE=1 SV=1 - [PGES2_HUMAN] | 4 | 3 | 3 | 377 | 41.9 | 9.16 |
| O75489 | NADH dehydrogenase [ubiquinone] iron-sulfur protein 3. mitochondrial OS=Homo sapiens GN=NDUFS3 PE=1 SV=1 - [NDUS3_HUMAN] | 4 | 3 | 3 | 264 | 30.2 | 7.50 |
| P31942 | Heterogeneous nuclear ribonucleoprotein H3 OS=Homo sapiens GN=HNRNPH3 PE=1 SV=2 - [HNRH3_HUMAN] | 4 | 3 | 4 | 346 | 36.9 | 6.87 |
| Q16543 | Hsp90 co-chaperone Cdc37 OS=Homo sapiens GN=CDC37 PE=1 SV=1 - [CDC37_HUMAN] | 4 | 1 | 4 | 378 | 44.4 | 5.25 |
| P05089 | Arginase-1 OS=Homo sapiens GN=ARG1 PE=1 SV=2 - [ARGI1_HUMAN] | 4 | 3 | 4 | 322 | 34.7 | 7.21 |
| Q8TCT9 | Minor histocompatibility antigen H13 OS=Homo sapiens GN=HM13 PE=1 SV=1 - [HM13_HUMAN] | 4 | 5 | 5 | 377 | 41.5 | 6.43 |
| P40429 | 60S ribosomal protein L13a OS=Homo sapiens GN=RPL13A PE=1 SV=2 - [RL13A_HUMAN] | 4 | 6 | 5 | 203 | 23.6 | 10.93 |
| O75356 | Ectonucleoside triphosphate diphosphohydrolase 5 OS=Homo sapiens GN=ENTPD5 PE=1 SV=1 - [ENTP5_HUMAN] | 4 | 2 | 4 | 428 | 47.5 | 6.33 |
| Q9Y5X3 | Sorting nexin-5 OS=Homo sapiens GN=SNX5 PE=1 SV=1 - [SNX5_HUMAN] | 4 | 4 | 2 | 404 | 46.8 | 6.76 |
| Q9P035 | Protein tyrosine phosphatase-like protein PTPLAD1 OS=Homo sapiens GN=PTPLAD1 PE=1 SV=2 - [PTAD1_HUMAN] | 4 | 2 | 5 | 362 | 43.1 | 8.94 |
| Q7Z4W1 | L-xylulose reductase OS=Homo sapiens GN=DCXR PE=1 SV=2 - [DCXR_HUMAN] | 4 | 3 | 3 | 244 | 25.9 | 8.10 |
| Q9NR45 | Sialic acid synthase OS=Homo sapiens GN=NANS PE=1 SV=2 - [SIAS_HUMAN] | 4 | 3 | 4 | 359 | 40.3 | 6.74 |
| O00629 | Importin subunit alpha-4 OS=Homo sapiens GN=KPNA4 PE=1 SV=1 - [IMA4_HUMAN] | 4 | 8 | 4 | 521 | 57.9 | 4.96 |
| P51570 | Galactokinase OS=Homo sapiens GN=GALK1 PE=1 SV=1 - [GALK1_HUMAN] | 4 | 4 | 3 | 392 | 42.2 | 6.46 |
| Q9NUJ1 | Mycophenolic acid acyl-glucuronide esterase. mitochondrial OS=Homo sapiens GN=ABHD10 PE=1 SV=1 - [ABHDA_HUMAN] | 4 | 2 | 5 | 306 | 33.9 | 8.57 |
| Q9H2U2 | Inorganic pyrophosphatase 2. mitochondrial OS=Homo sapiens GN=PPA2 PE=1 SV=2 - [IPYR2_HUMAN] | 4 | 5 | 4 | 334 | 37.9 | 7.39 |
| O96019 | Actin-like protein 6A OS=Homo sapiens GN=ACTL6A PE=1 SV=1 - [ACL6A_HUMAN] | 4 | 3 | 3 | 429 | 47.4 | 5.60 |
| P11766 | Alcohol dehydrogenase class-3 OS=Homo sapiens GN=ADH5 PE=1 SV=4 - [ADHX_HUMAN] | 4 | 2 | 4 | 374 | 39.7 | 7.49 |
| Q99729 | Heterogeneous nuclear ribonucleoprotein A/B OS=Homo sapiens GN=HNRNPAB PE=1 SV=2 - [ROAA_HUMAN] | 4 | 5 | 5 | 332 | 36.2 | 8.21 |
| Q16698 | 2.4-dienoyl-CoA reductase. mitochondrial OS=Homo sapiens GN=DECR1 PE=1 SV=1 - [DECR_HUMAN] | 4 | 3 | 3 | 335 | 36.0 | 9.28 |
| Q9UHD1 | Cysteine and histidine-rich domain-containing protein 1 OS=Homo sapiens GN=CHORDC1 PE=1 SV=2 - [CHRD1_HUMAN] | 4 | 1 | 4 | 332 | 37.5 | 7.87 |
| Q9Y624 | Junctional adhesion molecule A OS=Homo sapiens GN=F11R PE=1 SV=1 - [JAM1_HUMAN] | 4 | 3 | 3 | 299 | 32.6 | 7.90 |
| O00764 | Pyridoxal kinase OS=Homo sapiens GN=PDXK PE=1 SV=1 - [PDXK_HUMAN] | 4 | 6 | 5 | 312 | 35.1 | 6.13 |
| P23258 | Tubulin gamma-1 chain OS=Homo sapiens GN=TUBG1 PE=1 SV=2 - [TBG1_HUMAN] | 4 | 3 | 3 | 451 | 51.1 | 6.14 |
| Q14651 | Plastin-1 OS=Homo sapiens GN=PLS1 PE=1 SV=2 - [PLSI_HUMAN] | 4 | 6 | 9 | 629 | 70.2 | 5.41 |
| P35250 | Replication factor C subunit 2 OS=Homo sapiens GN=RFC2 PE=1 SV=3 - [RFC2_HUMAN] | 4 | 3 | 5 | 354 | 39.1 | 6.44 |
| O14979 | Heterogeneous nuclear ribonucleoprotein D-like OS=Homo sapiens GN=HNRPDL PE=1 SV=3 - [HNRDL_HUMAN] | 4 | 6 | 7 | 420 | 46.4 | 9.57 |
| O95433 | Activator of 90 kDa heat shock protein ATPase homolog 1 OS=Homo sapiens GN=AHSA1 PE=1 SV=1 - [AHSA1_HUMAN] | 4 | 3 | 3 | 338 | 38.3 | 5.53 |
| Q53GQ0 | Estradiol 17-beta-dehydrogenase 12 OS=Homo sapiens GN=HSD17B12 PE=1 SV=2 - [DHB12_HUMAN] | 4 | 4 | 3 | 312 | 34.3 | 9.32 |
| P54619 | 5'-AMP-activated protein kinase subunit gamma-1 OS=Homo sapiens GN=PRKAG1 PE=1 SV=1 - [AAKG1_HUMAN] | 4 | 7 | 5 | 331 | 37.6 | 6.92 |
| O60256 | Phosphoribosyl pyrophosphate synthase-associated protein 2 OS=Homo sapiens GN=PRPSAP2 PE=1 SV=1 - [KPRB_HUMAN] | 4 | 1 | 4 | 369 | 40.9 | 7.44 |
| Q15257 | Serine/threonine-protein phosphatase 2A regulatory subunit B' OS=Homo sapiens GN=PPP2R4 PE=1 SV=3 - [PTPA_HUMAN] | 4 | 4 | 5 | 358 | 40.6 | 5.94 |
| Q12904 | Aminoacyl tRNA synthetase complex-interacting multifunctional protein 1 OS=Homo sapiens GN=AIMP1 PE=1 SV=2 - [AIMP1_HUMAN] | 4 | 3 | 3 | 312 | 34.3 | 8.43 |
| P55263 | Adenosine kinase OS=Homo sapiens GN=ADK PE=1 SV=2 - [ADK_HUMAN] | 4 | 4 | 4 | 362 | 40.5 | 6.70 |
| Q99961 | Endophilin-A2 OS=Homo sapiens GN=SH3GL1 PE=1 SV=1 - [SH3G1_HUMAN] | 4 | 4 | 3 | 368 | 41.5 | 5.43 |
| P34913 | Bifunctional epoxide hydrolase 2 OS=Homo sapiens GN=EPHX2 PE=1 SV=2 - [HYES_HUMAN] | 4 | 4 | 3 | 555 | 62.6 | 6.28 |
| Q92734 | Protein TFG OS=Homo sapiens GN=TFG PE=1 SV=2 - [TFG_HUMAN] | 4 | 4 | 2 | 400 | 43.4 | 5.10 |
| O75436 | Vacuolar protein sorting-associated protein 26A OS=Homo sapiens GN=VPS26A PE=1 SV=2 - [VP26A_HUMAN] | 4 | 4 | 3 | 327 | 38.1 | 6.57 |
| Q8TDN6 | Ribosome biogenesis protein BRX1 homolog OS=Homo sapiens GN=BRIX1 PE=1 SV=2 - [BRX1_HUMAN] | 4 | 3 | 3 | 353 | 41.4 | 9.92 |
| O95340 | Bifunctional 3'-phosphoadenosine 5'-phosphosulfate synthase 2 OS=Homo sapiens GN=PAPSS2 PE=1 SV=2 - [PAPS2_HUMAN] | 4 | 7 | 5 | 614 | 69.5 | 8.03 |
| P10644 | cAMP-dependent protein kinase type I-alpha regulatory subunit OS=Homo sapiens GN=PRKAR1A PE=1 SV=1 - [KAP0_HUMAN] | 4 | 3 | 4 | 381 | 43.0 | 5.35 |
| P21283 | V-type proton ATPase subunit C 1 OS=Homo sapiens GN=ATP6V1C1 PE=1 SV=4 - [VATC1_HUMAN] | 4 | 2 | 3 | 382 | 43.9 | 7.46 |
| Q13283 | Ras GTPase-activating protein-binding protein 1 OS=Homo sapiens GN=G3BP1 PE=1 SV=1 - [G3BP1_HUMAN] | 4 | 2 | 5 | 466 | 52.1 | 5.52 |
| Q9Y3I0 | UPF0027 protein C22orf28 OS=Homo sapiens GN=C22orf28 PE=1 SV=1 - [CV028_HUMAN] | 4 | 3 | 3 | 505 | 55.2 | 7.23 |
| P04632 | Calpain small subunit 1 OS=Homo sapiens GN=CAPNS1 PE=1 SV=1 - [CPNS1_HUMAN] | 4 | 5 | 3 | 268 | 28.3 | 5.20 |
| P53990 | IST1 homolog OS=Homo sapiens GN=KIAA0174 PE=1 SV=1 - [IST1_HUMAN] | 4 | 3 | 3 | 364 | 39.7 | 5.35 |
| P04083 | Annexin A1 OS=Homo sapiens GN=ANXA1 PE=1 SV=2 - [ANXA1_HUMAN] | 4 | 4 | 3 | 346 | 38.7 | 7.02 |
| P53985 | Monocarboxylate transporter 1 OS=Homo sapiens GN=SLC16A1 PE=1 SV=3 - [MOT1_HUMAN] | 4 | 2 | 4 | 500 | 53.9 | 8.66 |
| Q9NZ01 | Trans-2.3-enoyl-CoA reductase OS=Homo sapiens GN=TECR PE=1 SV=1 - [TECR_HUMAN] | 4 | 5 | 3 | 308 | 36.0 | 9.45 |
| P37059 | Estradiol 17-beta-dehydrogenase 2 OS=Homo sapiens GN=HSD17B2 PE=1 SV=1 - [DHB2_HUMAN] | 4 | 3 | 4 | 387 | 42.8 | 8.50 |
| Q15427 | Splicing factor 3B subunit 4 OS=Homo sapiens GN=SF3B4 PE=1 SV=1 - [SF3B4_HUMAN] | 4 | 3 | 5 | 424 | 44.4 | 8.56 |
| Q9Y2T2 | AP-3 complex subunit mu-1 OS=Homo sapiens GN=AP3M1 PE=1 SV=1 - [AP3M1_HUMAN] | 4 | 3 | 5 | 418 | 46.9 | 6.93 |
| P41235 | Hepatocyte nuclear factor 4-alpha OS=Homo sapiens GN=HNF4A PE=1 SV=3 - [HNF4A_HUMAN] | 4 | 2 | 3 | 474 | 52.8 | 7.43 |
| P35613 | Basigin OS=Homo sapiens GN=BSG PE=1 SV=2 - [BASI_HUMAN] | 4 | 6 | 6 | 385 | 42.2 | 5.66 |
| P04062 | Glucosylceramidase OS=Homo sapiens GN=GBA PE=1 SV=3 - [GLCM_HUMAN] | 4 | 3 | 3 | 536 | 59.7 | 7.61 |
| Q13177 | Serine/threonine-protein kinase PAK 2 OS=Homo sapiens GN=PAK2 PE=1 SV=3 - [PAK2_HUMAN] | 4 | 4 | 5 | 524 | 58.0 | 5.96 |
| O75844 | CAAX prenyl protease 1 homolog OS=Homo sapiens GN=ZMPSTE24 PE=1 SV=2 - [FACE1_HUMAN] | 4 | 5 | 4 | 475 | 54.8 | 7.49 |
| Q9Y4P3 | Transducin beta-like protein 2 OS=Homo sapiens GN=TBL2 PE=1 SV=1 - [TBL2_HUMAN] | 4 | 3 | 3 | 447 | 49.8 | 9.44 |
| P42330 | Aldo-keto reductase family 1 member C3 OS=Homo sapiens GN=AKR1C3 PE=1 SV=4 - [AK1C3_HUMAN] | 4 | 5 | 4 | 323 | 36.8 | 7.94 |
| Q8NBJ4 | Golgi membrane protein 1 OS=Homo sapiens GN=GOLM1 PE=1 SV=1 - [GOLM1_HUMAN] | 4 | 2 | 4 | 401 | 45.3 | 4.97 |
| Q07666 | KH domain-containing. RNA-binding. signal transduction-associated protein 1 OS=Homo sapiens GN=KHDRBS1 PE=1 SV=1 - [KHDR1_HUMAN] | 4 | 5 | 6 | 443 | 48.2 | 8.66 |
| Q8NC51 | Plasminogen activator inhibitor 1 RNA-binding protein OS=Homo sapiens GN=SERBP1 PE=1 SV=2 - [PAIRB_HUMAN] | 4 | 6 | 6 | 408 | 44.9 | 8.65 |
| O14773 | Tripeptidyl-peptidase 1 OS=Homo sapiens GN=TPP1 PE=1 SV=2 - [TPP1_HUMAN] | 4 | 1 | 5 | 563 | 61.2 | 6.48 |
| P62333 | 26S protease regulatory subunit S10B OS=Homo sapiens GN=PSMC6 PE=1 SV=1 - [PRS10_HUMAN] | 4 | 5 | 5 | 389 | 44.1 | 7.49 |
| O60784 | Target of Myb protein 1 OS=Homo sapiens GN=TOM1 PE=1 SV=2 - [TOM1_HUMAN] | 4 | 2 | 3 | 492 | 53.8 | 4.70 |
| Q8WX92 | Negative elongation factor B OS=Homo sapiens GN=COBRA1 PE=1 SV=1 - [NELFB_HUMAN] | 4 | 3 | 4 | 580 | 65.7 | 6.13 |
| O75907 | Diacylglycerol O-acyltransferase 1 OS=Homo sapiens GN=DGAT1 PE=1 SV=2 - [DGAT1_HUMAN] | 4 | 4 | 3 | 488 | 55.2 | 9.32 |
| Q9UHG3 | Prenylcysteine oxidase 1 OS=Homo sapiens GN=PCYOX1 PE=1 SV=3 - [PCYOX_HUMAN] | 4 | 5 | 4 | 505 | 56.6 | 6.18 |
| Q6XQN6 | Nicotinate phosphoribosyltransferase OS=Homo sapiens GN=NAPRT1 PE=1 SV=2 - [PNCB_HUMAN] | 4 | 2 | 5 | 538 | 57.5 | 5.68 |
| P14324 | Farnesyl pyrophosphate synthase OS=Homo sapiens GN=FDPS PE=1 SV=4 - [FPPS_HUMAN] | 4 | 4 | 4 | 419 | 48.2 | 6.15 |
| O75131 | Copine-3 OS=Homo sapiens GN=CPNE3 PE=1 SV=1 - [CPNE3_HUMAN] | 4 | 3 | 3 | 537 | 60.1 | 5.85 |
| P07686 | Beta-hexosaminidase subunit beta OS=Homo sapiens GN=HEXB PE=1 SV=3 - [HEXB_HUMAN] | 4 | 3 | 3 | 556 | 63.1 | 6.76 |
| P02675 | Fibrinogen beta chain OS=Homo sapiens GN=FGB PE=1 SV=2 - [FIBB_HUMAN] | 4 | 1 | 4 | 491 | 55.9 | 8.27 |
| O75955 | Flotillin-1 OS=Homo sapiens GN=FLOT1 PE=1 SV=3 - [FLOT1_HUMAN] | 4 | 3 | 3 | 427 | 47.3 | 7.49 |
| P37268 | Squalene synthase OS=Homo sapiens GN=FDFT1 PE=1 SV=1 - [FDFT_HUMAN] | 4 | 1 | 4 | 417 | 48.1 | 6.54 |
| Q9BY77 | Polymerase delta-interacting protein 3 OS=Homo sapiens GN=POLDIP3 PE=1 SV=2 - [PDIP3_HUMAN] | 4 | 3 | 3 | 421 | 46.1 | 9.99 |
| Q15008 | 26S proteasome non-ATPase regulatory subunit 6 OS=Homo sapiens GN=PSMD6 PE=1 SV=1 - [PSMD6_HUMAN] | 4 | 3 | 4 | 389 | 45.5 | 5.62 |
| O00499 | Myc box-dependent-interacting protein 1 OS=Homo sapiens GN=BIN1 PE=1 SV=1 - [BIN1_HUMAN] | 4 | 3 | 4 | 593 | 64.7 | 5.06 |
| P26572 | Alpha-1.3-mannosyl-glycoprotein 2-beta-N-acetylglucosaminyltransferase OS=Homo sapiens GN=MGAT1 PE=1 SV=2 - [MGAT1_HUMAN] | 4 | 2 | 3 | 445 | 50.8 | 9.16 |
| Q9ULA0 | Aspartyl aminopeptidase OS=Homo sapiens GN=DNPEP PE=1 SV=1 - [DNPEP_HUMAN] | 4 | 2 | 3 | 475 | 52.4 | 7.42 |
| Q709F0 | Acyl-CoA dehydrogenase family member 11 OS=Homo sapiens OX=9606 GN=ACAD11 PE=1 SV=3 - [ACD11_HUMAN] | 4 | 3 | 5 | 780 | 87.2 | 8.02 |
| Q16658 | Fascin OS=Homo sapiens GN=FSCN1 PE=1 SV=3 - [FSCN1_HUMAN] | 4 | 3 | 5 | 493 | 54.5 | 7.24 |
| O14744 | Protein arginine N-methyltransferase 5 OS=Homo sapiens GN=PRMT5 PE=1 SV=4 - [ANM5_HUMAN] | 4 | 3 | 5 | 637 | 72.6 | 6.29 |
| P49591 | Seryl-tRNA synthetase. cytoplasmic OS=Homo sapiens GN=SARS PE=1 SV=3 - [SYSC_HUMAN] | 4 | 3 | 4 | 514 | 58.7 | 6.43 |
| Q92990 | Glomulin OS=Homo sapiens GN=GLMN PE=1 SV=2 - [GLMN_HUMAN] | 4 | 4 | 3 | 594 | 68.2 | 5.33 |
| P21281 | V-type proton ATPase subunit B. brain isoform OS=Homo sapiens GN=ATP6V1B2 PE=1 SV=3 - [VATB2_HUMAN] | 4 | 4 | 5 | 511 | 56.5 | 5.81 |
| Q96CM8 | Acyl-CoA synthetase family member 2. mitochondrial OS=Homo sapiens GN=ACSF2 PE=1 SV=2 - [ACSF2_HUMAN] | 4 | 2 | 5 | 615 | 68.1 | 7.55 |
| Q10471 | Polypeptide N-acetylgalactosaminyltransferase 2 OS=Homo sapiens GN=GALNT2 PE=1 SV=1 - [GALT2_HUMAN] | 4 | 2 | 3 | 571 | 64.7 | 8.35 |
| Q9H3G5 | Probable serine carboxypeptidase CPVL OS=Homo sapiens GN=CPVL PE=1 SV=2 - [CPVL_HUMAN] | 4 | 2 | 3 | 476 | 54.1 | 5.62 |
| Q14108 | Lysosome membrane protein 2 OS=Homo sapiens GN=SCARB2 PE=1 SV=2 - [SCRB2_HUMAN] | 4 | 4 | 6 | 478 | 54.3 | 5.14 |
| Q14498 | RNA-binding protein 39 OS=Homo sapiens GN=RBM39 PE=1 SV=2 - [RBM39_HUMAN] | 4 | 3 | 4 | 530 | 59.3 | 10.10 |
| P33908 | Mannosyl-oligosaccharide 1.2-alpha-mannosidase IA OS=Homo sapiens GN=MAN1A1 PE=1 SV=3 - [MA1A1_HUMAN] | 4 | 2 | 4 | 653 | 72.9 | 6.47 |
| Q5R3I4 | Tetratricopeptide repeat protein 38 OS=Homo sapiens GN=TTC38 PE=1 SV=1 - [TTC38_HUMAN] | 4 | 4 | 3 | 469 | 52.8 | 5.99 |
| Q9Y2X3 | Nucleolar protein 58 OS=Homo sapiens GN=NOP58 PE=1 SV=1 - [NOP58_HUMAN] | 4 | 4 | 2 | 529 | 59.5 | 8.92 |
| Q9H9A6 | Leucine-rich repeat-containing protein 40 OS=Homo sapiens GN=LRRC40 PE=1 SV=1 - [LRC40_HUMAN] | 4 | 4 | 3 | 602 | 68.2 | 6.43 |
| O15427 | Monocarboxylate transporter 4 OS=Homo sapiens GN=SLC16A3 PE=1 SV=1 - [MOT4_HUMAN] | 4 | 4 | 2 | 465 | 49.4 | 7.96 |
| Q96HC4 | PDZ and LIM domain protein 5 OS=Homo sapiens GN=PDLIM5 PE=1 SV=5 - [PDLI5_HUMAN] | 4 | 4 | 3 | 596 | 63.9 | 8.21 |
| Q06124 | Tyrosine-protein phosphatase non-receptor type 11 OS=Homo sapiens GN=PTPN11 PE=1 SV=2 - [PTN11_HUMAN] | 4 | 2 | 4 | 597 | 68.4 | 7.30 |
| Q92542 | Nicastrin OS=Homo sapiens GN=NCSTN PE=1 SV=2 - [NICA_HUMAN] | 4 | 5 | 3 | 709 | 78.4 | 5.99 |
| P80404 | 4-aminobutyrate aminotransferase. mitochondrial OS=Homo sapiens GN=ABAT PE=1 SV=3 - [GABT_HUMAN] | 4 | 3 | 6 | 500 | 56.4 | 7.96 |
| P33121 | Long-chain-fatty-acid--CoA ligase 1 OS=Homo sapiens GN=ACSL1 PE=1 SV=1 - [ACSL1_HUMAN] | 4 | 5 | 3 | 698 | 77.9 | 7.15 |
| Q13620 | Cullin-4B OS=Homo sapiens GN=CUL4B PE=1 SV=4 - [CUL4B_HUMAN] | 4 | 7 | 7 | 913 | 103.9 | 7.37 |
| O95782 | AP-2 complex subunit alpha-1 OS=Homo sapiens GN=AP2A1 PE=1 SV=3 - [AP2A1_HUMAN] | 4 | 4 | 5 | 977 | 107.5 | 7.03 |
| P23921 | Ribonucleoside-diphosphate reductase large subunit OS=Homo sapiens GN=RRM1 PE=1 SV=1 - [RIR1_HUMAN] | 4 | 3 | 4 | 792 | 90.0 | 7.15 |
| Q9NZT2 | Opioid growth factor receptor OS=Homo sapiens GN=OGFR PE=1 SV=3 - [OGFR_HUMAN] | 4 | 3 | 5 | 677 | 73.3 | 4.84 |
| Q9H223 | EH domain-containing protein 4 OS=Homo sapiens GN=EHD4 PE=1 SV=1 - [EHD4_HUMAN] | 4 | 3 | 2 | 541 | 61.1 | 6.76 |
| Q9NUU7 | ATP-dependent RNA helicase DDX19A OS=Homo sapiens GN=DDX19A PE=1 SV=1 - [DD19A_HUMAN] | 4 | 4 | 4 | 478 | 53.9 | 6.58 |
| P36957 | Dihydrolipoyllysine-residue succinyltransferase component of 2-oxoglutarate dehydrogenase complex. mitochondrial OS=Homo sapiens GN=DLST PE=1 SV=4 - [ODO2_HUMAN] | 4 | 3 | 3 | 453 | 48.7 | 8.95 |
| P11279 | Lysosome-associated membrane glycoprotein 1 OS=Homo sapiens GN=LAMP1 PE=1 SV=3 - [LAMP1_HUMAN] | 4 | 5 | 4 | 417 | 44.9 | 8.75 |
| Q5SSJ5 | Heterochromatin protein 1-binding protein 3 OS=Homo sapiens GN=HP1BP3 PE=1 SV=1 - [HP1B3_HUMAN] | 4 | 3 | 3 | 553 | 61.2 | 9.67 |
| Q9NTJ5 | Phosphatidylinositide phosphatase SAC1 OS=Homo sapiens GN=SACM1L PE=1 SV=2 - [SAC1_HUMAN] | 4 | 2 | 4 | 587 | 66.9 | 7.12 |
| Q08426 | Peroxisomal bifunctional enzyme OS=Homo sapiens GN=EHHADH PE=1 SV=3 - [ECHP_HUMAN] | 4 | 2 | 3 | 723 | 79.4 | 9.14 |
| P15151 | Poliovirus receptor OS=Homo sapiens GN=PVR PE=1 SV=2 - [PVR_HUMAN] | 4 | 5 | 2 | 417 | 45.3 | 6.52 |
| Q14166 | Tubulin--tyrosine ligase-like protein 12 OS=Homo sapiens GN=TTLL12 PE=1 SV=2 - [TTL12_HUMAN] | 4 | 3 | 3 | 644 | 74.4 | 5.53 |
| P11387 | DNA topoisomerase 1 OS=Homo sapiens GN=TOP1 PE=1 SV=2 - [TOP1_HUMAN] | 4 | 4 | 3 | 765 | 90.7 | 9.31 |
| P49756 | RNA-binding protein 25 OS=Homo sapiens GN=RBM25 PE=1 SV=3 - [RBM25_HUMAN] | 4 | 3 | 3 | 843 | 100.1 | 6.32 |
| Q5BKZ1 | DBIRD complex subunit ZNF326 OS=Homo sapiens GN=ZNF326 PE=1 SV=2 - [ZN326_HUMAN] | 4 | 4 | 4 | 582 | 65.6 | 5.15 |
| P15586 | N-acetylglucosamine-6-sulfatase OS=Homo sapiens GN=GNS PE=1 SV=3 - [GNS_HUMAN] | 4 | 3 | 4 | 552 | 62.0 | 8.31 |
| Q14444 | Caprin-1 OS=Homo sapiens GN=CAPRIN1 PE=1 SV=2 - [CAPR1_HUMAN] | 4 | 5 | 6 | 709 | 78.3 | 5.25 |
| P32189 | Glycerol kinase OS=Homo sapiens GN=GK PE=1 SV=3 - [GLPK_HUMAN] | 4 | 3 | 2 | 559 | 61.2 | 6.54 |
| P08237 | 6-phosphofructokinase. muscle type OS=Homo sapiens GN=PFKM PE=1 SV=2 - [K6PF_HUMAN] | 4 | 1 | 4 | 780 | 85.1 | 7.99 |
| P12931 | Proto-oncogene tyrosine-protein kinase Src OS=Homo sapiens GN=SRC PE=1 SV=3 - [SRC_HUMAN] | 4 | 3 | 3 | 536 | 59.8 | 7.42 |
| Q9BXJ9 | NMDA receptor-regulated protein 1 OS=Homo sapiens GN=NARG1 PE=1 SV=1 - [NARG1_HUMAN] | 4 | 4 | 1 | 866 | 101.2 | 7.42 |
| Q9H3U1 | Protein unc-45 homolog A OS=Homo sapiens GN=UNC45A PE=1 SV=1 - [UN45A_HUMAN] | 4 | 4 | 5 | 944 | 103.0 | 6.07 |
| O94826 | Mitochondrial import receptor subunit TOM70 OS=Homo sapiens GN=TOMM70A PE=1 SV=1 - [TOM70_HUMAN] | 4 | 4 | 5 | 608 | 67.4 | 7.12 |
| P22307 | Non-specific lipid-transfer protein OS=Homo sapiens GN=SCP2 PE=1 SV=2 - [NLTP_HUMAN] | 4 | 3 | 4 | 547 | 59.0 | 6.89 |
| P50416 | Carnitine O-palmitoyltransferase 1. liver isoform OS=Homo sapiens GN=CPT1A PE=1 SV=2 - [CPT1A_HUMAN] | 4 | 3 | 3 | 773 | 88.3 | 8.65 |
| Q1KMD3 | Heterogeneous nuclear ribonucleoprotein U-like protein 2 OS=Homo sapiens GN=HNRNPUL2 PE=1 SV=1 - [HNRL2_HUMAN] | 4 | 2 | 4 | 747 | 85.1 | 4.91 |
| Q8TAT6 | Nuclear protein localization protein 4 homolog OS=Homo sapiens GN=NPLOC4 PE=1 SV=3 - [NPL4_HUMAN] | 4 | 2 | 3 | 608 | 68.1 | 6.38 |
| Q9BUJ2 | Heterogeneous nuclear ribonucleoprotein U-like protein 1 OS=Homo sapiens GN=HNRNPUL1 PE=1 SV=2 - [HNRL1_HUMAN] | 4 | 2 | 4 | 856 | 95.7 | 6.92 |
| Q9UBN7 | Histone deacetylase 6 OS=Homo sapiens GN=HDAC6 PE=1 SV=2 - [HDAC6_HUMAN] | 4 | 2 | 3 | 1215 | 131.3 | 5.30 |
| P05556 | Integrin beta-1 OS=Homo sapiens GN=ITGB1 PE=1 SV=2 - [ITB1_HUMAN] | 4 | 4 | 3 | 798 | 88.4 | 5.39 |
| Q6NUQ4 | Transmembrane protein 214 OS=Homo sapiens GN=TMEM214 PE=1 SV=2 - [TM214_HUMAN] | 4 | 2 | 4 | 689 | 77.1 | 9.14 |
| Q9BUQ8 | Probable ATP-dependent RNA helicase DDX23 OS=Homo sapiens GN=DDX23 PE=1 SV=3 - [DDX23_HUMAN] | 4 | 5 | 3 | 820 | 95.5 | 9.55 |
| Q08J23 | tRNA (cytosine-5-)-methyltransferase NSUN2 OS=Homo sapiens GN=NSUN2 PE=1 SV=2 - [NSUN2_HUMAN] | 4 | 2 | 3 | 767 | 86.4 | 6.77 |
| Q92544 | Transmembrane 9 superfamily member 4 OS=Homo sapiens GN=TM9SF4 PE=1 SV=2 - [TM9S4_HUMAN] | 4 | 4 | 4 | 642 | 74.5 | 6.54 |
| O00541 | Pescadillo homolog OS=Homo sapiens GN=PES1 PE=1 SV=1 - [PESC_HUMAN] | 4 | 2 | 4 | 588 | 68.0 | 7.33 |
| Q13586 | Stromal interaction molecule 1 OS=Homo sapiens GN=STIM1 PE=1 SV=3 - [STIM1_HUMAN] | 4 | 1 | 3 | 685 | 77.4 | 6.67 |
| Q6Y7W6 | PERQ amino acid-rich with GYF domain-containing protein 2 OS=Homo sapiens GN=GIGYF2 PE=1 SV=1 - [PERQ2_HUMAN] | 4 | 2 | 3 | 1299 | 150.0 | 5.54 |
| O14974 | Protein phosphatase 1 regulatory subunit 12A OS=Homo sapiens GN=PPP1R12A PE=1 SV=1 - [MYPT1_HUMAN] | 4 | 2 | 4 | 1030 | 115.2 | 5.40 |
| Q6P2E9 | Enhancer of mRNA-decapping protein 4 OS=Homo sapiens GN=EDC4 PE=1 SV=1 - [EDC4_HUMAN] | 4 | 3 | 4 | 1401 | 151.6 | 5.86 |
| P10515 | Dihydrolipoyllysine-residue acetyltransferase component of pyruvate dehydrogenase complex. mitochondrial OS=Homo sapiens GN=DLAT PE=1 SV=3 - [ODP2_HUMAN] | 4 | 1 | 4 | 647 | 69.0 | 7.84 |
| Q96T37 | Putative RNA-binding protein 15 OS=Homo sapiens GN=RBM15 PE=1 SV=2 - [RBM15_HUMAN] | 4 | 1 | 5 | 977 | 107.1 | 10.08 |
| O96005 | Cleft lip and palate transmembrane protein 1 OS=Homo sapiens GN=CLPTM1 PE=1 SV=1 - [CLPT1_HUMAN] | 4 | 3 | 4 | 669 | 76.0 | 6.30 |
| Q92974 | Rho guanine nucleotide exchange factor 2 OS=Homo sapiens GN=ARHGEF2 PE=1 SV=4 - [ARHG2_HUMAN] | 4 | 4 | 3 | 986 | 111.5 | 7.27 |
| O15294 | UDP-N-acetylglucosamine--peptide N-acetylglucosaminyltransferase 110 kDa subunit OS=Homo sapiens GN=OGT PE=1 SV=3 - [OGT1_HUMAN] | 4 | 3 | 2 | 1046 | 116.8 | 6.70 |
| O76024 | Wolframin OS=Homo sapiens GN=WFS1 PE=1 SV=2 - [WFS1_HUMAN] | 4 | 3 | 3 | 890 | 100.2 | 8.05 |
| P15056 | Serine/threonine-protein kinase B-raf OS=Homo sapiens GN=BRAF PE=1 SV=4 - [BRAF_HUMAN] | 4 | 4 | 2 | 766 | 84.4 | 7.53 |
| Q9BX66 | Sorbin and SH3 domain-containing protein 1 OS=Homo sapiens GN=SORBS1 PE=1 SV=3 - [SRBS1_HUMAN] | 4 | 3 | 5 | 1292 | 142.4 | 6.84 |
| Q5JTH9 | RRP12-like protein OS=Homo sapiens GN=RRP12 PE=1 SV=2 - [RRP12_HUMAN] | 4 | 2 | 3 | 1297 | 143.6 | 8.75 |
| Q8WWM7 | Ataxin-2-like protein OS=Homo sapiens GN=ATXN2L PE=1 SV=2 - [ATX2L_HUMAN] | 4 | 4 | 4 | 1075 | 113.3 | 8.59 |
| Q13045 | Protein flightless-1 homolog OS=Homo sapiens GN=FLII PE=1 SV=2 - [FLII_HUMAN] | 4 | 3 | 3 | 1269 | 144.7 | 6.05 |
| Q8WUM0 | Nuclear pore complex protein Nup133 OS=Homo sapiens GN=NUP133 PE=1 SV=2 - [NU133_HUMAN] | 4 | 2 | 4 | 1156 | 128.9 | 5.10 |
| Q12769 | Nuclear pore complex protein Nup160 OS=Homo sapiens GN=NUP160 PE=1 SV=3 - [NU160_HUMAN] | 4 | 3 | 5 | 1436 | 162.0 | 5.50 |
| Q9HAV4 | Exportin-5 OS=Homo sapiens GN=XPO5 PE=1 SV=1 - [XPO5_HUMAN] | 4 | 2 | 3 | 1204 | 136.2 | 5.80 |
| Q9UHN6 | Transmembrane protein 2 OS=Homo sapiens GN=TMEM2 PE=1 SV=1 - [TMEM2_HUMAN] | 4 | 1 | 3 | 1383 | 154.3 | 8.15 |
| Q92620 | Pre-mRNA-splicing factor ATP-dependent RNA helicase PRP16 OS=Homo sapiens GN=DHX38 PE=1 SV=2 - [PRP16_HUMAN] | 4 | 5 | 4 | 1227 | 140.4 | 6.54 |
| Q15021 | Condensin complex subunit 1 OS=Homo sapiens GN=NCAPD2 PE=1 SV=3 - [CND1_HUMAN] | 4 | 5 | 5 | 1401 | 157.1 | 6.61 |
| Q9NQC3 | Reticulon-4 OS=Homo sapiens GN=RTN4 PE=1 SV=2 - [RTN4_HUMAN] | 4 | 5 | 5 | 1192 | 129.9 | 4.50 |
| Q9NTZ6 | RNA-binding protein 12 OS=Homo sapiens GN=RBM12 PE=1 SV=1 - [RBM12_HUMAN] | 4 | 3 | 4 | 932 | 97.3 | 8.63 |
| Q9H2M9 | Rab3 GTPase-activating protein non-catalytic subunit OS=Homo sapiens GN=RAB3GAP2 PE=1 SV=1 - [RBGPR_HUMAN] | 4 | 2 | 4 | 1393 | 155.9 | 5.62 |
| Q7L576 | Cytoplasmic FMR1-interacting protein 1 OS=Homo sapiens GN=CYFIP1 PE=1 SV=1 - [CYFP1_HUMAN] | 4 | 3 | 2 | 1253 | 145.1 | 6.90 |
| P02458 | Collagen alpha-1(II) chain OS=Homo sapiens GN=COL2A1 PE=1 SV=3 - [CO2A1_HUMAN] | 4 | 1 | 4 | 1487 | 141.7 | 6.92 |
| Q69YN4 | Protein virilizer homolog OS=Homo sapiens GN=KIAA1429 PE=1 SV=2 - [VIR_HUMAN] | 4 | 3 | 4 | 1812 | 201.9 | 5.01 |
| Q8NI27 | THO complex subunit 2 OS=Homo sapiens GN=THOC2 PE=1 SV=2 - [THOC2_HUMAN] | 4 | 3 | 4 | 1593 | 182.7 | 8.44 |
| Q92621 | Nuclear pore complex protein Nup205 OS=Homo sapiens GN=NUP205 PE=1 SV=3 - [NU205_HUMAN] | 4 | 4 | 2 | 2012 | 227.8 | 6.19 |
| P68371 | Tubulin beta-2C chain OS=Homo sapiens GN=TUBB2C PE=1 SV=1 - [TBB2C_HUMAN] | 3 | 76 | 78 | 445 | 49.8 | 4.89 |
| Q9BQE3 | Tubulin alpha-1C chain OS=Homo sapiens GN=TUBA1C PE=1 SV=1 - [TBA1C_HUMAN] | 3 | 74 | 76 | 449 | 49.9 | 5.10 |
| P84085 | ADP-ribosylation factor 5 OS=Homo sapiens GN=ARF5 PE=1 SV=2 - [ARF5_HUMAN] | 3 | 9 | 12 | 180 | 20.5 | 6.79 |
| P18085 | ADP-ribosylation factor 4 OS=Homo sapiens GN=ARF4 PE=1 SV=3 - [ARF4_HUMAN] | 3 | 9 | 10 | 180 | 20.5 | 7.14 |
| P22392 | Nucleoside diphosphate kinase B OS=Homo sapiens GN=NME2 PE=1 SV=1 - [NDKB_HUMAN] | 3 | 12 | 14 | 152 | 17.3 | 8.41 |
| Q9H0U4 | Ras-related protein Rab-1B OS=Homo sapiens GN=RAB1B PE=1 SV=1 - [RAB1B_HUMAN] | 3 | 13 | 16 | 201 | 22.2 | 5.73 |
| Q13509 | Tubulin beta-3 chain OS=Homo sapiens GN=TUBB3 PE=1 SV=2 - [TBB3_HUMAN] | 3 | 39 | 49 | 450 | 50.4 | 4.93 |
| P63172 | Dynein light chain Tctex-type 1 OS=Homo sapiens GN=DYNLT1 PE=1 SV=1 - [DYLT1_HUMAN] | 3 | 3 | 5 | 113 | 12.4 | 5.08 |
| P07108 | Acyl-CoA-binding protein OS=Homo sapiens GN=DBI PE=1 SV=2 - [ACBP_HUMAN] | 3 | 2 | 2 | 87 | 10.0 | 6.57 |
| Q9NR31 | GTP-binding protein SAR1a OS=Homo sapiens GN=SAR1A PE=1 SV=1 - [SAR1A_HUMAN] | 3 | 3 | 5 | 198 | 22.4 | 6.68 |
| P05386 | 60S acidic ribosomal protein P1 OS=Homo sapiens GN=RPLP1 PE=1 SV=1 - [RLA1_HUMAN] | 3 | 6 | 3 | 114 | 11.5 | 4.32 |
| P08708 | 40S ribosomal protein S17 OS=Homo sapiens GN=RPS17 PE=1 SV=2 - [RS17_HUMAN] | 3 | 7 | 7 | 135 | 15.5 | 9.85 |
| P41567 | Eukaryotic translation initiation factor 1 OS=Homo sapiens GN=EIF1 PE=1 SV=1 - [EIF1_HUMAN] | 3 | 2 | 3 | 113 | 12.7 | 7.44 |
| Q5RI15 | Cytochrome c oxidase protein 20 homolog OS=Homo sapiens GN=COX20 PE=1 SV=2 - [COX20_HUMAN] | 3 | 3 | 3 | 118 | 13.3 | 8.76 |
| P42677 | 40S ribosomal protein S27 OS=Homo sapiens GN=RPS27 PE=1 SV=3 - [RS27_HUMAN] | 3 | 2 | 3 | 84 | 9.5 | 9.45 |
| P98179 | Putative RNA-binding protein 3 OS=Homo sapiens GN=RBM3 PE=1 SV=1 - [RBM3_HUMAN] | 3 | 5 | 4 | 157 | 17.2 | 8.91 |
| P10599 | Thioredoxin OS=Homo sapiens GN=TXN PE=1 SV=3 - [THIO_HUMAN] | 3 | 1 | 3 | 105 | 11.7 | 4.92 |
| P63279 | SUMO-conjugating enzyme UBC9 OS=Homo sapiens GN=UBE2I PE=1 SV=1 - [UBC9_HUMAN] | 3 | 3 | 4 | 158 | 18.0 | 8.66 |
| P63173 | 60S ribosomal protein L38 OS=Homo sapiens GN=RPL38 PE=1 SV=2 - [RL38_HUMAN] | 3 | 3 | 4 | 70 | 8.2 | 10.10 |
| P25398 | 40S ribosomal protein S12 OS=Homo sapiens GN=RPS12 PE=1 SV=3 - [RS12_HUMAN] | 3 | 8 | 6 | 132 | 14.5 | 7.21 |
| O00762 | Ubiquitin-conjugating enzyme E2 C OS=Homo sapiens GN=UBE2C PE=1 SV=1 - [UBE2C_HUMAN] | 3 | 2 | 2 | 179 | 19.6 | 7.37 |
| P20290 | Transcription factor BTF3 OS=Homo sapiens GN=BTF3 PE=1 SV=1 - [BTF3_HUMAN] | 3 | 3 | 3 | 206 | 22.2 | 9.38 |
| P61006 | Ras-related protein Rab-8A OS=Homo sapiens GN=RAB8A PE=1 SV=1 - [RAB8A_HUMAN] | 3 | 9 | 7 | 207 | 23.7 | 9.07 |
| Q96AB3 | Isochorismatase domain-containing protein 2. mitochondrial OS=Homo sapiens GN=ISOC2 PE=1 SV=1 - [ISOC2_HUMAN] | 3 | 2 | 3 | 205 | 22.3 | 7.77 |
| P42167 | Lamina-associated polypeptide 2. isoforms beta/gamma OS=Homo sapiens GN=TMPO PE=1 SV=2 - [LAP2B_HUMAN] | 3 | 10 | 15 | 454 | 50.6 | 9.38 |
| P00441 | Superoxide dismutase [Cu-Zn] OS=Homo sapiens GN=SOD1 PE=1 SV=2 - [SODC_HUMAN] | 3 | 3 | 5 | 154 | 15.9 | 6.13 |
| Q9Y3E5 | Peptidyl-tRNA hydrolase 2. mitochondrial OS=Homo sapiens GN=PTRH2 PE=1 SV=1 - [PTH2_HUMAN] | 3 | 3 | 4 | 179 | 19.2 | 8.73 |
| P62318 | Small nuclear ribonucleoprotein Sm D3 OS=Homo sapiens GN=SNRPD3 PE=1 SV=1 - [SMD3_HUMAN] | 3 | 3 | 4 | 126 | 13.9 | 10.32 |
| P0C0S5 | Histone H2A.Z OS=Homo sapiens GN=H2AFZ PE=1 SV=2 - [H2AZ_HUMAN] | 3 | 7 | 7 | 128 | 13.5 | 10.58 |
| P31150 | Rab GDP dissociation inhibitor alpha OS=Homo sapiens GN=GDI1 PE=1 SV=2 - [GDIA_HUMAN] | 3 | 11 | 11 | 447 | 50.6 | 5.14 |
| O75431 | Metaxin-2 OS=Homo sapiens GN=MTX2 PE=1 SV=1 - [MTX2_HUMAN] | 3 | 3 | 3 | 263 | 29.7 | 6.29 |
| P61026 | Ras-related protein Rab-10 OS=Homo sapiens GN=RAB10 PE=1 SV=1 - [RAB10_HUMAN] | 3 | 8 | 7 | 200 | 22.5 | 8.38 |
| Q96DE0 | U8 snoRNA-decapping enzyme OS=Homo sapiens GN=NUDT16 PE=1 SV=2 - [NUD16_HUMAN] | 3 | 3 | 4 | 195 | 21.3 | 6.89 |
| P50120 | Retinol-binding protein 2 OS=Homo sapiens GN=RBP2 PE=1 SV=3 - [RET2_HUMAN] | 3 | 3 | 3 | 134 | 15.7 | 5.43 |
| P02792 | Ferritin light chain OS=Homo sapiens GN=FTL PE=1 SV=2 - [FRIL_HUMAN] | 3 | 3 | 5 | 175 | 20.0 | 5.78 |
| P41223 | Protein BUD31 homolog OS=Homo sapiens GN=BUD31 PE=1 SV=2 - [BUD31_HUMAN] | 3 | 1 | 3 | 144 | 17.0 | 8.82 |
| P20340 | Ras-related protein Rab-6A OS=Homo sapiens GN=RAB6A PE=1 SV=3 - [RAB6A_HUMAN] | 3 | 7 | 7 | 208 | 23.6 | 5.54 |
| Q9BX68 | Histidine triad nucleotide-binding protein 2. mitochondrial OS=Homo sapiens GN=HINT2 PE=1 SV=1 - [HINT2_HUMAN] | 3 | 3 | 3 | 163 | 17.2 | 9.16 |
| O00264 | Membrane-associated progesterone receptor component 1 OS=Homo sapiens GN=PGRMC1 PE=1 SV=3 - [PGRC1_HUMAN] | 3 | 3 | 2 | 195 | 21.7 | 4.70 |
| O43324 | Eukaryotic translation elongation factor 1 epsilon-1 OS=Homo sapiens GN=EEF1E1 PE=1 SV=1 - [MCA3_HUMAN] | 3 | 1 | 3 | 174 | 19.8 | 8.54 |
| P63208 | S-phase kinase-associated protein 1 OS=Homo sapiens GN=SKP1 PE=1 SV=2 - [SKP1_HUMAN] | 3 | 3 | 3 | 163 | 18.6 | 4.54 |
| Q14011 | Cold-inducible RNA-binding protein OS=Homo sapiens GN=CIRBP PE=1 SV=1 - [CIRBP_HUMAN] | 3 | 2 | 4 | 172 | 18.6 | 9.51 |
| Q9UHV9 | Prefoldin subunit 2 OS=Homo sapiens GN=PFDN2 PE=1 SV=1 - [PFD2_HUMAN] | 3 | 3 | 2 | 154 | 16.6 | 6.58 |
| P84103 | Splicing factor. arginine/serine-rich 3 OS=Homo sapiens GN=SFRS3 PE=1 SV=1 - [SFRS3_HUMAN] | 3 | 3 | 2 | 164 | 19.3 | 11.65 |
| O43399 | Tumor protein D54 OS=Homo sapiens GN=TPD52L2 PE=1 SV=2 - [TPD54_HUMAN] | 3 | 3 | 2 | 206 | 22.2 | 5.36 |
| Q8TEA8 | D-tyrosyl-tRNA(Tyr) deacylase 1 OS=Homo sapiens GN=DTD1 PE=1 SV=2 - [DTD1_HUMAN] | 3 | 2 | 3 | 209 | 23.4 | 8.24 |
| P60983 | Glia maturation factor beta OS=Homo sapiens GN=GMFB PE=1 SV=2 - [GMFB_HUMAN] | 3 | 2 | 3 | 142 | 16.7 | 5.29 |
| P60866 | 40S ribosomal protein S20 OS=Homo sapiens GN=RPS20 PE=1 SV=1 - [RS20_HUMAN] | 3 | 3 | 4 | 119 | 13.4 | 9.94 |
| Q9NPJ3 | Acyl-coenzyme A thioesterase 13 OS=Homo sapiens GN=ACOT13 PE=1 SV=1 - [ACO13_HUMAN] | 3 | 4 | 4 | 140 | 15.0 | 9.14 |
| Q8WW12 | PEST proteolytic signal-containing nuclear protein OS=Homo sapiens GN=PCNP PE=1 SV=2 - [PCNP_HUMAN] | 3 | 3 | 2 | 178 | 18.9 | 7.49 |
| P08579 | U2 small nuclear ribonucleoprotein B'' OS=Homo sapiens GN=SNRPB2 PE=1 SV=1 - [RU2B_HUMAN] | 3 | 3 | 5 | 225 | 25.5 | 9.72 |
| Q9GZT3 | SRA stem-loop-interacting RNA-binding protein. mitochondrial OS=Homo sapiens GN=SLIRP PE=1 SV=1 - [SLIRP_HUMAN] | 3 | 1 | 3 | 109 | 12.3 | 10.24 |
| P30043 | Flavin reductase OS=Homo sapiens GN=BLVRB PE=1 SV=3 - [BLVRB_HUMAN] | 3 | 3 | 3 | 206 | 22.1 | 7.65 |
| P13073 | Cytochrome c oxidase subunit 4 isoform 1. mitochondrial OS=Homo sapiens GN=COX4I1 PE=1 SV=1 - [COX41_HUMAN] | 3 | 2 | 3 | 169 | 19.6 | 9.51 |
| Q16576 | Histone-binding protein RBBP7 OS=Homo sapiens GN=RBBP7 PE=1 SV=1 - [RBBP7_HUMAN] | 3 | 5 | 7 | 425 | 47.8 | 5.05 |
| P16949 | Stathmin OS=Homo sapiens GN=STMN1 PE=1 SV=3 - [STMN1_HUMAN] | 3 | 3 | 4 | 149 | 17.3 | 5.97 |
| P19105 | Myosin regulatory light chain 12A OS=Homo sapiens GN=MYL12A PE=1 SV=2 - [ML12A_HUMAN] | 3 | 3 | 1 | 171 | 19.8 | 4.81 |
| Q15102 | Platelet-activating factor acetylhydrolase IB subunit gamma OS=Homo sapiens GN=PAFAH1B3 PE=1 SV=1 - [PA1B3_HUMAN] | 3 | 3 | 2 | 231 | 25.7 | 6.84 |
| P51148 | Ras-related protein Rab-5C OS=Homo sapiens GN=RAB5C PE=1 SV=2 - [RAB5C_HUMAN] | 3 | 6 | 5 | 216 | 23.5 | 8.41 |
| P62851 | 40S ribosomal protein S25 OS=Homo sapiens GN=RPS25 PE=1 SV=1 - [RS25_HUMAN] | 3 | 4 | 3 | 125 | 13.7 | 10.11 |
| P61758 | Prefoldin subunit 3 OS=Homo sapiens OX=9606 GN=VBP1 PE=1 SV=4 - [PFD3_HUMAN] | 3 |  | 3 | 197 | 22.6 | 7.11 |
| P60981 | Destrin OS=Homo sapiens GN=DSTN PE=1 SV=3 - [DEST_HUMAN] | 3 | 3 | 3 | 165 | 18.5 | 7.85 |
| P08754 | Guanine nucleotide-binding protein G(k) subunit alpha OS=Homo sapiens GN=GNAI3 PE=1 SV=3 - [GNAI3_HUMAN] | 3 | 7 | 6 | 354 | 40.5 | 5.69 |
| Q96H79 | Zinc finger CCCH-type antiviral protein 1-like OS=Homo sapiens GN=ZC3HAV1L PE=1 SV=2 - [ZCCHL_HUMAN] | 3 | 2 | 4 | 300 | 32.9 | 8.13 |
| Q93062 | RNA-binding protein with multiple splicing OS=Homo sapiens GN=RBPMS PE=1 SV=1 - [RBPMS_HUMAN] | 3 | 3 | 2 | 196 | 21.8 | 8.07 |
| P61163 | Alpha-centractin OS=Homo sapiens GN=ACTR1A PE=1 SV=1 - [ACTZ_HUMAN] | 3 | 6 | 4 | 376 | 42.6 | 6.64 |
| P67812 | Signal peptidase complex catalytic subunit SEC11A OS=Homo sapiens GN=SEC11A PE=1 SV=1 - [SC11A_HUMAN] | 3 | 2 | 3 | 179 | 20.6 | 9.48 |
| P46779 | 60S ribosomal protein L28 OS=Homo sapiens GN=RPL28 PE=1 SV=3 - [RL28_HUMAN] | 3 | 2 | 3 | 137 | 15.7 | 12.02 |
| O94905 | Erlin-2 OS=Homo sapiens GN=ERLIN2 PE=1 SV=1 - [ERLN2_HUMAN] | 3 | 4 | 5 | 339 | 37.8 | 5.62 |
| P61353 | 60S ribosomal protein L27 OS=Homo sapiens GN=RPL27 PE=1 SV=2 - [RL27_HUMAN] | 3 | 2 | 3 | 136 | 15.8 | 10.56 |
| P61081 | NEDD8-conjugating enzyme Ubc12 OS=Homo sapiens GN=UBE2M PE=1 SV=1 - [UBC12_HUMAN] | 3 | 2 | 2 | 183 | 20.9 | 7.69 |
| O14737 | Programmed cell death protein 5 OS=Homo sapiens GN=PDCD5 PE=1 SV=3 - [PDCD5_HUMAN] | 3 | 3 | 2 | 125 | 14.3 | 6.04 |
| P37108 | Signal recognition particle 14 kDa protein OS=Homo sapiens GN=SRP14 PE=1 SV=2 - [SRP14_HUMAN] | 3 | 3 | 4 | 136 | 14.6 | 10.04 |
| P21266 | Glutathione S-transferase Mu 3 OS=Homo sapiens GN=GSTM3 PE=1 SV=3 - [GSTM3_HUMAN] | 3 | 4 | 5 | 225 | 26.5 | 5.54 |
| Q13765 | Nascent polypeptide-associated complex subunit alpha OS=Homo sapiens GN=NACA PE=1 SV=1 - [NACA_HUMAN] | 3 | 4 | 5 | 215 | 23.4 | 4.56 |
| P46926 | Glucosamine-6-phosphate isomerase 1 OS=Homo sapiens GN=GNPDA1 PE=1 SV=1 - [GNPI1_HUMAN] | 3 | 1 | 3 | 289 | 32.6 | 6.92 |
| P10620 | Microsomal glutathione S-transferase 1 OS=Homo sapiens GN=MGST1 PE=1 SV=1 - [MGST1_HUMAN] | 3 | 3 | 4 | 155 | 17.6 | 9.39 |
| P10768 | S-formylglutathione hydrolase OS=Homo sapiens GN=ESD PE=1 SV=2 - [ESTD_HUMAN] | 3 | 1 | 2 | 282 | 31.4 | 7.02 |
| P36543 | V-type proton ATPase subunit E 1 OS=Homo sapiens GN=ATP6V1E1 PE=1 SV=1 - [VATE1_HUMAN] | 3 | 2 | 2 | 226 | 26.1 | 8.00 |
| P02753 | Retinol-binding protein 4 OS=Homo sapiens GN=RBP4 PE=1 SV=3 - [RET4_HUMAN] | 3 |  | 4 | 201 | 23.0 | 6.07 |
| P53999 | Activated RNA polymerase II transcriptional coactivator p15 OS=Homo sapiens GN=SUB1 PE=1 SV=3 - [TCP4_HUMAN] | 3 | 2 | 3 | 127 | 14.4 | 9.60 |
| P80217 | Interferon-induced 35 kDa protein OS=Homo sapiens GN=IFI35 PE=1 SV=5 - [IN35_HUMAN] | 3 | 2 | 2 | 286 | 31.5 | 6.09 |
| Q9Y3E0 | Vesicle transport protein GOT1B OS=Homo sapiens GN=GOLT1B PE=1 SV=1 - [GOT1B_HUMAN] | 3 | 5 | 3 | 138 | 15.4 | 10.36 |
| Q96CN7 | Isochorismatase domain-containing protein 1 OS=Homo sapiens GN=ISOC1 PE=1 SV=3 - [ISOC1_HUMAN] | 3 | 4 | 4 | 298 | 32.2 | 7.39 |
| Q15181 | Inorganic pyrophosphatase OS=Homo sapiens GN=PPA1 PE=1 SV=2 - [IPYR_HUMAN] | 3 | 3 | 5 | 289 | 32.6 | 5.86 |
| O43681 | ATPase ASNA1 OS=Homo sapiens GN=ASNA1 PE=1 SV=2 - [ASNA_HUMAN] | 3 | 1 | 4 | 348 | 38.8 | 4.91 |
| O96000 | NADH dehydrogenase [ubiquinone] 1 beta subcomplex subunit 10 OS=Homo sapiens GN=NDUFB10 PE=1 SV=3 - [NDUBA_HUMAN] | 3 | 3 | 3 | 172 | 20.8 | 8.48 |
| P82979 | SAP domain-containing ribonucleoprotein OS=Homo sapiens GN=SARNP PE=1 SV=3 - [SARNP_HUMAN] | 3 | 2 | 3 | 210 | 23.7 | 6.42 |
| Q9BV57 | 1.2-dihydroxy-3-keto-5-methylthiopentene dioxygenase OS=Homo sapiens GN=ADI1 PE=1 SV=1 - [MTND_HUMAN] | 3 | 1 | 3 | 179 | 21.5 | 5.68 |
| O15173 | Membrane-associated progesterone receptor component 2 OS=Homo sapiens GN=PGRMC2 PE=1 SV=1 - [PGRC2_HUMAN] | 3 | 2 | 3 | 223 | 23.8 | 4.88 |
| Q13151 | Heterogeneous nuclear ribonucleoprotein A0 OS=Homo sapiens GN=HNRNPA0 PE=1 SV=1 - [ROA0_HUMAN] | 3 | 4 | 5 | 305 | 30.8 | 9.29 |
| P62760 | Visinin-like protein 1 OS=Homo sapiens GN=VSNL1 PE=1 SV=2 - [VISL1_HUMAN] | 3 | 1 | 2 | 191 | 22.1 | 5.15 |
| Q14257 | Reticulocalbin-2 OS=Homo sapiens GN=RCN2 PE=1 SV=1 - [RCN2_HUMAN] | 3 |  | 3 | 317 | 36.9 | 4.40 |
| Q9Y2T3 | Guanine deaminase OS=Homo sapiens GN=GDA PE=1 SV=1 - [GUAD_HUMAN] | 3 | 2 | 3 | 454 | 51.0 | 5.68 |
| Q15785 | Mitochondrial import receptor subunit TOM34 OS=Homo sapiens GN=TOMM34 PE=1 SV=2 - [TOM34_HUMAN] | 3 | 2 | 1 | 309 | 34.5 | 8.98 |
| Q9BQE5 | Apolipoprotein L2 OS=Homo sapiens GN=APOL2 PE=1 SV=1 - [APOL2_HUMAN] | 3 | 3 |  | 337 | 37.1 | 6.74 |
| Q9BRK5 | 45 kDa calcium-binding protein OS=Homo sapiens GN=SDF4 PE=1 SV=1 - [CAB45_HUMAN] | 3 | 4 | 3 | 362 | 41.8 | 4.86 |
| Q92979 | Probable ribosome biogenesis protein NEP1 OS=Homo sapiens GN=EMG1 PE=1 SV=4 - [NEP1_HUMAN] | 3 | 3 | 1 | 244 | 26.7 | 9.17 |
| Q9BTT0 | Acidic leucine-rich nuclear phosphoprotein 32 family member E OS=Homo sapiens GN=ANP32E PE=1 SV=1 - [AN32E_HUMAN] | 3 | 2 | 3 | 268 | 30.7 | 3.85 |
| O15260 | Surfeit locus protein 4 OS=Homo sapiens GN=SURF4 PE=1 SV=3 - [SURF4_HUMAN] | 3 | 9 | 10 | 269 | 30.4 | 7.78 |
| Q9H0W9 | Ester hydrolase C11orf54 OS=Homo sapiens GN=C11orf54 PE=1 SV=1 - [CK054_HUMAN] | 3 | 3 | 2 | 315 | 35.1 | 6.70 |
| Q8NCW5 | Apolipoprotein A-I-binding protein OS=Homo sapiens GN=APOA1BP PE=1 SV=2 - [AIBP_HUMAN] | 3 | 1 | 3 | 288 | 31.7 | 7.66 |
| O75352 | Mannose-P-dolichol utilization defect 1 protein OS=Homo sapiens GN=MPDU1 PE=1 SV=2 - [MPU1_HUMAN] | 3 | 1 | 3 | 247 | 26.6 | 8.94 |
| P23919 | Thymidylate kinase OS=Homo sapiens GN=DTYMK PE=1 SV=4 - [KTHY_HUMAN] | 3 | 2 | 3 | 212 | 23.8 | 8.27 |
| P78330 | Phosphoserine phosphatase OS=Homo sapiens GN=PSPH PE=1 SV=2 - [SERB_HUMAN] | 3 | 3 | 3 | 225 | 25.0 | 5.69 |
| Q14558 | Phosphoribosyl pyrophosphate synthase-associated protein 1 OS=Homo sapiens GN=PRPSAP1 PE=1 SV=2 - [KPRA_HUMAN] | 3 | 2 | 3 | 356 | 39.4 | 7.20 |
| Q15125 | 3-beta-hydroxysteroid-Delta(8).Delta(7)-isomerase OS=Homo sapiens GN=EBP PE=1 SV=3 - [EBP_HUMAN] | 3 | 3 | 4 | 230 | 26.3 | 7.90 |
| P24534 | Elongation factor 1-beta OS=Homo sapiens GN=EEF1B2 PE=1 SV=3 - [EF1B_HUMAN] | 3 | 2 | 3 | 225 | 24.7 | 4.67 |
| P09417 | Dihydropteridine reductase OS=Homo sapiens GN=QDPR PE=1 SV=2 - [DHPR_HUMAN] | 3 | 4 | 5 | 244 | 25.8 | 7.37 |
| O75569 | Interferon-inducible double stranded RNA-dependent protein kinase activator A OS=Homo sapiens GN=PRKRA PE=1 SV=1 - [PRKRA_HUMAN] | 3 | 4 |  | 313 | 34.4 | 8.41 |
| P50914 | 60S ribosomal protein L14 OS=Homo sapiens GN=RPL14 PE=1 SV=4 - [RL14_HUMAN] | 3 | 4 | 4 | 215 | 23.4 | 10.93 |
| Q9Y6E2 | Basic leucine zipper and W2 domain-containing protein 2 OS=Homo sapiens GN=BZW2 PE=1 SV=1 - [BZW2_HUMAN] | 3 | 3 | 4 | 419 | 48.1 | 6.68 |
| Q99439 | Calponin-2 OS=Homo sapiens GN=CNN2 PE=1 SV=4 - [CNN2_HUMAN] | 3 | 3 | 3 | 309 | 33.7 | 7.33 |
| P00492 | Hypoxanthine-guanine phosphoribosyltransferase OS=Homo sapiens GN=HPRT1 PE=1 SV=2 - [HPRT_HUMAN] | 3 | 2 | 3 | 218 | 24.6 | 6.68 |
| Q9BTT6 | Leucine-rich repeat-containing protein 1 OS=Homo sapiens GN=LRRC1 PE=1 SV=1 - [LRRC1_HUMAN] | 3 | 3 | 3 | 524 | 59.2 | 5.02 |
| P61160 | Actin-related protein 2 OS=Homo sapiens GN=ACTR2 PE=1 SV=1 - [ARP2_HUMAN] | 3 | 4 | 2 | 394 | 44.7 | 6.74 |
| Q99447 | Ethanolamine-phosphate cytidylyltransferase OS=Homo sapiens GN=PCYT2 PE=1 SV=1 - [PCY2_HUMAN] | 3 | 1 | 3 | 389 | 43.8 | 6.92 |
| P46109 | Crk-like protein OS=Homo sapiens GN=CRKL PE=1 SV=1 - [CRKL_HUMAN] | 3 | 3 | 3 | 303 | 33.8 | 6.74 |
| Q6IBS0 | Twinfilin-2 OS=Homo sapiens GN=TWF2 PE=1 SV=2 - [TWF2_HUMAN] | 3 | 1 | 4 | 349 | 39.5 | 6.84 |
| Q96HR9 | Receptor expression-enhancing protein 6 OS=Homo sapiens OX=9606 GN=REEP6 PE=1 SV=2 - [REEP6_HUMAN] | 3 | 7 | 6 | 211 | 23.4 | 8.56 |
| Q9H444 | Charged multivesicular body protein 4b OS=Homo sapiens GN=CHMP4B PE=1 SV=1 - [CHM4B_HUMAN] | 3 | 3 | 2 | 224 | 24.9 | 4.82 |
| P48739 | Phosphatidylinositol transfer protein beta isoform OS=Homo sapiens GN=PITPNB PE=1 SV=2 - [PIPNB_HUMAN] | 3 | 2 | 3 | 271 | 31.5 | 6.87 |
| Q9UM00 | Calcium load-activated calcium channel OS=Homo sapiens OX=9606 GN=TMCO1 PE=1 SV=2 - [TMCO1_HUMAN] | 3 | 2 | 3 | 239 | 27.1 | 10.26 |
| P27635 | 60S ribosomal protein L10 OS=Homo sapiens GN=RPL10 PE=1 SV=4 - [RL10_HUMAN] | 3 | 2 | 4 | 214 | 24.6 | 10.08 |
| P40937 | Replication factor C subunit 5 OS=Homo sapiens GN=RFC5 PE=1 SV=1 - [RFC5_HUMAN] | 3 | 3 | 3 | 340 | 38.5 | 7.20 |
| O60762 | Dolichol-phosphate mannosyltransferase OS=Homo sapiens GN=DPM1 PE=1 SV=1 - [DPM1_HUMAN] | 3 | 5 | 3 | 260 | 29.6 | 9.57 |
| P22061 | Protein-L-isoaspartate(D-aspartate) O-methyltransferase OS=Homo sapiens GN=PCMT1 PE=1 SV=4 - [PIMT_HUMAN] | 3 | 1 | 2 | 227 | 24.6 | 7.21 |
| Q9HAT2 | Sialate O-acetylesterase OS=Homo sapiens GN=SIAE PE=1 SV=1 - [SIAE_HUMAN] | 3 | 2 | 2 | 523 | 58.3 | 7.33 |
| P19623 | Spermidine synthase OS=Homo sapiens GN=SRM PE=1 SV=1 - [SPEE_HUMAN] | 3 | 3 | 2 | 302 | 33.8 | 5.49 |
| Q16762 | Thiosulfate sulfurtransferase OS=Homo sapiens GN=TST PE=1 SV=4 - [THTR_HUMAN] | 3 | 3 | 1 | 297 | 33.4 | 7.25 |
| Q9HAV7 | GrpE protein homolog 1. mitochondrial OS=Homo sapiens GN=GRPEL1 PE=1 SV=2 - [GRPE1_HUMAN] | 3 | 2 | 3 | 217 | 24.3 | 8.12 |
| P06730 | Eukaryotic translation initiation factor 4E OS=Homo sapiens GN=EIF4E PE=1 SV=2 - [IF4E_HUMAN] | 3 | 2 | 2 | 217 | 25.1 | 6.15 |
| Q92600 | Cell differentiation protein RCD1 homolog OS=Homo sapiens GN=RQCD1 PE=1 SV=1 - [RCD1_HUMAN] | 3 | 2 | 3 | 299 | 33.6 | 8.03 |
| Q96C36 | Pyrroline-5-carboxylate reductase 2 OS=Homo sapiens GN=PYCR2 PE=1 SV=1 - [P5CR2_HUMAN] | 3 | 3 | 3 | 320 | 33.6 | 7.77 |
| Q9NZL4 | Hsp70-binding protein 1 OS=Homo sapiens OX=9606 GN=HSPBP1 PE=1 SV=2 - [HPBP1_HUMAN] | 3 | 2 | 2 | 359 | 39.3 | 5.21 |
| Q8TC12 | Retinol dehydrogenase 11 OS=Homo sapiens GN=RDH11 PE=1 SV=2 - [RDH11_HUMAN] | 3 | 2 | 3 | 318 | 35.4 | 8.82 |
| A6NDG6 | Phosphoglycolate phosphatase OS=Homo sapiens GN=PGP PE=1 SV=1 - [PGP_HUMAN] | 3 | 2 | 4 | 321 | 34.0 | 6.14 |
| O00505 | Importin subunit alpha-3 OS=Homo sapiens GN=KPNA3 PE=1 SV=2 - [IMA3_HUMAN] | 3 | 3 | 3 | 521 | 57.8 | 4.94 |
| O14908 | PDZ domain-containing protein GIPC1 OS=Homo sapiens GN=GIPC1 PE=1 SV=2 - [GIPC1_HUMAN] | 3 | 1 | 3 | 333 | 36.0 | 6.28 |
| Q9BRX8 | Redox-regulatory protein FAM213A OS=Homo sapiens GN=FAM213A PE=1 SV=3 - [F213A_HUMAN] | 3 | 2 | 3 | 229 | 25.7 | 8.84 |
| P62995 | Transformer-2 protein homolog beta OS=Homo sapiens GN=TRA2B PE=1 SV=1 - [TRA2B_HUMAN] | 3 | 3 | 2 | 288 | 33.6 | 11.25 |
| O00487 | 26S proteasome non-ATPase regulatory subunit 14 OS=Homo sapiens GN=PSMD14 PE=1 SV=1 - [PSDE_HUMAN] | 3 | 4 | 4 | 310 | 34.6 | 6.52 |
| Q9NP79 | Vacuolar protein sorting-associated protein VTA1 homolog OS=Homo sapiens GN=VTA1 PE=1 SV=1 - [VTA1_HUMAN] | 3 | 3 | 3 | 307 | 33.9 | 6.29 |
| P19404 | NADH dehydrogenase [ubiquinone] flavoprotein 2. mitochondrial OS=Homo sapiens GN=NDUFV2 PE=1 SV=2 - [NDUV2_HUMAN] | 3 | 3 | 3 | 249 | 27.4 | 8.06 |
| Q13242 | Splicing factor. arginine/serine-rich 9 OS=Homo sapiens GN=SFRS9 PE=1 SV=1 - [SFRS9_HUMAN] | 3 |  | 3 | 221 | 25.5 | 8.65 |
| O00233 | 26S proteasome non-ATPase regulatory subunit 9 OS=Homo sapiens GN=PSMD9 PE=1 SV=3 - [PSMD9_HUMAN] | 3 |  | 3 | 223 | 24.7 | 6.95 |
| O60547 | GDP-mannose 4.6 dehydratase OS=Homo sapiens GN=GMDS PE=1 SV=1 - [GMDS_HUMAN] | 3 | 1 | 3 | 372 | 41.9 | 7.31 |
| P17612 | cAMP-dependent protein kinase catalytic subunit alpha OS=Homo sapiens GN=PRKACA PE=1 SV=2 - [KAPCA_HUMAN] | 3 | 1 | 3 | 351 | 40.6 | 8.79 |
| Q9H2V7 | Protein spinster homolog 1 OS=Homo sapiens GN=SPNS1 PE=1 SV=1 - [SPNS1_HUMAN] | 3 | 1 | 2 | 528 | 56.6 | 6.64 |
| P54920 | Alpha-soluble NSF attachment protein OS=Homo sapiens GN=NAPA PE=1 SV=3 - [SNAA_HUMAN] | 3 | 2 | 3 | 295 | 33.2 | 5.36 |
| O75822 | Eukaryotic translation initiation factor 3 subunit J OS=Homo sapiens GN=EIF3J PE=1 SV=2 - [EIF3J_HUMAN] | 3 | 2 | 3 | 258 | 29.0 | 4.83 |
| Q9BQA1 | Methylosome protein 50 OS=Homo sapiens GN=WDR77 PE=1 SV=1 - [MEP50_HUMAN] | 3 | 2 | 2 | 342 | 36.7 | 5.17 |
| Q96N66 | Lysophospholipid acyltransferase 7 OS=Homo sapiens GN=MBOAT7 PE=1 SV=2 - [MBOA7_HUMAN] | 3 | 2 | 2 | 472 | 52.7 | 8.97 |
| Q96S66 | Chloride channel CLIC-like protein 1 OS=Homo sapiens GN=CLCC1 PE=1 SV=1 - [CLCC1_HUMAN] | 3 | 1 | 3 | 551 | 62.0 | 5.55 |
| P43034 | Platelet-activating factor acetylhydrolase IB subunit alpha OS=Homo sapiens GN=PAFAH1B1 PE=1 SV=2 - [LIS1_HUMAN] | 3 | 5 | 2 | 410 | 46.6 | 7.37 |
| Q9UK22 | F-box only protein 2 OS=Homo sapiens GN=FBXO2 PE=1 SV=2 - [FBX2_HUMAN] | 3 | 2 | 3 | 296 | 33.3 | 4.37 |
| Q9BRF8 | Serine/threonine-protein phosphatase CPPED1 OS=Homo sapiens GN=CPPED1 PE=1 SV=3 - [CPPED_HUMAN] | 3 | 3 | 1 | 314 | 35.5 | 6.20 |
| Q13510 | Acid ceramidase OS=Homo sapiens GN=ASAH1 PE=1 SV=5 - [ASAH1_HUMAN] | 3 | 1 | 3 | 395 | 44.6 | 7.62 |
| Q9UGK3 | Signal-transducing adaptor protein 2 OS=Homo sapiens GN=STAP2 PE=1 SV=2 - [STAP2_HUMAN] | 3 | 1 | 3 | 403 | 44.9 | 8.16 |
| P39748 | Flap endonuclease 1 OS=Homo sapiens GN=FEN1 PE=1 SV=1 - [FEN1_HUMAN] | 3 | 3 | 4 | 380 | 42.6 | 8.62 |
| Q9BT78 | COP9 signalosome complex subunit 4 OS=Homo sapiens GN=COPS4 PE=1 SV=1 - [CSN4_HUMAN] | 3 | 4 | 4 | 406 | 46.2 | 5.83 |
| Q13867 | Bleomycin hydrolase OS=Homo sapiens GN=BLMH PE=1 SV=1 - [BLMH_HUMAN] | 3 | 2 | 3 | 455 | 52.5 | 6.27 |
| Q13309 | S-phase kinase-associated protein 2 OS=Homo sapiens GN=SKP2 PE=1 SV=2 - [SKP2_HUMAN] | 3 | 3 | 3 | 424 | 47.7 | 7.11 |
| Q9NYL9 | Tropomodulin-3 OS=Homo sapiens GN=TMOD3 PE=1 SV=1 - [TMOD3_HUMAN] | 3 | 3 | 4 | 352 | 39.6 | 5.19 |
| O95232 | Luc7-like protein 3 OS=Homo sapiens GN=LUC7L3 PE=1 SV=2 - [LC7L3_HUMAN] | 3 | 3 | 3 | 432 | 51.4 | 9.79 |
| Q9NUI1 | Peroxisomal 2.4-dienoyl-CoA reductase OS=Homo sapiens GN=DECR2 PE=1 SV=1 - [DECR2_HUMAN] | 3 | 1 | 3 | 292 | 30.8 | 9.22 |
| P17050 | Alpha-N-acetylgalactosaminidase OS=Homo sapiens GN=NAGA PE=1 SV=2 - [NAGAB_HUMAN] | 3 |  | 3 | 411 | 46.5 | 5.19 |
| Q96HD9 | N-acyl-aromatic-L-amino acid amidohydrolase (carboxylate-forming) OS=Homo sapiens OX=9606 GN=ACY3 PE=1 SV=1 - [ACY3_HUMAN] | 3 | 1 | 2 | 319 | 35.2 | 5.90 |
| P49841 | Glycogen synthase kinase-3 beta OS=Homo sapiens GN=GSK3B PE=1 SV=2 - [GSK3B_HUMAN] | 3 | 4 |  | 420 | 46.7 | 8.78 |
| Q9NX63 | Coiled-coil-helix-coiled-coil-helix domain-containing protein 3. mitochondrial OS=Homo sapiens GN=CHCHD3 PE=1 SV=1 - [CHCH3_HUMAN] | 3 | 2 | 3 | 227 | 26.1 | 8.28 |
| P51116 | Fragile X mental retardation syndrome-related protein 2 OS=Homo sapiens GN=FXR2 PE=1 SV=2 - [FXR2_HUMAN] | 3 | 2 | 5 | 673 | 74.2 | 6.23 |
| P49354 | Protein farnesyltransferase/geranylgeranyltransferase type-1 subunit alpha OS=Homo sapiens GN=FNTA PE=1 SV=1 - [FNTA_HUMAN] | 3 | 1 | 3 | 379 | 44.4 | 5.08 |
| Q9NVD7 | Alpha-parvin OS=Homo sapiens GN=PARVA PE=1 SV=1 - [PARVA_HUMAN] | 3 | 3 | 2 | 372 | 42.2 | 5.95 |
| Q12907 | Vesicular integral-membrane protein VIP36 OS=Homo sapiens GN=LMAN2 PE=1 SV=1 - [LMAN2_HUMAN] | 3 | 2 | 2 | 356 | 40.2 | 6.95 |
| P21397 | Amine oxidase [flavin-containing] A OS=Homo sapiens GN=MAOA PE=1 SV=1 - [AOFA_HUMAN] | 3 | 4 | 5 | 527 | 59.6 | 7.85 |
| P57088 | Transmembrane protein 33 OS=Homo sapiens GN=TMEM33 PE=1 SV=2 - [TMM33_HUMAN] | 3 | 2 | 3 | 247 | 28.0 | 9.70 |
| P19784 | Casein kinase II subunit alpha' OS=Homo sapiens GN=CSNK2A2 PE=1 SV=1 - [CSK22_HUMAN] | 3 | 3 | 1 | 350 | 41.2 | 8.56 |
| Q70UQ0 | Inhibitor of nuclear factor kappa-B kinase-interacting protein OS=Homo sapiens GN=IKIP PE=1 SV=1 - [IKIP_HUMAN] | 3 | 2 | 3 | 350 | 39.3 | 9.17 |
| Q96IJ6 | Mannose-1-phosphate guanyltransferase alpha OS=Homo sapiens GN=GMPPA PE=1 SV=1 - [GMPPA_HUMAN] | 3 | 2 | 2 | 420 | 46.3 | 7.21 |
| Q15717 | ELAV-like protein 1 OS=Homo sapiens GN=ELAVL1 PE=1 SV=2 - [ELAV1_HUMAN] | 3 | 2 | 3 | 326 | 36.1 | 9.17 |
| P48728 | Aminomethyltransferase. mitochondrial OS=Homo sapiens OX=9606 GN=AMT PE=1 SV=1 - [GCST_HUMAN] | 3 | 2 | 3 | 403 | 43.9 | 8.57 |
| Q9ULX3 | RNA-binding protein NOB1 OS=Homo sapiens GN=NOB1 PE=1 SV=1 - [NOB1_HUMAN] | 3 | 2 | 2 | 412 | 46.6 | 7.18 |
| P53602 | Diphosphomevalonate decarboxylase OS=Homo sapiens GN=MVD PE=1 SV=1 - [MVD1_HUMAN] | 3 | 3 | 1 | 400 | 43.4 | 7.23 |
| P08397 | Porphobilinogen deaminase OS=Homo sapiens GN=HMBS PE=1 SV=2 - [HEM3_HUMAN] | 3 | 5 | 3 | 361 | 39.3 | 7.18 |
| P0C7P4 | Cytochrome b-c1 complex subunit Rieske-like protein 1 OS=Homo sapiens GN=UQCRFSL1 PE=2 SV=1 - [UCRIL_HUMAN] | 3 | 1 | 3 | 283 | 30.8 | 8.87 |
| Q5JVF3 | PCI domain-containing protein 2 OS=Homo sapiens GN=PCID2 PE=1 SV=2 - [PCID2_HUMAN] | 3 | 3 | 3 | 399 | 46.0 | 8.53 |
| Q01081 | Splicing factor U2AF 35 kDa subunit OS=Homo sapiens GN=U2AF1 PE=1 SV=3 - [U2AF1_HUMAN] | 3 | 4 | 4 | 240 | 27.9 | 8.81 |
| P40938 | Replication factor C subunit 3 OS=Homo sapiens GN=RFC3 PE=1 SV=2 - [RFC3_HUMAN] | 3 | 3 | 2 | 356 | 40.5 | 8.34 |
| P50224 | Sulfotransferase 1A3/1A4 OS=Homo sapiens GN=SULT1A3 PE=1 SV=1 - [ST1A3_HUMAN] | 3 | 3 | 3 | 295 | 34.2 | 6.01 |
| P13796 | Plastin-2 OS=Homo sapiens GN=LCP1 PE=1 SV=6 - [PLSL_HUMAN] | 3 | 7 | 7 | 627 | 70.2 | 5.43 |
| P29083 | General transcription factor IIE subunit 1 OS=Homo sapiens GN=GTF2E1 PE=1 SV=2 - [T2EA_HUMAN] | 3 | 3 | 3 | 439 | 49.4 | 4.82 |
| Q08752 | Peptidyl-prolyl cis-trans isomerase D OS=Homo sapiens GN=PPID PE=1 SV=3 - [PPID_HUMAN] | 3 | 3 | 2 | 370 | 40.7 | 7.21 |
| Q9H8Y8 | Golgi reassembly-stacking protein 2 OS=Homo sapiens GN=GORASP2 PE=1 SV=3 - [GORS2_HUMAN] | 3 | 3 | 3 | 452 | 47.1 | 4.82 |
| Q3ZCQ8 | Mitochondrial import inner membrane translocase subunit TIM50 OS=Homo sapiens GN=TIMM50 PE=1 SV=2 - [TIM50_HUMAN] | 3 | 3 | 2 | 353 | 39.6 | 8.37 |
| P31937 | 3-hydroxyisobutyrate dehydrogenase. mitochondrial OS=Homo sapiens GN=HIBADH PE=1 SV=2 - [3HIDH_HUMAN] | 3 | 3 | 2 | 336 | 35.3 | 8.13 |
| Q96HS1 | Serine/threonine-protein phosphatase PGAM5. mitochondrial OS=Homo sapiens GN=PGAM5 PE=1 SV=2 - [PGAM5_HUMAN] | 3 | 1 | 3 | 289 | 32.0 | 8.68 |
| Q6P1A2 | Lysophospholipid acyltransferase 5 OS=Homo sapiens GN=LPCAT3 PE=1 SV=1 - [MBOA5_HUMAN] | 3 | 2 | 3 | 487 | 56.0 | 8.69 |
| Q92552 | 28S ribosomal protein S27. mitochondrial OS=Homo sapiens GN=MRPS27 PE=1 SV=3 - [RT27_HUMAN] | 3 | 1 | 3 | 414 | 47.6 | 6.18 |
| Q16539 | Mitogen-activated protein kinase 14 OS=Homo sapiens GN=MAPK14 PE=1 SV=3 - [MK14_HUMAN] | 3 | 3 | 2 | 360 | 41.3 | 5.78 |
| Q9Y6G9 | Cytoplasmic dynein 1 light intermediate chain 1 OS=Homo sapiens GN=DYNC1LI1 PE=1 SV=3 - [DC1L1_HUMAN] | 3 | 3 | 4 | 523 | 56.5 | 6.42 |
| P18754 | Regulator of chromosome condensation OS=Homo sapiens GN=RCC1 PE=1 SV=1 - [RCC1_HUMAN] | 3 | 4 | 2 | 421 | 44.9 | 7.52 |
| P05121 | Plasminogen activator inhibitor 1 OS=Homo sapiens GN=SERPINE1 PE=1 SV=1 - [PAI1_HUMAN] | 3 | 3 | 1 | 402 | 45.0 | 7.20 |
| Q96M27 | Protein PRRC1 OS=Homo sapiens GN=PRRC1 PE=1 SV=1 - [PRRC1_HUMAN] | 3 | 3 | 4 | 445 | 46.7 | 5.83 |
| Q16537 | Serine/threonine-protein phosphatase 2A 56 kDa regulatory subunit epsilon isoform OS=Homo sapiens GN=PPP2R5E PE=1 SV=1 - [2A5E_HUMAN] | 3 | 3 | 2 | 467 | 54.7 | 6.95 |
| Q8IUR7 | Armadillo repeat-containing protein 8 OS=Homo sapiens GN=ARMC8 PE=1 SV=2 - [ARMC8_HUMAN] | 3 | 3 | 2 | 673 | 75.5 | 6.73 |
| Q9H4M9 | EH domain-containing protein 1 OS=Homo sapiens GN=EHD1 PE=1 SV=2 - [EHD1_HUMAN] | 3 | 2 | 2 | 534 | 60.6 | 6.83 |
| Q9UEW8 | STE20/SPS1-related proline-alanine-rich protein kinase OS=Homo sapiens GN=STK39 PE=1 SV=3 - [STK39_HUMAN] | 3 | 2 | 3 | 545 | 59.4 | 6.29 |
| O43837 | Isocitrate dehydrogenase [NAD] subunit beta. mitochondrial OS=Homo sapiens GN=IDH3B PE=1 SV=2 - [IDH3B_HUMAN] | 3 | 3 | 2 | 385 | 42.2 | 8.46 |
| Q14254 | Flotillin-2 OS=Homo sapiens GN=FLOT2 PE=1 SV=2 - [FLOT2_HUMAN] | 3 | 3 | 3 | 428 | 47.0 | 5.25 |
| Q9Y512 | Sorting and assembly machinery component 50 homolog OS=Homo sapiens GN=SAMM50 PE=1 SV=3 - [SAM50_HUMAN] | 3 | 3 | 2 | 469 | 51.9 | 6.90 |
| P11177 | Pyruvate dehydrogenase E1 component subunit beta. mitochondrial OS=Homo sapiens GN=PDHB PE=1 SV=3 - [ODPB_HUMAN] | 3 | 3 | 2 | 359 | 39.2 | 6.65 |
| P04181 | Ornithine aminotransferase. mitochondrial OS=Homo sapiens GN=OAT PE=1 SV=1 - [OAT_HUMAN] | 3 | 1 | 4 | 439 | 48.5 | 7.03 |
| Q5TDH0 | Protein DDI1 homolog 2 OS=Homo sapiens GN=DDI2 PE=1 SV=1 - [DDI2_HUMAN] | 3 | 1 | 3 | 399 | 44.5 | 5.05 |
| P50995 | Annexin A11 OS=Homo sapiens GN=ANXA11 PE=1 SV=1 - [ANX11_HUMAN] | 3 | 3 | 4 | 505 | 54.4 | 7.65 |
| Q9BVC6 | Transmembrane protein 109 OS=Homo sapiens GN=TMEM109 PE=1 SV=1 - [TM109_HUMAN] | 3 | 2 | 3 | 243 | 26.2 | 10.48 |
| Q9UBF2 | Coatomer subunit gamma-2 OS=Homo sapiens GN=COPG2 PE=1 SV=1 - [COPG2_HUMAN] | 3 | 6 | 6 | 871 | 97.6 | 5.81 |
| Q9HD26 | Golgi-associated PDZ and coiled-coil motif-containing protein OS=Homo sapiens GN=GOPC PE=1 SV=1 - [GOPC_HUMAN] | 3 | 3 |  | 462 | 50.5 | 5.92 |
| Q9UJU6 | Drebrin-like protein OS=Homo sapiens GN=DBNL PE=1 SV=1 - [DBNL_HUMAN] | 3 | 3 | 3 | 430 | 48.2 | 5.05 |
| Q9UI12 | V-type proton ATPase subunit H OS=Homo sapiens GN=ATP6V1H PE=1 SV=1 - [VATH_HUMAN] | 3 | 3 | 2 | 483 | 55.8 | 6.48 |
| Q9UBB4 | Ataxin-10 OS=Homo sapiens GN=ATXN10 PE=1 SV=1 - [ATX10_HUMAN] | 3 | 1 | 4 | 475 | 53.5 | 5.25 |
| Q15642 | Cdc42-interacting protein 4 OS=Homo sapiens GN=TRIP10 PE=1 SV=3 - [CIP4_HUMAN] | 3 | 3 | 2 | 601 | 68.3 | 5.73 |
| Q9UHR4 | Brain-specific angiogenesis inhibitor 1-associated protein 2-like protein 1 OS=Homo sapiens GN=BAIAP2L1 PE=1 SV=2 - [BI2L1_HUMAN] | 3 | 3 | 3 | 511 | 56.8 | 8.68 |
| O43493 | Trans-Golgi network integral membrane protein 2 OS=Homo sapiens OX=9606 GN=TGOLN2 PE=1 SV=3 - [TGON2_HUMAN] | 3 | 3 | 3 | 479 | 51.0 | 5.73 |
| Q6NUK1 | Calcium-binding mitochondrial carrier protein SCaMC-1 OS=Homo sapiens GN=SLC25A24 PE=1 SV=2 - [SCMC1_HUMAN] | 3 | 3 | 4 | 477 | 53.3 | 6.33 |
| P52594 | Arf-GAP domain and FG repeats-containing protein 1 OS=Homo sapiens GN=AGFG1 PE=1 SV=2 - [AGFG1_HUMAN] | 3 | 2 | 3 | 562 | 58.2 | 8.63 |
| P35659 | Protein DEK OS=Homo sapiens GN=DEK PE=1 SV=1 - [DEK_HUMAN] | 3 | 2 | 2 | 375 | 42.6 | 8.56 |
| Q9UN86 | Ras GTPase-activating protein-binding protein 2 OS=Homo sapiens GN=G3BP2 PE=1 SV=2 - [G3BP2_HUMAN] | 3 | 3 | 4 | 482 | 54.1 | 5.55 |
| Q9UKF6 | Cleavage and polyadenylation specificity factor subunit 3 OS=Homo sapiens GN=CPSF3 PE=1 SV=1 - [CPSF3_HUMAN] | 3 | 3 | 2 | 684 | 77.4 | 5.60 |
| Q14554 | Protein disulfide-isomerase A5 OS=Homo sapiens GN=PDIA5 PE=1 SV=1 - [PDIA5_HUMAN] | 3 | 2 | 2 | 519 | 59.6 | 7.91 |
| Q9NUD5 | Zinc finger CCHC domain-containing protein 3 OS=Homo sapiens OX=9606 GN=ZCCHC3 PE=1 SV=2 - [ZCHC3_HUMAN] | 3 | 2 | 3 | 403 | 43.5 | 8.53 |
| Q9C0E8 | Protein lunapark OS=Homo sapiens GN=LNP PE=1 SV=2 - [LNP_HUMAN] | 3 | 2 | 3 | 428 | 47.7 | 5.11 |
| P04004 | Vitronectin OS=Homo sapiens GN=VTN PE=1 SV=1 - [VTNC_HUMAN] | 3 | 2 | 3 | 478 | 54.3 | 5.80 |
| O14787 | Transportin-2 OS=Homo sapiens GN=TNPO2 PE=1 SV=3 - [TNPO2_HUMAN] | 3 | 3 | 5 | 897 | 101.3 | 5.01 |
| Q9NUQ8 | ATP-binding cassette sub-family F member 3 OS=Homo sapiens GN=ABCF3 PE=1 SV=2 - [ABCF3_HUMAN] | 3 | 2 | 2 | 709 | 79.7 | 6.34 |
| Q14739 | Lamin-B receptor OS=Homo sapiens GN=LBR PE=1 SV=2 - [LBR_HUMAN] | 3 | 1 | 2 | 615 | 70.7 | 9.36 |
| Q9UFC0 | Leucine-rich repeat and WD repeat-containing protein 1 OS=Homo sapiens GN=LRWD1 PE=1 SV=2 - [LRWD1_HUMAN] | 3 | 3 | 3 | 647 | 70.8 | 7.21 |
| P63151 | Serine/threonine-protein phosphatase 2A 55 kDa regulatory subunit B alpha isoform OS=Homo sapiens GN=PPP2R2A PE=1 SV=1 - [2ABA_HUMAN] | 3 | 4 | 3 | 447 | 51.7 | 6.20 |
| Q9HB07 | UPF0160 protein MYG1. mitochondrial OS=Homo sapiens GN=C12orf10 PE=1 SV=2 - [MYG1_HUMAN] | 3 | 1 | 3 | 376 | 42.4 | 6.67 |
| P08559 | Pyruvate dehydrogenase E1 component subunit alpha. somatic form. mitochondrial OS=Homo sapiens GN=PDHA1 PE=1 SV=3 - [ODPA_HUMAN] | 3 | 1 | 3 | 390 | 43.3 | 8.06 |
| Q13416 | Origin recognition complex subunit 2 OS=Homo sapiens GN=ORC2L PE=1 SV=2 - [ORC2_HUMAN] | 3 | 2 | 2 | 577 | 65.9 | 6.51 |
| Q13895 | Bystin OS=Homo sapiens GN=BYSL PE=1 SV=3 - [BYST_HUMAN] | 3 | 2 | 3 | 437 | 49.6 | 8.12 |
| Q9HCM4 | Band 4.1-like protein 5 OS=Homo sapiens GN=EPB41L5 PE=1 SV=3 - [E41L5_HUMAN] | 3 | 3 | 1 | 733 | 81.8 | 6.58 |
| O95470 | Sphingosine-1-phosphate lyase 1 OS=Homo sapiens GN=SGPL1 PE=1 SV=3 - [SGPL1_HUMAN] | 3 | 2 | 3 | 568 | 63.5 | 9.16 |
| Q96AC1 | Fermitin family homolog 2 OS=Homo sapiens GN=FERMT2 PE=1 SV=1 - [FERM2_HUMAN] | 3 | 3 | 2 | 680 | 77.8 | 6.70 |
| Q13033 | Striatin-3 OS=Homo sapiens GN=STRN3 PE=1 SV=3 - [STRN3_HUMAN] | 3 | 2 | 3 | 797 | 87.2 | 5.36 |
| P08243 | Asparagine synthetase [glutamine-hydrolyzing] OS=Homo sapiens GN=ASNS PE=1 SV=4 - [ASNS_HUMAN] | 3 | 4 | 3 | 561 | 64.3 | 6.86 |
| Q9UJX5 | Anaphase-promoting complex subunit 4 OS=Homo sapiens GN=ANAPC4 PE=1 SV=2 - [APC4_HUMAN] | 3 | 2 | 2 | 808 | 92.1 | 5.53 |
| Q9NUQ6 | SPATS2-like protein OS=Homo sapiens GN=SPATS2L PE=1 SV=2 - [SPS2L_HUMAN] | 3 | 3 | 3 | 558 | 61.7 | 9.64 |
| Q9UNS2 | COP9 signalosome complex subunit 3 OS=Homo sapiens GN=COPS3 PE=1 SV=3 - [CSN3_HUMAN] | 3 | 3 | 2 | 423 | 47.8 | 6.65 |
| Q9BV44 | THUMP domain-containing protein 3 OS=Homo sapiens GN=THUMPD3 PE=1 SV=1 - [THUM3_HUMAN] | 3 | 2 | 4 | 507 | 57.0 | 6.37 |
| P19823 | Inter-alpha-trypsin inhibitor heavy chain H2 OS=Homo sapiens GN=ITIH2 PE=1 SV=2 - [ITIH2_HUMAN] | 3 | 3 | 2 | 946 | 106.4 | 6.86 |
| P16278 | Beta-galactosidase OS=Homo sapiens GN=GLB1 PE=1 SV=2 - [BGAL_HUMAN] | 3 | 1 | 3 | 677 | 76.0 | 6.57 |
| Q8WYA6 | Beta-catenin-like protein 1 OS=Homo sapiens GN=CTNNBL1 PE=1 SV=1 - [CTBL1_HUMAN] | 3 | 3 | 2 | 563 | 65.1 | 5.05 |
| Q9HCD5 | Nuclear receptor coactivator 5 OS=Homo sapiens GN=NCOA5 PE=1 SV=2 - [NCOA5_HUMAN] | 3 | 2 | 1 | 579 | 65.5 | 9.60 |
| Q7Z4V5 | Hepatoma-derived growth factor-related protein 2 OS=Homo sapiens GN=HDGFRP2 PE=1 SV=1 - [HDGR2_HUMAN] | 3 | 2 | 4 | 671 | 74.3 | 7.49 |
| Q9H9T3 | Elongator complex protein 3 OS=Homo sapiens GN=ELP3 PE=1 SV=2 - [ELP3_HUMAN] | 3 | 2 | 3 | 547 | 62.2 | 8.88 |
| Q96A33 | Coiled-coil domain-containing protein 47 OS=Homo sapiens GN=CCDC47 PE=1 SV=1 - [CCD47_HUMAN] | 3 | 2 | 2 | 483 | 55.8 | 4.87 |
| O60568 | Procollagen-lysine.2-oxoglutarate 5-dioxygenase 3 OS=Homo sapiens GN=PLOD3 PE=1 SV=1 - [PLOD3_HUMAN] | 3 | 3 | 3 | 738 | 84.7 | 6.05 |
| P61201 | COP9 signalosome complex subunit 2 OS=Homo sapiens GN=COPS2 PE=1 SV=1 - [CSN2_HUMAN] | 3 | 2 | 2 | 443 | 51.6 | 5.53 |
| O14975 | Very long-chain acyl-CoA synthetase OS=Homo sapiens GN=SLC27A2 PE=1 SV=2 - [S27A2_HUMAN] | 3 | 3 | 5 | 620 | 70.3 | 8.51 |
| Q9UBD5 | Origin recognition complex subunit 3 OS=Homo sapiens GN=ORC3L PE=1 SV=1 - [ORC3_HUMAN] | 3 | 3 | 2 | 711 | 82.2 | 7.61 |
| Q9UKM7 | Endoplasmic reticulum mannosyl-oligosaccharide 1.2-alpha-mannosidase OS=Homo sapiens GN=MAN1B1 PE=1 SV=2 - [MA1B1_HUMAN] | 3 | 3 | 3 | 699 | 79.5 | 7.72 |
| P10155 | 60 kDa SS-A/Ro ribonucleoprotein OS=Homo sapiens GN=TROVE2 PE=1 SV=2 - [RO60_HUMAN] | 3 | 2 | 2 | 538 | 60.6 | 8.03 |
| Q9H074 | Polyadenylate-binding protein-interacting protein 1 OS=Homo sapiens GN=PAIP1 PE=1 SV=1 - [PAIP1_HUMAN] | 3 | 3 | 1 | 479 | 53.5 | 4.81 |
| Q9NQW7 | Xaa-Pro aminopeptidase 1 OS=Homo sapiens GN=XPNPEP1 PE=1 SV=3 - [XPP1_HUMAN] | 3 | 3 | 2 | 623 | 69.9 | 5.67 |
| Q9UNF0 | Protein kinase C and casein kinase substrate in neurons protein 2 OS=Homo sapiens GN=PACSIN2 PE=1 SV=2 - [PACN2_HUMAN] | 3 | 2 | 2 | 486 | 55.7 | 5.20 |
| P09960 | Leukotriene A-4 hydrolase OS=Homo sapiens GN=LTA4H PE=1 SV=2 - [LKHA4_HUMAN] | 3 | 1 | 3 | 611 | 69.2 | 6.18 |
| Q9UI26 | Importin-11 OS=Homo sapiens GN=IPO11 PE=1 SV=1 - [IPO11_HUMAN] | 3 | 4 | 2 | 975 | 112.5 | 5.25 |
| Q68E01 | Integrator complex subunit 3 OS=Homo sapiens GN=INTS3 PE=1 SV=1 - [INT3_HUMAN] | 3 | 1 | 3 | 1043 | 118.0 | 5.80 |
| Q15637 | Splicing factor 1 OS=Homo sapiens GN=SF1 PE=1 SV=4 - [SF01_HUMAN] | 3 | 2 | 3 | 639 | 68.3 | 8.98 |
| Q14139 | Ubiquitin conjugation factor E4 A OS=Homo sapiens GN=UBE4A PE=1 SV=2 - [UBE4A_HUMAN] | 3 | 3 | 4 | 1066 | 122.5 | 5.24 |
| O43929 | Origin recognition complex subunit 4 OS=Homo sapiens GN=ORC4L PE=1 SV=2 - [ORC4_HUMAN] | 3 | 3 | 2 | 436 | 50.3 | 8.00 |
| Q0VDF9 | Heat shock 70 kDa protein 14 OS=Homo sapiens GN=HSPA14 PE=1 SV=1 - [HSP7E_HUMAN] | 3 | 1 | 3 | 509 | 54.8 | 5.59 |
| P28370 | Probable global transcription activator SNF2L1 OS=Homo sapiens GN=SMARCA1 PE=1 SV=2 - [SMCA1_HUMAN] | 3 | 3 | 4 | 1054 | 122.5 | 8.09 |
| Q7L8L6 | FAST kinase domain-containing protein 5. mitochondrial OS=Homo sapiens GN=FASTKD5 PE=1 SV=1 - [FAKD5_HUMAN] | 3 | 3 | 2 | 764 | 86.5 | 8.13 |
| Q9Y450 | HBS1-like protein OS=Homo sapiens GN=HBS1L PE=1 SV=1 - [HBS1L_HUMAN] | 3 | 3 | 2 | 684 | 75.4 | 6.61 |
| P20073 | Annexin A7 OS=Homo sapiens GN=ANXA7 PE=1 SV=3 - [ANXA7_HUMAN] | 3 |  | 3 | 488 | 52.7 | 5.68 |
| P52735 | Guanine nucleotide exchange factor VAV2 OS=Homo sapiens GN=VAV2 PE=1 SV=2 - [VAV2_HUMAN] | 3 | 4 | 4 | 878 | 101.2 | 7.08 |
| Q02318 | Sterol 26-hydroxylase. mitochondrial OS=Homo sapiens GN=CYP27A1 PE=1 SV=1 - [CP27A_HUMAN] | 3 | 2 | 2 | 531 | 60.2 | 8.90 |
| Q9NSK0 | Kinesin light chain 4 OS=Homo sapiens GN=KLC4 PE=1 SV=3 - [KLC4_HUMAN] | 3 | 3 | 2 | 619 | 68.6 | 6.18 |
| P52888 | Thimet oligopeptidase OS=Homo sapiens GN=THOP1 PE=1 SV=2 - [THOP1_HUMAN] | 3 | 3 | 2 | 689 | 78.8 | 6.05 |
| O75534 | Cold shock domain-containing protein E1 OS=Homo sapiens GN=CSDE1 PE=1 SV=2 - [CSDE1_HUMAN] | 3 | 2 | 2 | 798 | 88.8 | 6.25 |
| O43490 | Prominin-1 OS=Homo sapiens GN=PROM1 PE=1 SV=1 - [PROM1_HUMAN] | 3 | 3 | 3 | 865 | 97.1 | 7.27 |
| Q13617 | Cullin-2 OS=Homo sapiens GN=CUL2 PE=1 SV=2 - [CUL2_HUMAN] | 3 | 1 | 2 | 745 | 86.9 | 6.92 |
| Q9Y2A7 | Nck-associated protein 1 OS=Homo sapiens GN=NCKAP1 PE=1 SV=1 - [NCKP1_HUMAN] | 3 | 1 | 3 | 1128 | 128.7 | 6.62 |
| Q99442 | Translocation protein SEC62 OS=Homo sapiens GN=SEC62 PE=1 SV=1 - [SEC62_HUMAN] | 3 |  | 3 | 399 | 45.8 | 7.12 |
| Q9GZR7 | ATP-dependent RNA helicase DDX24 OS=Homo sapiens GN=DDX24 PE=1 SV=1 - [DDX24_HUMAN] | 3 | 2 | 2 | 859 | 96.3 | 9.06 |
| Q6ZT62 | Bargin OS=Homo sapiens OX=9606 GN=BARGIN PE=1 SV=2 - [BGIN_HUMAN] | 3 | 2 | 2 | 677 | 73.6 | 5.24 |
| P02765 | Alpha-2-HS-glycoprotein OS=Homo sapiens OX=9606 GN=AHSG PE=1 SV=2 - [FETUA_HUMAN] | 3 | 5 | 5 | 367 | 39.3 | 5.72 |
| Q02809 | Procollagen-lysine.2-oxoglutarate 5-dioxygenase 1 OS=Homo sapiens GN=PLOD1 PE=1 SV=2 - [PLOD1_HUMAN] | 3 | 3 | 3 | 727 | 83.5 | 6.95 |
| Q6P1J9 | Parafibromin OS=Homo sapiens GN=CDC73 PE=1 SV=1 - [CDC73_HUMAN] | 3 | 2 | 3 | 531 | 60.5 | 9.61 |
| Q86TG7 | Retrotransposon-derived protein PEG10 OS=Homo sapiens GN=PEG10 PE=1 SV=2 - [PEG10_HUMAN] | 3 | 3 | 2 | 708 | 80.1 | 6.39 |
| Q9Y5A7 | NEDD8 ultimate buster 1 OS=Homo sapiens GN=NUB1 PE=1 SV=2 - [NUB1_HUMAN] | 3 |  | 3 | 615 | 70.5 | 5.96 |
| Q9NRZ9 | Lymphoid-specific helicase OS=Homo sapiens GN=HELLS PE=1 SV=1 - [HELLS_HUMAN] | 3 | 2 | 2 | 838 | 97.0 | 7.93 |
| Q02218 | 2-oxoglutarate dehydrogenase. mitochondrial OS=Homo sapiens GN=OGDH PE=1 SV=3 - [ODO1_HUMAN] | 3 | 3 | 3 | 1023 | 115.9 | 6.86 |
| O60264 | SWI/SNF-related matrix-associated actin-dependent regulator of chromatin subfamily A member 5 OS=Homo sapiens GN=SMARCA5 PE=1 SV=1 - [SMCA5_HUMAN] | 3 | 4 | 2 | 1052 | 121.8 | 8.09 |
| Q04726 | Transducin-like enhancer protein 3 OS=Homo sapiens GN=TLE3 PE=1 SV=2 - [TLE3_HUMAN] | 3 | 2 | 3 | 772 | 83.4 | 7.20 |
| Q14527 | Helicase-like transcription factor OS=Homo sapiens GN=HLTF PE=1 SV=2 - [HLTF_HUMAN] | 3 | 3 | 2 | 1009 | 113.9 | 8.60 |
| O00461 | Golgi integral membrane protein 4 OS=Homo sapiens GN=GOLIM4 PE=1 SV=1 - [GOLI4_HUMAN] | 3 | 2 | 3 | 696 | 81.8 | 4.77 |
| P29144 | Tripeptidyl-peptidase 2 OS=Homo sapiens GN=TPP2 PE=1 SV=4 - [TPP2_HUMAN] | 3 | 1 | 2 | 1249 | 138.3 | 6.32 |
| P55011 | Solute carrier family 12 member 2 OS=Homo sapiens GN=SLC12A2 PE=1 SV=1 - [S12A2_HUMAN] | 3 | 2 | 1 | 1212 | 131.4 | 6.40 |
| Q86XP3 | ATP-dependent RNA helicase DDX42 OS=Homo sapiens GN=DDX42 PE=1 SV=1 - [DDX42_HUMAN] | 3 | 3 | 1 | 938 | 102.9 | 7.02 |
| Q9BZH6 | WD repeat-containing protein 11 OS=Homo sapiens GN=WDR11 PE=1 SV=1 - [WDR11_HUMAN] | 3 | 3 | 3 | 1224 | 136.6 | 6.92 |
| P46087 | Putative ribosomal RNA methyltransferase NOP2 OS=Homo sapiens GN=NOP2 PE=1 SV=2 - [NOP2_HUMAN] | 3 | 2 | 3 | 812 | 89.2 | 9.23 |
| P02671 | Fibrinogen alpha chain OS=Homo sapiens GN=FGA PE=1 SV=2 - [FIBA_HUMAN] | 3 | 1 | 3 | 866 | 94.9 | 6.01 |
| Q32P28 | Prolyl 3-hydroxylase 1 OS=Homo sapiens GN=LEPRE1 PE=1 SV=2 - [P3H1_HUMAN] | 3 | 3 | 3 | 736 | 83.3 | 5.14 |
| Q9NYF8 | Bcl-2-associated transcription factor 1 OS=Homo sapiens GN=BCLAF1 PE=1 SV=2 - [BCLF1_HUMAN] | 3 | 3 | 3 | 920 | 106.1 | 9.98 |
| Q8N3U4 | Cohesin subunit SA-2 OS=Homo sapiens GN=STAG2 PE=1 SV=3 - [STAG2_HUMAN] | 3 | 2 | 3 | 1231 | 141.2 | 5.43 |
| O00139 | Kinesin-like protein KIF2A OS=Homo sapiens GN=KIF2A PE=1 SV=3 - [KIF2A_HUMAN] | 3 | 2 | 2 | 706 | 79.9 | 6.68 |
| O00159 | Unconventional myosin-Ic OS=Homo sapiens GN=MYO1C PE=1 SV=4 - [MYO1C_HUMAN] | 3 | 2 | 4 | 1063 | 121.6 | 9.41 |
| Q12797 | Aspartyl/asparaginyl beta-hydroxylase OS=Homo sapiens GN=ASPH PE=1 SV=3 - [ASPH_HUMAN] | 3 | 2 | 2 | 758 | 85.8 | 5.01 |
| O95163 | Elongator complex protein 1 OS=Homo sapiens GN=IKBKAP PE=1 SV=3 - [ELP1_HUMAN] | 3 | 2 | 3 | 1332 | 150.2 | 5.94 |
| Q9Y2W1 | Thyroid hormone receptor-associated protein 3 OS=Homo sapiens GN=THRAP3 PE=1 SV=2 - [TR150_HUMAN] | 3 | 3 | 2 | 955 | 108.6 | 10.15 |
| Q13435 | Splicing factor 3B subunit 2 OS=Homo sapiens GN=SF3B2 PE=1 SV=2 - [SF3B2_HUMAN] | 3 | 3 | 2 | 895 | 100.2 | 5.67 |
| Q7Z478 | ATP-dependent RNA helicase DHX29 OS=Homo sapiens GN=DHX29 PE=1 SV=2 - [DHX29_HUMAN] | 3 | 3 | 3 | 1369 | 155.1 | 8.09 |
| Q7Z460 | CLIP-associating protein 1 OS=Homo sapiens GN=CLASP1 PE=1 SV=1 - [CLAP1_HUMAN] | 3 | 3 | 5 | 1538 | 169.3 | 9.03 |
| Q01970 | 1-phosphatidylinositol-4.5-bisphosphate phosphodiesterase beta-3 OS=Homo sapiens GN=PLCB3 PE=1 SV=2 - [PLCB3_HUMAN] | 3 | 4 | 2 | 1234 | 138.7 | 5.90 |
| P25098 | Beta-adrenergic receptor kinase 1 OS=Homo sapiens GN=ADRBK1 PE=1 SV=2 - [ARBK1_HUMAN] | 3 | 1 | 3 | 689 | 79.5 | 7.28 |
| Q9BZJ0 | Crooked neck-like protein 1 OS=Homo sapiens GN=CRNKL1 PE=1 SV=4 - [CRNL1_HUMAN] | 3 | 3 | 3 | 848 | 100.4 | 8.00 |
| Q8NBJ5 | Procollagen galactosyltransferase 1 OS=Homo sapiens GN=GLT25D1 PE=1 SV=1 - [GT251_HUMAN] | 3 | 1 | 3 | 622 | 71.6 | 7.31 |
| O94832 | Myosin-Id OS=Homo sapiens GN=MYO1D PE=1 SV=2 - [MYO1D_HUMAN] | 3 | 3 | 3 | 1006 | 116.1 | 9.39 |
| Q9H583 | HEAT repeat-containing protein 1 OS=Homo sapiens GN=HEATR1 PE=1 SV=3 - [HEAT1_HUMAN] | 3 | 2 | 2 | 2144 | 242.2 | 6.54 |
| Q16706 | Alpha-mannosidase 2 OS=Homo sapiens GN=MAN2A1 PE=1 SV=2 - [MA2A1_HUMAN] | 3 | 2 | 1 | 1144 | 131.1 | 7.58 |
| O94906 | Pre-mRNA-processing factor 6 OS=Homo sapiens GN=PRPF6 PE=1 SV=1 - [PRP6_HUMAN] | 3 | 2 | 2 | 941 | 106.9 | 8.25 |
| P35251 | Replication factor C subunit 1 OS=Homo sapiens GN=RFC1 PE=1 SV=4 - [RFC1_HUMAN] | 3 | 2 | 3 | 1148 | 128.2 | 9.36 |
| Q6PKG0 | La-related protein 1 OS=Homo sapiens GN=LARP1 PE=1 SV=2 - [LARP1_HUMAN] | 3 | 3 | 4 | 1096 | 123.4 | 8.82 |
| Q86TI2 | Dipeptidyl peptidase 9 OS=Homo sapiens GN=DPP9 PE=1 SV=3 - [DPP9_HUMAN] | 3 | 2 | 2 | 863 | 98.2 | 6.46 |
| Q96QU8 | Exportin-6 OS=Homo sapiens GN=XPO6 PE=1 SV=1 - [XPO6_HUMAN] | 3 | 4 |  | 1125 | 128.8 | 6.35 |
| Q96ST3 | Paired amphipathic helix protein Sin3a OS=Homo sapiens GN=SIN3A PE=1 SV=2 - [SIN3A_HUMAN] | 3 | 1 | 4 | 1273 | 145.1 | 7.25 |
| P42695 | Condensin-2 complex subunit D3 OS=Homo sapiens GN=NCAPD3 PE=1 SV=2 - [CNDD3_HUMAN] | 3 | 2 | 3 | 1498 | 168.8 | 7.50 |
| O95487 | Protein transport protein Sec24B OS=Homo sapiens GN=SEC24B PE=1 SV=2 - [SC24B_HUMAN] | 3 | 2 | 3 | 1268 | 137.3 | 6.67 |
| Q6WKZ4 | Rab11 family-interacting protein 1 OS=Homo sapiens GN=RAB11FIP1 PE=1 SV=3 - [RFIP1_HUMAN] | 3 | 1 | 3 | 1283 | 137.1 | 5.43 |
| Q14683 | Structural maintenance of chromosomes protein 1A OS=Homo sapiens GN=SMC1A PE=1 SV=2 - [SMC1A_HUMAN] | 3 | 2 | 4 | 1233 | 143.1 | 7.64 |
| P51610 | Host cell factor 1 OS=Homo sapiens GN=HCFC1 PE=1 SV=2 - [HCFC1_HUMAN] | 3 | 2 | 3 | 2035 | 208.6 | 7.46 |
| Q9H3S7 | Tyrosine-protein phosphatase non-receptor type 23 OS=Homo sapiens GN=PTPN23 PE=1 SV=1 - [PTN23_HUMAN] | 3 | 1 | 2 | 1636 | 178.9 | 6.92 |
| Q10570 | Cleavage and polyadenylation specificity factor subunit 1 OS=Homo sapiens GN=CPSF1 PE=1 SV=2 - [CPSF1_HUMAN] | 3 | 1 | 2 | 1443 | 160.8 | 6.40 |
| O94822 | E3 ubiquitin-protein ligase listerin OS=Homo sapiens GN=LTN1 PE=1 SV=6 - [LTN1_HUMAN] | 3 | 2 | 3 | 1766 | 200.4 | 6.25 |
| Q14008 | Cytoskeleton-associated protein 5 OS=Homo sapiens GN=CKAP5 PE=1 SV=3 - [CKAP5_HUMAN] | 3 | 2 | 3 | 2032 | 225.4 | 7.80 |
| Q9UM54 | Myosin-VI OS=Homo sapiens GN=MYO6 PE=1 SV=4 - [MYO6_HUMAN] | 3 | 2 | 1 | 1294 | 149.6 | 8.53 |
| A5YKK6 | CCR4-NOT transcription complex subunit 1 OS=Homo sapiens GN=CNOT1 PE=1 SV=2 - [CNOT1_HUMAN] | 3 | 2 | 3 | 2376 | 266.8 | 7.11 |
| Q9BTC0 | Death-inducer obliterator 1 OS=Homo sapiens GN=DIDO1 PE=1 SV=5 - [DIDO1_HUMAN] | 3 | 2 | 2 | 2240 | 243.7 | 7.88 |
| Q09666 | Neuroblast differentiation-associated protein AHNAK OS=Homo sapiens GN=AHNAK PE=1 SV=2 - [AHNK_HUMAN] | 3 |  | 3 | 5890 | 628.7 | 6.15 |
| P61224 | Ras-related protein Rap-1b OS=Homo sapiens GN=RAP1B PE=1 SV=1 - [RAP1B_HUMAN] | 2 | 8 | 13 | 184 | 20.8 | 5.78 |
| P12236 | ADP/ATP translocase 3 OS=Homo sapiens GN=SLC25A6 PE=1 SV=4 - [ADT3_HUMAN] | 2 | 13 | 12 | 298 | 32.8 | 9.74 |
| Q9Y241 | HIG1 domain family member 1A OS=Homo sapiens GN=HIGD1A PE=1 SV=1 - [HIG1A_HUMAN] | 2 | 4 | 6 | 93 | 10.1 | 9.79 |
| P61586 | Transforming protein RhoA OS=Homo sapiens GN=RHOA PE=1 SV=1 - [RHOA_HUMAN] | 2 | 4 | 7 | 193 | 21.8 | 6.10 |
| P61970 | Nuclear transport factor 2 OS=Homo sapiens GN=NUTF2 PE=1 SV=1 - [NTF2_HUMAN] | 2 | 1 | 2 | 127 | 14.5 | 5.38 |
| Q9C005 | Protein dpy-30 homolog OS=Homo sapiens GN=DPY30 PE=1 SV=1 - [DPY30_HUMAN] | 2 | 3 |  | 99 | 11.2 | 4.88 |
| Q9Y281 | Cofilin-2 OS=Homo sapiens GN=CFL2 PE=1 SV=1 - [COF2_HUMAN] | 2 | 9 | 8 | 166 | 18.7 | 7.88 |
| P04908 | Histone H2A type 1-B/E OS=Homo sapiens GN=HIST1H2AB PE=1 SV=2 - [H2A1B_HUMAN] | 2 | 8 | 7 | 130 | 14.1 | 11.05 |
| Q96QV6 | Histone H2A type 1-A OS=Homo sapiens GN=HIST1H2AA PE=1 SV=3 - [H2A1A_HUMAN] | 2 | 6 | 6 | 131 | 14.2 | 10.86 |
| Q15843 | NEDD8 OS=Homo sapiens GN=NEDD8 PE=1 SV=1 - [NEDD8_HUMAN] | 2 | 5 | 4 | 81 | 9.1 | 8.43 |
| P68032 | Actin. alpha cardiac muscle 1 OS=Homo sapiens GN=ACTC1 PE=1 SV=1 - [ACTC_HUMAN] | 2 | 31 | 40 | 377 | 42.0 | 5.39 |
| P62841 | 40S ribosomal protein S15 OS=Homo sapiens GN=RPS15 PE=1 SV=2 - [RS15_HUMAN] | 2 | 2 | 3 | 145 | 17.0 | 10.39 |
| P04080 | Cystatin-B OS=Homo sapiens GN=CSTB PE=1 SV=2 - [CYTB_HUMAN] | 2 | 2 | 1 | 98 | 11.1 | 7.56 |
| P58546 | Myotrophin OS=Homo sapiens GN=MTPN PE=1 SV=2 - [MTPN_HUMAN] | 2 | 2 | 3 | 118 | 12.9 | 5.52 |
| Q8N2U0 | Transmembrane protein 256 OS=Homo sapiens GN=TMEM256 PE=3 SV=1 - [TM256_HUMAN] | 2 | 2 | 2 | 113 | 11.7 | 8.94 |
| P68036 | Ubiquitin-conjugating enzyme E2 L3 OS=Homo sapiens GN=UBE2L3 PE=1 SV=1 - [UB2L3_HUMAN] | 2 | 3 | 4 | 154 | 17.9 | 8.51 |
| Q15836 | Vesicle-associated membrane protein 3 OS=Homo sapiens GN=VAMP3 PE=1 SV=3 - [VAMP3_HUMAN] | 2 | 1 | 1 | 100 | 11.3 | 8.79 |
| P61513 | 60S ribosomal protein L37a OS=Homo sapiens GN=RPL37A PE=1 SV=2 - [RL37A_HUMAN] | 2 | 1 | 2 | 92 | 10.3 | 10.43 |
| Q8N668 | COMM domain-containing protein 1 OS=Homo sapiens GN=COMMD1 PE=1 SV=1 - [COMD1_HUMAN] | 2 | 3 | 1 | 190 | 21.2 | 6.20 |
| Q9UDX5 | Mitochondrial 18 kDa protein OS=Homo sapiens GN=MTP18 PE=1 SV=1 - [MTP18_HUMAN] | 2 | 1 | 2 | 166 | 18.0 | 9.31 |
| P56134 | ATP synthase subunit f. mitochondrial OS=Homo sapiens GN=ATP5J2 PE=1 SV=3 - [ATPK_HUMAN] | 2 | 1 | 3 | 94 | 10.9 | 9.67 |
| Q92930 | Ras-related protein Rab-8B OS=Homo sapiens GN=RAB8B PE=1 SV=2 - [RAB8B_HUMAN] | 2 | 9 | 8 | 207 | 23.6 | 9.07 |
| P06899 | Histone H2B type 1-J OS=Homo sapiens GN=HIST1H2BJ PE=1 SV=3 - [H2B1J_HUMAN] | 2 | 7 | 6 | 126 | 13.9 | 10.32 |
| O60814 | Histone H2B type 1-K OS=Homo sapiens GN=HIST1H2BK PE=1 SV=3 - [H2B1K_HUMAN] | 2 | 7 | 8 | 126 | 13.9 | 10.32 |
| P36873 | Serine/threonine-protein phosphatase PP1-gamma catalytic subunit OS=Homo sapiens GN=PPP1CC PE=1 SV=1 - [PP1G_HUMAN] | 2 | 10 | 10 | 323 | 37.0 | 6.54 |
| O14933 | Ubiquitin/ISG15-conjugating enzyme E2 L6 OS=Homo sapiens GN=UBE2L6 PE=1 SV=4 - [UB2L6_HUMAN] | 2 | 2 | 3 | 153 | 17.8 | 7.88 |
| P20674 | Cytochrome c oxidase subunit 5A. mitochondrial OS=Homo sapiens GN=COX5A PE=1 SV=2 - [COX5A_HUMAN] | 2 | 1 | 1 | 150 | 16.8 | 6.79 |
| P49458 | Signal recognition particle 9 kDa protein OS=Homo sapiens GN=SRP9 PE=1 SV=2 - [SRP09_HUMAN] | 2 | 2 | 2 | 86 | 10.1 | 7.97 |
| Q92688 | Acidic leucine-rich nuclear phosphoprotein 32 family member B OS=Homo sapiens GN=ANP32B PE=1 SV=1 - [AN32B_HUMAN] | 2 | 5 | 5 | 251 | 28.8 | 4.06 |
| P62136 | Serine/threonine-protein phosphatase PP1-alpha catalytic subunit OS=Homo sapiens GN=PPP1CA PE=1 SV=1 - [PP1A_HUMAN] | 2 | 10 | 8 | 330 | 37.5 | 6.33 |
| P07951 | Tropomyosin beta chain OS=Homo sapiens GN=TPM2 PE=1 SV=1 - [TPM2_HUMAN] | 2 | 8 | 6 | 284 | 32.8 | 4.70 |
| P09488 | Glutathione S-transferase Mu 1 OS=Homo sapiens GN=GSTM1 PE=1 SV=3 - [GSTM1_HUMAN] | 2 | 3 | 3 | 218 | 25.7 | 6.70 |
| P62306 | Small nuclear ribonucleoprotein F OS=Homo sapiens GN=SNRPF PE=1 SV=1 - [RUXF_HUMAN] | 2 | 2 | 1 | 86 | 9.7 | 4.67 |
| P61077 | Ubiquitin-conjugating enzyme E2 D3 OS=Homo sapiens GN=UBE2D3 PE=1 SV=1 - [UB2D3_HUMAN] | 2 | 3 | 3 | 147 | 16.7 | 7.80 |
| Q9NS69 | Mitochondrial import receptor subunit TOM22 homolog OS=Homo sapiens GN=TOMM22 PE=1 SV=3 - [TOM22_HUMAN] | 2 | 1 | 4 | 142 | 15.5 | 4.34 |
| P51668 | Ubiquitin-conjugating enzyme E2 D1 OS=Homo sapiens GN=UBE2D1 PE=1 SV=1 - [UB2D1_HUMAN] | 2 | 1 | 2 | 147 | 16.6 | 7.42 |
| O60888 | Protein CutA OS=Homo sapiens GN=CUTA PE=1 SV=2 - [CUTA_HUMAN] | 2 | 2 | 1 | 179 | 19.1 | 5.50 |
| Q9UI30 | tRNA methyltransferase 112 homolog OS=Homo sapiens GN=TRMT112 PE=1 SV=1 - [TR112_HUMAN] | 2 | 2 | 2 | 125 | 14.2 | 5.26 |
| Q99471 | Prefoldin subunit 5 OS=Homo sapiens GN=PFDN5 PE=1 SV=2 - [PFD5_HUMAN] | 2 | 2 | 2 | 154 | 17.3 | 6.33 |
| P52434 | DNA-directed RNA polymerases I. II. and III subunit RPABC3 OS=Homo sapiens GN=POLR2H PE=1 SV=4 - [RPAB3_HUMAN] | 2 | 3 | 2 | 150 | 17.1 | 4.68 |
| O75348 | V-type proton ATPase subunit G 1 OS=Homo sapiens GN=ATP6V1G1 PE=1 SV=3 - [VATG1_HUMAN] | 2 | 2 | 1 | 118 | 13.7 | 8.79 |
| P30046 | D-dopachrome decarboxylase OS=Homo sapiens GN=DDT PE=1 SV=3 - [DOPD_HUMAN] | 2 | 1 | 2 | 118 | 12.7 | 7.30 |
| P84090 | Enhancer of rudimentary homolog OS=Homo sapiens GN=ERH PE=1 SV=1 - [ERH_HUMAN] | 2 | 2 | 1 | 104 | 12.3 | 5.92 |
| Q9P000 | COMM domain-containing protein 9 OS=Homo sapiens GN=COMMD9 PE=1 SV=2 - [COMD9_HUMAN] | 2 | 2 | 2 | 198 | 21.8 | 5.88 |
| P62847 | 40S ribosomal protein S24 OS=Homo sapiens GN=RPS24 PE=1 SV=1 - [RS24_HUMAN] | 2 | 1 | 3 | 133 | 15.4 | 10.78 |
| P26885 | Peptidyl-prolyl cis-trans isomerase FKBP2 OS=Homo sapiens GN=FKBP2 PE=1 SV=2 - [FKBP2_HUMAN] | 2 | 2 | 1 | 142 | 15.6 | 9.13 |
| P62993 | Growth factor receptor-bound protein 2 OS=Homo sapiens GN=GRB2 PE=1 SV=1 - [GRB2_HUMAN] | 2 | 2 | 2 | 217 | 25.2 | 6.32 |
| P24390 | ER lumen protein-retaining receptor 1 OS=Homo sapiens GN=KDELR1 PE=1 SV=1 - [ERD21_HUMAN] | 2 | 3 | 4 | 212 | 24.5 | 8.62 |
| Q9P0J0 | NADH dehydrogenase [ubiquinone] 1 alpha subcomplex subunit 13 OS=Homo sapiens GN=NDUFA13 PE=1 SV=3 - [NDUAD_HUMAN] | 2 | 3 | 3 | 144 | 16.7 | 8.43 |
| P55769 | NHP2-like protein 1 OS=Homo sapiens GN=NHP2L1 PE=1 SV=3 - [NH2L1_HUMAN] | 2 | 2 | 2 | 128 | 14.2 | 8.46 |
| P56556 | NADH dehydrogenase [ubiquinone] 1 alpha subcomplex subunit 6 OS=Homo sapiens OX=9606 GN=NDUFA6 PE=1 SV=4 - [NDUA6_HUMAN] | 2 | 2 | 2 | 128 | 15.1 | 9.98 |
| P42766 | 60S ribosomal protein L35 OS=Homo sapiens GN=RPL35 PE=1 SV=2 - [RL35_HUMAN] | 2 | 2 | 2 | 123 | 14.5 | 11.05 |
| Q9H061 | Transmembrane protein 126A OS=Homo sapiens GN=TMEM126A PE=1 SV=1 - [T126A_HUMAN] | 2 | 2 |  | 195 | 21.5 | 9.26 |
| O75934 | Pre-mRNA-splicing factor SPF27 OS=Homo sapiens GN=BCAS2 PE=1 SV=1 - [SPF27_HUMAN] | 2 | 1 | 2 | 225 | 26.1 | 5.66 |
| Q9Y3U8 | 60S ribosomal protein L36 OS=Homo sapiens GN=RPL36 PE=1 SV=3 - [RL36_HUMAN] | 2 | 1 | 3 | 105 | 12.2 | 11.59 |
| Q15404 | Ras suppressor protein 1 OS=Homo sapiens GN=RSU1 PE=1 SV=3 - [RSU1_HUMAN] | 2 | 2 | 2 | 277 | 31.5 | 8.65 |
| P27361 | Mitogen-activated protein kinase 3 OS=Homo sapiens GN=MAPK3 PE=1 SV=4 - [MK03_HUMAN] | 2 | 5 | 3 | 379 | 43.1 | 6.74 |
| Q8N5M1 | ATP synthase mitochondrial F1 complex assembly factor 2 OS=Homo sapiens GN=ATPAF2 PE=1 SV=1 - [ATPF2_HUMAN] | 2 | 2 | 2 | 289 | 32.8 | 7.09 |
| Q08623 | Haloacid dehalogenase-like hydrolase domain-containing protein 1A OS=Homo sapiens GN=HDHD1A PE=1 SV=3 - [HDD1A_HUMAN] | 2 | 1 | 2 | 228 | 25.2 | 5.31 |
| P14174 | Macrophage migration inhibitory factor OS=Homo sapiens GN=MIF PE=1 SV=4 - [MIF_HUMAN] | 2 | 4 | 6 | 115 | 12.5 | 7.88 |
| P16455 | Methylated-DNA--protein-cysteine methyltransferase OS=Homo sapiens GN=MGMT PE=1 SV=1 - [MGMT_HUMAN] | 2 |  | 2 | 207 | 21.6 | 8.10 |
| O15116 | U6 snRNA-associated Sm-like protein LSm1 OS=Homo sapiens GN=LSM1 PE=1 SV=1 - [LSM1_HUMAN] | 2 | 2 | 1 | 133 | 15.2 | 5.22 |
| Q9Y5S9 | RNA-binding protein 8A OS=Homo sapiens GN=RBM8A PE=1 SV=1 - [RBM8A_HUMAN] | 2 | 3 | 3 | 174 | 19.9 | 5.72 |
| P20339 | Ras-related protein Rab-5A OS=Homo sapiens GN=RAB5A PE=1 SV=2 - [RAB5A_HUMAN] | 2 | 3 | 3 | 215 | 23.6 | 8.15 |
| P61020 | Ras-related protein Rab-5B OS=Homo sapiens GN=RAB5B PE=1 SV=1 - [RAB5B_HUMAN] | 2 | 2 | 3 | 215 | 23.7 | 8.13 |
| Q96DG6 | Carboxymethylenebutenolidase homolog OS=Homo sapiens GN=CMBL PE=1 SV=1 - [CMBL_HUMAN] | 2 |  | 2 | 245 | 28.0 | 7.18 |
| Q8N0U8 | Vitamin K epoxide reductase complex subunit 1-like protein 1 OS=Homo sapiens GN=VKORC1L1 PE=1 SV=2 - [VKORL_HUMAN] | 2 |  | 2 | 176 | 19.8 | 9.13 |
| P62910 | 60S ribosomal protein L32 OS=Homo sapiens GN=RPL32 PE=1 SV=2 - [RL32_HUMAN] | 2 | 2 | 4 | 135 | 15.8 | 11.33 |
| P32322 | Pyrroline-5-carboxylate reductase 1. mitochondrial OS=Homo sapiens GN=PYCR1 PE=1 SV=2 - [P5CR1_HUMAN] | 2 | 3 | 4 | 319 | 33.3 | 7.61 |
| O95292 | Vesicle-associated membrane protein-associated protein B/C OS=Homo sapiens GN=VAPB PE=1 SV=3 - [VAPB_HUMAN] | 2 | 1 | 4 | 243 | 27.2 | 7.30 |
| P51571 | Translocon-associated protein subunit delta OS=Homo sapiens GN=SSR4 PE=1 SV=1 - [SSRD_HUMAN] | 2 | 3 | 2 | 173 | 19.0 | 6.15 |
| Q8IUR0 | Trafficking protein particle complex subunit 5 OS=Homo sapiens GN=TRAPPC5 PE=1 SV=1 - [TPPC5_HUMAN] | 2 |  | 2 | 188 | 20.8 | 9.66 |
| Q9BPX5 | Actin-related protein 2/3 complex subunit 5-like protein OS=Homo sapiens GN=ARPC5L PE=1 SV=1 - [ARP5L_HUMAN] | 2 | 2 | 1 | 153 | 16.9 | 6.60 |
| P62714 | Serine/threonine-protein phosphatase 2A catalytic subunit beta isoform OS=Homo sapiens GN=PPP2CB PE=1 SV=1 - [PP2AB_HUMAN] | 2 | 2 | 3 | 309 | 35.6 | 5.43 |
| P28161 | Glutathione S-transferase Mu 2 OS=Homo sapiens GN=GSTM2 PE=1 SV=2 - [GSTM2_HUMAN] | 2 | 1 | 3 | 218 | 25.7 | 6.37 |
| P62633 | Cellular nucleic acid-binding protein OS=Homo sapiens GN=CNBP PE=1 SV=1 - [CNBP_HUMAN] | 2 | 1 | 3 | 177 | 19.4 | 7.71 |
| O95563 | Brain protein 44 OS=Homo sapiens GN=BRP44 PE=1 SV=1 - [BR44_HUMAN] | 2 |  | 2 | 127 | 14.3 | 10.43 |
| Q9UMY4 | Sorting nexin-12 OS=Homo sapiens GN=SNX12 PE=1 SV=3 - [SNX12_HUMAN] | 2 | 2 | 1 | 172 | 19.7 | 7.87 |
| Q04837 | Single-stranded DNA-binding protein. mitochondrial OS=Homo sapiens GN=SSBP1 PE=1 SV=1 - [SSBP_HUMAN] | 2 | 2 | 2 | 148 | 17.2 | 9.60 |
| O15258 | Protein RER1 OS=Homo sapiens GN=RER1 PE=1 SV=1 - [RER1_HUMAN] | 2 | 2 | 2 | 196 | 22.9 | 9.54 |
| P51452 | Dual specificity protein phosphatase 3 OS=Homo sapiens GN=DUSP3 PE=1 SV=1 - [DUS3_HUMAN] | 2 | 2 | 1 | 185 | 20.5 | 7.80 |
| Q86X83 | COMM domain-containing protein 2 OS=Homo sapiens GN=COMMD2 PE=1 SV=2 - [COMD2_HUMAN] | 2 | 1 | 2 | 199 | 22.7 | 6.73 |
| O75663 | TIP41-like protein OS=Homo sapiens GN=TIPRL PE=1 SV=2 - [TIPRL_HUMAN] | 2 | 1 | 1 | 272 | 31.4 | 5.91 |
| O15305 | Phosphomannomutase 2 OS=Homo sapiens GN=PMM2 PE=1 SV=1 - [PMM2_HUMAN] | 2 | 1 | 2 | 246 | 28.1 | 6.77 |
| Q9BRV3 | Sugar transporter SWEET1 OS=Homo sapiens OX=9606 GN=SLC50A1 PE=2 SV=1 - [SWET1_HUMAN] | 2 | 2 | 1 | 221 | 25.0 | 8.54 |
| O60493 | Sorting nexin-3 OS=Homo sapiens GN=SNX3 PE=1 SV=3 - [SNX3_HUMAN] | 2 | 3 | 1 | 162 | 18.8 | 8.66 |
| Q99436 | Proteasome subunit beta type-7 OS=Homo sapiens GN=PSMB7 PE=1 SV=1 - [PSB7_HUMAN] | 2 | 2 | 2 | 277 | 29.9 | 7.68 |
| O95372 | Acyl-protein thioesterase 2 OS=Homo sapiens GN=LYPLA2 PE=1 SV=1 - [LYPA2_HUMAN] | 2 | 3 | 2 | 231 | 24.7 | 7.23 |
| Q16629 | Splicing factor. arginine/serine-rich 7 OS=Homo sapiens GN=SFRS7 PE=1 SV=1 - [SFRS7_HUMAN] | 2 | 3 |  | 238 | 27.4 | 11.82 |
| P24666 | Low molecular weight phosphotyrosine protein phosphatase OS=Homo sapiens GN=ACP1 PE=1 SV=3 - [PPAC_HUMAN] | 2 | 1 | 1 | 158 | 18.0 | 6.74 |
| P49006 | MARCKS-related protein OS=Homo sapiens GN=MARCKSL1 PE=1 SV=2 - [MRP_HUMAN] | 2 | 2 | 1 | 195 | 19.5 | 4.67 |
| P55957 | BH3-interacting domain death agonist OS=Homo sapiens GN=BID PE=1 SV=1 - [BID_HUMAN] | 2 | 1 | 2 | 195 | 22.0 | 5.44 |
| Q9UKY7 | Protein CDV3 homolog OS=Homo sapiens GN=CDV3 PE=1 SV=1 - [CDV3_HUMAN] | 2 | 2 |  | 258 | 27.3 | 6.40 |
| Q15800 | Methylsterol monooxygenase 1 OS=Homo sapiens GN=MSMO1 PE=1 SV=1 - [MSMO1_HUMAN] | 2 | 1 | 2 | 293 | 35.2 | 7.23 |
| P99999 | Cytochrome c OS=Homo sapiens GN=CYCS PE=1 SV=2 - [CYC_HUMAN] | 2 | 2 | 2 | 105 | 11.7 | 9.57 |
| Q9Y3C4 | TP53RK-binding protein OS=Homo sapiens GN=TPRKB PE=1 SV=1 - [TPRKB_HUMAN] | 2 | 1 | 1 | 175 | 19.6 | 6.79 |
| P51572 | B-cell receptor-associated protein 31 OS=Homo sapiens GN=BCAP31 PE=1 SV=3 - [BAP31_HUMAN] | 2 | 1 | 1 | 246 | 28.0 | 8.44 |
| Q13232 | Nucleoside diphosphate kinase 3 OS=Homo sapiens GN=NME3 PE=1 SV=2 - [NDK3_HUMAN] | 2 | 1 | 2 | 169 | 19.0 | 7.84 |
| Q13126 | S-methyl-5'-thioadenosine phosphorylase OS=Homo sapiens GN=MTAP PE=1 SV=2 - [MTAP_HUMAN] | 2 |  | 2 | 283 | 31.2 | 7.18 |
| P11233 | Ras-related protein Ral-A OS=Homo sapiens GN=RALA PE=1 SV=1 - [RALA_HUMAN] | 2 | 1 | 2 | 206 | 23.6 | 7.11 |
| Q9H9Q2 | COP9 signalosome complex subunit 7b OS=Homo sapiens GN=COPS7B PE=1 SV=1 - [CSN7B_HUMAN] | 2 | 4 | 3 | 264 | 29.6 | 6.15 |
| O95881 | Thioredoxin domain-containing protein 12 OS=Homo sapiens GN=TXNDC12 PE=1 SV=1 - [TXD12_HUMAN] | 2 | 1 | 1 | 172 | 19.2 | 5.40 |
| Q9UBX3 | Mitochondrial dicarboxylate carrier OS=Homo sapiens GN=SLC25A10 PE=1 SV=2 - [DIC_HUMAN] | 2 | 3 | 2 | 287 | 31.3 | 9.54 |
| P30049 | ATP synthase subunit delta. mitochondrial OS=Homo sapiens GN=ATP5D PE=1 SV=2 - [ATPD_HUMAN] | 2 | 2 | 2 | 168 | 17.5 | 5.49 |
| Q13907 | Isopentenyl-diphosphate Delta-isomerase 1 OS=Homo sapiens GN=IDI1 PE=1 SV=2 - [IDI1_HUMAN] | 2 | 1 | 2 | 227 | 26.3 | 6.34 |
| P40261 | Nicotinamide N-methyltransferase OS=Homo sapiens GN=NNMT PE=1 SV=1 - [NNMT_HUMAN] | 2 | 1 | 2 | 264 | 29.6 | 5.74 |
| P54709 | Sodium/potassium-transporting ATPase subunit beta-3 OS=Homo sapiens GN=ATP1B3 PE=1 SV=1 - [AT1B3_HUMAN] | 2 | 1 | 2 | 279 | 31.5 | 8.35 |
| Q969G6 | Riboflavin kinase OS=Homo sapiens GN=RFK PE=1 SV=2 - [RIFK_HUMAN] | 2 | 4 | 3 | 155 | 17.6 | 8.13 |
| O15145 | Actin-related protein 2/3 complex subunit 3 OS=Homo sapiens GN=ARPC3 PE=1 SV=3 - [ARPC3_HUMAN] | 2 | 2 | 1 | 178 | 20.5 | 8.59 |
| Q96GA7 | Serine dehydratase-like OS=Homo sapiens GN=SDSL PE=1 SV=1 - [SDSL_HUMAN] | 2 | 1 | 2 | 329 | 34.7 | 6.89 |
| O00161 | Synaptosomal-associated protein 23 OS=Homo sapiens GN=SNAP23 PE=1 SV=1 - [SNP23_HUMAN] | 2 | 2 | 1 | 211 | 23.3 | 5.01 |
| P54725 | UV excision repair protein RAD23 homolog A OS=Homo sapiens GN=RAD23A PE=1 SV=1 - [RD23A_HUMAN] | 2 | 4 | 3 | 363 | 39.6 | 4.58 |
| P09012 | U1 small nuclear ribonucleoprotein A OS=Homo sapiens GN=SNRPA PE=1 SV=3 - [SNRPA_HUMAN] | 2 | 2 | 3 | 282 | 31.3 | 9.83 |
| Q8N5M9 | Protein jagunal homolog 1 OS=Homo sapiens GN=JAGN1 PE=1 SV=1 - [JAGN1_HUMAN] | 2 |  | 2 | 183 | 21.1 | 9.73 |
| Q5BJD5 | Transmembrane protein 41B OS=Homo sapiens GN=TMEM41B PE=1 SV=1 - [TM41B_HUMAN] | 2 | 2 | 3 | 291 | 32.5 | 9.58 |
| Q15382 | GTP-binding protein Rheb OS=Homo sapiens GN=RHEB PE=1 SV=1 - [RHEB_HUMAN] | 2 | 1 | 2 | 184 | 20.5 | 5.92 |
| P21291 | Cysteine and glycine-rich protein 1 OS=Homo sapiens GN=CSRP1 PE=1 SV=3 - [CSRP1_HUMAN] | 2 | 2 | 1 | 193 | 20.6 | 8.57 |
| Q16527 | Cysteine and glycine-rich protein 2 OS=Homo sapiens GN=CSRP2 PE=1 SV=3 - [CSRP2_HUMAN] | 2 | 1 | 2 | 193 | 20.9 | 8.62 |
| P00568 | Adenylate kinase isoenzyme 1 OS=Homo sapiens GN=AK1 PE=1 SV=3 - [KAD1_HUMAN] | 2 | 2 | 3 | 194 | 21.6 | 8.63 |
| Q9BRP8 | Partner of Y14 and mago OS=Homo sapiens GN=PYM1 PE=1 SV=1 - [PYM1_HUMAN] | 2 | 2 | 2 | 204 | 22.6 | 9.45 |
| Q16836 | Hydroxyacyl-coenzyme A dehydrogenase. mitochondrial OS=Homo sapiens GN=HADH PE=1 SV=3 - [HCDH_HUMAN] | 2 | 2 | 3 | 314 | 34.3 | 8.85 |
| P52294 | Importin subunit alpha-5 OS=Homo sapiens GN=KPNA1 PE=1 SV=3 - [IMA5_HUMAN] | 2 | 5 | 2 | 538 | 60.2 | 5.01 |
| Q9NP72 | Ras-related protein Rab-18 OS=Homo sapiens GN=RAB18 PE=1 SV=1 - [RAB18_HUMAN] | 2 |  | 2 | 206 | 23.0 | 5.24 |
| P53365 | Arfaptin-2 OS=Homo sapiens GN=ARFIP2 PE=1 SV=1 - [ARFP2_HUMAN] | 2 | 1 | 2 | 341 | 37.8 | 6.04 |
| Q86YN1 | Dolichyldiphosphatase 1 OS=Homo sapiens GN=DOLPP1 PE=2 SV=1 - [DOPP1_HUMAN] | 2 | 3 | 1 | 238 | 27.0 | 9.44 |
| Q96DI7 | U5 small nuclear ribonucleoprotein 40 kDa protein OS=Homo sapiens GN=SNRNP40 PE=1 SV=1 - [SNR40_HUMAN] | 2 |  | 2 | 357 | 39.3 | 8.10 |
| Q07812 | Apoptosis regulator BAX OS=Homo sapiens GN=BAX PE=1 SV=1 - [BAX_HUMAN] | 2 | 1 | 1 | 192 | 21.2 | 5.22 |
| Q02750 | Dual specificity mitogen-activated protein kinase kinase 1 OS=Homo sapiens GN=MAP2K1 PE=1 SV=2 - [MP2K1_HUMAN] | 2 | 4 | 4 | 393 | 43.4 | 6.62 |
| Q01995 | Transgelin OS=Homo sapiens GN=TAGLN PE=1 SV=4 - [TAGL_HUMAN] | 2 | 2 |  | 201 | 22.6 | 8.84 |
| Q9UBQ5 | Eukaryotic translation initiation factor 3 subunit K OS=Homo sapiens GN=EIF3K PE=1 SV=1 - [EIF3K_HUMAN] | 2 | 3 | 3 | 218 | 25.0 | 4.93 |
| O75477 | Erlin-1 OS=Homo sapiens OX=9606 GN=ERLIN1 PE=1 SV=2 - [ERLN1_HUMAN] | 2 | 2 | 3 | 348 | 39.1 | 7.87 |
| Q92520 | Protein FAM3C OS=Homo sapiens GN=FAM3C PE=1 SV=1 - [FAM3C_HUMAN] | 2 |  | 2 | 227 | 24.7 | 8.29 |
| O75223 | Gamma-glutamylcyclotransferase OS=Homo sapiens GN=GGCT PE=1 SV=1 - [GGCT_HUMAN] | 2 |  | 2 | 188 | 21.0 | 5.14 |
| Q9NRX1 | RNA-binding protein PNO1 OS=Homo sapiens GN=PNO1 PE=1 SV=1 - [PNO1_HUMAN] | 2 | 2 | 1 | 252 | 27.9 | 9.73 |
| Q9UIJ7 | GTP:AMP phosphotransferase mitochondrial OS=Homo sapiens GN=AK3 PE=1 SV=4 - [KAD3_HUMAN] | 2 | 1 | 1 | 227 | 25.5 | 9.16 |
| Q04323 | UBX domain-containing protein 1 OS=Homo sapiens GN=UBXN1 PE=1 SV=2 - [UBXN1_HUMAN] | 2 | 1 | 1 | 297 | 33.3 | 5.25 |
| O14929 | Histone acetyltransferase type B catalytic subunit OS=Homo sapiens GN=HAT1 PE=1 SV=1 - [HAT1_HUMAN] | 2 | 1 | 2 | 419 | 49.5 | 5.69 |
| Q96A26 | Protein FAM162A OS=Homo sapiens GN=FAM162A PE=1 SV=2 - [F162A_HUMAN] | 2 | 2 | 2 | 154 | 17.3 | 9.77 |
| P61009 | Signal peptidase complex subunit 3 OS=Homo sapiens GN=SPCS3 PE=1 SV=1 - [SPCS3_HUMAN] | 2 | 1 | 2 | 180 | 20.3 | 8.62 |
| Q9NZJ9 | Diphosphoinositol polyphosphate phosphohydrolase 2 OS=Homo sapiens GN=NUDT4 PE=1 SV=2 - [NUDT4_HUMAN] | 2 |  | 2 | 180 | 20.3 | 6.35 |
| O14732 | Inositol monophosphatase 2 OS=Homo sapiens GN=IMPA2 PE=1 SV=1 - [IMPA2_HUMAN] | 2 | 2 | 2 | 288 | 31.3 | 6.61 |
| Q9BSE5 | Agmatinase. mitochondrial OS=Homo sapiens GN=AGMAT PE=1 SV=2 - [SPEB_HUMAN] | 2 | 2 | 1 | 352 | 37.6 | 7.59 |
| Q9NX58 | Cell growth-regulating nucleolar protein OS=Homo sapiens GN=LYAR PE=1 SV=2 - [LYAR_HUMAN] | 2 | 1 | 2 | 379 | 43.6 | 9.54 |
| P61964 | WD repeat-containing protein 5 OS=Homo sapiens GN=WDR5 PE=1 SV=1 - [WDR5_HUMAN] | 2 | 2 | 1 | 334 | 36.6 | 8.27 |
| Q13190 | Syntaxin-5 OS=Homo sapiens GN=STX5 PE=1 SV=2 - [STX5_HUMAN] | 2 | 2 |  | 355 | 39.6 | 9.16 |
| Q15126 | Phosphomevalonate kinase OS=Homo sapiens GN=PMVK PE=1 SV=3 - [PMVK_HUMAN] | 2 | 2 | 3 | 192 | 22.0 | 5.73 |
| Q9NPL8 | Complex I assembly factor TIMMDC1. mitochondrial OS=Homo sapiens GN=TIMMDC1 PE=1 SV=2 - [TIDC1_HUMAN] | 2 | 2 | 2 | 285 | 32.2 | 8.50 |
| O95758 | Regulator of differentiation 1 OS=Homo sapiens GN=ROD1 PE=1 SV=2 - [ROD1_HUMAN] | 2 | 5 | 4 | 552 | 59.7 | 9.04 |
| Q96K37 | Solute carrier family 35 member E1 OS=Homo sapiens GN=SLC35E1 PE=1 SV=2 - [S35E1_HUMAN] | 2 | 1 | 2 | 410 | 44.7 | 9.79 |
| Q99943 | 1-acyl-sn-glycerol-3-phosphate acyltransferase alpha OS=Homo sapiens GN=AGPAT1 PE=2 SV=2 - [PLCA_HUMAN] | 2 | 2 | 2 | 283 | 31.7 | 9.38 |
| P30837 | Aldehyde dehydrogenase X. mitochondrial OS=Homo sapiens GN=ALDH1B1 PE=1 SV=3 - [AL1B1_HUMAN] | 2 | 5 | 5 | 517 | 57.2 | 6.80 |
| Q8WVC6 | Dephospho-CoA kinase domain-containing protein OS=Homo sapiens GN=DCAKD PE=1 SV=1 - [DCAKD_HUMAN] | 2 |  | 2 | 231 | 26.5 | 9.58 |
| Q9BY43 | Charged multivesicular body protein 4a OS=Homo sapiens GN=CHMP4A PE=1 SV=3 - [CHM4A_HUMAN] | 2 | 1 | 1 | 222 | 25.1 | 4.70 |
| Q9NRN7 | L-aminoadipate-semialdehyde dehydrogenase-phosphopantetheinyl transferase OS=Homo sapiens GN=AASDHPPT PE=1 SV=2 - [ADPPT_HUMAN] | 2 | 3 | 3 | 309 | 35.8 | 6.80 |
| Q6PIU2 | Neutral cholesterol ester hydrolase 1 OS=Homo sapiens GN=NCEH1 PE=1 SV=3 - [NCEH1_HUMAN] | 2 | 2 | 3 | 408 | 45.8 | 7.23 |
| Q8NEJ9 | Neuroguidin OS=Homo sapiens GN=NGDN PE=1 SV=1 - [NGDN_HUMAN] | 2 | 2 | 2 | 315 | 35.9 | 9.57 |
| P27216 | Annexin A13 OS=Homo sapiens GN=ANXA13 PE=1 SV=3 - [ANX13_HUMAN] | 2 | 3 | 1 | 316 | 35.4 | 5.60 |
| Q96C86 | m7GpppX diphosphatase OS=Homo sapiens GN=DCPS PE=1 SV=2 - [DCPS_HUMAN] | 2 | 1 | 1 | 337 | 38.6 | 6.38 |
| P55058 | Phospholipid transfer protein OS=Homo sapiens GN=PLTP PE=1 SV=1 - [PLTP_HUMAN] | 2 |  | 2 | 493 | 54.7 | 7.01 |
| Q9NZL9 | Methionine adenosyltransferase 2 subunit beta OS=Homo sapiens GN=MAT2B PE=1 SV=1 - [MAT2B_HUMAN] | 2 | 2 | 2 | 334 | 37.5 | 7.36 |
| Q9Y3B3 | Transmembrane emp24 domain-containing protein 7 OS=Homo sapiens GN=TMED7 PE=1 SV=2 - [TMED7_HUMAN] | 2 | 2 | 2 | 224 | 25.2 | 6.89 |
| Q92504 | Zinc transporter SLC39A7 OS=Homo sapiens GN=SLC39A7 PE=1 SV=2 - [S39A7_HUMAN] | 2 | 3 | 2 | 469 | 50.1 | 6.87 |
| Q9UKZ1 | UPF0760 protein C2orf29 OS=Homo sapiens GN=C2orf29 PE=1 SV=1 - [CB029_HUMAN] | 2 | 2 |  | 510 | 55.2 | 6.40 |
| Q9P016 | Thymocyte nuclear protein 1 OS=Homo sapiens GN=THYN1 PE=1 SV=1 - [THYN1_HUMAN] | 2 | 2 | 3 | 225 | 25.7 | 9.25 |
| Q15645 | Thyroid receptor-interacting protein 13 OS=Homo sapiens GN=TRIP13 PE=1 SV=2 - [TRP13_HUMAN] | 2 | 2 | 1 | 432 | 48.5 | 6.09 |
| Q86W42 | THO complex subunit 6 homolog OS=Homo sapiens GN=THOC6 PE=1 SV=1 - [THOC6_HUMAN] | 2 | 2 | 1 | 341 | 37.5 | 7.43 |
| P49755 | Transmembrane emp24 domain-containing protein 10 OS=Homo sapiens GN=TMED10 PE=1 SV=2 - [TMEDA_HUMAN] | 2 | 1 | 2 | 219 | 25.0 | 7.44 |
| O95299 | NADH dehydrogenase [ubiquinone] 1 alpha subcomplex subunit 10. mitochondrial OS=Homo sapiens GN=NDUFA10 PE=1 SV=1 - [NDUAA_HUMAN] | 2 | 1 | 2 | 355 | 40.7 | 8.48 |
| P50213 | Isocitrate dehydrogenase [NAD] subunit alpha. mitochondrial OS=Homo sapiens GN=IDH3A PE=1 SV=1 - [IDH3A_HUMAN] | 2 | 1 | 2 | 366 | 39.6 | 6.92 |
| Q96C90 | Protein phosphatase 1 regulatory subunit 14B OS=Homo sapiens GN=PPP1R14B PE=1 SV=3 - [PP14B_HUMAN] | 2 | 2 |  | 147 | 15.9 | 4.86 |
| O00217 | NADH dehydrogenase [ubiquinone] iron-sulfur protein 8. mitochondrial OS=Homo sapiens GN=NDUFS8 PE=1 SV=1 - [NDUS8_HUMAN] | 2 | 1 | 2 | 210 | 23.7 | 6.34 |
| Q8NFH4 | Nucleoporin Nup37 OS=Homo sapiens GN=NUP37 PE=1 SV=1 - [NUP37_HUMAN] | 2 | 1 | 2 | 326 | 36.7 | 5.92 |
| Q5VT66 | Mitochondrial amidoxime-reducing component 1 OS=Homo sapiens GN=MARC1 PE=1 SV=1 - [MARC1_HUMAN] | 2 | 1 | 2 | 337 | 37.5 | 8.88 |
| O95816 | BAG family molecular chaperone regulator 2 OS=Homo sapiens GN=BAG2 PE=1 SV=1 - [BAG2_HUMAN] | 2 | 1 | 2 | 211 | 23.8 | 6.70 |
| Q9BT22 | Chitobiosyldiphosphodolichol beta-mannosyltransferase OS=Homo sapiens GN=ALG1 PE=1 SV=2 - [ALG1_HUMAN] | 2 | 1 | 2 | 464 | 52.5 | 7.23 |
| P28070 | Proteasome subunit beta type-4 OS=Homo sapiens GN=PSMB4 PE=1 SV=4 - [PSB4_HUMAN] | 2 | 2 | 3 | 264 | 29.2 | 5.97 |
| Q92604 | Acyl-CoA:lysophosphatidylglycerol acyltransferase 1 OS=Homo sapiens GN=LPGAT1 PE=1 SV=1 - [LGAT1_HUMAN] | 2 | 2 | 1 | 370 | 43.1 | 8.92 |
| Q9BV86 | Methyltransferase-like protein 11A OS=Homo sapiens GN=METTL11A PE=1 SV=3 - [ME11A_HUMAN] | 2 | 2 | 2 | 223 | 25.4 | 5.52 |
| P15153 | Ras-related C3 botulinum toxin substrate 2 OS=Homo sapiens GN=RAC2 PE=1 SV=1 - [RAC2_HUMAN] | 2 | 2 | 2 | 192 | 21.4 | 7.61 |
| P23381 | Tryptophanyl-tRNA synthetase. cytoplasmic OS=Homo sapiens GN=WARS PE=1 SV=2 - [SYWC_HUMAN] | 2 | 1 | 2 | 471 | 53.1 | 6.23 |
| Q4G0N4 | UPF0465 protein C5orf33 OS=Homo sapiens GN=C5orf33 PE=1 SV=2 - [CE033_HUMAN] | 2 | 1 | 2 | 442 | 49.4 | 8.18 |
| O14828 | Secretory carrier-associated membrane protein 3 OS=Homo sapiens GN=SCAMP3 PE=1 SV=3 - [SCAM3_HUMAN] | 2 | 1 | 2 | 347 | 38.3 | 7.64 |
| Q9UKX7 | Nuclear pore complex protein Nup50 OS=Homo sapiens GN=NUP50 PE=1 SV=2 - [NUP50_HUMAN] | 2 | 2 | 1 | 468 | 50.1 | 7.06 |
| P84098 | 60S ribosomal protein L19 OS=Homo sapiens GN=RPL19 PE=1 SV=1 - [RL19_HUMAN] | 2 | 2 | 2 | 196 | 23.5 | 11.47 |
| Q8IYS1 | Peptidase M20 domain-containing protein 2 OS=Homo sapiens GN=PM20D2 PE=1 SV=2 - [P20D2_HUMAN] | 2 |  | 2 | 436 | 47.7 | 5.85 |
| Q9NRX2 | 39S ribosomal protein L17. mitochondrial OS=Homo sapiens GN=MRPL17 PE=1 SV=1 - [RM17_HUMAN] | 2 | 2 | 2 | 175 | 20.0 | 10.11 |
| Q9NVX2 | Notchless protein homolog 1 OS=Homo sapiens GN=NLE1 PE=1 SV=4 - [NLE1_HUMAN] | 2 | 3 | 2 | 485 | 53.3 | 7.34 |
| P08263 | Glutathione S-transferase A1 OS=Homo sapiens GN=GSTA1 PE=1 SV=3 - [GSTA1_HUMAN] | 2 | 3 | 3 | 222 | 25.6 | 8.88 |
| P53597 | Succinate--CoA ligase [ADP/GDP-forming] subunit alpha. mitochondrial OS=Homo sapiens GN=SUCLG1 PE=1 SV=4 - [SUCA_HUMAN] | 2 | 1 | 2 | 346 | 36.2 | 8.79 |
| P49366 | Deoxyhypusine synthase OS=Homo sapiens GN=DHPS PE=1 SV=1 - [DHYS_HUMAN] | 2 | 1 | 3 | 369 | 40.9 | 5.36 |
| Q9H3N1 | Thioredoxin-related transmembrane protein 1 OS=Homo sapiens GN=TMX1 PE=1 SV=1 - [TMX1_HUMAN] | 2 | 2 | 1 | 280 | 31.8 | 4.98 |
| P16422 | Epithelial cell adhesion molecule OS=Homo sapiens GN=EPCAM PE=1 SV=2 - [EPCAM_HUMAN] | 2 | 1 | 2 | 314 | 34.9 | 7.46 |
| Q9P289 | Serine/threonine-protein kinase MST4 OS=Homo sapiens GN=MST4 PE=1 SV=2 - [MST4_HUMAN] | 2 | 1 | 2 | 416 | 46.5 | 5.29 |
| Q9UBU8 | Mortality factor 4-like protein 1 OS=Homo sapiens GN=MORF4L1 PE=1 SV=2 - [MO4L1_HUMAN] | 2 | 2 | 3 | 362 | 41.4 | 9.28 |
| Q9H8S9 | MOB kinase activator 1A OS=Homo sapiens GN=MOB1A PE=1 SV=4 - [MOB1A_HUMAN] | 2 | 2 | 2 | 216 | 25.1 | 6.95 |
| Q13619 | Cullin-4A OS=Homo sapiens GN=CUL4A PE=1 SV=3 - [CUL4A_HUMAN] | 2 | 5 | 5 | 759 | 87.6 | 8.13 |
| Q96KB5 | Lymphokine-activated killer T-cell-originated protein kinase OS=Homo sapiens GN=PBK PE=1 SV=3 - [TOPK_HUMAN] | 2 |  | 2 | 322 | 36.1 | 5.12 |
| Q15014 | Mortality factor 4-like protein 2 OS=Homo sapiens GN=MORF4L2 PE=1 SV=1 - [MO4L2_HUMAN] | 2 | 1 | 2 | 288 | 32.3 | 9.72 |
| P25685 | DnaJ homolog subfamily B member 1 OS=Homo sapiens GN=DNAJB1 PE=1 SV=4 - [DNJB1_HUMAN] | 2 | 1 | 2 | 340 | 38.0 | 8.63 |
| Q9BVK6 | Transmembrane emp24 domain-containing protein 9 OS=Homo sapiens GN=TMED9 PE=1 SV=2 - [TMED9_HUMAN] | 2 | 2 | 2 | 235 | 27.3 | 8.02 |
| Q96C23 | Aldose 1-epimerase OS=Homo sapiens GN=GALM PE=1 SV=1 - [GALM_HUMAN] | 2 | 2 | 2 | 342 | 37.7 | 6.65 |
| Q15006 | Tetratricopeptide repeat protein 35 OS=Homo sapiens GN=TTC35 PE=1 SV=1 - [TTC35_HUMAN] | 2 | 2 | 2 | 297 | 34.8 | 6.57 |
| Q15738 | Sterol-4-alpha-carboxylate 3-dehydrogenase. decarboxylating OS=Homo sapiens GN=NSDHL PE=1 SV=2 - [NSDHL_HUMAN] | 2 | 2 | 3 | 373 | 41.9 | 8.06 |
| A6NH11 | Glycolipid transfer protein domain-containing protein 2 OS=Homo sapiens OX=9606 GN=GLTPD2 PE=1 SV=2 - [GLTD2_HUMAN] | 2 | 1 | 2 | 291 | 31.6 | 10.15 |
| Q00535 | Cell division protein kinase 5 OS=Homo sapiens GN=CDK5 PE=1 SV=3 - [CDK5_HUMAN] | 2 | 4 | 3 | 292 | 33.3 | 7.66 |
| P21912 | Succinate dehydrogenase [ubiquinone] iron-sulfur subunit. mitochondrial OS=Homo sapiens GN=SDHB PE=1 SV=3 - [DHSB_HUMAN] | 2 | 2 | 2 | 280 | 31.6 | 8.76 |
| Q9P2R7 | Succinyl-CoA ligase [ADP-forming] subunit beta. mitochondrial OS=Homo sapiens GN=SUCLA2 PE=1 SV=3 - [SUCB1_HUMAN] | 2 | 1 | 1 | 463 | 50.3 | 7.42 |
| O60832 | H/ACA ribonucleoprotein complex subunit 4 OS=Homo sapiens GN=DKC1 PE=1 SV=3 - [DKC1_HUMAN] | 2 | 1 | 2 | 514 | 57.6 | 9.42 |
| Q13595 | Transformer-2 protein homolog alpha OS=Homo sapiens GN=TRA2A PE=1 SV=1 - [TRA2A_HUMAN] | 2 | 2 | 1 | 282 | 32.7 | 11.27 |
| Q53FA7 | Quinone oxidoreductase PIG3 OS=Homo sapiens GN=TP53I3 PE=1 SV=2 - [QORX_HUMAN] | 2 | 1 | 2 | 332 | 35.5 | 7.17 |
| O95674 | Phosphatidate cytidylyltransferase 2 OS=Homo sapiens GN=CDS2 PE=1 SV=1 - [CDS2_HUMAN] | 2 | 2 | 1 | 445 | 51.4 | 7.09 |
| Q03426 | Mevalonate kinase OS=Homo sapiens GN=MVK PE=1 SV=1 - [KIME_HUMAN] | 2 | 1 | 2 | 396 | 42.4 | 6.46 |
| P42785 | Lysosomal Pro-X carboxypeptidase OS=Homo sapiens GN=PRCP PE=1 SV=1 - [PCP_HUMAN] | 2 | 2 | 2 | 496 | 55.8 | 7.21 |
| Q6DKJ4 | Nucleoredoxin OS=Homo sapiens GN=NXN PE=1 SV=2 - [NXN_HUMAN] | 2 | 2 | 2 | 435 | 48.4 | 4.97 |
| Q16222 | UDP-N-acetylhexosamine pyrophosphorylase OS=Homo sapiens GN=UAP1 PE=1 SV=3 - [UAP1_HUMAN] | 2 | 1 | 3 | 522 | 58.7 | 6.33 |
| Q9H490 | Phosphatidylinositol glycan anchor biosynthesis class U protein OS=Homo sapiens GN=PIGU PE=1 SV=3 - [PIGU_HUMAN] | 2 | 2 | 2 | 435 | 50.0 | 7.72 |
| P43307 | Translocon-associated protein subunit alpha OS=Homo sapiens GN=SSR1 PE=1 SV=3 - [SSRA_HUMAN] | 2 | 3 | 2 | 286 | 32.2 | 4.49 |
| O14618 | Copper chaperone for superoxide dismutase OS=Homo sapiens GN=CCS PE=1 SV=1 - [CCS_HUMAN] | 2 | 2 | 1 | 274 | 29.0 | 5.58 |
| Q5VWZ2 | Lysophospholipase-like protein 1 OS=Homo sapiens GN=LYPLAL1 PE=1 SV=3 - [LYPL1_HUMAN] | 2 |  | 2 | 237 | 26.3 | 7.84 |
| P35914 | Hydroxymethylglutaryl-CoA lyase. mitochondrial OS=Homo sapiens GN=HMGCL PE=1 SV=2 - [HMGCL_HUMAN] | 2 | 2 | 2 | 325 | 34.3 | 8.54 |
| Q15654 | Thyroid receptor-interacting protein 6 OS=Homo sapiens GN=TRIP6 PE=1 SV=3 - [TRIP6_HUMAN] | 2 | 1 | 2 | 476 | 50.3 | 7.37 |
| Q7L5D6 | Golgi to ER traffic protein 4 homolog OS=Homo sapiens GN=GET4 PE=1 SV=1 - [GET4_HUMAN] | 2 | 1 | 3 | 327 | 36.5 | 5.41 |
| Q96C19 | EF-hand domain-containing protein D2 OS=Homo sapiens GN=EFHD2 PE=1 SV=1 - [EFHD2_HUMAN] | 2 | 2 | 2 | 240 | 26.7 | 5.20 |
| Q9H0S4 | Probable ATP-dependent RNA helicase DDX47 OS=Homo sapiens GN=DDX47 PE=1 SV=1 - [DDX47_HUMAN] | 2 | 1 | 2 | 455 | 50.6 | 9.10 |
| P30520 | Adenylosuccinate synthetase isozyme 2 OS=Homo sapiens GN=ADSS PE=1 SV=3 - [PURA2_HUMAN] | 2 | 2 | 2 | 456 | 50.1 | 6.55 |
| Q9BW60 | Elongation of very long chain fatty acids protein 1 OS=Homo sapiens GN=ELOVL1 PE=1 SV=1 - [ELOV1_HUMAN] | 2 | 1 | 2 | 279 | 32.6 | 9.60 |
| Q96F86 | Enhancer of mRNA-decapping protein 3 OS=Homo sapiens GN=EDC3 PE=1 SV=1 - [EDC3_HUMAN] | 2 | 1 | 2 | 508 | 56.0 | 7.11 |
| Q53GS9 | U4/U6.U5 tri-snRNP-associated protein 2 OS=Homo sapiens GN=USP39 PE=1 SV=2 - [SNUT2_HUMAN] | 2 | 2 | 1 | 565 | 65.3 | 8.91 |
| O43657 | Tetraspanin-6 OS=Homo sapiens GN=TSPAN6 PE=1 SV=1 - [TSN6_HUMAN] | 2 | 1 | 1 | 245 | 27.5 | 8.10 |
| Q9NQZ2 | Something about silencing protein 10 OS=Homo sapiens GN=UTP3 PE=1 SV=1 - [SAS10_HUMAN] | 2 | 2 |  | 479 | 54.5 | 5.62 |
| P08574 | Cytochrome c1. heme protein. mitochondrial OS=Homo sapiens GN=CYC1 PE=1 SV=3 - [CY1_HUMAN] | 2 | 2 | 3 | 325 | 35.4 | 9.00 |
| Q96EP5 | DAZ-associated protein 1 OS=Homo sapiens GN=DAZAP1 PE=1 SV=1 - [DAZP1_HUMAN] | 2 | 2 | 1 | 407 | 43.4 | 8.56 |
| Q9UHK6 | Alpha-methylacyl-CoA racemase OS=Homo sapiens OX=9606 GN=AMACR PE=1 SV=2 - [AMACR_HUMAN] | 2 | 2 | 2 | 382 | 42.4 | 6.44 |
| Q6UW02 | Cytochrome P450 20A1 OS=Homo sapiens GN=CYP20A1 PE=1 SV=1 - [CP20A_HUMAN] | 2 | 2 | 1 | 462 | 52.4 | 6.21 |
| Q9BSH5 | Haloacid dehalogenase-like hydrolase domain-containing protein 3 OS=Homo sapiens GN=HDHD3 PE=1 SV=1 - [HDHD3_HUMAN] | 2 | 2 | 2 | 251 | 28.0 | 6.71 |
| O15143 | Actin-related protein 2/3 complex subunit 1B OS=Homo sapiens GN=ARPC1B PE=1 SV=3 - [ARC1B_HUMAN] | 2 | 2 | 2 | 372 | 40.9 | 8.35 |
| O43237 | Cytoplasmic dynein 1 light intermediate chain 2 OS=Homo sapiens GN=DYNC1LI2 PE=1 SV=1 - [DC1L2_HUMAN] | 2 | 2 | 2 | 492 | 54.1 | 6.38 |
| Q8IZ81 | ELMO domain-containing protein 2 OS=Homo sapiens OX=9606 GN=ELMOD2 PE=1 SV=1 - [ELMD2_HUMAN] | 2 |  | 2 | 293 | 34.9 | 8.05 |
| P00403 | Cytochrome c oxidase subunit 2 OS=Homo sapiens GN=MT-CO2 PE=1 SV=1 - [COX2_HUMAN] | 2 | 1 | 2 | 227 | 25.5 | 4.82 |
| Q99638 | Cell cycle checkpoint control protein RAD9A OS=Homo sapiens GN=RAD9A PE=1 SV=1 - [RAD9A_HUMAN] | 2 | 2 |  | 391 | 42.5 | 5.66 |
| Q9BYD6 | 39S ribosomal protein L1. mitochondrial OS=Homo sapiens GN=MRPL1 PE=1 SV=2 - [RM01_HUMAN] | 2 | 1 | 1 | 325 | 36.9 | 8.78 |
| P10909 | Clusterin OS=Homo sapiens GN=CLU PE=1 SV=1 - [CLUS_HUMAN] | 2 |  | 2 | 449 | 52.5 | 6.27 |
| Q8IYS2 | Uncharacterized protein KIAA2013 OS=Homo sapiens GN=KIAA2013 PE=1 SV=1 - [K2013_HUMAN] | 2 | 1 | 2 | 634 | 69.1 | 8.19 |
| O00625 | Pirin OS=Homo sapiens GN=PIR PE=1 SV=1 - [PIR_HUMAN] | 2 | 2 | 2 | 290 | 32.1 | 6.92 |
| Q9BXK5 | Bcl-2-like protein 13 OS=Homo sapiens GN=BCL2L13 PE=1 SV=1 - [B2L13_HUMAN] | 2 | 1 | 2 | 485 | 52.7 | 4.44 |
| P07306 | Asialoglycoprotein receptor 1 OS=Homo sapiens GN=ASGR1 PE=1 SV=2 - [ASGR1_HUMAN] | 2 | 1 | 2 | 291 | 33.2 | 5.50 |
| P40121 | Macrophage-capping protein OS=Homo sapiens GN=CAPG PE=1 SV=2 - [CAPG_HUMAN] | 2 | 2 | 1 | 348 | 38.5 | 6.19 |
| P06865 | Beta-hexosaminidase subunit alpha OS=Homo sapiens GN=HEXA PE=1 SV=2 - [HEXA_HUMAN] | 2 | 2 | 2 | 529 | 60.7 | 5.16 |
| O95551 | Tyrosyl-DNA phosphodiesterase 2 OS=Homo sapiens GN=TDP2 PE=1 SV=1 - [TYDP2_HUMAN] | 2 | 1 | 2 | 362 | 40.9 | 5.10 |
| Q52LJ0 | Protein FAM98B OS=Homo sapiens OX=9606 GN=FAM98B PE=1 SV=2 - [FA98B_HUMAN] | 2 |  | 2 | 433 | 45.5 | 8.69 |
| Q9Y2Z4 | Tyrosyl-tRNA synthetase. mitochondrial OS=Homo sapiens GN=YARS2 PE=1 SV=2 - [SYYM_HUMAN] | 2 | 1 | 2 | 477 | 53.2 | 8.98 |
| P82673 | 28S ribosomal protein S35. mitochondrial OS=Homo sapiens GN=MRPS35 PE=1 SV=1 - [RT35_HUMAN] | 2 | 1 | 2 | 323 | 36.8 | 8.24 |
| P33240 | Cleavage stimulation factor subunit 2 OS=Homo sapiens GN=CSTF2 PE=1 SV=1 - [CSTF2_HUMAN] | 2 | 2 | 3 | 577 | 60.9 | 6.83 |
| Q9UN81 | LINE-1 retrotransposable element ORF1 protein OS=Homo sapiens GN=L1RE1 PE=1 SV=1 - [LORF1_HUMAN] | 2 | 1 | 2 | 338 | 40.0 | 9.51 |
| Q9NX61 | Transmembrane protein 161A OS=Homo sapiens GN=TMEM161A PE=1 SV=1 - [T161A_HUMAN] | 2 | 2 | 2 | 479 | 53.6 | 8.44 |
| Q3KQV9 | UDP-N-acetylhexosamine pyrophosphorylase-like protein 1 OS=Homo sapiens GN=UAP1L1 PE=2 SV=2 - [UAP1L_HUMAN] | 2 | 4 | 3 | 507 | 57.0 | 6.32 |
| P04818 | Thymidylate synthase OS=Homo sapiens GN=TYMS PE=1 SV=3 - [TYSY_HUMAN] | 2 | 2 | 2 | 313 | 35.7 | 7.01 |
| P13688 | Carcinoembryonic antigen-related cell adhesion molecule 1 OS=Homo sapiens GN=CEACAM1 PE=1 SV=2 - [CEAM1_HUMAN] | 2 |  | 2 | 526 | 57.5 | 5.97 |
| P15170 | Eukaryotic peptide chain release factor GTP-binding subunit ERF3A OS=Homo sapiens GN=GSPT1 PE=1 SV=1 - [ERF3A_HUMAN] | 2 | 1 | 2 | 499 | 55.7 | 5.62 |
| Q5VIR6 | Vacuolar protein sorting-associated protein 53 homolog OS=Homo sapiens GN=VPS53 PE=1 SV=1 - [VPS53_HUMAN] | 2 | 2 |  | 699 | 79.6 | 6.02 |
| Q96P11 | Putative methyltransferase NSUN5 OS=Homo sapiens GN=NSUN5 PE=1 SV=2 - [NSUN5_HUMAN] | 2 | 3 | 1 | 429 | 46.7 | 8.62 |
| Q9UJ70 | N-acetyl-D-glucosamine kinase OS=Homo sapiens GN=NAGK PE=1 SV=4 - [NAGK_HUMAN] | 2 | 1 | 1 | 344 | 37.4 | 6.24 |
| Q9BQ67 | Glutamate-rich WD repeat-containing protein 1 OS=Homo sapiens GN=GRWD1 PE=1 SV=1 - [GRWD1_HUMAN] | 2 | 3 | 3 | 446 | 49.4 | 4.92 |
| O60749 | Sorting nexin-2 OS=Homo sapiens GN=SNX2 PE=1 SV=2 - [SNX2_HUMAN] | 2 | 2 | 2 | 519 | 58.4 | 5.12 |
| Q7Z2H8 | Proton-coupled amino acid transporter 1 OS=Homo sapiens GN=SLC36A1 PE=1 SV=1 - [S36A1_HUMAN] | 2 | 1 | 2 | 476 | 53.0 | 6.99 |
| P82930 | 28S ribosomal protein S34. mitochondrial OS=Homo sapiens GN=MRPS34 PE=1 SV=2 - [RT34_HUMAN] | 2 | 2 | 1 | 218 | 25.6 | 9.98 |
| Q9H7B2 | Ribosome production factor 2 homolog OS=Homo sapiens GN=RPF2 PE=1 SV=2 - [RPF2_HUMAN] | 2 | 1 | 2 | 306 | 35.6 | 9.99 |
| P42357 | Histidine ammonia-lyase OS=Homo sapiens GN=HAL PE=1 SV=1 - [HUTH_HUMAN] | 2 | 1 | 2 | 657 | 72.7 | 6.95 |
| Q96S52 | GPI transamidase component PIG-S OS=Homo sapiens GN=PIGS PE=1 SV=3 - [PIGS_HUMAN] | 2 | 1 | 2 | 555 | 61.6 | 6.49 |
| P35680 | Hepatocyte nuclear factor 1-beta OS=Homo sapiens GN=HNF1B PE=1 SV=1 - [HNF1B_HUMAN] | 2 | 2 | 2 | 557 | 61.3 | 7.77 |
| Q969G3 | SWI/SNF-related matrix-associated actin-dependent regulator of chromatin subfamily E member 1 OS=Homo sapiens GN=SMARCE1 PE=1 SV=2 - [SMCE1_HUMAN] | 2 | 1 | 2 | 411 | 46.6 | 4.88 |
| Q9Y3B7 | 39S ribosomal protein L11. mitochondrial OS=Homo sapiens GN=MRPL11 PE=1 SV=1 - [RM11_HUMAN] | 2 |  | 2 | 192 | 20.7 | 9.91 |
| P08240 | Signal recognition particle receptor subunit alpha OS=Homo sapiens GN=SRPR PE=1 SV=2 - [SRPR_HUMAN] | 2 | 2 | 1 | 638 | 69.8 | 8.95 |
| P35610 | Sterol O-acyltransferase 1 OS=Homo sapiens GN=SOAT1 PE=1 SV=3 - [SOAT1_HUMAN] | 2 | 2 | 2 | 550 | 64.7 | 8.94 |
| P30533 | Alpha-2-macroglobulin receptor-associated protein OS=Homo sapiens GN=LRPAP1 PE=1 SV=1 - [AMRP_HUMAN] | 2 | 2 | 1 | 357 | 41.4 | 8.78 |
| Q9BZ23 | Pantothenate kinase 2. mitochondrial OS=Homo sapiens GN=PANK2 PE=1 SV=3 - [PANK2_HUMAN] | 2 | 2 | 1 | 570 | 62.6 | 9.28 |
| Q9NQ88 | Probable fructose-2.6-bisphosphatase TIGAR OS=Homo sapiens GN=TIGAR PE=1 SV=1 - [TIGAR_HUMAN] | 2 | 1 | 1 | 270 | 30.0 | 7.69 |
| Q15397 | Pumilio domain-containing protein KIAA0020 OS=Homo sapiens GN=KIAA0020 PE=1 SV=3 - [K0020_HUMAN] | 2 | 2 | 2 | 648 | 73.5 | 9.64 |
| Q8N1B4 | Vacuolar protein sorting-associated protein 52 homolog OS=Homo sapiens GN=VPS52 PE=1 SV=1 - [VPS52_HUMAN] | 2 | 2 | 1 | 723 | 82.2 | 5.99 |
| P51114 | Fragile X mental retardation syndrome-related protein 1 OS=Homo sapiens GN=FXR1 PE=1 SV=3 - [FXR1_HUMAN] | 2 | 3 | 5 | 621 | 69.7 | 6.15 |
| P55039 | Developmentally-regulated GTP-binding protein 2 OS=Homo sapiens GN=DRG2 PE=1 SV=1 - [DRG2_HUMAN] | 2 | 1 | 1 | 364 | 40.7 | 8.88 |
| Q8TB37 | Iron-sulfur protein NUBPL OS=Homo sapiens OX=9606 GN=NUBPL PE=1 SV=3 - [NUBPL_HUMAN] | 2 | 1 | 2 | 319 | 34.1 | 9.04 |
| O00442 | RNA 3'-terminal phosphate cyclase OS=Homo sapiens GN=RTCD1 PE=1 SV=1 - [RTC1_HUMAN] | 2 |  | 2 | 366 | 39.3 | 7.85 |
| Q9UM21 | Alpha-1.3-mannosyl-glycoprotein 4-beta-N-acetylglucosaminyltransferase A OS=Homo sapiens GN=MGAT4A PE=1 SV=1 - [MGT4A_HUMAN] | 2 | 1 | 2 | 535 | 61.5 | 7.40 |
| Q9C0B1 | Protein fto OS=Homo sapiens GN=FTO PE=1 SV=3 - [FTO_HUMAN] | 2 | 2 | 1 | 505 | 58.2 | 5.22 |
| Q15043 | Zinc transporter ZIP14 OS=Homo sapiens GN=SLC39A14 PE=1 SV=3 - [S39AE_HUMAN] | 2 | 1 | 1 | 492 | 54.2 | 5.33 |
| O75306 | NADH dehydrogenase [ubiquinone] iron-sulfur protein 2. mitochondrial OS=Homo sapiens GN=NDUFS2 PE=1 SV=2 - [NDUS2_HUMAN] | 2 | 1 | 2 | 463 | 52.5 | 7.55 |
| Q9H1I8 | Activating signal cointegrator 1 complex subunit 2 OS=Homo sapiens GN=ASCC2 PE=1 SV=3 - [ASCC2_HUMAN] | 2 | 2 | 2 | 757 | 86.3 | 5.16 |
| P28300 | Protein-lysine 6-oxidase OS=Homo sapiens GN=LOX PE=1 SV=2 - [LYOX_HUMAN] | 2 | 1 | 1 | 417 | 46.9 | 8.09 |
| Q99836 | Myeloid differentiation primary response protein MyD88 OS=Homo sapiens GN=MYD88 PE=1 SV=1 - [MYD88_HUMAN] | 2 |  | 2 | 296 | 33.2 | 6.15 |
| P51654 | Glypican-3 OS=Homo sapiens GN=GPC3 PE=1 SV=1 - [GPC3_HUMAN] | 2 | 2 | 1 | 580 | 65.5 | 6.37 |
| Q9Y223 | Bifunctional UDP-N-acetylglucosamine 2-epimerase/N-acetylmannosamine kinase OS=Homo sapiens GN=GNE PE=1 SV=1 - [GLCNE_HUMAN] | 2 | 1 | 2 | 722 | 79.2 | 6.80 |
| Q01650 | Large neutral amino acids transporter small subunit 1 OS=Homo sapiens GN=SLC7A5 PE=1 SV=2 - [LAT1_HUMAN] | 2 | 2 |  | 507 | 55.0 | 7.72 |
| P40123 | Adenylyl cyclase-associated protein 2 OS=Homo sapiens GN=CAP2 PE=1 SV=1 - [CAP2_HUMAN] | 2 | 2 | 3 | 477 | 52.8 | 6.37 |
| P35813 | Protein phosphatase 1A OS=Homo sapiens GN=PPM1A PE=1 SV=1 - [PPM1A_HUMAN] | 2 |  | 2 | 382 | 42.4 | 5.36 |
| P14678 | Small nuclear ribonucleoprotein-associated proteins B and B' OS=Homo sapiens GN=SNRPB PE=1 SV=2 - [RSMB_HUMAN] | 2 | 3 | 3 | 240 | 24.6 | 11.19 |
| P02679 | Fibrinogen gamma chain OS=Homo sapiens GN=FGG PE=1 SV=3 - [FIBG_HUMAN] | 2 | 1 | 2 | 453 | 51.5 | 5.62 |
| Q9HD45 | Transmembrane 9 superfamily member 3 OS=Homo sapiens GN=TM9SF3 PE=1 SV=2 - [TM9S3_HUMAN] | 2 | 3 | 3 | 589 | 67.8 | 7.21 |
| P55036 | 26S proteasome non-ATPase regulatory subunit 4 OS=Homo sapiens GN=PSMD4 PE=1 SV=1 - [PSMD4_HUMAN] | 2 | 1 | 2 | 377 | 40.7 | 4.79 |
| Q3SXM5 | Inactive hydroxysteroid dehydrogenase-like protein 1 OS=Homo sapiens GN=HSDL1 PE=1 SV=3 - [HSDL1_HUMAN] | 2 | 2 | 1 | 330 | 37.0 | 8.72 |
| P54802 | Alpha-N-acetylglucosaminidase OS=Homo sapiens GN=NAGLU PE=1 SV=2 - [ANAG_HUMAN] | 2 | 1 | 4 | 743 | 82.2 | 6.65 |
| O00273 | DNA fragmentation factor subunit alpha OS=Homo sapiens GN=DFFA PE=1 SV=1 - [DFFA_HUMAN] | 2 | 2 |  | 331 | 36.5 | 4.79 |
| Q9H2C0 | Gigaxonin OS=Homo sapiens GN=GAN PE=1 SV=1 - [GAN_HUMAN] | 2 | 2 | 2 | 597 | 67.6 | 5.85 |
| O43505 | Beta-1.4-glucuronyltransferase 1 OS=Homo sapiens GN=B4GAT1 PE=1 SV=1 - [B4GA1_HUMAN] | 2 | 1 | 1 | 415 | 47.1 | 7.20 |
| Q13505 | Metaxin-1 OS=Homo sapiens OX=9606 GN=MTX1 PE=1 SV=3 - [MTX1_HUMAN] | 2 | 2 | 2 | 466 | 51.4 | 9.79 |
| Q8IXI1 | Mitochondrial Rho GTPase 2 OS=Homo sapiens GN=RHOT2 PE=1 SV=2 - [MIRO2_HUMAN] | 2 | 2 | 1 | 618 | 68.1 | 5.86 |
| Q2Q1W2 | Tripartite motif-containing protein 71 OS=Homo sapiens GN=TRIM71 PE=2 SV=1 - [LIN41_HUMAN] | 2 |  | 2 | 868 | 93.3 | 7.61 |
| Q13501 | Sequestosome-1 OS=Homo sapiens GN=SQSTM1 PE=1 SV=1 - [SQSTM_HUMAN] | 2 | 2 | 2 | 440 | 47.7 | 5.22 |
| P11474 | Steroid hormone receptor ERR1 OS=Homo sapiens GN=ESRRA PE=1 SV=3 - [ERR1_HUMAN] | 2 | 2 | 1 | 423 | 45.5 | 6.38 |
| Q9NVH1 | DnaJ homolog subfamily C member 11 OS=Homo sapiens GN=DNAJC11 PE=1 SV=2 - [DJC11_HUMAN] | 2 | 1 | 2 | 559 | 63.2 | 8.40 |
| O43818 | U3 small nucleolar RNA-interacting protein 2 OS=Homo sapiens GN=RRP9 PE=1 SV=1 - [U3IP2_HUMAN] | 2 | 3 | 2 | 475 | 51.8 | 7.85 |
| Q5T6V5 | UPF0553 protein C9orf64 OS=Homo sapiens GN=C9orf64 PE=1 SV=1 - [CI064_HUMAN] | 2 | 2 | 2 | 341 | 39.0 | 5.88 |
| Q9NRG9 | Aladin OS=Homo sapiens GN=AAAS PE=1 SV=1 - [AAAS_HUMAN] | 2 | 1 | 2 | 546 | 59.5 | 7.50 |
| Q14919 | Dr1-associated corepressor OS=Homo sapiens GN=DRAP1 PE=1 SV=3 - [NC2A_HUMAN] | 2 | 1 | 1 | 205 | 22.3 | 5.17 |
| Q9BVI4 | Nucleolar complex protein 4 homolog OS=Homo sapiens GN=NOC4L PE=1 SV=1 - [NOC4L_HUMAN] | 2 | 2 | 2 | 516 | 58.4 | 7.49 |
| Q16647 | Prostacyclin synthase OS=Homo sapiens GN=PTGIS PE=1 SV=1 - [PTGIS_HUMAN] | 2 | 1 | 1 | 500 | 57.1 | 7.31 |
| Q12765 | Secernin-1 OS=Homo sapiens GN=SCRN1 PE=1 SV=2 - [SCRN1_HUMAN] | 2 | 2 | 1 | 414 | 46.4 | 4.75 |
| Q9HAB8 | Phosphopantothenate--cysteine ligase OS=Homo sapiens GN=PPCS PE=1 SV=2 - [PPCS_HUMAN] | 2 |  | 2 | 311 | 34.0 | 6.71 |
| Q13884 | Beta-1-syntrophin OS=Homo sapiens GN=SNTB1 PE=1 SV=3 - [SNTB1_HUMAN] | 2 | 1 | 1 | 538 | 58.0 | 8.63 |
| Q8N8S7 | Protein enabled homolog OS=Homo sapiens GN=ENAH PE=1 SV=2 - [ENAH_HUMAN] | 2 | 1 | 2 | 591 | 66.5 | 6.93 |
| A8MUH7 | Putative PDZ domain-containing protein PDZK1P1 OS=Homo sapiens GN=PDZK1P1 PE=5 SV=2 - [PDZ1P_HUMAN] | 2 |  | 2 | 402 | 44.0 | 5.63 |
| Q6UWE0 | E3 ubiquitin-protein ligase LRSAM1 OS=Homo sapiens GN=LRSAM1 PE=1 SV=1 - [LRSM1_HUMAN] | 2 | 1 | 2 | 723 | 83.5 | 5.94 |
| O43772 | Mitochondrial carnitine/acylcarnitine carrier protein OS=Homo sapiens GN=SLC25A20 PE=1 SV=1 - [MCAT_HUMAN] | 2 | 1 | 2 | 301 | 32.9 | 9.41 |
| Q8IY67 | Ribonucleoprotein PTB-binding 1 OS=Homo sapiens GN=RAVER1 PE=1 SV=1 - [RAVR1_HUMAN] | 2 | 1 | 1 | 606 | 63.8 | 8.48 |
| P49589 | Cysteinyl-tRNA synthetase. cytoplasmic OS=Homo sapiens GN=CARS PE=1 SV=3 - [SYCC_HUMAN] | 2 | 3 | 1 | 748 | 85.4 | 6.76 |
| O95260 | Arginyl-tRNA--protein transferase 1 OS=Homo sapiens GN=ATE1 PE=1 SV=2 - [ATE1_HUMAN] | 2 | 2 | 2 | 518 | 59.1 | 7.93 |
| Q969Z0 | Protein TBRG4 OS=Homo sapiens GN=TBRG4 PE=1 SV=1 - [TBRG4_HUMAN] | 2 | 2 | 2 | 631 | 70.7 | 7.42 |
| Q9UID3 | Protein fat-free homolog OS=Homo sapiens GN=FFR PE=1 SV=2 - [FFR_HUMAN] | 2 | 1 | 1 | 782 | 86.0 | 6.47 |
| Q9BWE0 | Replication initiator 1 OS=Homo sapiens GN=REPIN1 PE=1 SV=1 - [REPI1_HUMAN] | 2 |  | 2 | 567 | 63.5 | 9.98 |
| Q16186 | Proteasomal ubiquitin receptor ADRM1 OS=Homo sapiens GN=ADRM1 PE=1 SV=2 - [ADRM1_HUMAN] | 2 | 2 | 2 | 407 | 42.1 | 5.07 |
| O75351 | Vacuolar protein sorting-associated protein 4B OS=Homo sapiens GN=VPS4B PE=1 SV=2 - [VPS4B_HUMAN] | 2 |  | 2 | 444 | 49.3 | 7.23 |
| Q9H0U3 | Magnesium transporter protein 1 OS=Homo sapiens GN=MAGT1 PE=1 SV=1 - [MAGT1_HUMAN] | 2 | 1 | 1 | 335 | 38.0 | 9.63 |
| P37198 | Nuclear pore glycoprotein p62 OS=Homo sapiens GN=NUP62 PE=1 SV=3 - [NUP62_HUMAN] | 2 | 3 | 3 | 522 | 53.2 | 5.31 |
| Q969V3 | Nicalin OS=Homo sapiens GN=NCLN PE=1 SV=2 - [NCLN_HUMAN] | 2 | 3 | 1 | 563 | 62.9 | 6.89 |
| O95219 | Sorting nexin-4 OS=Homo sapiens GN=SNX4 PE=1 SV=1 - [SNX4_HUMAN] | 2 | 2 | 2 | 450 | 51.9 | 5.99 |
| Q9BV38 | WD repeat-containing protein 18 OS=Homo sapiens GN=WDR18 PE=1 SV=2 - [WDR18_HUMAN] | 2 | 1 | 2 | 432 | 47.4 | 6.70 |
| Q9UH99 | Protein unc-84 homolog B OS=Homo sapiens GN=UNC84B PE=1 SV=3 - [UN84B_HUMAN] | 2 | 1 | 2 | 717 | 80.3 | 6.73 |
| Q06265 | Exosome complex component RRP45 OS=Homo sapiens GN=EXOSC9 PE=1 SV=3 - [EXOS9_HUMAN] | 2 | 1 | 1 | 439 | 48.9 | 5.29 |
| Q9BUI4 | DNA-directed RNA polymerase III subunit RPC3 OS=Homo sapiens GN=POLR3C PE=1 SV=1 - [RPC3_HUMAN] | 2 | 2 |  | 534 | 60.6 | 7.31 |
| P49959 | Double-strand break repair protein MRE11A OS=Homo sapiens GN=MRE11A PE=1 SV=3 - [MRE11_HUMAN] | 2 |  | 2 | 708 | 80.5 | 5.90 |
| Q8WU90 | Zinc finger CCCH domain-containing protein 15 OS=Homo sapiens GN=ZC3H15 PE=1 SV=1 - [ZC3HF_HUMAN] | 2 | 2 | 2 | 426 | 48.6 | 5.31 |
| Q9H7D7 | WD repeat-containing protein 26 OS=Homo sapiens GN=WDR26 PE=1 SV=3 - [WDR26_HUMAN] | 2 | 1 | 2 | 661 | 72.1 | 6.16 |
| P41240 | Tyrosine-protein kinase CSK OS=Homo sapiens GN=CSK PE=1 SV=1 - [CSK_HUMAN] | 2 | 1 | 2 | 450 | 50.7 | 7.06 |
| P32780 | General transcription factor IIH subunit 1 OS=Homo sapiens GN=GTF2H1 PE=1 SV=1 - [TF2H1_HUMAN] | 2 |  | 2 | 548 | 62.0 | 8.66 |
| Q8IXH7 | Negative elongation factor C/D OS=Homo sapiens GN=TH1L PE=1 SV=2 - [NELFD_HUMAN] | 2 | 2 | 3 | 590 | 66.2 | 5.10 |
| Q8NFW8 | N-acylneuraminate cytidylyltransferase OS=Homo sapiens GN=CMAS PE=1 SV=2 - [NEUA_HUMAN] | 2 | 2 | 2 | 434 | 48.3 | 7.93 |
| Q7Z417 | Nuclear fragile X mental retardation-interacting protein 2 OS=Homo sapiens GN=NUFIP2 PE=1 SV=1 - [NUFP2_HUMAN] | 2 | 2 | 1 | 695 | 76.1 | 8.70 |
| Q01844 | RNA-binding protein EWS OS=Homo sapiens GN=EWSR1 PE=1 SV=1 - [EWS_HUMAN] | 2 | 2 | 2 | 656 | 68.4 | 9.33 |
| P15291 | Beta-1.4-galactosyltransferase 1 OS=Homo sapiens GN=B4GALT1 PE=1 SV=5 - [B4GT1_HUMAN] | 2 | 1 | 2 | 398 | 43.9 | 8.65 |
| Q9BQ70 | Transcription factor 25 OS=Homo sapiens GN=TCF25 PE=1 SV=1 - [TCF25_HUMAN] | 2 | 2 | 2 | 676 | 76.6 | 6.35 |
| O15228 | Dihydroxyacetone phosphate acyltransferase OS=Homo sapiens GN=GNPAT PE=1 SV=1 - [GNPAT_HUMAN] | 2 | 1 | 2 | 680 | 77.1 | 6.57 |
| O43172 | U4/U6 small nuclear ribonucleoprotein Prp4 OS=Homo sapiens GN=PRPF4 PE=1 SV=2 - [PRP4_HUMAN] | 2 | 1 | 2 | 522 | 58.4 | 7.42 |
| Q6NVY1 | 3-hydroxyisobutyryl-CoA hydrolase. mitochondrial OS=Homo sapiens GN=HIBCH PE=1 SV=2 - [HIBCH_HUMAN] | 2 | 2 | 2 | 386 | 43.5 | 8.19 |
| Q6NUM9 | All-trans-retinol 13.14-reductase OS=Homo sapiens GN=RETSAT PE=1 SV=2 - [RETST_HUMAN] | 2 | 2 | 1 | 610 | 66.8 | 8.28 |
| P49643 | DNA primase large subunit OS=Homo sapiens GN=PRIM2 PE=1 SV=2 - [PRI2_HUMAN] | 2 | 2 | 1 | 509 | 58.8 | 7.91 |
| Q9NW13 | RNA-binding protein 28 OS=Homo sapiens GN=RBM28 PE=1 SV=3 - [RBM28_HUMAN] | 2 | 1 | 1 | 759 | 85.7 | 9.22 |
| O43660 | Pleiotropic regulator 1 OS=Homo sapiens GN=PLRG1 PE=1 SV=1 - [PLRG1_HUMAN] | 2 | 2 |  | 514 | 57.2 | 9.17 |
| Q969N2 | GPI transamidase component PIG-T OS=Homo sapiens GN=PIGT PE=1 SV=1 - [PIGT_HUMAN] | 2 | 3 | 3 | 578 | 65.7 | 8.38 |
| Q71RC2 | La-related protein 4 OS=Homo sapiens GN=LARP4 PE=1 SV=3 - [LARP4_HUMAN] | 2 | 3 | 2 | 724 | 80.5 | 6.61 |
| Q9Y3T9 | Nucleolar complex protein 2 homolog OS=Homo sapiens GN=NOC2L PE=1 SV=4 - [NOC2L_HUMAN] | 2 |  | 2 | 749 | 84.9 | 5.62 |
| Q92905 | COP9 signalosome complex subunit 5 OS=Homo sapiens GN=COPS5 PE=1 SV=4 - [CSN5_HUMAN] | 2 | 2 | 1 | 334 | 37.6 | 6.54 |
| Q8TC07 | TBC1 domain family member 15 OS=Homo sapiens GN=TBC1D15 PE=1 SV=2 - [TBC15_HUMAN] | 2 | 1 | 2 | 691 | 79.4 | 5.67 |
| P36915 | Guanine nucleotide-binding protein-like 1 OS=Homo sapiens GN=GNL1 PE=1 SV=2 - [GNL1_HUMAN] | 2 | 2 | 1 | 607 | 68.6 | 5.80 |
| Q9NRW7 | Vacuolar protein sorting-associated protein 45 OS=Homo sapiens GN=VPS45 PE=1 SV=1 - [VPS45_HUMAN] | 2 | 2 | 2 | 570 | 65.0 | 8.24 |
| P32455 | Guanylate-binding protein 1 OS=Homo sapiens GN=GBP1 PE=1 SV=2 - [GBP1_HUMAN] | 2 | 2 | 1 | 592 | 67.9 | 6.32 |
| Q13573 | SNW domain-containing protein 1 OS=Homo sapiens GN=SNW1 PE=1 SV=1 - [SNW1_HUMAN] | 2 | 2 | 2 | 536 | 61.5 | 9.52 |
| Q86UE4 | Protein LYRIC OS=Homo sapiens GN=MTDH PE=1 SV=2 - [LYRIC_HUMAN] | 2 | 1 | 1 | 582 | 63.8 | 9.32 |
| Q9NRK6 | ATP-binding cassette sub-family B member 10. mitochondrial OS=Homo sapiens GN=ABCB10 PE=1 SV=2 - [ABCBA_HUMAN] | 2 |  | 2 | 738 | 79.1 | 9.85 |
| Q4G148 | Glucoside xylosyltransferase 1 OS=Homo sapiens GN=GXYLT1 PE=1 SV=2 - [GXLT1_HUMAN] | 2 | 2 | 2 | 440 | 50.5 | 8.65 |
| Q9H6R4 | Nucleolar protein 6 OS=Homo sapiens GN=NOL6 PE=1 SV=2 - [NOL6_HUMAN] | 2 | 1 | 2 | 1146 | 127.5 | 7.64 |
| P49189 | 4-trimethylaminobutyraldehyde dehydrogenase OS=Homo sapiens GN=ALDH9A1 PE=1 SV=3 - [AL9A1_HUMAN] | 2 | 2 | 2 | 494 | 53.8 | 5.87 |
| Q9UNH7 | Sorting nexin-6 OS=Homo sapiens GN=SNX6 PE=1 SV=1 - [SNX6_HUMAN] | 2 | 1 | 1 | 406 | 46.6 | 6.16 |
| Q03169 | Tumor necrosis factor alpha-induced protein 2 OS=Homo sapiens GN=TNFAIP2 PE=1 SV=2 - [TNAP2_HUMAN] | 2 | 3 | 2 | 654 | 72.6 | 6.46 |
| P55010 | Eukaryotic translation initiation factor 5 OS=Homo sapiens GN=EIF5 PE=1 SV=2 - [IF5_HUMAN] | 2 | 2 | 2 | 431 | 49.2 | 5.58 |
| Q6P3W7 | SCY1-like protein 2 OS=Homo sapiens GN=SCYL2 PE=1 SV=1 - [SCYL2_HUMAN] | 2 | 3 | 3 | 929 | 103.6 | 8.22 |
| Q8WWI5 | Choline transporter-like protein 1 OS=Homo sapiens GN=SLC44A1 PE=1 SV=1 - [CTL1_HUMAN] | 2 | 2 | 2 | 657 | 73.3 | 8.60 |
| Q9NTJ3 | Structural maintenance of chromosomes protein 4 OS=Homo sapiens GN=SMC4 PE=1 SV=2 - [SMC4_HUMAN] | 2 | 2 | 1 | 1288 | 147.1 | 6.79 |
| Q8WZA9 | Immunity-related GTPase family Q protein OS=Homo sapiens GN=IRGQ PE=1 SV=1 - [IRGQ_HUMAN] | 2 |  | 2 | 623 | 62.7 | 4.88 |
| Q14677 | Clathrin interactor 1 OS=Homo sapiens GN=CLINT1 PE=1 SV=1 - [EPN4_HUMAN] | 2 | 1 | 2 | 625 | 68.2 | 6.42 |
| O15254 | Peroxisomal acyl-coenzyme A oxidase 3 OS=Homo sapiens GN=ACOX3 PE=1 SV=2 - [ACOX3_HUMAN] | 2 | 1 | 1 | 700 | 77.6 | 7.25 |
| Q9BRZ2 | Tripartite motif-containing protein 56 OS=Homo sapiens GN=TRIM56 PE=1 SV=3 - [TRI56_HUMAN] | 2 | 2 | 1 | 755 | 81.4 | 7.74 |
| Q93034 | Cullin-5 OS=Homo sapiens GN=CUL5 PE=1 SV=4 - [CUL5_HUMAN] | 2 |  | 2 | 780 | 90.9 | 7.94 |
| Q7L0Y3 | Mitochondrial ribonuclease P protein 1 OS=Homo sapiens GN=RG9MTD1 PE=1 SV=2 - [MRRP1_HUMAN] | 2 |  | 2 | 403 | 47.3 | 9.36 |
| Q9BXB4 | Oxysterol-binding protein-related protein 11 OS=Homo sapiens GN=OSBPL11 PE=1 SV=2 - [OSB11_HUMAN] | 2 | 1 | 2 | 747 | 83.6 | 7.06 |
| Q92609 | TBC1 domain family member 5 OS=Homo sapiens GN=TBC1D5 PE=1 SV=1 - [TBCD5_HUMAN] | 2 | 1 | 3 | 795 | 88.9 | 6.54 |
| Q96CW1 | AP-2 complex subunit mu OS=Homo sapiens GN=AP2M1 PE=1 SV=2 - [AP2M1_HUMAN] | 2 |  | 2 | 435 | 49.6 | 9.54 |
| Q96JB2 | Conserved oligomeric Golgi complex subunit 3 OS=Homo sapiens GN=COG3 PE=1 SV=3 - [COG3_HUMAN] | 2 | 2 | 2 | 828 | 94.0 | 5.57 |
| P23786 | Carnitine O-palmitoyltransferase 2. mitochondrial OS=Homo sapiens GN=CPT2 PE=1 SV=2 - [CPT2_HUMAN] | 2 | 1 | 1 | 658 | 73.7 | 8.18 |
| Q8IV08 | Phospholipase D3 OS=Homo sapiens GN=PLD3 PE=1 SV=1 - [PLD3_HUMAN] | 2 | 2 | 2 | 490 | 54.7 | 6.47 |
| P50443 | Sulfate transporter OS=Homo sapiens GN=SLC26A2 PE=1 SV=2 - [S26A2_HUMAN] | 2 | 2 | 1 | 739 | 81.6 | 8.38 |
| P46063 | ATP-dependent DNA helicase Q1 OS=Homo sapiens GN=RECQL PE=1 SV=3 - [RECQ1_HUMAN] | 2 | 1 | 2 | 649 | 73.4 | 7.88 |
| Q9H0A0 | RNA cytidine acetyltransferase OS=Homo sapiens GN=NAT10 PE=1 SV=2 - [NAT10_HUMAN] | 2 | 1 | 3 | 1025 | 115.7 | 8.27 |
| Q70E73 | Ras-associated and pleckstrin homology domains-containing protein 1 OS=Homo sapiens GN=RAPH1 PE=1 SV=3 - [RAPH1_HUMAN] | 2 |  | 2 | 1250 | 135.2 | 8.85 |
| P06400 | Retinoblastoma-associated protein OS=Homo sapiens GN=RB1 PE=1 SV=2 - [RB_HUMAN] | 2 | 1 | 1 | 928 | 106.1 | 7.94 |
| Q9UBB6 | Neurochondrin OS=Homo sapiens GN=NCDN PE=1 SV=1 - [NCDN_HUMAN] | 2 | 1 | 2 | 729 | 78.8 | 5.48 |
| Q15291 | Retinoblastoma-binding protein 5 OS=Homo sapiens GN=RBBP5 PE=1 SV=2 - [RBBP5_HUMAN] | 2 | 2 | 2 | 538 | 59.1 | 5.10 |
| Q2TAY7 | WD40 repeat-containing protein SMU1 OS=Homo sapiens GN=SMU1 PE=1 SV=2 - [SMU1_HUMAN] | 2 | 2 | 1 | 513 | 57.5 | 7.18 |
| P53634 | Dipeptidyl peptidase 1 OS=Homo sapiens GN=CTSC PE=1 SV=2 - [CATC_HUMAN] | 2 | 1 | 2 | 463 | 51.8 | 6.99 |
| Q9P2W9 | Syntaxin-18 OS=Homo sapiens GN=STX18 PE=1 SV=1 - [STX18_HUMAN] | 2 | 2 | 1 | 335 | 38.6 | 5.49 |
| Q9NTI5 | Sister chromatid cohesion protein PDS5 homolog B OS=Homo sapiens GN=PDS5B PE=1 SV=1 - [PDS5B_HUMAN] | 2 | 2 | 3 | 1447 | 164.6 | 8.47 |
| A6NGU5 | Putative gamma-glutamyltranspeptidase 3 OS=Homo sapiens GN=GGT3P PE=5 SV=2 - [GGT3_HUMAN] | 2 | 2 | 2 | 568 | 61.5 | 7.14 |
| Q14203 | Dynactin subunit 1 OS=Homo sapiens GN=DCTN1 PE=1 SV=3 - [DCTN1_HUMAN] | 2 | 2 | 1 | 1278 | 141.6 | 5.81 |
| P06756 | Integrin alpha-V OS=Homo sapiens GN=ITGAV PE=1 SV=2 - [ITAV_HUMAN] | 2 | 2 | 1 | 1048 | 116.0 | 5.68 |
| Q93009 | Ubiquitin carboxyl-terminal hydrolase 7 OS=Homo sapiens GN=USP7 PE=1 SV=2 - [UBP7_HUMAN] | 2 |  | 2 | 1102 | 128.2 | 5.55 |
| Q96RE7 | Nucleus accumbens-associated protein 1 OS=Homo sapiens GN=NACC1 PE=1 SV=1 - [NACC1_HUMAN] | 2 |  | 2 | 527 | 57.2 | 5.74 |
| O76031 | ATP-dependent Clp protease ATP-binding subunit clpX-like. mitochondrial OS=Homo sapiens GN=CLPX PE=1 SV=2 - [CLPX_HUMAN] | 2 |  | 2 | 633 | 69.2 | 7.58 |
| Q8WUX9 | Charged multivesicular body protein 7 OS=Homo sapiens GN=CHMP7 PE=1 SV=1 - [CHMP7_HUMAN] | 2 | 3 | 2 | 453 | 50.9 | 5.35 |
| Q9HCG8 | Pre-mRNA-splicing factor CWC22 homolog OS=Homo sapiens GN=CWC22 PE=1 SV=3 - [CWC22_HUMAN] | 2 | 1 | 2 | 908 | 105.4 | 7.03 |
| Q14978 | Nucleolar and coiled-body phosphoprotein 1 OS=Homo sapiens GN=NOLC1 PE=1 SV=2 - [NOLC1_HUMAN] | 2 | 3 | 3 | 699 | 73.6 | 9.47 |
| Q8IY17 | Neuropathy target esterase OS=Homo sapiens OX=9606 GN=PNPLA6 PE=1 SV=3 - [PLPL6_HUMAN] | 2 | 1 | 2 | 1375 | 150.9 | 7.74 |
| Q9NQ66 | 1-phosphatidylinositol-4.5-bisphosphate phosphodiesterase beta-1 OS=Homo sapiens GN=PLCB1 PE=1 SV=1 - [PLCB1_HUMAN] | 2 | 2 | 1 | 1216 | 138.5 | 6.23 |
| Q9P253 | Vacuolar protein sorting-associated protein 18 homolog OS=Homo sapiens GN=VPS18 PE=1 SV=2 - [VPS18_HUMAN] | 2 |  | 2 | 973 | 110.1 | 6.07 |
| Q96SU4 | Oxysterol-binding protein-related protein 9 OS=Homo sapiens GN=OSBPL9 PE=1 SV=2 - [OSBL9_HUMAN] | 2 | 2 | 1 | 736 | 83.1 | 6.18 |
| Q7Z2W4 | Zinc finger CCCH-type antiviral protein 1 OS=Homo sapiens GN=ZC3HAV1 PE=1 SV=3 - [ZCCHV_HUMAN] | 2 | 1 | 2 | 902 | 101.4 | 8.40 |
| Q9NXF1 | Testis-expressed sequence 10 protein OS=Homo sapiens GN=TEX10 PE=1 SV=2 - [TEX10_HUMAN] | 2 | 2 | 2 | 929 | 105.6 | 9.36 |
| P11166 | Solute carrier family 2. facilitated glucose transporter member 1 OS=Homo sapiens GN=SLC2A1 PE=1 SV=2 - [GTR1_HUMAN] | 2 | 1 | 2 | 492 | 54.0 | 8.72 |
| Q9Y263 | Phospholipase A-2-activating protein OS=Homo sapiens GN=PLAA PE=1 SV=2 - [PLAP_HUMAN] | 2 | 1 | 2 | 795 | 87.1 | 6.37 |
| Q9P2I0 | Cleavage and polyadenylation specificity factor subunit 2 OS=Homo sapiens GN=CPSF2 PE=1 SV=2 - [CPSF2_HUMAN] | 2 | 2 | 2 | 782 | 88.4 | 5.11 |
| Q8IVH2 | Forkhead box protein P4 OS=Homo sapiens GN=FOXP4 PE=1 SV=1 - [FOXP4_HUMAN] | 2 | 1 | 1 | 680 | 73.4 | 6.40 |
| P52569 | Low affinity cationic amino acid transporter 2 OS=Homo sapiens GN=SLC7A2 PE=1 SV=2 - [CTR2_HUMAN] | 2 | 1 | 1 | 658 | 71.6 | 7.28 |
| Q9BVJ6 | U3 small nucleolar RNA-associated protein 14 homolog A OS=Homo sapiens GN=UTP14A PE=1 SV=1 - [UT14A_HUMAN] | 2 | 2 | 1 | 771 | 87.9 | 7.87 |
| Q12996 | Cleavage stimulation factor subunit 3 OS=Homo sapiens GN=CSTF3 PE=1 SV=1 - [CSTF3_HUMAN] | 2 | 2 | 2 | 717 | 82.9 | 8.12 |
| P51003 | Poly(A) polymerase alpha OS=Homo sapiens GN=PAPOLA PE=1 SV=4 - [PAPOA_HUMAN] | 2 | 1 | 1 | 745 | 82.8 | 7.37 |
| Q5JTZ9 | Probable alanyl-tRNA synthetase. mitochondrial OS=Homo sapiens GN=AARS2 PE=1 SV=1 - [SYAM_HUMAN] | 2 | 2 |  | 985 | 107.3 | 6.27 |
| P23378 | Glycine dehydrogenase [decarboxylating]. mitochondrial OS=Homo sapiens GN=GLDC PE=1 SV=2 - [GCSP_HUMAN] | 2 | 1 | 2 | 1020 | 112.7 | 7.11 |
| P51649 | Succinate-semialdehyde dehydrogenase. mitochondrial OS=Homo sapiens GN=ALDH5A1 PE=1 SV=2 - [SSDH_HUMAN] | 2 | 1 | 2 | 535 | 57.2 | 8.28 |
| Q9BTW9 | Tubulin-specific chaperone D OS=Homo sapiens GN=TBCD PE=1 SV=2 - [TBCD_HUMAN] | 2 | 2 | 1 | 1192 | 132.5 | 6.19 |
| Q9H307 | Pinin OS=Homo sapiens OX=9606 GN=PNN PE=1 SV=5 - [PININ_HUMAN] | 2 | 1 | 2 | 717 | 81.6 | 7.14 |
| Q9NXH9 | N(2).N(2)-dimethylguanosine tRNA methyltransferase OS=Homo sapiens GN=TRMT1 PE=1 SV=1 - [TRM1_HUMAN] | 2 |  | 2 | 659 | 72.2 | 7.64 |
| Q8TDD1 | ATP-dependent RNA helicase DDX54 OS=Homo sapiens GN=DDX54 PE=1 SV=2 - [DDX54_HUMAN] | 2 | 1 | 2 | 881 | 98.5 | 10.02 |
| Q96A65 | Exocyst complex component 4 OS=Homo sapiens GN=EXOC4 PE=1 SV=1 - [EXOC4_HUMAN] | 2 | 2 | 2 | 974 | 110.4 | 6.49 |
| Q15424 | Scaffold attachment factor B1 OS=Homo sapiens GN=SAFB PE=1 SV=4 - [SAFB1_HUMAN] | 2 | 2 | 2 | 915 | 102.6 | 5.47 |
| Q9NVE7 | Pantothenate kinase 4 OS=Homo sapiens GN=PANK4 PE=1 SV=1 - [PANK4_HUMAN] | 2 |  | 2 | 773 | 85.9 | 6.28 |
| Q8IYB3 | Serine/arginine repetitive matrix protein 1 OS=Homo sapiens GN=SRRM1 PE=1 SV=2 - [SRRM1_HUMAN] | 2 | 2 | 2 | 904 | 102.3 | 11.84 |
| Q9NPQ8 | Synembryn-A OS=Homo sapiens GN=RIC8A PE=1 SV=3 - [RIC8A_HUMAN] | 2 | 2 | 2 | 531 | 59.7 | 5.33 |
| Q9BWU0 | Kanadaptin OS=Homo sapiens GN=SLC4A1AP PE=1 SV=1 - [NADAP_HUMAN] | 2 |  | 2 | 796 | 88.8 | 5.19 |
| Q9NZB2 | Constitutive coactivator of PPAR-gamma-like protein 1 OS=Homo sapiens GN=FAM120A PE=1 SV=2 - [F120A_HUMAN] | 2 | 1 | 2 | 1118 | 121.8 | 8.88 |
| Q92805 | Golgin subfamily A member 1 OS=Homo sapiens GN=GOLGA1 PE=1 SV=3 - [GOGA1_HUMAN] | 2 | 1 | 1 | 767 | 88.1 | 5.27 |
| Q9BYT8 | Neurolysin. mitochondrial OS=Homo sapiens GN=NLN PE=1 SV=1 - [NEUL_HUMAN] | 2 | 2 | 1 | 704 | 80.6 | 6.64 |
| Q8IZ83 | Aldehyde dehydrogenase family 16 member A1 OS=Homo sapiens GN=ALDH16A1 PE=1 SV=2 - [A16A1_HUMAN] | 2 | 2 | 3 | 802 | 85.1 | 6.79 |
| O15042 | U2-associated protein SR140 OS=Homo sapiens GN=SR140 PE=1 SV=2 - [SR140_HUMAN] | 2 | 3 | 3 | 1029 | 118.2 | 8.47 |
| O94855 | Protein transport protein Sec24D OS=Homo sapiens GN=SEC24D PE=1 SV=2 - [SC24D_HUMAN] | 2 | 1 | 2 | 1032 | 112.9 | 7.25 |
| Q96R06 | Sperm-associated antigen 5 OS=Homo sapiens GN=SPAG5 PE=1 SV=2 - [SPAG5_HUMAN] | 2 | 1 | 2 | 1193 | 134.3 | 5.00 |
| Q5MIZ7 | Serine/threonine-protein phosphatase 4 regulatory subunit 3B OS=Homo sapiens GN=PPP4R3B PE=1 SV=2 - [P4R3B_HUMAN] | 2 |  | 2 | 849 | 97.4 | 4.96 |
| P06213 | Insulin receptor OS=Homo sapiens GN=INSR PE=1 SV=4 - [INSR_HUMAN] | 2 | 1 | 2 | 1382 | 156.2 | 6.20 |
| P13798 | Acylamino-acid-releasing enzyme OS=Homo sapiens GN=APEH PE=1 SV=4 - [ACPH_HUMAN] | 2 | 1 | 2 | 732 | 81.2 | 5.48 |
| Q96AQ6 | Pre-B-cell leukemia transcription factor-interacting protein 1 OS=Homo sapiens GN=PBXIP1 PE=1 SV=1 - [PBIP1_HUMAN] | 2 | 2 | 2 | 731 | 80.6 | 5.33 |
| Q9BZE4 | Nucleolar GTP-binding protein 1 OS=Homo sapiens GN=GTPBP4 PE=1 SV=3 - [NOG1_HUMAN] | 2 | 1 | 2 | 634 | 73.9 | 9.50 |
| P43246 | DNA mismatch repair protein Msh2 OS=Homo sapiens GN=MSH2 PE=1 SV=1 - [MSH2_HUMAN] | 2 | 1 | 2 | 934 | 104.7 | 5.77 |
| P07225 | Vitamin K-dependent protein S OS=Homo sapiens GN=PROS1 PE=1 SV=1 - [PROS_HUMAN] | 2 | 1 | 1 | 676 | 75.1 | 5.67 |
| Q5T160 | Probable arginyl-tRNA synthetase. mitochondrial OS=Homo sapiens GN=RARS2 PE=1 SV=1 - [SYRM_HUMAN] | 2 |  | 2 | 578 | 65.5 | 8.21 |
| P18858 | DNA ligase 1 OS=Homo sapiens GN=LIG1 PE=1 SV=1 - [DNLI1_HUMAN] | 2 | 3 | 3 | 919 | 101.7 | 5.62 |
| Q9BSJ2 | Gamma-tubulin complex component 2 OS=Homo sapiens GN=TUBGCP2 PE=1 SV=2 - [GCP2_HUMAN] | 2 | 2 | 2 | 902 | 102.5 | 6.84 |
| O75448 | Mediator of RNA polymerase II transcription subunit 24 OS=Homo sapiens GN=MED24 PE=1 SV=1 - [MED24_HUMAN] | 2 |  | 2 | 989 | 110.2 | 6.95 |
| Q92922 | SWI/SNF complex subunit SMARCC1 OS=Homo sapiens GN=SMARCC1 PE=1 SV=3 - [SMRC1_HUMAN] | 2 | 4 | 1 | 1105 | 122.8 | 5.76 |
| Q9UDT6 | CAP-Gly domain-containing linker protein 2 OS=Homo sapiens GN=CLIP2 PE=1 SV=1 - [CLIP2_HUMAN] | 2 | 1 | 2 | 1046 | 115.8 | 6.73 |
| Q9Y4F1 | FERM. RhoGEF and pleckstrin domain-containing protein 1 OS=Homo sapiens GN=FARP1 PE=1 SV=1 - [FARP1_HUMAN] | 2 |  | 2 | 1045 | 118.6 | 8.15 |
| Q9HCS7 | Pre-mRNA-splicing factor SYF1 OS=Homo sapiens GN=XAB2 PE=1 SV=2 - [SYF1_HUMAN] | 2 | 2 | 1 | 855 | 99.9 | 6.23 |
| O00754 | Lysosomal alpha-mannosidase OS=Homo sapiens GN=MAN2B1 PE=1 SV=3 - [MA2B1_HUMAN] | 2 | 2 | 2 | 1011 | 113.7 | 7.28 |
| Q86Y56 | Dynein assembly factor 5. axonemal OS=Homo sapiens GN=DNAAF5 PE=1 SV=4 - [DAAF5_HUMAN] | 2 |  | 2 | 855 | 93.5 | 6.42 |
| Q2M389 | WASH complex subunit SWIP OS=Homo sapiens GN=KIAA1033 PE=1 SV=2 - [WASH7_HUMAN] | 2 |  | 2 | 1173 | 136.3 | 7.44 |
| O95155 | Ubiquitin conjugation factor E4 B OS=Homo sapiens GN=UBE4B PE=1 SV=1 - [UBE4B_HUMAN] | 2 | 2 | 3 | 1302 | 146.1 | 6.55 |
| O75400 | Pre-mRNA-processing factor 40 homolog A OS=Homo sapiens GN=PRPF40A PE=1 SV=2 - [PR40A_HUMAN] | 2 | 2 | 2 | 957 | 108.7 | 7.56 |
| O60524 | Serologically defined colon cancer antigen 1 OS=Homo sapiens GN=SDCCAG1 PE=1 SV=4 - [SDCG1_HUMAN] | 2 | 1 | 1 | 1076 | 122.9 | 6.35 |
| Q5T8P6 | RNA-binding protein 26 OS=Homo sapiens GN=RBM26 PE=1 SV=3 - [RBM26_HUMAN] | 2 | 2 | 2 | 1007 | 113.5 | 9.16 |
| Q9UPT8 | Zinc finger CCCH domain-containing protein 4 OS=Homo sapiens GN=ZC3H4 PE=1 SV=3 - [ZC3H4_HUMAN] | 2 |  | 2 | 1303 | 140.2 | 6.27 |
| O75150 | E3 ubiquitin-protein ligase BRE1B OS=Homo sapiens OX=9606 GN=RNF40 PE=1 SV=5 - [BRE1B_HUMAN] | 2 | 2 | 2 | 1001 | 113.6 | 6.29 |
| Q9Y5K6 | CD2-associated protein OS=Homo sapiens GN=CD2AP PE=1 SV=1 - [CD2AP_HUMAN] | 2 | 2 | 2 | 639 | 71.4 | 6.40 |
| O60244 | Mediator of RNA polymerase II transcription subunit 14 OS=Homo sapiens GN=MED14 PE=1 SV=2 - [MED14_HUMAN] | 2 | 2 |  | 1454 | 160.5 | 8.73 |
| Q86TB9 | Protein PAT1 homolog 1 OS=Homo sapiens GN=PATL1 PE=1 SV=2 - [PATL1_HUMAN] | 2 | 1 | 2 | 770 | 86.8 | 6.67 |
| O43432 | Eukaryotic translation initiation factor 4 gamma 3 OS=Homo sapiens GN=EIF4G3 PE=1 SV=2 - [IF4G3_HUMAN] | 2 | 2 | 3 | 1585 | 176.5 | 5.38 |
| P57740 | Nuclear pore complex protein Nup107 OS=Homo sapiens GN=NUP107 PE=1 SV=1 - [NU107_HUMAN] | 2 | 2 | 2 | 925 | 106.3 | 5.43 |
| O14727 | Apoptotic protease-activating factor 1 OS=Homo sapiens GN=APAF1 PE=1 SV=2 - [APAF_HUMAN] | 2 | 2 | 2 | 1248 | 141.7 | 6.40 |
| O95197 | Reticulon-3 OS=Homo sapiens GN=RTN3 PE=1 SV=2 - [RTN3_HUMAN] | 2 | 1 | 1 | 1032 | 112.5 | 4.96 |
| Q86X55 | Histone-arginine methyltransferase CARM1 OS=Homo sapiens GN=CARM1 PE=1 SV=3 - [CARM1_HUMAN] | 2 | 1 | 1 | 608 | 65.8 | 6.73 |
| O60610 | Protein diaphanous homolog 1 OS=Homo sapiens GN=DIAPH1 PE=1 SV=2 - [DIAP1_HUMAN] | 2 | 2 | 2 | 1272 | 141.3 | 5.41 |
| O14617 | AP-3 complex subunit delta-1 OS=Homo sapiens GN=AP3D1 PE=1 SV=1 - [AP3D1_HUMAN] | 2 | 1 | 1 | 1153 | 130.1 | 8.48 |
| Q9Y6K5 | 2'-5'-oligoadenylate synthase 3 OS=Homo sapiens GN=OAS3 PE=1 SV=3 - [OAS3_HUMAN] | 2 | 1 | 1 | 1087 | 121.1 | 8.40 |
| Q8IX12 | Cell division cycle and apoptosis regulator protein 1 OS=Homo sapiens GN=CCAR1 PE=1 SV=2 - [CCAR1_HUMAN] | 2 | 1 | 2 | 1150 | 132.7 | 5.76 |
| Q92614 | Myosin-XVIIIa OS=Homo sapiens GN=MYO18A PE=1 SV=3 - [MY18A_HUMAN] | 2 | 1 | 1 | 2054 | 233.0 | 6.30 |
| P28340 | DNA polymerase delta catalytic subunit OS=Homo sapiens GN=POLD1 PE=1 SV=2 - [DPOD1_HUMAN] | 2 | 3 | 2 | 1107 | 123.6 | 7.03 |
| O95819 | Mitogen-activated protein kinase kinase kinase kinase 4 OS=Homo sapiens GN=MAP4K4 PE=1 SV=2 - [M4K4_HUMAN] | 2 | 2 | 1 | 1239 | 142.0 | 7.46 |
| Q5JRA6 | Melanoma inhibitory activity protein 3 OS=Homo sapiens GN=MIA3 PE=1 SV=1 - [MIA3_HUMAN] | 2 | 1 | 2 | 1907 | 213.6 | 4.84 |
| Q8N3C0 | Activating signal cointegrator 1 complex subunit 3 OS=Homo sapiens GN=ASCC3 PE=1 SV=3 - [HELC1_HUMAN] | 2 | 2 | 1 | 2202 | 251.3 | 7.09 |
| Q9BXW9 | Fanconi anemia group D2 protein OS=Homo sapiens GN=FANCD2 PE=1 SV=2 - [FACD2_HUMAN] | 2 |  | 2 | 1451 | 164.0 | 5.88 |
| Q13464 | Rho-associated protein kinase 1 OS=Homo sapiens GN=ROCK1 PE=1 SV=1 - [ROCK1_HUMAN] | 2 | 2 |  | 1354 | 158.1 | 5.90 |
| Q14690 | Protein RRP5 homolog OS=Homo sapiens GN=PDCD11 PE=1 SV=3 - [RRP5_HUMAN] | 2 |  | 2 | 1871 | 208.6 | 8.87 |
| P30876 | DNA-directed RNA polymerase II subunit RPB2 OS=Homo sapiens GN=POLR2B PE=1 SV=1 - [RPB2_HUMAN] | 2 | 2 |  | 1174 | 133.8 | 6.87 |
| O95347 | Structural maintenance of chromosomes protein 2 OS=Homo sapiens GN=SMC2 PE=1 SV=2 - [SMC2_HUMAN] | 2 | 1 | 2 | 1197 | 135.6 | 8.43 |
| O15031 | Plexin-B2 OS=Homo sapiens GN=PLXNB2 PE=1 SV=3 - [PLXB2_HUMAN] | 2 | 1 | 1 | 1838 | 205.0 | 6.24 |
| Q9UKV3 | Apoptotic chromatin condensation inducer in the nucleus OS=Homo sapiens GN=ACIN1 PE=1 SV=2 - [ACINU_HUMAN] | 2 | 2 | 2 | 1341 | 151.8 | 6.43 |
| P26358 | DNA (cytosine-5)-methyltransferase 1 OS=Homo sapiens GN=DNMT1 PE=1 SV=2 - [DNMT1_HUMAN] | 2 | 1 | 2 | 1616 | 183.0 | 7.75 |
| Q9P2M7 | Cingulin OS=Homo sapiens GN=CGN PE=1 SV=2 - [CING_HUMAN] | 2 | 1 | 1 | 1197 | 136.3 | 5.54 |
| Q9Y2H6 | Fibronectin type-III domain-containing protein 3A OS=Homo sapiens GN=FNDC3A PE=1 SV=4 - [FND3A_HUMAN] | 2 |  | 2 | 1198 | 131.8 | 6.71 |
| Q07157 | Tight junction protein ZO-1 OS=Homo sapiens GN=TJP1 PE=1 SV=3 - [ZO1_HUMAN] | 2 | 2 | 1 | 1748 | 195.3 | 6.70 |
| Q9NQT8 | Kinesin-like protein KIF13B OS=Homo sapiens GN=KIF13B PE=1 SV=2 - [KI13B_HUMAN] | 2 | 2 | 2 | 1826 | 202.7 | 5.88 |
| Q01433 | AMP deaminase 2 OS=Homo sapiens GN=AMPD2 PE=1 SV=2 - [AMPD2_HUMAN] | 2 | 1 | 1 | 879 | 100.6 | 6.93 |
| Q96K76 | Ubiquitin carboxyl-terminal hydrolase 47 OS=Homo sapiens GN=USP47 PE=1 SV=3 - [UBP47_HUMAN] | 2 | 1 | 1 | 1375 | 157.2 | 5.08 |
| P01023 | Alpha-2-macroglobulin OS=Homo sapiens GN=A2M PE=1 SV=3 - [A2MG_HUMAN] | 2 | 1 | 3 | 1474 | 163.2 | 6.46 |
| Q7KZ85 | Transcription elongation factor SPT6 OS=Homo sapiens GN=SUPT6H PE=1 SV=2 - [SPT6H_HUMAN] | 2 | 2 |  | 1726 | 198.9 | 4.91 |
| P35658 | Nuclear pore complex protein Nup214 OS=Homo sapiens GN=NUP214 PE=1 SV=2 - [NU214_HUMAN] | 2 |  | 2 | 2090 | 213.5 | 7.47 |
| P12270 | Nucleoprotein TPR OS=Homo sapiens GN=TPR PE=1 SV=3 - [TPR_HUMAN] | 2 | 2 | 1 | 2363 | 267.1 | 5.02 |
| Q9H4A3 | Serine/threonine-protein kinase WNK1 OS=Homo sapiens GN=WNK1 PE=1 SV=2 - [WNK1_HUMAN] | 2 |  | 2 | 2382 | 250.6 | 6.34 |
| P02751 | Fibronectin OS=Homo sapiens GN=FN1 PE=1 SV=4 - [FINC_HUMAN] | 2 | 1 | 2 | 2386 | 262.5 | 5.71 |
| A6NHR9 | Structural maintenance of chromosomes flexible hinge domain-containing protein 1 OS=Homo sapiens GN=SMCHD1 PE=1 SV=2 - [SMHD1_HUMAN] | 2 |  | 2 | 2005 | 226.2 | 7.30 |
| O75165 | DnaJ homolog subfamily C member 13 OS=Homo sapiens GN=DNAJC13 PE=1 SV=5 - [DJC13_HUMAN] | 2 | 2 | 1 | 2243 | 254.3 | 6.74 |

**Supplementary Table 4:** The full list of proteins commonly expressed in Huh7*^mock^* and Huh7*^hMT3^* cells. Out of total 1.774 common protein. 197 were up-regulated (FR ≥ 1.5) and 103 were downregulated (FR < 0.5).

| **Accession** | **Protein** | **Σ Unique Peptides** | **PSM HuH7**  ***^hMT3^*** | **PSM HuH7**  ***^mock^*** | **FR** |
| --- | --- | --- | --- | --- | --- |
| P35579 | Myosin-9 OS=Homo sapiens GN=MYH9 PE=1 SV=4 - [MYH9_HUMAN] | 5 | 7 | 1 | 7 |
| P35580 | Myosin-10 OS=Homo sapiens GN=MYH10 PE=1 SV=3 - [MYH10_HUMAN] | 11 | 14 | 3 | 4.6 |
| Q9BXJ9 | NMDA receptor-regulated protein 1 OS=Homo sapiens GN=NARG1 PE=1 SV=1 - [NARG1_HUMAN] | 4 | 4 | 1 | 4 |
| Q92922 | SWI/SNF complex subunit SMARCC1 OS=Homo sapiens GN=SMARCC1 PE=1 SV=3 - [SMRC1_HUMAN] | 2 | 4 | 1 | 4 |
| P61289 | Proteasome activator complex subunit 3 OS=Homo sapiens GN=PSME3 PE=1 SV=1 - [PSME3_HUMAN] | 5 | 6 | 2 | 3 |
| P19105 | Myosin regulatory light chain 12A OS=Homo sapiens GN=MYL12A PE=1 SV=2 - [ML12A_HUMAN] | 3 | 3 | 1 | 3 |
| Q92979 | Probable ribosome biogenesis protein NEP1 OS=Homo sapiens GN=EMG1 PE=1 SV=4 - [NEP1_HUMAN] | 3 | 3 | 1 | 3 |
| Q16762 | Thiosulfate sulfurtransferase OS=Homo sapiens GN=TST PE=1 SV=4 - [THTR_HUMAN] | 3 | 3 | 1 | 3 |
| Q9BRF8 | Serine/threonine-protein phosphatase CPPED1 OS=Homo sapiens GN=CPPED1 PE=1 SV=3 - [CPPED_HUMAN] | 3 | 3 | 1 | 3 |
| P19784 | Casein kinase II subunit alpha' OS=Homo sapiens GN=CSNK2A2 PE=1 SV=1 - [CSK22_HUMAN] | 3 | 3 | 1 | 3 |
| P53602 | Diphosphomevalonate decarboxylase OS=Homo sapiens GN=MVD PE=1 SV=1 - [MVD1_HUMAN] | 3 | 3 | 1 | 3 |
| P05121 | Plasminogen activator inhibitor 1 OS=Homo sapiens GN=SERPINE1 PE=1 SV=1 - [PAI1_HUMAN] | 3 | 3 | 1 | 3 |
| Q9HCM4 | Band 4.1-like protein 5 OS=Homo sapiens GN=EPB41L5 PE=1 SV=3 - [E41L5_HUMAN] | 3 | 3 | 1 | 3 |
| Q9H074 | Polyadenylate-binding protein-interacting protein 1 OS=Homo sapiens GN=PAIP1 PE=1 SV=1 - [PAIP1_HUMAN] | 3 | 3 | 1 | 3 |
| Q86XP3 | ATP-dependent RNA helicase DDX42 OS=Homo sapiens GN=DDX42 PE=1 SV=1 - [DDX42_HUMAN] | 3 | 3 | 1 | 3 |
| Q8N668 | COMM domain-containing protein 1 OS=Homo sapiens GN=COMMD1 PE=1 SV=1 - [COMD1_HUMAN] | 2 | 3 | 1 | 3 |
| O60493 | Sorting nexin-3 OS=Homo sapiens GN=SNX3 PE=1 SV=3 - [SNX3_HUMAN] | 2 | 3 | 1 | 3 |
| Q86YN1 | Dolichyldiphosphatase 1 OS=Homo sapiens GN=DOLPP1 PE=2 SV=1 - [DOPP1_HUMAN] | 2 | 3 | 1 | 3 |
| P27216 | Annexin A13 OS=Homo sapiens GN=ANXA13 PE=1 SV=3 - [ANX13_HUMAN] | 2 | 3 | 1 | 3 |
| Q96P11 | Putative methyltransferase NSUN5 OS=Homo sapiens GN=NSUN5 PE=1 SV=2 - [NSUN5_HUMAN] | 2 | 3 | 1 | 3 |
| P49589 | Cysteinyl-tRNA synthetase. cytoplasmic OS=Homo sapiens GN=CARS PE=1 SV=3 - [SYCC_HUMAN] | 2 | 3 | 1 | 3 |
| Q969V3 | Nicalin OS=Homo sapiens GN=NCLN PE=1 SV=2 - [NCLN_HUMAN] | 2 | 3 | 1 | 3 |
| P12429 | Annexin A3 OS=Homo sapiens GN=ANXA3 PE=1 SV=3 - [ANXA3_HUMAN] | 11 | 13 | 5 | 2.6 |
| P52789 | Hexokinase-2 OS=Homo sapiens GN=HK2 PE=1 SV=2 - [HXK2_HUMAN] | 5 | 5 | 2 | 2.5 |
| Q9Y2B0 | Protein canopy homolog 2 OS=Homo sapiens GN=CNPY2 PE=1 SV=1 - [CNPY2_HUMAN] | 4 | 5 | 2 | 2.5 |
| P15151 | Poliovirus receptor OS=Homo sapiens GN=PVR PE=1 SV=2 - [PVR_HUMAN] | 4 | 5 | 2 | 2.5 |
| P43034 | Platelet-activating factor acetylhydrolase IB subunit alpha OS=Homo sapiens GN=PAFAH1B1 PE=1 SV=2 - [LIS1_HUMAN] | 3 | 5 | 2 | 2.5 |
| P52294 | Importin subunit alpha-5 OS=Homo sapiens GN=KPNA1 PE=1 SV=3 - [IMA5_HUMAN] | 2 | 5 | 2 | 2.5 |
| Q13363 | C-terminal-binding protein 1 OS=Homo sapiens GN=CTBP1 PE=1 SV=2 - [CTBP1_HUMAN] | 5 | 4 | 2 | 2 |
| O00629 | Importin subunit alpha-4 OS=Homo sapiens GN=KPNA4 PE=1 SV=1 - [IMA4_HUMAN] | 4 | 8 | 4 | 2 |
| Q9P0L0 | Vesicle-associated membrane protein-associated protein A OS=Homo sapiens GN=VAPA PE=1 SV=3 - [VAPA_HUMAN] | 4 | 6 | 3 | 2 |
| Q9Y5X3 | Sorting nexin-5 OS=Homo sapiens GN=SNX5 PE=1 SV=1 - [SNX5_HUMAN] | 4 | 4 | 2 | 2 |
| Q92734 | Protein TFG OS=Homo sapiens GN=TFG PE=1 SV=2 - [TFG_HUMAN] | 4 | 4 | 2 | 2 |
| Q9Y2X3 | Nucleolar protein 58 OS=Homo sapiens GN=NOP58 PE=1 SV=1 - [NOP58_HUMAN] | 4 | 4 | 2 | 2 |
| O15427 | Monocarboxylate transporter 4 OS=Homo sapiens GN=SLC16A3 PE=1 SV=1 - [MOT4_HUMAN] | 4 | 4 | 2 | 2 |
| P15056 | Serine/threonine-protein kinase B-raf OS=Homo sapiens GN=BRAF PE=1 SV=4 - [BRAF_HUMAN] | 4 | 4 | 2 | 2 |
| Q92621 | Nuclear pore complex protein Nup205 OS=Homo sapiens GN=NUP205 PE=1 SV=3 - [NU205_HUMAN] | 4 | 4 | 2 | 2 |
| P05386 | 60S acidic ribosomal protein P1 OS=Homo sapiens GN=RPLP1 PE=1 SV=1 - [RLA1_HUMAN] | 3 | 6 | 3 | 2 |
| P61160 | Actin-related protein 2 OS=Homo sapiens GN=ACTR2 PE=1 SV=1 - [ARP2_HUMAN] | 3 | 4 | 2 | 2 |
| P18754 | Regulator of chromosome condensation OS=Homo sapiens GN=RCC1 PE=1 SV=1 - [RCC1_HUMAN] | 3 | 4 | 2 | 2 |
| Q9UI26 | Importin-11 OS=Homo sapiens GN=IPO11 PE=1 SV=1 - [IPO11_HUMAN] | 3 | 4 | 2 | 2 |
| O60264 | SWI/SNF-related matrix-associated actin-dependent regulator of chromatin subfamily A member 5 OS=Homo sapiens GN=SMARCA5 PE=1 SV=1 - [SMCA5_HUMAN] | 3 | 4 | 2 | 2 |
| Q01970 | 1-phosphatidylinositol-4.5-bisphosphate phosphodiesterase beta-3 OS=Homo sapiens GN=PLCB3 PE=1 SV=2 - [PLCB3_HUMAN] | 3 | 4 | 2 | 2 |
| Q15785 | Mitochondrial import receptor subunit TOM34 OS=Homo sapiens GN=TOMM34 PE=1 SV=2 - [TOM34_HUMAN] | 3 | 2 | 1 | 2 |
| Q9HCD5 | Nuclear receptor coactivator 5 OS=Homo sapiens GN=NCOA5 PE=1 SV=2 - [NCOA5_HUMAN] | 3 | 2 | 1 | 2 |
| P55011 | Solute carrier family 12 member 2 OS=Homo sapiens GN=SLC12A2 PE=1 SV=1 - [S12A2_HUMAN] | 3 | 2 | 1 | 2 |
| Q16706 | Alpha-mannosidase 2 OS=Homo sapiens GN=MAN2A1 PE=1 SV=2 - [MA2A1_HUMAN] | 3 | 2 | 1 | 2 |
| Q9UM54 | Myosin-VI OS=Homo sapiens GN=MYO6 PE=1 SV=4 - [MYO6_HUMAN] | 3 | 2 | 1 | 2 |
| P04080 | Cystatin-B OS=Homo sapiens GN=CSTB PE=1 SV=2 - [CYTB_HUMAN] | 2 | 2 | 1 | 2 |
| P62306 | Small nuclear ribonucleoprotein F OS=Homo sapiens GN=SNRPF PE=1 SV=1 - [RUXF_HUMAN] | 2 | 2 | 1 | 2 |
| O60888 | Protein CutA OS=Homo sapiens GN=CUTA PE=1 SV=2 - [CUTA_HUMAN] | 2 | 2 | 1 | 2 |
| O75348 | V-type proton ATPase subunit G 1 OS=Homo sapiens GN=ATP6V1G1 PE=1 SV=3 - [VATG1_HUMAN] | 2 | 2 | 1 | 2 |
| P84090 | Enhancer of rudimentary homolog OS=Homo sapiens GN=ERH PE=1 SV=1 - [ERH_HUMAN] | 2 | 2 | 1 | 2 |
| P26885 | Peptidyl-prolyl cis-trans isomerase FKBP2 OS=Homo sapiens GN=FKBP2 PE=1 SV=2 - [FKBP2_HUMAN] | 2 | 2 | 1 | 2 |
| O15116 | U6 snRNA-associated Sm-like protein LSm1 OS=Homo sapiens GN=LSM1 PE=1 SV=1 - [LSM1_HUMAN] | 2 | 2 | 1 | 2 |
| Q9BPX5 | Actin-related protein 2/3 complex subunit 5-like protein OS=Homo sapiens GN=ARPC5L PE=1 SV=1 - [ARP5L_HUMAN] | 2 | 2 | 1 | 2 |
| Q9UMY4 | Sorting nexin-12 OS=Homo sapiens GN=SNX12 PE=1 SV=3 - [SNX12_HUMAN] | 2 | 2 | 1 | 2 |
| P51452 | Dual specificity protein phosphatase 3 OS=Homo sapiens GN=DUSP3 PE=1 SV=1 - [DUS3_HUMAN] | 2 | 2 | 1 | 2 |
| Q9BRV3 | Sugar transporter SWEET1 OS=Homo sapiens OX=9606 GN=SLC50A1 PE=2 SV=1 - [SWET1_HUMAN] | 2 | 2 | 1 | 2 |
| P49006 | MARCKS-related protein OS=Homo sapiens GN=MARCKSL1 PE=1 SV=2 - [MRP_HUMAN] | 2 | 2 | 1 | 2 |
| O15145 | Actin-related protein 2/3 complex subunit 3 OS=Homo sapiens GN=ARPC3 PE=1 SV=3 - [ARPC3_HUMAN] | 2 | 2 | 1 | 2 |
| O00161 | Synaptosomal-associated protein 23 OS=Homo sapiens GN=SNAP23 PE=1 SV=1 - [SNP23_HUMAN] | 2 | 2 | 1 | 2 |
| P21291 | Cysteine and glycine-rich protein 1 OS=Homo sapiens GN=CSRP1 PE=1 SV=3 - [CSRP1_HUMAN] | 2 | 2 | 1 | 2 |
| Q9NRX1 | RNA-binding protein PNO1 OS=Homo sapiens GN=PNO1 PE=1 SV=1 - [PNO1_HUMAN] | 2 | 2 | 1 | 2 |
| Q9BSE5 | Agmatinase. mitochondrial OS=Homo sapiens GN=AGMAT PE=1 SV=2 - [SPEB_HUMAN] | 2 | 2 | 1 | 2 |
| P61964 | WD repeat-containing protein 5 OS=Homo sapiens GN=WDR5 PE=1 SV=1 - [WDR5_HUMAN] | 2 | 2 | 1 | 2 |
| Q15645 | Thyroid receptor-interacting protein 13 OS=Homo sapiens GN=TRIP13 PE=1 SV=2 - [TRP13_HUMAN] | 2 | 2 | 1 | 2 |
| Q86W42 | THO complex subunit 6 homolog OS=Homo sapiens GN=THOC6 PE=1 SV=1 - [THOC6_HUMAN] | 2 | 2 | 1 | 2 |
| Q92604 | Acyl-CoA:lysophosphatidylglycerol acyltransferase 1 OS=Homo sapiens GN=LPGAT1 PE=1 SV=1 - [LGAT1_HUMAN] | 2 | 2 | 1 | 2 |
| Q9UKX7 | Nuclear pore complex protein Nup50 OS=Homo sapiens GN=NUP50 PE=1 SV=2 - [NUP50_HUMAN] | 2 | 2 | 1 | 2 |
| Q9H3N1 | Thioredoxin-related transmembrane protein 1 OS=Homo sapiens GN=TMX1 PE=1 SV=1 - [TMX1_HUMAN] | 2 | 2 | 1 | 2 |
| Q13595 | Transformer-2 protein homolog alpha OS=Homo sapiens GN=TRA2A PE=1 SV=1 - [TRA2A_HUMAN] | 2 | 2 | 1 | 2 |
| O95674 | Phosphatidate cytidylyltransferase 2 OS=Homo sapiens GN=CDS2 PE=1 SV=1 - [CDS2_HUMAN] | 2 | 2 | 1 | 2 |
| O14618 | Copper chaperone for superoxide dismutase OS=Homo sapiens GN=CCS PE=1 SV=1 - [CCS_HUMAN] | 2 | 2 | 1 | 2 |
| Q53GS9 | U4/U6.U5 tri-snRNP-associated protein 2 OS=Homo sapiens GN=USP39 PE=1 SV=2 - [SNUT2_HUMAN] | 2 | 2 | 1 | 2 |
| Q96EP5 | DAZ-associated protein 1 OS=Homo sapiens GN=DAZAP1 PE=1 SV=1 - [DAZP1_HUMAN] | 2 | 2 | 1 | 2 |
| Q6UW02 | Cytochrome P450 20A1 OS=Homo sapiens GN=CYP20A1 PE=1 SV=1 - [CP20A_HUMAN] | 2 | 2 | 1 | 2 |
| P40121 | Macrophage-capping protein OS=Homo sapiens GN=CAPG PE=1 SV=2 - [CAPG_HUMAN] | 2 | 2 | 1 | 2 |
| P82930 | 28S ribosomal protein S34. mitochondrial OS=Homo sapiens GN=MRPS34 PE=1 SV=2 - [RT34_HUMAN] | 2 | 2 | 1 | 2 |
| P08240 | Signal recognition particle receptor subunit alpha OS=Homo sapiens GN=SRPR PE=1 SV=2 - [SRPR_HUMAN] | 2 | 2 | 1 | 2 |
| P30533 | Alpha-2-macroglobulin receptor-associated protein OS=Homo sapiens GN=LRPAP1 PE=1 SV=1 - [AMRP_HUMAN] | 2 | 2 | 1 | 2 |
| Q9BZ23 | Pantothenate kinase 2. mitochondrial OS=Homo sapiens GN=PANK2 PE=1 SV=3 - [PANK2_HUMAN] | 2 | 2 | 1 | 2 |
| Q8N1B4 | Vacuolar protein sorting-associated protein 52 homolog OS=Homo sapiens GN=VPS52 PE=1 SV=1 - [VPS52_HUMAN] | 2 | 2 | 1 | 2 |
| Q9C0B1 | Protein fto OS=Homo sapiens GN=FTO PE=1 SV=3 - [FTO_HUMAN] | 2 | 2 | 1 | 2 |
| P51654 | Glypican-3 OS=Homo sapiens GN=GPC3 PE=1 SV=1 - [GPC3_HUMAN] | 2 | 2 | 1 | 2 |
| Q3SXM5 | Inactive hydroxysteroid dehydrogenase-like protein 1 OS=Homo sapiens GN=HSDL1 PE=1 SV=3 - [HSDL1_HUMAN] | 2 | 2 | 1 | 2 |
| Q8IXI1 | Mitochondrial Rho GTPase 2 OS=Homo sapiens GN=RHOT2 PE=1 SV=2 - [MIRO2_HUMAN] | 2 | 2 | 1 | 2 |
| P11474 | Steroid hormone receptor ERR1 OS=Homo sapiens GN=ESRRA PE=1 SV=3 - [ERR1_HUMAN] | 2 | 2 | 1 | 2 |
| Q12765 | Secernin-1 OS=Homo sapiens GN=SCRN1 PE=1 SV=2 - [SCRN1_HUMAN] | 2 | 2 | 1 | 2 |
| Q7Z417 | Nuclear fragile X mental retardation-interacting protein 2 OS=Homo sapiens GN=NUFIP2 PE=1 SV=1 - [NUFP2_HUMAN] | 2 | 2 | 1 | 2 |
| Q6NUM9 | All-trans-retinol 13.14-reductase OS=Homo sapiens GN=RETSAT PE=1 SV=2 - [RETST_HUMAN] | 2 | 2 | 1 | 2 |
| P49643 | DNA primase large subunit OS=Homo sapiens GN=PRIM2 PE=1 SV=2 - [PRI2_HUMAN] | 2 | 2 | 1 | 2 |
| Q92905 | COP9 signalosome complex subunit 5 OS=Homo sapiens GN=COPS5 PE=1 SV=4 - [CSN5_HUMAN] | 2 | 2 | 1 | 2 |
| P36915 | Guanine nucleotide-binding protein-like 1 OS=Homo sapiens GN=GNL1 PE=1 SV=2 - [GNL1_HUMAN] | 2 | 2 | 1 | 2 |
| P32455 | Guanylate-binding protein 1 OS=Homo sapiens GN=GBP1 PE=1 SV=2 - [GBP1_HUMAN] | 2 | 2 | 1 | 2 |
| Q9NTJ3 | Structural maintenance of chromosomes protein 4 OS=Homo sapiens GN=SMC4 PE=1 SV=2 - [SMC4_HUMAN] | 2 | 2 | 1 | 2 |
| Q9BRZ2 | Tripartite motif-containing protein 56 OS=Homo sapiens GN=TRIM56 PE=1 SV=3 - [TRI56_HUMAN] | 2 | 2 | 1 | 2 |
| P50443 | Sulfate transporter OS=Homo sapiens GN=SLC26A2 PE=1 SV=2 - [S26A2_HUMAN] | 2 | 2 | 1 | 2 |
| Q2TAY7 | WD40 repeat-containing protein SMU1 OS=Homo sapiens GN=SMU1 PE=1 SV=2 - [SMU1_HUMAN] | 2 | 2 | 1 | 2 |
| Q9P2W9 | Syntaxin-18 OS=Homo sapiens GN=STX18 PE=1 SV=1 - [STX18_HUMAN] | 2 | 2 | 1 | 2 |
| Q14203 | Dynactin subunit 1 OS=Homo sapiens GN=DCTN1 PE=1 SV=3 - [DCTN1_HUMAN] | 2 | 2 | 1 | 2 |
| P06756 | Integrin alpha-V OS=Homo sapiens GN=ITGAV PE=1 SV=2 - [ITAV_HUMAN] | 2 | 2 | 1 | 2 |
| Q9NQ66 | 1-phosphatidylinositol-4.5-bisphosphate phosphodiesterase beta-1 OS=Homo sapiens GN=PLCB1 PE=1 SV=1 - [PLCB1_HUMAN] | 2 | 2 | 1 | 2 |
| Q96SU4 | Oxysterol-binding protein-related protein 9 OS=Homo sapiens GN=OSBPL9 PE=1 SV=2 - [OSBL9_HUMAN] | 2 | 2 | 1 | 2 |
| Q9BVJ6 | U3 small nucleolar RNA-associated protein 14 homolog A OS=Homo sapiens GN=UTP14A PE=1 SV=1 - [UT14A_HUMAN] | 2 | 2 | 1 | 2 |
| Q9BTW9 | Tubulin-specific chaperone D OS=Homo sapiens GN=TBCD PE=1 SV=2 - [TBCD_HUMAN] | 2 | 2 | 1 | 2 |
| Q9BYT8 | Neurolysin. mitochondrial OS=Homo sapiens GN=NLN PE=1 SV=1 - [NEUL_HUMAN] | 2 | 2 | 1 | 2 |
| Q9HCS7 | Pre-mRNA-splicing factor SYF1 OS=Homo sapiens GN=XAB2 PE=1 SV=2 - [SYF1_HUMAN] | 2 | 2 | 1 | 2 |
| O95819 | Mitogen-activated protein kinase kinase kinase kinase 4 OS=Homo sapiens GN=MAP4K4 PE=1 SV=2 - [M4K4_HUMAN] | 2 | 2 | 1 | 2 |
| Q8N3C0 | Activating signal cointegrator 1 complex subunit 3 OS=Homo sapiens GN=ASCC3 PE=1 SV=3 - [HELC1_HUMAN] | 2 | 2 | 1 | 2 |
| Q07157 | Tight junction protein ZO-1 OS=Homo sapiens GN=TJP1 PE=1 SV=3 - [ZO1_HUMAN] | 2 | 2 | 1 | 2 |
| P12270 | Nucleoprotein TPR OS=Homo sapiens GN=TPR PE=1 SV=3 - [TPR_HUMAN] | 2 | 2 | 1 | 2 |
| O75165 | DnaJ homolog subfamily C member 13 OS=Homo sapiens GN=DNAJC13 PE=1 SV=5 - [DJC13_HUMAN] | 2 | 2 | 1 | 2 |
| Q3LXA3 | Triokinase/FMN cyclase OS=Homo sapiens GN=TKFC PE=1 SV=2 - [TKFC_HUMAN] | 7 | 7 | 4 | 1.75 |
| Q9HDC9 | Adipocyte plasma membrane-associated protein OS=Homo sapiens GN=APMAP PE=1 SV=2 - [APMAP_HUMAN] | 6 | 7 | 4 | 1.75 |
| Q9Y3A5 | Ribosome maturation protein SBDS OS=Homo sapiens GN=SBDS PE=1 SV=4 - [SBDS_HUMAN] | 6 | 5 | 3 | 1.66 |
| O43592 | Exportin-T OS=Homo sapiens GN=XPOT PE=1 SV=2 - [XPOT_HUMAN] | 6 | 5 | 3 | 1.66 |
| P05387 | 60S acidic ribosomal protein P2 OS=Homo sapiens GN=RPLP2 PE=1 SV=1 - [RLA2_HUMAN] | 4 | 5 | 3 | 1.66 |
| Q14165 | Malectin OS=Homo sapiens GN=MLEC PE=1 SV=1 - [MLEC_HUMAN] | 4 | 5 | 3 | 1.66 |
| P04632 | Calpain small subunit 1 OS=Homo sapiens GN=CAPNS1 PE=1 SV=1 - [CPNS1_HUMAN] | 4 | 5 | 3 | 1.66 |
| Q9NZ01 | Trans-2.3-enoyl-CoA reductase OS=Homo sapiens GN=TECR PE=1 SV=1 - [TECR_HUMAN] | 4 | 5 | 3 | 1.66 |
| Q92542 | Nicastrin OS=Homo sapiens GN=NCSTN PE=1 SV=2 - [NICA_HUMAN] | 4 | 5 | 3 | 1.66 |
| P33121 | Long-chain-fatty-acid--CoA ligase 1 OS=Homo sapiens GN=ACSL1 PE=1 SV=1 - [ACSL1_HUMAN] | 4 | 5 | 3 | 1.66 |
| Q9BUQ8 | Probable ATP-dependent RNA helicase DDX23 OS=Homo sapiens GN=DDX23 PE=1 SV=3 - [DDX23_HUMAN] | 4 | 5 | 3 | 1.66 |
| Q9Y3E0 | Vesicle transport protein GOT1B OS=Homo sapiens GN=GOLT1B PE=1 SV=1 - [GOT1B_HUMAN] | 3 | 5 | 3 | 1.66 |
| O60762 | Dolichol-phosphate mannosyltransferase OS=Homo sapiens GN=DPM1 PE=1 SV=1 - [DPM1_HUMAN] | 3 | 5 | 3 | 1.66 |
| P08397 | Porphobilinogen deaminase OS=Homo sapiens GN=HMBS PE=1 SV=2 - [HEM3_HUMAN] | 3 | 5 | 3 | 1.66 |
| P27361 | Mitogen-activated protein kinase 3 OS=Homo sapiens GN=MAPK3 PE=1 SV=4 - [MK03_HUMAN] | 2 | 5 | 3 | 1.66 |
| P00558 | Phosphoglycerate kinase 1 OS=Homo sapiens GN=PGK1 PE=1 SV=3 - [PGK1_HUMAN] | 15 | 21 | 13 | 1.61 |
| O95865 | N(G).N(G)-dimethylarginine dimethylaminohydrolase 2 OS=Homo sapiens GN=DDAH2 PE=1 SV=1 - [DDAH2_HUMAN] | 8 | 8 | 5 | 1.6 |
| P22695 | Cytochrome b-c1 complex subunit 2. mitochondrial OS=Homo sapiens GN=UQCRC2 PE=1 SV=3 - [QCR2_HUMAN] | 8 | 8 | 5 | 1.6 |
| O00151 | PDZ and LIM domain protein 1 OS=Homo sapiens GN=PDLIM1 PE=1 SV=4 - [PDLI1_HUMAN] | 7 | 8 | 5 | 1.6 |
| P28482 | Mitogen-activated protein kinase 1 OS=Homo sapiens GN=MAPK1 PE=1 SV=3 - [MK01_HUMAN] | 4 | 11 | 7 | 1.57 |
| P27824 | Calnexin OS=Homo sapiens GN=CANX PE=1 SV=2 - [CALX_HUMAN] | 12 | 12 | 8 | 1.5 |
| P25789 | Proteasome subunit alpha type-4 OS=Homo sapiens GN=PSMA4 PE=1 SV=1 - [PSA4_HUMAN] | 8 | 9 | 6 | 1.5 |
| P61158 | Actin-related protein 3 OS=Homo sapiens GN=ACTR3 PE=1 SV=3 - [ARP3_HUMAN] | 8 | 9 | 6 | 1.5 |
| O75347 | Tubulin-specific chaperone A OS=Homo sapiens GN=TBCA PE=1 SV=3 - [TBCA_HUMAN] | 7 | 9 | 6 | 1.5 |
| Q13155 | Aminoacyl tRNA synthetase complex-interacting multifunctional protein 2 OS=Homo sapiens GN=AIMP2 PE=1 SV=2 - [AIMP2_HUMAN] | 5 | 6 | 4 | 1.5 |
| Q13148 | TAR DNA-binding protein 43 OS=Homo sapiens GN=TARDBP PE=1 SV=1 - [TADBP_HUMAN] | 5 | 6 | 4 | 1.5 |
| Q9H223 | EH domain-containing protein 4 OS=Homo sapiens GN=EHD4 PE=1 SV=1 - [EHD4_HUMAN] | 4 | 3 | 2 | 1.5 |
| P32189 | Glycerol kinase OS=Homo sapiens GN=GK PE=1 SV=3 - [GLPK_HUMAN] | 4 | 3 | 2 | 1.5 |
| O15294 | UDP-N-acetylglucosamine--peptide N-acetylglucosaminyltransferase 110 kDa subunit OS=Homo sapiens GN=OGT PE=1 SV=3 - [OGT1_HUMAN] | 4 | 3 | 2 | 1.5 |
| Q7L576 | Cytoplasmic FMR1-interacting protein 1 OS=Homo sapiens GN=CYFIP1 PE=1 SV=1 - [CYFP1_HUMAN] | 4 | 3 | 2 | 1.5 |
| P61163 | Alpha-centractin OS=Homo sapiens GN=ACTR1A PE=1 SV=1 - [ACTZ_HUMAN] | 3 | 6 | 4 | 1.5 |
| O00264 | Membrane-associated progesterone receptor component 1 OS=Homo sapiens GN=PGRMC1 PE=1 SV=3 - [PGRC1_HUMAN] | 3 | 3 | 2 | 1.5 |
| Q9UHV9 | Prefoldin subunit 2 OS=Homo sapiens GN=PFDN2 PE=1 SV=1 - [PFD2_HUMAN] | 3 | 3 | 2 | 1.5 |
| P84103 | Splicing factor. arginine/serine-rich 3 OS=Homo sapiens GN=SFRS3 PE=1 SV=1 - [SFRS3_HUMAN] | 3 | 3 | 2 | 1.5 |
| O43399 | Tumor protein D54 OS=Homo sapiens GN=TPD52L2 PE=1 SV=2 - [TPD54_HUMAN] | 3 | 3 | 2 | 1.5 |
| Q8WW12 | PEST proteolytic signal-containing nuclear protein OS=Homo sapiens GN=PCNP PE=1 SV=2 - [PCNP_HUMAN] | 3 | 3 | 2 | 1.5 |
| Q15102 | Platelet-activating factor acetylhydrolase IB subunit gamma OS=Homo sapiens GN=PAFAH1B3 PE=1 SV=1 - [PA1B3_HUMAN] | 3 | 3 | 2 | 1.5 |
| Q93062 | RNA-binding protein with multiple splicing OS=Homo sapiens GN=RBPMS PE=1 SV=1 - [RBPMS_HUMAN] | 3 | 3 | 2 | 1.5 |
| O14737 | Programmed cell death protein 5 OS=Homo sapiens GN=PDCD5 PE=1 SV=3 - [PDCD5_HUMAN] | 3 | 3 | 2 | 1.5 |
| Q9H0W9 | Ester hydrolase C11orf54 OS=Homo sapiens GN=C11orf54 PE=1 SV=1 - [CK054_HUMAN] | 3 | 3 | 2 | 1.5 |
| Q9H444 | Charged multivesicular body protein 4b OS=Homo sapiens GN=CHMP4B PE=1 SV=1 - [CHM4B_HUMAN] | 3 | 3 | 2 | 1.5 |
| P19623 | Spermidine synthase OS=Homo sapiens GN=SRM PE=1 SV=1 - [SPEE_HUMAN] | 3 | 3 | 2 | 1.5 |
| P62995 | Transformer-2 protein homolog beta OS=Homo sapiens GN=TRA2B PE=1 SV=1 - [TRA2B_HUMAN] | 3 | 3 | 2 | 1.5 |
| Q9NVD7 | Alpha-parvin OS=Homo sapiens GN=PARVA PE=1 SV=1 - [PARVA_HUMAN] | 3 | 3 | 2 | 1.5 |
| P40938 | Replication factor C subunit 3 OS=Homo sapiens GN=RFC3 PE=1 SV=2 - [RFC3_HUMAN] | 3 | 3 | 2 | 1.5 |
| Q08752 | Peptidyl-prolyl cis-trans isomerase D OS=Homo sapiens GN=PPID PE=1 SV=3 - [PPID_HUMAN] | 3 | 3 | 2 | 1.5 |
| Q3ZCQ8 | Mitochondrial import inner membrane translocase subunit TIM50 OS=Homo sapiens GN=TIMM50 PE=1 SV=2 - [TIM50_HUMAN] | 3 | 3 | 2 | 1.5 |
| P31937 | 3-hydroxyisobutyrate dehydrogenase. mitochondrial OS=Homo sapiens GN=HIBADH PE=1 SV=2 - [3HIDH_HUMAN] | 3 | 3 | 2 | 1.5 |
| Q16539 | Mitogen-activated protein kinase 14 OS=Homo sapiens GN=MAPK14 PE=1 SV=3 - [MK14_HUMAN] | 3 | 3 | 2 | 1.5 |
| Q16537 | Serine/threonine-protein phosphatase 2A 56 kDa regulatory subunit epsilon isoform OS=Homo sapiens GN=PPP2R5E PE=1 SV=1 - [2A5E_HUMAN] | 3 | 3 | 2 | 1.5 |
| Q8IUR7 | Armadillo repeat-containing protein 8 OS=Homo sapiens GN=ARMC8 PE=1 SV=2 - [ARMC8_HUMAN] | 3 | 3 | 2 | 1.5 |
| O43837 | Isocitrate dehydrogenase [NAD] subunit beta. mitochondrial OS=Homo sapiens GN=IDH3B PE=1 SV=2 - [IDH3B_HUMAN] | 3 | 3 | 2 | 1.5 |
| Q9Y512 | Sorting and assembly machinery component 50 homolog OS=Homo sapiens GN=SAMM50 PE=1 SV=3 - [SAM50_HUMAN] | 3 | 3 | 2 | 1.5 |
| P11177 | Pyruvate dehydrogenase E1 component subunit beta. mitochondrial OS=Homo sapiens GN=PDHB PE=1 SV=3 - [ODPB_HUMAN] | 3 | 3 | 2 | 1.5 |
| Q9UI12 | V-type proton ATPase subunit H OS=Homo sapiens GN=ATP6V1H PE=1 SV=1 - [VATH_HUMAN] | 3 | 3 | 2 | 1.5 |
| Q15642 | Cdc42-interacting protein 4 OS=Homo sapiens GN=TRIP10 PE=1 SV=3 - [CIP4_HUMAN] | 3 | 3 | 2 | 1.5 |
| Q9UKF6 | Cleavage and polyadenylation specificity factor subunit 3 OS=Homo sapiens GN=CPSF3 PE=1 SV=1 - [CPSF3_HUMAN] | 3 | 3 | 2 | 1.5 |
| Q96AC1 | Fermitin family homolog 2 OS=Homo sapiens GN=FERMT2 PE=1 SV=1 - [FERM2_HUMAN] | 3 | 3 | 2 | 1.5 |
| Q9UNS2 | COP9 signalosome complex subunit 3 OS=Homo sapiens GN=COPS3 PE=1 SV=3 - [CSN3_HUMAN] | 3 | 3 | 2 | 1.5 |
| P19823 | Inter-alpha-trypsin inhibitor heavy chain H2 OS=Homo sapiens GN=ITIH2 PE=1 SV=2 - [ITIH2_HUMAN] | 3 | 3 | 2 | 1.5 |
| Q8WYA6 | Beta-catenin-like protein 1 OS=Homo sapiens GN=CTNNBL1 PE=1 SV=1 - [CTBL1_HUMAN] | 3 | 3 | 2 | 1.5 |
| Q9UBD5 | Origin recognition complex subunit 3 OS=Homo sapiens GN=ORC3L PE=1 SV=1 - [ORC3_HUMAN] | 3 | 3 | 2 | 1.5 |
| Q9NQW7 | Xaa-Pro aminopeptidase 1 OS=Homo sapiens GN=XPNPEP1 PE=1 SV=3 - [XPP1_HUMAN] | 3 | 3 | 2 | 1.5 |
| O43929 | Origin recognition complex subunit 4 OS=Homo sapiens GN=ORC4L PE=1 SV=2 - [ORC4_HUMAN] | 3 | 3 | 2 | 1.5 |
| Q7L8L6 | FAST kinase domain-containing protein 5. mitochondrial OS=Homo sapiens GN=FASTKD5 PE=1 SV=1 - [FAKD5_HUMAN] | 3 | 3 | 2 | 1.5 |
| Q9Y450 | HBS1-like protein OS=Homo sapiens GN=HBS1L PE=1 SV=1 - [HBS1L_HUMAN] | 3 | 3 | 2 | 1.5 |
| Q9NSK0 | Kinesin light chain 4 OS=Homo sapiens GN=KLC4 PE=1 SV=3 - [KLC4_HUMAN] | 3 | 3 | 2 | 1.5 |
| P52888 | Thimet oligopeptidase OS=Homo sapiens GN=THOP1 PE=1 SV=2 - [THOP1_HUMAN] | 3 | 3 | 2 | 1.5 |
| Q86TG7 | Retrotransposon-derived protein PEG10 OS=Homo sapiens GN=PEG10 PE=1 SV=2 - [PEG10_HUMAN] | 3 | 3 | 2 | 1.5 |
| Q14527 | Helicase-like transcription factor OS=Homo sapiens GN=HLTF PE=1 SV=2 - [HLTF_HUMAN] | 3 | 3 | 2 | 1.5 |
| Q9Y2W1 | Thyroid hormone receptor-associated protein 3 OS=Homo sapiens GN=THRAP3 PE=1 SV=2 - [TR150_HUMAN] | 3 | 3 | 2 | 1.5 |
| Q13435 | Splicing factor 3B subunit 2 OS=Homo sapiens GN=SF3B2 PE=1 SV=2 - [SF3B2_HUMAN] | 3 | 3 | 2 | 1.5 |
| P52434 | DNA-directed RNA polymerases I. II. and III subunit RPABC3 OS=Homo sapiens GN=POLR2H PE=1 SV=4 - [RPAB3_HUMAN] | 2 | 3 | 2 | 1.5 |
| P51571 | Translocon-associated protein subunit delta OS=Homo sapiens GN=SSR4 PE=1 SV=1 - [SSRD_HUMAN] | 2 | 3 | 2 | 1.5 |
| O95372 | Acyl-protein thioesterase 2 OS=Homo sapiens GN=LYPLA2 PE=1 SV=1 - [LYPA2_HUMAN] | 2 | 3 | 2 | 1.5 |
| Q9UBX3 | Mitochondrial dicarboxylate carrier OS=Homo sapiens GN=SLC25A10 PE=1 SV=2 - [DIC_HUMAN] | 2 | 3 | 2 | 1.5 |
| Q92504 | Zinc transporter SLC39A7 OS=Homo sapiens GN=SLC39A7 PE=1 SV=2 - [S39A7_HUMAN] | 2 | 3 | 2 | 1.5 |
| Q9NVX2 | Notchless protein homolog 1 OS=Homo sapiens GN=NLE1 PE=1 SV=4 - [NLE1_HUMAN] | 2 | 3 | 2 | 1.5 |
| P43307 | Translocon-associated protein subunit alpha OS=Homo sapiens GN=SSR1 PE=1 SV=3 - [SSRA_HUMAN] | 2 | 3 | 2 | 1.5 |
| O43818 | U3 small nucleolar RNA-interacting protein 2 OS=Homo sapiens GN=RRP9 PE=1 SV=1 - [U3IP2_HUMAN] | 2 | 3 | 2 | 1.5 |
| Q71RC2 | La-related protein 4 OS=Homo sapiens GN=LARP4 PE=1 SV=3 - [LARP4_HUMAN] | 2 | 3 | 2 | 1.5 |
| Q03169 | Tumor necrosis factor alpha-induced protein 2 OS=Homo sapiens GN=TNFAIP2 PE=1 SV=2 - [TNAP2_HUMAN] | 2 | 3 | 2 | 1.5 |
| Q8WUX9 | Charged multivesicular body protein 7 OS=Homo sapiens GN=CHMP7 PE=1 SV=1 - [CHMP7_HUMAN] | 2 | 3 | 2 | 1.5 |
| P28340 | DNA polymerase delta catalytic subunit OS=Homo sapiens GN=POLD1 PE=1 SV=2 - [DPOD1_HUMAN] | 2 | 3 | 2 | 1.5 |
| Q02539 | Histone H1.1 OS=Homo sapiens GN=HIST1H1A PE=1 SV=3 - [H11_HUMAN] | 4 | 5 | 11 | 0.45 |
| P42765 | 3-ketoacyl-CoA thiolase. mitochondrial OS=Homo sapiens GN=ACAA2 PE=1 SV=2 - [THIM_HUMAN] | 9 | 4 | 9 | 0.44 |
| P54577 | Tyrosyl-tRNA synthetase. cytoplasmic OS=Homo sapiens GN=YARS PE=1 SV=4 - [SYYC_HUMAN] | 9 | 4 | 9 | 0.44 |
| Q9HCE1 | Putative helicase MOV-10 OS=Homo sapiens GN=MOV10 PE=1 SV=2 - [MOV10_HUMAN] | 9 | 4 | 9 | 0.44 |
| Q9NVJ2 | ADP-ribosylation factor-like protein 8B OS=Homo sapiens GN=ARL8B PE=1 SV=1 - [ARL8B_HUMAN] | 7 | 4 | 9 | 0.44 |
| P62195 | 26S protease regulatory subunit 8 OS=Homo sapiens GN=PSMC5 PE=1 SV=1 - [PRS8_HUMAN] | 7 | 4 | 9 | 0.44 |
| Q92598 | Heat shock protein 105 kDa OS=Homo sapiens GN=HSPH1 PE=1 SV=1 - [HS105_HUMAN] | 7 | 4 | 9 | 0.44 |
| Q9ULV4 | Coronin-1C OS=Homo sapiens GN=CORO1C PE=1 SV=1 - [COR1C_HUMAN] | 8 | 3 | 7 | 0.42 |
| P26639 | Threonyl-tRNA synthetase. cytoplasmic OS=Homo sapiens GN=TARS PE=1 SV=3 - [SYTC_HUMAN] | 8 | 3 | 7 | 0.42 |
| Q99536 | Synaptic vesicle membrane protein VAT-1 homolog OS=Homo sapiens GN=VAT1 PE=1 SV=2 - [VAT1_HUMAN] | 6 | 3 | 7 | 0.42 |
| P30040 | Endoplasmic reticulum resident protein 29 OS=Homo sapiens GN=ERP29 PE=1 SV=4 - [ERP29_HUMAN] | 6 | 3 | 7 | 0.42 |
| P39023 | 60S ribosomal protein L3 OS=Homo sapiens GN=RPL3 PE=1 SV=2 - [RL3_HUMAN] | 6 | 3 | 7 | 0.42 |
| Q9BS26 | Endoplasmic reticulum resident protein 44 OS=Homo sapiens GN=ERP44 PE=1 SV=1 - [ERP44_HUMAN] | 6 | 3 | 7 | 0.42 |
| Q16881 | Thioredoxin reductase 1. cytoplasmic OS=Homo sapiens GN=TXNRD1 PE=1 SV=3 - [TRXR1_HUMAN] | 6 | 3 | 7 | 0.42 |
| O14579 | Coatomer subunit epsilon OS=Homo sapiens GN=COPE PE=1 SV=3 - [COPE_HUMAN] | 5 | 3 | 7 | 0.42 |
| Q7L1Q6 | Basic leucine zipper and W2 domain-containing protein 1 OS=Homo sapiens GN=BZW1 PE=1 SV=1 - [BZW1_HUMAN] | 5 | 3 | 7 | 0.42 |
| O43795 | Myosin-Ib OS=Homo sapiens GN=MYO1B PE=1 SV=3 - [MYO1B_HUMAN] | 5 | 3 | 7 | 0.42 |
| Q9UDY2 | Tight junction protein ZO-2 OS=Homo sapiens GN=TJP2 PE=1 SV=2 - [ZO2_HUMAN] | 8 | 4 | 10 | 0.4 |
| P31153 | S-adenosylmethionine synthetase isoform type-2 OS=Homo sapiens GN=MAT2A PE=1 SV=1 - [METK2_HUMAN] | 5 | 2 | 5 | 0.4 |
| Q9H3P7 | Golgi resident protein GCP60 OS=Homo sapiens GN=ACBD3 PE=1 SV=4 - [GCP60_HUMAN] | 5 | 2 | 5 | 0.4 |
| Q9UNF1 | Melanoma-associated antigen D2 OS=Homo sapiens GN=MAGED2 PE=1 SV=2 - [MAGD2_HUMAN] | 5 | 2 | 5 | 0.4 |
| Q9P035 | Protein tyrosine phosphatase-like protein PTPLAD1 OS=Homo sapiens GN=PTPLAD1 PE=1 SV=2 - [PTAD1_HUMAN] | 4 | 2 | 5 | 0.4 |
| Q9NUJ1 | Mycophenolic acid acyl-glucuronide esterase. mitochondrial OS=Homo sapiens GN=ABHD10 PE=1 SV=1 - [ABHDA_HUMAN] | 4 | 2 | 5 | 0.4 |
| Q13283 | Ras GTPase-activating protein-binding protein 1 OS=Homo sapiens GN=G3BP1 PE=1 SV=1 - [G3BP1_HUMAN] | 4 | 2 | 5 | 0.4 |
| Q6XQN6 | Nicotinate phosphoribosyltransferase OS=Homo sapiens GN=NAPRT1 PE=1 SV=2 - [PNCB_HUMAN] | 4 | 2 | 5 | 0.4 |
| Q96CM8 | Acyl-CoA synthetase family member 2. mitochondrial OS=Homo sapiens GN=ACSF2 PE=1 SV=2 - [ACSF2_HUMAN] | 4 | 2 | 5 | 0.4 |
| P51116 | Fragile X mental retardation syndrome-related protein 2 OS=Homo sapiens GN=FXR2 PE=1 SV=2 - [FXR2_HUMAN] | 3 | 2 | 5 | 0.4 |
| P24941 | Cell division protein kinase 2 OS=Homo sapiens GN=CDK2 PE=1 SV=2 - [CDK2_HUMAN] | 6 | 3 | 8 | 0.37 |
| Q13017 | Rho GTPase-activating protein 5 OS=Homo sapiens GN=ARHGAP5 PE=1 SV=2 - [RHG05_HUMAN] | 6 | 3 | 8 | 0.37 |
| P50570 | Dynamin-2 OS=Homo sapiens GN=DNM2 PE=1 SV=2 - [DYN2_HUMAN] | 7 | 3 | 9 | 0.33 |
| P49821 | NADH dehydrogenase [ubiquinone] flavoprotein 1. mitochondrial OS=Homo sapiens GN=NDUFV1 PE=1 SV=4 - [NDUV1_HUMAN] | 6 | 2 | 6 | 0.33 |
| Q13586 | Stromal interaction molecule 1 OS=Homo sapiens GN=STIM1 PE=1 SV=3 - [STIM1_HUMAN] | 4 | 1 | 3 | 0.33 |
| Q9UHN6 | Transmembrane protein 2 OS=Homo sapiens GN=TMEM2 PE=1 SV=1 - [TMEM2_HUMAN] | 4 | 1 | 3 | 0.33 |
| P10599 | Thioredoxin OS=Homo sapiens GN=TXN PE=1 SV=3 - [THIO_HUMAN] | 3 | 1 | 3 | 0.33 |
| P41223 | Protein BUD31 homolog OS=Homo sapiens GN=BUD31 PE=1 SV=2 - [BUD31_HUMAN] | 3 | 1 | 3 | 0.33 |
| O43324 | Eukaryotic translation elongation factor 1 epsilon-1 OS=Homo sapiens GN=EEF1E1 PE=1 SV=1 - [MCA3_HUMAN] | 3 | 1 | 3 | 0.33 |
| Q9GZT3 | SRA stem-loop-interacting RNA-binding protein. mitochondrial OS=Homo sapiens GN=SLIRP PE=1 SV=1 - [SLIRP_HUMAN] | 3 | 1 | 3 | 0.33 |
| P46926 | Glucosamine-6-phosphate isomerase 1 OS=Homo sapiens GN=GNPDA1 PE=1 SV=1 - [GNPI1_HUMAN] | 3 | 1 | 3 | 0.33 |
| Q9BV57 | 1.2-dihydroxy-3-keto-5-methylthiopentene dioxygenase OS=Homo sapiens GN=ADI1 PE=1 SV=1 - [MTND_HUMAN] | 3 | 1 | 3 | 0.33 |
| Q8NCW5 | Apolipoprotein A-I-binding protein OS=Homo sapiens GN=APOA1BP PE=1 SV=2 - [AIBP_HUMAN] | 3 | 1 | 3 | 0.33 |
| O75352 | Mannose-P-dolichol utilization defect 1 protein OS=Homo sapiens GN=MPDU1 PE=1 SV=2 - [MPU1_HUMAN] | 3 | 1 | 3 | 0.33 |
| Q99447 | Ethanolamine-phosphate cytidylyltransferase OS=Homo sapiens GN=PCYT2 PE=1 SV=1 - [PCY2_HUMAN] | 3 | 1 | 3 | 0.33 |
| O14908 | PDZ domain-containing protein GIPC1 OS=Homo sapiens GN=GIPC1 PE=1 SV=2 - [GIPC1_HUMAN] | 3 | 1 | 3 | 0.33 |
| O60547 | GDP-mannose 4.6 dehydratase OS=Homo sapiens GN=GMDS PE=1 SV=1 - [GMDS_HUMAN] | 3 | 1 | 3 | 0.33 |
| P17612 | cAMP-dependent protein kinase catalytic subunit alpha OS=Homo sapiens GN=PRKACA PE=1 SV=2 - [KAPCA_HUMAN] | 3 | 1 | 3 | 0.33 |
| Q96S66 | Chloride channel CLIC-like protein 1 OS=Homo sapiens GN=CLCC1 PE=1 SV=1 - [CLCC1_HUMAN] | 3 | 1 | 3 | 0.33 |
| Q13510 | Acid ceramidase OS=Homo sapiens GN=ASAH1 PE=1 SV=5 - [ASAH1_HUMAN] | 3 | 1 | 3 | 0.33 |
| Q9UGK3 | Signal-transducing adaptor protein 2 OS=Homo sapiens GN=STAP2 PE=1 SV=2 - [STAP2_HUMAN] | 3 | 1 | 3 | 0.33 |
| Q9NUI1 | Peroxisomal 2.4-dienoyl-CoA reductase OS=Homo sapiens GN=DECR2 PE=1 SV=1 - [DECR2_HUMAN] | 3 | 1 | 3 | 0.33 |
| P49354 | Protein farnesyltransferase/geranylgeranyltransferase type-1 subunit alpha OS=Homo sapiens GN=FNTA PE=1 SV=1 - [FNTA_HUMAN] | 3 | 1 | 3 | 0.33 |
| P0C7P4 | Cytochrome b-c1 complex subunit Rieske-like protein 1 OS=Homo sapiens GN=UQCRFSL1 PE=2 SV=1 - [UCRIL_HUMAN] | 3 | 1 | 3 | 0.33 |
| Q96HS1 | Serine/threonine-protein phosphatase PGAM5. mitochondrial OS=Homo sapiens GN=PGAM5 PE=1 SV=2 - [PGAM5_HUMAN] | 3 | 1 | 3 | 0.33 |
| Q92552 | 28S ribosomal protein S27. mitochondrial OS=Homo sapiens GN=MRPS27 PE=1 SV=3 - [RT27_HUMAN] | 3 | 1 | 3 | 0.33 |
| Q5TDH0 | Protein DDI1 homolog 2 OS=Homo sapiens GN=DDI2 PE=1 SV=1 - [DDI2_HUMAN] | 3 | 1 | 3 | 0.33 |
| Q9HB07 | UPF0160 protein MYG1. mitochondrial OS=Homo sapiens GN=C12orf10 PE=1 SV=2 - [MYG1_HUMAN] | 3 | 1 | 3 | 0.33 |
| P08559 | Pyruvate dehydrogenase E1 component subunit alpha. somatic form. mitochondrial OS=Homo sapiens GN=PDHA1 PE=1 SV=3 - [ODPA_HUMAN] | 3 | 1 | 3 | 0.33 |
| P16278 | Beta-galactosidase OS=Homo sapiens GN=GLB1 PE=1 SV=2 - [BGAL_HUMAN] | 3 | 1 | 3 | 0.33 |
| P09960 | Leukotriene A-4 hydrolase OS=Homo sapiens GN=LTA4H PE=1 SV=2 - [LKHA4_HUMAN] | 3 | 1 | 3 | 0.33 |
| Q68E01 | Integrator complex subunit 3 OS=Homo sapiens GN=INTS3 PE=1 SV=1 - [INT3_HUMAN] | 3 | 1 | 3 | 0.33 |
| Q0VDF9 | Heat shock 70 kDa protein 14 OS=Homo sapiens GN=HSPA14 PE=1 SV=1 - [HSP7E_HUMAN] | 3 | 1 | 3 | 0.33 |
| Q9Y2A7 | Nck-associated protein 1 OS=Homo sapiens GN=NCKAP1 PE=1 SV=1 - [NCKP1_HUMAN] | 3 | 1 | 3 | 0.33 |
| P02671 | Fibrinogen alpha chain OS=Homo sapiens GN=FGA PE=1 SV=2 - [FIBA_HUMAN] | 3 | 1 | 3 | 0.33 |
| P25098 | Beta-adrenergic receptor kinase 1 OS=Homo sapiens GN=ADRBK1 PE=1 SV=2 - [ARBK1_HUMAN] | 3 | 1 | 3 | 0.33 |
| Q8NBJ5 | Procollagen galactosyltransferase 1 OS=Homo sapiens GN=GLT25D1 PE=1 SV=1 - [GT251_HUMAN] | 3 | 1 | 3 | 0.33 |
| Q6WKZ4 | Rab11 family-interacting protein 1 OS=Homo sapiens GN=RAB11FIP1 PE=1 SV=3 - [RFIP1_HUMAN] | 3 | 1 | 3 | 0.33 |
| P56134 | ATP synthase subunit f. mitochondrial OS=Homo sapiens GN=ATP5J2 PE=1 SV=3 - [ATPK_HUMAN] | 2 | 1 | 3 | 0.33 |
| P62847 | 40S ribosomal protein S24 OS=Homo sapiens GN=RPS24 PE=1 SV=1 - [RS24_HUMAN] | 2 | 1 | 3 | 0.33 |
| Q9Y3U8 | 60S ribosomal protein L36 OS=Homo sapiens GN=RPL36 PE=1 SV=3 - [RL36_HUMAN] | 2 | 1 | 3 | 0.33 |
| P28161 | Glutathione S-transferase Mu 2 OS=Homo sapiens GN=GSTM2 PE=1 SV=2 - [GSTM2_HUMAN] | 2 | 1 | 3 | 0.33 |
| P62633 | Cellular nucleic acid-binding protein OS=Homo sapiens GN=CNBP PE=1 SV=1 - [CNBP_HUMAN] | 2 | 1 | 3 | 0.33 |
| P49366 | Deoxyhypusine synthase OS=Homo sapiens GN=DHPS PE=1 SV=1 - [DHYS_HUMAN] | 2 | 1 | 3 | 0.33 |
| Q16222 | UDP-N-acetylhexosamine pyrophosphorylase OS=Homo sapiens GN=UAP1 PE=1 SV=3 - [UAP1_HUMAN] | 2 | 1 | 3 | 0.33 |
| Q7L5D6 | Golgi to ER traffic protein 4 homolog OS=Homo sapiens GN=GET4 PE=1 SV=1 - [GET4_HUMAN] | 2 | 1 | 3 | 0.33 |
| Q92609 | TBC1 domain family member 5 OS=Homo sapiens GN=TBC1D5 PE=1 SV=1 - [TBCD5_HUMAN] | 2 | 1 | 3 | 0.33 |
| Q9H0A0 | RNA cytidine acetyltransferase OS=Homo sapiens GN=NAT10 PE=1 SV=2 - [NAT10_HUMAN] | 2 | 1 | 3 | 0.33 |
| P01023 | Alpha-2-macroglobulin OS=Homo sapiens GN=A2M PE=1 SV=3 - [A2MG_HUMAN] | 2 | 1 | 3 | 0.33 |
| Q96AE4 | Far upstream element-binding protein 1 OS=Homo sapiens GN=FUBP1 PE=1 SV=3 - [FUBP1_HUMAN] | 7 | 3 | 10 | 0.3 |
| Q8N766 | Uncharacterized protein KIAA0090 OS=Homo sapiens GN=KIAA0090 PE=1 SV=1 - [K0090_HUMAN] | 7 | 2 | 7 | 0.28 |
| Q9BPX3 | Condensin complex subunit 3 OS=Homo sapiens GN=NCAPG PE=1 SV=1 - [CND3_HUMAN] | 5 | 1 | 4 | 0.25 |
| P49773 | Histidine triad nucleotide-binding protein 1 OS=Homo sapiens GN=HINT1 PE=1 SV=2 - [HINT1_HUMAN] | 4 | 1 | 4 | 0.25 |
| O60869 | Endothelial differentiation-related factor 1 OS=Homo sapiens GN=EDF1 PE=1 SV=1 - [EDF1_HUMAN] | 4 | 1 | 4 | 0.25 |
| Q16543 | Hsp90 co-chaperone Cdc37 OS=Homo sapiens GN=CDC37 PE=1 SV=1 - [CDC37_HUMAN] | 4 | 1 | 4 | 0.25 |
| Q9UHD1 | Cysteine and histidine-rich domain-containing protein 1 OS=Homo sapiens GN=CHORDC1 PE=1 SV=2 - [CHRD1_HUMAN] | 4 | 1 | 4 | 0.25 |
| O60256 | Phosphoribosyl pyrophosphate synthase-associated protein 2 OS=Homo sapiens GN=PRPSAP2 PE=1 SV=1 - [KPRB_HUMAN] | 4 | 1 | 4 | 0.25 |
| P02675 | Fibrinogen beta chain OS=Homo sapiens GN=FGB PE=1 SV=2 - [FIBB_HUMAN] | 4 | 1 | 4 | 0.25 |
| P37268 | Squalene synthase OS=Homo sapiens GN=FDFT1 PE=1 SV=1 - [FDFT_HUMAN] | 4 | 1 | 4 | 0.25 |
| P08237 | 6-phosphofructokinase. muscle type OS=Homo sapiens GN=PFKM PE=1 SV=2 - [K6PF_HUMAN] | 4 | 1 | 4 | 0.25 |
| P10515 | Dihydrolipoyllysine-residue acetyltransferase component of pyruvate dehydrogenase complex. mitochondrial OS=Homo sapiens GN=DLAT PE=1 SV=3 - [ODP2_HUMAN] | 4 | 1 | 4 | 0.25 |
| P02458 | Collagen alpha-1(II) chain OS=Homo sapiens GN=COL2A1 PE=1 SV=3 - [CO2A1_HUMAN] | 4 | 1 | 4 | 0.25 |
| O43681 | ATPase ASNA1 OS=Homo sapiens GN=ASNA1 PE=1 SV=2 - [ASNA_HUMAN] | 3 | 1 | 4 | 0.25 |
| Q6IBS0 | Twinfilin-2 OS=Homo sapiens GN=TWF2 PE=1 SV=2 - [TWF2_HUMAN] | 3 | 1 | 4 | 0.25 |
| P04181 | Ornithine aminotransferase. mitochondrial OS=Homo sapiens GN=OAT PE=1 SV=1 - [OAT_HUMAN] | 3 | 1 | 4 | 0.25 |
| Q9UBB4 | Ataxin-10 OS=Homo sapiens GN=ATXN10 PE=1 SV=1 - [ATX10_HUMAN] | 3 | 1 | 4 | 0.25 |
| Q96ST3 | Paired amphipathic helix protein Sin3a OS=Homo sapiens GN=SIN3A PE=1 SV=2 - [SIN3A_HUMAN] | 3 | 1 | 4 | 0.25 |
| Q9NS69 | Mitochondrial import receptor subunit TOM22 homolog OS=Homo sapiens GN=TOMM22 PE=1 SV=3 - [TOM22_HUMAN] | 2 | 1 | 4 | 0.25 |
| O95292 | Vesicle-associated membrane protein-associated protein B/C OS=Homo sapiens GN=VAPB PE=1 SV=3 - [VAPB_HUMAN] | 2 | 1 | 4 | 0.25 |
| P54802 | Alpha-N-acetylglucosaminidase OS=Homo sapiens GN=NAGLU PE=1 SV=2 - [ANAG_HUMAN] | 2 | 1 | 4 | 0.25 |
| O14773 | Tripeptidyl-peptidase 1 OS=Homo sapiens GN=TPP1 PE=1 SV=2 - [TPP1_HUMAN] | 4 | 1 | 4 | 0.25 |
| Q96T37 | Putative RNA-binding protein 15 OS=Homo sapiens GN=RBM15 PE=1 SV=2 - [RBM15_HUMAN] | 4 | 1 | 4 | 0.25 |
| Q8NE71 | ATP-binding cassette sub-family F member 1 OS=Homo sapiens GN=ABCF1 PE=1 SV=2 - [ABCF1_HUMAN] | 6 | 1 | 5 | 0.2 |
| O76021 | Ribosomal L1 domain-containing protein 1 OS=Homo sapiens GN=RSL1D1 PE=1 SV=3 - [RL1D1_HUMAN] | 5 | 1 | 5 | 0.2 |
| Q86UP2 | Kinectin OS=Homo sapiens GN=KTN1 PE=1 SV=1 - [KTN1_HUMAN] | 5 | 1 | 5 | 0.2 |
| P50440 | Glycine amidinotransferase. mitochondrial OS=Homo sapiens GN=GATM PE=1 SV=1 - [GATM_HUMAN] | 5 | 1 | 5 | 0.2 |

**Supplementary Table 5.** The full list of proteins exclusively expressed in Huh7*^hMT3^* cells.

| **Accession** | **Description** | **ΣUnique**  **Peptides** | **PSM**  **Huh7**  ***^hMT3^*** | **PSM**  **Huh7**  ***^mock^*** | **AA** | **MW [kDa]** | **pI** |
| --- | --- | --- | --- | --- | --- | --- | --- |
| O75569 | Interferon-inducible double stranded RNA-dependent protein kinase activator A OS=Homo sapiens GN=PRKRA PE=1 SV=1 - [PRKRA_HUMAN] | 3 | 4 | 0 | 313 | 34.4 | 8.41 |
| P49841 | Glycogen synthase kinase-3 beta OS=Homo sapiens GN=GSK3B PE=1 SV=2 - [GSK3B_HUMAN] | 3 | 4 | 0 | 420 | 46.7 | 8.78 |
| Q96QU8 | Exportin-6 OS=Homo sapiens GN=XPO6 PE=1 SV=1 - [XPO6_HUMAN] | 3 | 4 | 0 | 1125 | 128.8 | 6.35 |
| Q9BQE5 | Apolipoprotein L2 OS=Homo sapiens GN=APOL2 PE=1 SV=1 - [APOL2_HUMAN] | 3 | 3 | 0 | 337 | 37.1 | 6.74 |
| Q9HD26 | Golgi-associated PDZ and coiled-coil motif-containing protein OS=Homo sapiens GN=GOPC PE=1 SV=1 - [GOPC_HUMAN] | 3 | 3 | 0 | 462 | 50.5 | 5.92 |
| Q9C005 | Protein dpy-30 homolog OS=Homo sapiens GN=DPY30 PE=1 SV=1 - [DPY30_HUMAN] | 2 | 3 | 0 | 99 | 11.2 | 4.88 |
| Q16629 | Splicing factor. arginine/serine-rich 7 OS=Homo sapiens GN=SFRS7 PE=1 SV=1 - [SFRS7_HUMAN] | 2 | 3 | 0 | 238 | 27.4 | 11.82 |
| Q9H061 | Transmembrane protein 126A OS=Homo sapiens GN=TMEM126A PE=1 SV=1 - [T126A_HUMAN] | 2 | 2 | 0 | 195 | 21.5 | 9.26 |
| Q9UKY7 | Protein CDV3 homolog OS=Homo sapiens GN=CDV3 PE=1 SV=1 - [CDV3_HUMAN] | 2 | 2 | 0 | 258 | 27.3 | 6.4 |
| Q01995 | Transgelin OS=Homo sapiens GN=TAGLN PE=1 SV=4 - [TAGL_HUMAN] | 2 | 2 | 0 | 201 | 22.6 | 8.84 |
| Q13190 | Syntaxin-5 OS=Homo sapiens GN=STX5 PE=1 SV=2 - [STX5_HUMAN] | 2 | 2 | 0 | 355 | 39.6 | 9.16 |
| Q9UKZ1 | UPF0760 protein C2orf29 OS=Homo sapiens GN=C2orf29 PE=1 SV=1 - [CB029_HUMAN] | 2 | 2 | 0 | 510 | 55.2 | 6.4 |
| Q96C90 | Protein phosphatase 1 regulatory subunit 14B OS=Homo sapiens GN=PPP1R14B PE=1 SV=3 - [PP14B_HUMAN] | 2 | 2 | 0 | 147 | 15.9 | 4.86 |
| Q9NQZ2 | Something about silencing protein 10 OS=Homo sapiens GN=UTP3 PE=1 SV=1 - [SAS10_HUMAN] | 2 | 2 | 0 | 479 | 54.5 | 5.62 |
| Q99638 | Cell cycle checkpoint control protein RAD9A OS=Homo sapiens GN=RAD9A PE=1 SV=1 - [RAD9A_HUMAN] | 2 | 2 | 0 | 391 | 42.5 | 5.66 |
| Q5VIR6 | Vacuolar protein sorting-associated protein 53 homolog OS=Homo sapiens GN=VPS53 PE=1 SV=1 - [VPS53_HUMAN] | 2 | 2 | 0 | 699 | 79.6 | 6.02 |
| Q01650 | Large neutral amino acids transporter small subunit 1 OS=Homo sapiens GN=SLC7A5 PE=1 SV=2 - [LAT1_HUMAN] | 2 | 2 | 0 | 507 | 55 | 7.72 |
| O00273 | DNA fragmentation factor subunit alpha OS=Homo sapiens GN=DFFA PE=1 SV=1 - [DFFA_HUMAN] | 2 | 2 | 0 | 331 | 36.5 | 4.79 |
| Q9BUI4 | DNA-directed RNA polymerase III subunit RPC3 OS=Homo sapiens GN=POLR3C PE=1 SV=1 - [RPC3_HUMAN] | 2 | 2 | 0 | 534 | 60.6 | 7.31 |
| O43660 | Pleiotropic regulator 1 OS=Homo sapiens GN=PLRG1 PE=1 SV=1 - [PLRG1_HUMAN] | 2 | 2 | 0 | 514 | 57.2 | 9.17 |
| Q5JTZ9 | Probable alanyl-tRNA synthetase. mitochondrial OS=Homo sapiens GN=AARS2 PE=1 SV=1 - [SYAM_HUMAN] | 2 | 2 | 0 | 985 | 107.3 | 6.27 |
| O60244 | Mediator of RNA polymerase II transcription subunit 14 OS=Homo sapiens GN=MED14 PE=1 SV=2 - [MED14_HUMAN] | 2 | 2 | 0 | 1454 | 160.5 | 8.73 |
| Q13464 | Rho-associated protein kinase 1 OS=Homo sapiens GN=ROCK1 PE=1 SV=1 - [ROCK1_HUMAN] | 2 | 2 | 0 | 1354 | 158.1 | 5.9 |
| P30876 | DNA-directed RNA polymerase II subunit RPB2 OS=Homo sapiens GN=POLR2B PE=1 SV=1 - [RPB2_HUMAN] | 2 | 2 | 0 | 1174 | 133.8 | 6.87 |
| Q7KZ85 | Transcription elongation factor SPT6 OS=Homo sapiens GN=SUPT6H PE=1 SV=2 - [SPT6H_HUMAN] | 2 | 2 | 0 | 1726 | 198.9 | 4.91 |

**Supplementary Table 6.** The full list of proteins detected in comparative proteomic analysis of BCLC-3*^WT^* and Huh7*^hMT3^* cells.

| **Accession** | **Protein** | **ΣUnique Peptides** | **PSM**  **BCLC-3*^WT^*** | **PSM Huh7**  ***^hMT3^*** |
| --- | --- | --- | --- | --- |
| P06733 | Alpha-enolase OS=Homo sapiens GN=ENO1 PE=1 SV=2 - [ENOA_HUMAN] | 27 | 113 | 81 |
| P08670 | Vimentin OS=Homo sapiens GN=VIM PE=1 SV=4 - [VIME_HUMAN] | 36 | 81 | 39 |
| P21333 | Filamin-A OS=Homo sapiens GN=FLNA PE=1 SV=4 - [FLNA_HUMAN] | 60 | 76 | 30 |
| P07437 | Tubulin beta chain OS=Homo sapiens GN=TUBB PE=1 SV=2 - [TBB5_HUMAN] | 5 | 75 | 76 |
| P08238 | Heat shock protein HSP 90-beta OS=Homo sapiens GN=HSP90AB1 PE=1 SV=4 - [HS90B_HUMAN] | 23 | 66 | 65 |
| Q00610 | Clathrin heavy chain 1 OS=Homo sapiens GN=CLTC PE=1 SV=5 - [CLH1_HUMAN] | 52 | 60 | 69 |
| P13639 | Elongation factor 2 OS=Homo sapiens GN=EEF2 PE=1 SV=4 - [EF2_HUMAN] | 37 | 58 | 83 |
| P04406 | Glyceraldehyde-3-phosphate dehydrogenase OS=Homo sapiens GN=GAPDH PE=1 SV=3 - [G3P_HUMAN] | 17 | 56 | 63 |
| Q9BQE3 | Tubulin alpha-1C chain OS=Homo sapiens GN=TUBA1C PE=1 SV=1 - [TBA1C_HUMAN] | 2 | 55 | 74 |
| O43707 | Alpha-actinin-4 OS=Homo sapiens GN=ACTN4 PE=1 SV=2 - [ACTN4_HUMAN] | 27 | 54 | 63 |
| P14618 | Pyruvate kinase isozymes M1/M2 OS=Homo sapiens GN=PKM2 PE=1 SV=4 - [KPYM_HUMAN] | 30 | 52 | 58 |
| P07900 | Heat shock protein HSP 90-alpha OS=Homo sapiens GN=HSP90AA1 PE=1 SV=5 - [HS90A_HUMAN] | 21 | 51 | 77 |
| P02545 | Lamin-A/C OS=Homo sapiens GN=LMNA PE=1 SV=1 - [LMNA_HUMAN] | 34 | 51 | 28 |
| P10809 | 60 kDa heat shock protein. mitochondrial OS=Homo sapiens GN=HSPD1 PE=1 SV=2 - [CH60_HUMAN] | 34 | 50 | 79 |
| P68366 | Tubulin alpha-4A chain OS=Homo sapiens GN=TUBA4A PE=1 SV=1 - [TBA4A_HUMAN] | 5 | 49 | 56 |
| O75369 | Filamin-B OS=Homo sapiens GN=FLNB PE=1 SV=2 - [FLNB_HUMAN] | 42 | 49 | 33 |
| Q9Y490 | Talin-1 OS=Homo sapiens GN=TLN1 PE=1 SV=3 - [TLN1_HUMAN] | 50 | 48 | 53 |
| P12814 | Alpha-actinin-1 OS=Homo sapiens GN=ACTN1 PE=1 SV=2 - [ACTN1_HUMAN] | 18 | 47 | 35 |
| P68104 | Elongation factor 1-alpha 1 OS=Homo sapiens GN=EEF1A1 PE=1 SV=1 - [EF1A1_HUMAN] | 9 | 46 | 60 |
| P07355 | Annexin A2 OS=Homo sapiens GN=ANXA2 PE=1 SV=2 - [ANXA2_HUMAN] | 26 | 46 | 39 |
| Q13813 | Spectrin alpha chain. brain OS=Homo sapiens GN=SPTAN1 PE=1 SV=3 - [SPTA2_HUMAN] | 69 | 45 | 84 |
| Q13509 | Tubulin beta-3 chain OS=Homo sapiens GN=TUBB3 PE=1 SV=2 - [TBB3_HUMAN] | 3 | 43 | 39 |
| P09104 | Gamma-enolase OS=Homo sapiens GN=ENO2 PE=1 SV=3 - [ENOG_HUMAN] | 6 | 41 | 26 |
| P11142 | Heat shock cognate 71 kDa protein OS=Homo sapiens GN=HSPA8 PE=1 SV=1 - [HSP7C_HUMAN] | 28 | 40 | 52 |
| Q9BUF5 | Tubulin beta-6 chain OS=Homo sapiens GN=TUBB6 PE=1 SV=1 - [TBB6_HUMAN] | 8 | 40 | 27 |
| P11021 | 78 kDa glucose-regulated protein OS=Homo sapiens GN=HSPA5 PE=1 SV=2 - [GRP78_HUMAN] | 28 | 38 | 45 |
| P68032 | Actin. alpha cardiac muscle 1 OS=Homo sapiens GN=ACTC1 PE=1 SV=1 - [ACTC_HUMAN] | 2 | 38 | 31 |
| P55072 | Transitional endoplasmic reticulum ATPase OS=Homo sapiens GN=VCP PE=1 SV=4 - [TERA_HUMAN] | 29 | 37 | 53 |
| P29401 | Transketolase OS=Homo sapiens GN=TKT PE=1 SV=3 - [TKT_HUMAN] | 19 | 35 | 22 |
| P00352 | Retinal dehydrogenase 1 OS=Homo sapiens GN=ALDH1A1 PE=1 SV=2 - [AL1A1_HUMAN] | 24 | 34 | 50 |
| P14625 | Endoplasmin OS=Homo sapiens GN=HSP90B1 PE=1 SV=1 - [ENPL_HUMAN] | 30 | 34 | 42 |
| P55060 | Exportin-2 OS=Homo sapiens GN=CSE1L PE=1 SV=3 - [XPO2_HUMAN] | 31 | 33 | 37 |
| P22314 | Ubiquitin-like modifier-activating enzyme 1 OS=Homo sapiens GN=UBA1 PE=1 SV=3 - [UBA1_HUMAN] | 25 | 33 | 36 |
| Q14697 | Neutral alpha-glucosidase AB OS=Homo sapiens GN=GANAB PE=1 SV=3 - [GANAB_HUMAN] | 31 | 32 | 49 |
| P42704 | Leucine-rich PPR motif-containing protein. mitochondrial OS=Homo sapiens GN=LRPPRC PE=1 SV=3 - [LPPRC_HUMAN] | 36 | 32 | 29 |
| P04075 | Fructose-bisphosphate aldolase A OS=Homo sapiens GN=ALDOA PE=1 SV=2 - [ALDOA_HUMAN] | 22 | 32 | 28 |
| P00338 | L-lactate dehydrogenase A chain OS=Homo sapiens GN=LDHA PE=1 SV=2 - [LDHA_HUMAN] | 24 | 31 | 52 |
| P50395 | Rab GDP dissociation inhibitor beta OS=Homo sapiens GN=GDI2 PE=1 SV=2 - [GDIB_HUMAN] | 15 | 31 | 28 |
| P18206 | Vinculin OS=Homo sapiens GN=VCL PE=1 SV=4 - [VINC_HUMAN] | 28 | 31 | 24 |
| P08107 | Heat shock 70 kDa protein 1A/1B OS=Homo sapiens GN=HSPA1A PE=1 SV=5 - [HSP71_HUMAN] | 19 | 31 | 18 |
| P04083 | Annexin A1 OS=Homo sapiens GN=ANXA1 PE=1 SV=2 - [ANXA1_HUMAN] | 19 | 31 | 4 |
| P60174 | Triosephosphate isomerase OS=Homo sapiens GN=TPI1 PE=1 SV=3 - [TPIS_HUMAN] | 17 | 30 | 34 |
| Q01082 | Spectrin beta chain. brain 1 OS=Homo sapiens GN=SPTBN1 PE=1 SV=2 - [SPTB2_HUMAN] | 50 | 29 | 58 |
| P37802 | Transgelin-2 OS=Homo sapiens GN=TAGLN2 PE=1 SV=3 - [TAGL2_HUMAN] | 13 | 29 | 13 |
| P07237 | Protein disulfide-isomerase OS=Homo sapiens GN=P4HB PE=1 SV=3 - [PDIA1_HUMAN] | 26 | 28 | 47 |
| Q14974 | Importin subunit beta-1 OS=Homo sapiens GN=KPNB1 PE=1 SV=2 - [IMB1_HUMAN] | 25 | 28 | 33 |
| P46940 | Ras GTPase-activating-like protein IQGAP1 OS=Homo sapiens GN=IQGAP1 PE=1 SV=1 - [IQGA1_HUMAN] | 28 | 28 | 24 |
| Q01518 | Adenylyl cyclase-associated protein 1 OS=Homo sapiens GN=CAP1 PE=1 SV=5 - [CAP1_HUMAN] | 18 | 27 | 33 |
| Q14315 | Filamin-C OS=Homo sapiens GN=FLNC PE=1 SV=3 - [FLNC_HUMAN] | 15 | 27 | 7 |
| P49327 | Fatty acid synthase OS=Homo sapiens GN=FASN PE=1 SV=3 - [FAS_HUMAN] | 49 | 26 | 64 |
| P38646 | Stress-70 protein. mitochondrial OS=Homo sapiens GN=HSPA9 PE=1 SV=2 - [GRP75_HUMAN] | 20 | 26 | 27 |
| P63104 | 14-3-3 protein zeta/delta OS=Homo sapiens GN=YWHAZ PE=1 SV=1 - [1433Z_HUMAN] | 13 | 25 | 21 |
| P15311 | Ezrin OS=Homo sapiens GN=EZR PE=1 SV=4 - [EZRI_HUMAN] | 14 | 25 | 17 |
| P26038 | Moesin OS=Homo sapiens GN=MSN PE=1 SV=3 - [MOES_HUMAN] | 14 | 25 | 3 |
| P06576 | ATP synthase subunit beta. mitochondrial OS=Homo sapiens GN=ATP5B PE=1 SV=3 - [ATPB_HUMAN] | 20 | 24 | 38 |
| O60701 | UDP-glucose 6-dehydrogenase OS=Homo sapiens GN=UGDH PE=1 SV=1 - [UGDH_HUMAN] | 25 | 24 | 35 |
| P26641 | Elongation factor 1-gamma OS=Homo sapiens GN=EEF1G PE=1 SV=3 - [EF1G_HUMAN] | 17 | 24 | 30 |
| Q08211 | ATP-dependent RNA helicase A OS=Homo sapiens GN=DHX9 PE=1 SV=4 - [DHX9_HUMAN] | 26 | 24 | 29 |
| P08758 | Annexin A5 OS=Homo sapiens GN=ANXA5 PE=1 SV=2 - [ANXA5_HUMAN] | 19 | 24 | 28 |
| P12277 | Creatine kinase B-type OS=Homo sapiens GN=CKB PE=1 SV=1 - [KCRB_HUMAN] | 19 | 23 | 90 |
| P30101 | Protein disulfide-isomerase A3 OS=Homo sapiens GN=PDIA3 PE=1 SV=4 - [PDIA3_HUMAN] | 22 | 23 | 33 |
| P19338 | Nucleolin OS=Homo sapiens GN=NCL PE=1 SV=3 - [NUCL_HUMAN] | 21 | 23 | 29 |
| P78371 | T-complex protein 1 subunit beta OS=Homo sapiens GN=CCT2 PE=1 SV=4 - [TCPB_HUMAN] | 20 | 23 | 22 |
| P62258 | 14-3-3 protein epsilon OS=Homo sapiens GN=YWHAE PE=1 SV=1 - [1433E_HUMAN] | 14 | 22 | 28 |
| P40227 | T-complex protein 1 subunit zeta OS=Homo sapiens GN=CCT6A PE=1 SV=3 - [TCPZ_HUMAN] | 15 | 22 | 27 |
| P23528 | Cofilin-1 OS=Homo sapiens GN=CFL1 PE=1 SV=3 - [COF1_HUMAN] | 8 | 22 | 24 |
| P62937 | Peptidyl-prolyl cis-trans isomerase A OS=Homo sapiens GN=PPIA PE=1 SV=2 - [PPIA_HUMAN] | 10 | 22 | 17 |
| Q14764 | Major vault protein OS=Homo sapiens GN=MVP PE=1 SV=4 - [MVP_HUMAN] | 21 | 22 | 0 |
| P61978 | Heterogeneous nuclear ribonucleoprotein K OS=Homo sapiens GN=HNRNPK PE=1 SV=1 - [HNRPK_HUMAN] | 16 | 21 | 38 |
| P27797 | Calreticulin OS=Homo sapiens GN=CALR PE=1 SV=1 - [CALR_HUMAN] | 14 | 21 | 22 |
| Q06830 | Peroxiredoxin-1 OS=Homo sapiens GN=PRDX1 PE=1 SV=1 - [PRDX1_HUMAN] | 11 | 21 | 16 |
| P34932 | Heat shock 70 kDa protein 4 OS=Homo sapiens GN=HSPA4 PE=1 SV=4 - [HSP74_HUMAN] | 16 | 21 | 9 |
| P60842 | Eukaryotic initiation factor 4A-I OS=Homo sapiens GN=EIF4A1 PE=1 SV=1 - [IF4A1_HUMAN] | 19 | 20 | 53 |
| P22626 | Heterogeneous nuclear ribonucleoproteins A2/B1 OS=Homo sapiens GN=HNRNPA2B1 PE=1 SV=2 - [ROA2_HUMAN] | 15 | 20 | 29 |
| Q7KZF4 | Staphylococcal nuclease domain-containing protein 1 OS=Homo sapiens GN=SND1 PE=1 SV=1 - [SND1_HUMAN] | 26 | 19 | 30 |
| P31939 | Bifunctional purine biosynthesis protein PURH OS=Homo sapiens GN=ATIC PE=1 SV=3 - [PUR9_HUMAN] | 23 | 19 | 21 |
| P00558 | Phosphoglycerate kinase 1 OS=Homo sapiens GN=PGK1 PE=1 SV=3 - [PGK1_HUMAN] | 15 | 19 | 21 |
| Q9BSJ8 | Extended synaptotagmin-1 OS=Homo sapiens GN=ESYT1 PE=1 SV=1 - [ESYT1_HUMAN] | 20 | 19 | 19 |
| P50454 | Serpin H1 OS=Homo sapiens GN=SERPINH1 PE=1 SV=2 - [SERPH_HUMAN] | 14 | 19 | 16 |
| P63244 | Guanine nucleotide-binding protein subunit beta-2-like 1 OS=Homo sapiens GN=GNB2L1 PE=1 SV=3 - [GBLP_HUMAN] | 16 | 19 | 14 |
| Q01813 | 6-phosphofructokinase type C OS=Homo sapiens GN=PFKP PE=1 SV=2 - [K6PP_HUMAN] | 14 | 19 | 0 |
| P13010 | ATP-dependent DNA helicase 2 subunit 2 OS=Homo sapiens GN=XRCC5 PE=1 SV=3 - [KU86_HUMAN] | 23 | 18 | 29 |
| P25705 | ATP synthase subunit alpha. mitochondrial OS=Homo sapiens GN=ATP5A1 PE=1 SV=1 - [ATPA_HUMAN] | 21 | 18 | 29 |
| P17987 | T-complex protein 1 subunit alpha OS=Homo sapiens GN=TCP1 PE=1 SV=1 - [TCPA_HUMAN] | 21 | 18 | 24 |
| Q99832 | T-complex protein 1 subunit eta OS=Homo sapiens GN=CCT7 PE=1 SV=2 - [TCPH_HUMAN] | 19 | 18 | 23 |
| P35221 | Catenin alpha-1 OS=Homo sapiens GN=CTNNA1 PE=1 SV=1 - [CTNA1_HUMAN] | 22 | 18 | 22 |
| P53621 | Coatomer subunit alpha OS=Homo sapiens GN=COPA PE=1 SV=2 - [COPA_HUMAN] | 22 | 18 | 22 |
| P04844 | Dolichyl-diphosphooligosaccharide--protein glycosyltransferase subunit 2 OS=Homo sapiens GN=RPN2 PE=1 SV=3 - [RPN2_HUMAN] | 18 | 18 | 21 |
| P27348 | 14-3-3 protein theta OS=Homo sapiens GN=YWHAQ PE=1 SV=1 - [1433T_HUMAN] | 9 | 18 | 14 |
| P42330 | Aldo-keto reductase family 1 member C3 OS=Homo sapiens GN=AKR1C3 PE=1 SV=4 - [AK1C3_HUMAN] | 7 | 18 | 5 |
| P26599 | Polypyrimidine tract-binding protein 1 OS=Homo sapiens GN=PTBP1 PE=1 SV=1 - [PTBP1_HUMAN] | 12 | 17 | 26 |
| O14980 | Exportin-1 OS=Homo sapiens GN=XPO1 PE=1 SV=1 - [XPO1_HUMAN] | 21 | 17 | 19 |
| Q02878 | 60S ribosomal protein L6 OS=Homo sapiens GN=RPL6 PE=1 SV=3 - [RL6_HUMAN] | 13 | 17 | 18 |
| P53396 | ATP-citrate synthase OS=Homo sapiens GN=ACLY PE=1 SV=3 - [ACLY_HUMAN] | 15 | 17 | 17 |
| P62424 | 60S ribosomal protein L7a OS=Homo sapiens GN=RPL7A PE=1 SV=2 - [RL7A_HUMAN] | 15 | 17 | 16 |
| P50990 | T-complex protein 1 subunit theta OS=Homo sapiens GN=CCT8 PE=1 SV=4 - [TCPQ_HUMAN] | 18 | 17 | 15 |
| O00299 | Chloride intracellular channel protein 1 OS=Homo sapiens GN=CLIC1 PE=1 SV=4 - [CLIC1_HUMAN] | 13 | 17 | 12 |
| Q13200 | 26S proteasome non-ATPase regulatory subunit 2 OS=Homo sapiens GN=PSMD2 PE=1 SV=3 - [PSMD2_HUMAN] | 14 | 17 | 11 |
| Q86VP6 | Cullin-associated NEDD8-dissociated protein 1 OS=Homo sapiens GN=CAND1 PE=1 SV=2 - [CAND1_HUMAN] | 27 | 16 | 38 |
| P0CG48 | Polyubiquitin-C OS=Homo sapiens GN=UBC PE=1 SV=3 - [UBC_HUMAN] | 5 | 16 | 28 |
| Q00839 | Heterogeneous nuclear ribonucleoprotein U OS=Homo sapiens GN=HNRNPU PE=1 SV=6 - [HNRPU_HUMAN] | 19 | 16 | 22 |
| P30153 | Serine/threonine-protein phosphatase 2A 65 kDa regulatory subunit A alpha isoform OS=Homo sapiens GN=PPP2R1A PE=1 SV=4 - [2AAA_HUMAN] | 11 | 16 | 22 |
| P12956 | ATP-dependent DNA helicase 2 subunit 1 OS=Homo sapiens GN=XRCC6 PE=1 SV=2 - [KU70_HUMAN] | 21 | 16 | 18 |
| Q9Y4L1 | Hypoxia up-regulated protein 1 OS=Homo sapiens GN=HYOU1 PE=1 SV=1 - [HYOU1_HUMAN] | 17 | 16 | 18 |
| P21796 | Voltage-dependent anion-selective channel protein 1 OS=Homo sapiens GN=VDAC1 PE=1 SV=2 - [VDAC1_HUMAN] | 12 | 16 | 17 |
| P07737 | Profilin-1 OS=Homo sapiens GN=PFN1 PE=1 SV=2 - [PROF1_HUMAN] | 10 | 16 | 16 |
| P63010 | AP-2 complex subunit beta OS=Homo sapiens GN=AP2B1 PE=1 SV=1 - [AP2B1_HUMAN] | 10 | 16 | 14 |
| P54136 | Arginyl-tRNA synthetase. cytoplasmic OS=Homo sapiens GN=RARS PE=1 SV=2 - [SYRC_HUMAN] | 15 | 16 | 13 |
| P11413 | Glucose-6-phosphate 1-dehydrogenase OS=Homo sapiens GN=G6PD PE=1 SV=4 - [G6PD_HUMAN] | 16 | 16 | 7 |
| O00159 | Unconventional myosin-Ic OS=Homo sapiens GN=MYO1C PE=1 SV=4 - [MYO1C_HUMAN] | 15 | 16 | 2 |
| P09651 | Heterogeneous nuclear ribonucleoprotein A1 OS=Homo sapiens GN=HNRNPA1 PE=1 SV=5 - [ROA1_HUMAN] | 13 | 15 | 28 |
| Q07065 | Cytoskeleton-associated protein 4 OS=Homo sapiens GN=CKAP4 PE=1 SV=2 - [CKAP4_HUMAN] | 21 | 15 | 26 |
| P04843 | Dolichyl-diphosphooligosaccharide--protein glycosyltransferase subunit 1 OS=Homo sapiens GN=RPN1 PE=1 SV=1 - [RPN1_HUMAN] | 18 | 15 | 24 |
| P33176 | Kinesin-1 heavy chain OS=Homo sapiens GN=KIF5B PE=1 SV=1 - [KINH_HUMAN] | 16 | 15 | 22 |
| P31946 | 14-3-3 protein beta/alpha OS=Homo sapiens GN=YWHAB PE=1 SV=3 - [1433B_HUMAN] | 9 | 15 | 20 |
| P43243 | Matrin-3 OS=Homo sapiens GN=MATR3 PE=1 SV=2 - [MATR3_HUMAN] | 16 | 15 | 18 |
| P50991 | T-complex protein 1 subunit delta OS=Homo sapiens GN=CCT4 PE=1 SV=4 - [TCPD_HUMAN] | 13 | 15 | 16 |
| P23526 | Adenosylhomocysteinase OS=Homo sapiens GN=AHCY PE=1 SV=4 - [SAHH_HUMAN] | 13 | 15 | 16 |
| Q99623 | Prohibitin-2 OS=Homo sapiens GN=PHB2 PE=1 SV=2 - [PHB2_HUMAN] | 12 | 15 | 16 |
| P11940 | Polyadenylate-binding protein 1 OS=Homo sapiens GN=PABPC1 PE=1 SV=2 - [PABP1_HUMAN] | 10 | 15 | 16 |
| Q14152 | Eukaryotic translation initiation factor 3 subunit A OS=Homo sapiens GN=EIF3A PE=1 SV=1 - [EIF3A_HUMAN] | 17 | 15 | 15 |
| P26640 | Valyl-tRNA synthetase OS=Homo sapiens GN=VARS PE=1 SV=4 - [SYVC_HUMAN] | 12 | 15 | 10 |
| P05388 | 60S acidic ribosomal protein P0 OS=Homo sapiens GN=RPLP0 PE=1 SV=1 - [RLA0_HUMAN] | 10 | 15 | 9 |
| P49588 | Alanyl-tRNA synthetase. cytoplasmic OS=Homo sapiens GN=AARS PE=1 SV=2 - [SYAC_HUMAN] | 24 | 14 | 30 |
| P13667 | Protein disulfide-isomerase A4 OS=Homo sapiens GN=PDIA4 PE=1 SV=2 - [PDIA4_HUMAN] | 18 | 14 | 20 |
| P53618 | Coatomer subunit beta OS=Homo sapiens GN=COPB1 PE=1 SV=3 - [COPB_HUMAN] | 20 | 14 | 19 |
| Q96QK1 | Vacuolar protein sorting-associated protein 35 OS=Homo sapiens GN=VPS35 PE=1 SV=2 - [VPS35_HUMAN] | 16 | 14 | 18 |
| P07814 | Bifunctional aminoacyl-tRNA synthetase OS=Homo sapiens GN=EPRS PE=1 SV=5 - [SYEP_HUMAN] | 19 | 14 | 17 |
| P35232 | Prohibitin OS=Homo sapiens GN=PHB PE=1 SV=1 - [PHB_HUMAN] | 14 | 14 | 16 |
| Q9Y262 | Eukaryotic translation initiation factor 3 subunit L OS=Homo sapiens GN=EIF3L PE=1 SV=1 - [EIF3L_HUMAN] | 15 | 14 | 15 |
| P08865 | 40S ribosomal protein SA OS=Homo sapiens GN=RPSA PE=1 SV=4 - [RSSA_HUMAN] | 13 | 14 | 15 |
| P23396 | 40S ribosomal protein S3 OS=Homo sapiens GN=RPS3 PE=1 SV=2 - [RS3_HUMAN] | 14 | 14 | 13 |
| P12429 | Annexin A3 OS=Homo sapiens GN=ANXA3 PE=1 SV=3 - [ANXA3_HUMAN] | 14 | 14 | 13 |
| P06737 | Glycogen phosphorylase. liver form OS=Homo sapiens GN=PYGL PE=1 SV=4 - [PYGL_HUMAN] | 14 | 14 | 13 |
| O60506 | Heterogeneous nuclear ribonucleoprotein Q OS=Homo sapiens GN=SYNCRIP PE=1 SV=2 - [HNRPQ_HUMAN] | 9 | 14 | 13 |
| P67809 | Nuclease-sensitive element-binding protein 1 OS=Homo sapiens GN=YBX1 PE=1 SV=3 - [YBOX1_HUMAN] | 7 | 14 | 13 |
| P08133 | Annexin A6 OS=Homo sapiens GN=ANXA6 PE=1 SV=3 - [ANXA6_HUMAN] | 17 | 14 | 12 |
| O43242 | 26S proteasome non-ATPase regulatory subunit 3 OS=Homo sapiens GN=PSMD3 PE=1 SV=2 - [PSMD3_HUMAN] | 14 | 14 | 7 |
| Q04828 | Aldo-keto reductase family 1 member C1 OS=Homo sapiens GN=AKR1C1 PE=1 SV=1 - [AK1C1_HUMAN] | 5 | 14 | 5 |
| Q16658 | Fascin OS=Homo sapiens GN=FSCN1 PE=1 SV=3 - [FSCN1_HUMAN] | 11 | 14 | 3 |
| P09525 | Annexin A4 OS=Homo sapiens GN=ANXA4 PE=1 SV=4 - [ANXA4_HUMAN] | 18 | 13 | 26 |
| P05023 | Sodium/potassium-transporting ATPase subunit alpha-1 OS=Homo sapiens GN=ATP1A1 PE=1 SV=1 - [AT1A1_HUMAN] | 12 | 13 | 23 |
| P40926 | Malate dehydrogenase. mitochondrial OS=Homo sapiens GN=MDH2 PE=1 SV=3 - [MDHM_HUMAN] | 13 | 13 | 19 |
| P49411 | Elongation factor Tu. mitochondrial OS=Homo sapiens GN=TUFM PE=1 SV=2 - [EFTU_HUMAN] | 17 | 13 | 16 |
| O00571 | ATP-dependent RNA helicase DDX3X OS=Homo sapiens GN=DDX3X PE=1 SV=3 - [DDX3X_HUMAN] | 13 | 13 | 16 |
| P47897 | Glutaminyl-tRNA synthetase OS=Homo sapiens GN=QARS PE=1 SV=1 - [SYQ_HUMAN] | 14 | 13 | 15 |
| Q04917 | 14-3-3 protein eta OS=Homo sapiens GN=YWHAH PE=1 SV=4 - [1433F_HUMAN] | 11 | 13 | 15 |
| P20700 | Lamin-B1 OS=Homo sapiens GN=LMNB1 PE=1 SV=2 - [LMNB1_HUMAN] | 11 | 13 | 15 |
| P40939 | Trifunctional enzyme subunit alpha. mitochondrial OS=Homo sapiens GN=HADHA PE=1 SV=2 - [ECHA_HUMAN] | 11 | 13 | 13 |
| Q15417 | Calponin-3 OS=Homo sapiens GN=CNN3 PE=1 SV=1 - [CNN3_HUMAN] | 9 | 13 | 11 |
| Q02790 | Peptidyl-prolyl cis-trans isomerase FKBP4 OS=Homo sapiens GN=FKBP4 PE=1 SV=3 - [FKBP4_HUMAN] | 12 | 13 | 10 |
| O00231 | 26S proteasome non-ATPase regulatory subunit 11 OS=Homo sapiens GN=PSMD11 PE=1 SV=3 - [PSD11_HUMAN] | 14 | 13 | 9 |
| O43491 | Band 4.1-like protein 2 OS=Homo sapiens GN=EPB41L2 PE=1 SV=1 - [E41L2_HUMAN] | 12 | 13 | 5 |
| Q9P2J5 | Leucyl-tRNA synthetase. cytoplasmic OS=Homo sapiens GN=LARS PE=1 SV=2 - [SYLC_HUMAN] | 14 | 13 | 4 |
| Q9HAV4 | Exportin-5 OS=Homo sapiens GN=XPO5 PE=1 SV=1 - [XPO5_HUMAN] | 12 | 13 | 2 |
| P30041 | Peroxiredoxin-6 OS=Homo sapiens GN=PRDX6 PE=1 SV=3 - [PRDX6_HUMAN] | 13 | 12 | 23 |
| Q8WUM4 | Programmed cell death 6-interacting protein OS=Homo sapiens GN=PDCD6IP PE=1 SV=1 - [PDC6I_HUMAN] | 16 | 12 | 20 |
| O75533 | Splicing factor 3B subunit 1 OS=Homo sapiens GN=SF3B1 PE=1 SV=3 - [SF3B1_HUMAN] | 16 | 12 | 20 |
| P35241 | Radixin OS=Homo sapiens GN=RDX PE=1 SV=1 - [RADI_HUMAN] | 8 | 12 | 17 |
| Q6P2Q9 | Pre-mRNA-processing-splicing factor 8 OS=Homo sapiens GN=PRPF8 PE=1 SV=2 - [PRP8_HUMAN] | 18 | 12 | 16 |
| Q16531 | DNA damage-binding protein 1 OS=Homo sapiens GN=DDB1 PE=1 SV=1 - [DDB1_HUMAN] | 16 | 12 | 16 |
| P22234 | Multifunctional protein ADE2 OS=Homo sapiens GN=PAICS PE=1 SV=3 - [PUR6_HUMAN] | 14 | 12 | 15 |
| P62826 | GTP-binding nuclear protein Ran OS=Homo sapiens GN=RAN PE=1 SV=3 - [RAN_HUMAN] | 10 | 12 | 15 |
| P13797 | Plastin-3 OS=Homo sapiens GN=PLS3 PE=1 SV=4 - [PLST_HUMAN] | 10 | 12 | 15 |
| P55884 | Eukaryotic translation initiation factor 3 subunit B OS=Homo sapiens GN=EIF3B PE=1 SV=3 - [EIF3B_HUMAN] | 17 | 12 | 14 |
| Q9BQG0 | Myb-binding protein 1A OS=Homo sapiens GN=MYBBP1A PE=1 SV=2 - [MBB1A_HUMAN] | 16 | 12 | 14 |
| P05455 | Lupus La protein OS=Homo sapiens GN=SSB PE=1 SV=2 - [LA_HUMAN] | 15 | 12 | 14 |
| Q13838 | Spliceosome RNA helicase BAT1 OS=Homo sapiens GN=BAT1 PE=1 SV=1 - [UAP56_HUMAN] | 11 | 12 | 14 |
| Q92945 | Far upstream element-binding protein 2 OS=Homo sapiens GN=KHSRP PE=1 SV=4 - [FUBP2_HUMAN] | 13 | 12 | 13 |
| Q9UJZ1 | Stomatin-like protein 2 OS=Homo sapiens GN=STOML2 PE=1 SV=1 - [STML2_HUMAN] | 11 | 12 | 13 |
| P40925 | Malate dehydrogenase. cytoplasmic OS=Homo sapiens GN=MDH1 PE=1 SV=4 - [MDHC_HUMAN] | 11 | 12 | 13 |
| P35606 | Coatomer subunit beta' OS=Homo sapiens GN=COPB2 PE=1 SV=2 - [COPB2_HUMAN] | 15 | 12 | 12 |
| P48643 | T-complex protein 1 subunit epsilon OS=Homo sapiens GN=CCT5 PE=1 SV=1 - [TCPE_HUMAN] | 13 | 12 | 12 |
| P27824 | Calnexin OS=Homo sapiens GN=CANX PE=1 SV=2 - [CALX_HUMAN] | 13 | 12 | 12 |
| P67936 | Tropomyosin alpha-4 chain OS=Homo sapiens GN=TPM4 PE=1 SV=3 - [TPM4_HUMAN] | 7 | 12 | 12 |
| O00410 | Importin-5 OS=Homo sapiens GN=IPO5 PE=1 SV=4 - [IPO5_HUMAN] | 14 | 12 | 11 |
| Q15393 | Splicing factor 3B subunit 3 OS=Homo sapiens GN=SF3B3 PE=1 SV=4 - [SF3B3_HUMAN] | 18 | 12 | 10 |
| P27816 | Microtubule-associated protein 4 OS=Homo sapiens GN=MAP4 PE=1 SV=3 - [MAP4_HUMAN] | 12 | 12 | 10 |
| P41091 | Eukaryotic translation initiation factor 2 subunit 3 OS=Homo sapiens GN=EIF2S3 PE=1 SV=3 - [IF2G_HUMAN] | 12 | 12 | 9 |
| P61247 | 40S ribosomal protein S3a OS=Homo sapiens GN=RPS3A PE=1 SV=2 - [RS3A_HUMAN] | 9 | 12 | 9 |
| Q13263 | Transcription intermediary factor 1-beta OS=Homo sapiens GN=TRIM28 PE=1 SV=5 - [TIF1B_HUMAN] | 12 | 12 | 8 |
| P18124 | 60S ribosomal protein L7 OS=Homo sapiens GN=RPL7 PE=1 SV=1 - [RL7_HUMAN] | 13 | 12 | 7 |
| P22102 | Trifunctional purine biosynthetic protein adenosine-3 OS=Homo sapiens GN=GART PE=1 SV=1 - [PUR2_HUMAN] | 12 | 12 | 7 |
| P45974 | Ubiquitin carboxyl-terminal hydrolase 5 OS=Homo sapiens GN=USP5 PE=1 SV=2 - [UBP5_HUMAN] | 13 | 12 | 6 |
| P12081 | Histidyl-tRNA synthetase. cytoplasmic OS=Homo sapiens GN=HARS PE=1 SV=2 - [SYHC_HUMAN] | 12 | 12 | 4 |
| P54577 | Tyrosyl-tRNA synthetase. cytoplasmic OS=Homo sapiens GN=YARS PE=1 SV=4 - [SYYC_HUMAN] | 11 | 12 | 4 |
| P22309 | UDP-glucuronosyltransferase 1-1 OS=Homo sapiens OX=9606 GN=UGT1A1 PE=1 SV=1 - [UD11_HUMAN] | 6 | 12 | 0 |
| Q9Y678 | Coatomer subunit gamma OS=Homo sapiens GN=COPG PE=1 SV=1 - [COPG_HUMAN] | 18 | 11 | 30 |
| P05091 | Aldehyde dehydrogenase. mitochondrial OS=Homo sapiens GN=ALDH2 PE=1 SV=2 - [ALDH2_HUMAN] | 21 | 11 | 27 |
| Q10567 | AP-1 complex subunit beta-1 OS=Homo sapiens GN=AP1B1 PE=1 SV=2 - [AP1B1_HUMAN] | 8 | 11 | 20 |
| P51659 | Peroxisomal multifunctional enzyme type 2 OS=Homo sapiens GN=HSD17B4 PE=1 SV=3 - [DHB4_HUMAN] | 18 | 11 | 19 |
| Q15084 | Protein disulfide-isomerase A6 OS=Homo sapiens GN=PDIA6 PE=1 SV=1 - [PDIA6_HUMAN] | 11 | 11 | 19 |
| P63241 | Eukaryotic translation initiation factor 5A-1 OS=Homo sapiens GN=EIF5A PE=1 SV=2 - [IF5A1_HUMAN] | 11 | 11 | 17 |
| P18669 | Phosphoglycerate mutase 1 OS=Homo sapiens GN=PGAM1 PE=1 SV=2 - [PGAM1_HUMAN] | 10 | 11 | 16 |
| O75083 | WD repeat-containing protein 1 OS=Homo sapiens GN=WDR1 PE=1 SV=4 - [WDR1_HUMAN] | 15 | 11 | 15 |
| O95373 | Importin-7 OS=Homo sapiens GN=IPO7 PE=1 SV=1 - [IPO7_HUMAN] | 15 | 11 | 14 |
| Q99829 | Copine-1 OS=Homo sapiens GN=CPNE1 PE=1 SV=1 - [CPNE1_HUMAN] | 12 | 11 | 13 |
| P09972 | Fructose-bisphosphate aldolase C OS=Homo sapiens GN=ALDOC PE=1 SV=2 - [ALDOC_HUMAN] | 9 | 11 | 13 |
| Q9H0U4 | Ras-related protein Rab-1B OS=Homo sapiens GN=RAB1B PE=1 SV=1 - [RAB1B_HUMAN] | 2 | 11 | 13 |
| P37837 | Transaldolase OS=Homo sapiens GN=TALDO1 PE=1 SV=2 - [TALDO_HUMAN] | 14 | 11 | 12 |
| P49368 | T-complex protein 1 subunit gamma OS=Homo sapiens GN=CCT3 PE=1 SV=4 - [TCPG_HUMAN] | 13 | 11 | 12 |
| O14818 | Proteasome subunit alpha type-7 OS=Homo sapiens GN=PSMA7 PE=1 SV=1 - [PSA7_HUMAN] | 10 | 11 | 12 |
| P36578 | 60S ribosomal protein L4 OS=Homo sapiens GN=RPL4 PE=1 SV=5 - [RL4_HUMAN] | 15 | 11 | 11 |
| Q16555 | Dihydropyrimidinase-related protein 2 OS=Homo sapiens GN=DPYSL2 PE=1 SV=1 - [DPYL2_HUMAN] | 12 | 11 | 11 |
| P52272 | Heterogeneous nuclear ribonucleoprotein M OS=Homo sapiens GN=HNRNPM PE=1 SV=3 - [HNRPM_HUMAN] | 12 | 11 | 11 |
| Q05682 | Caldesmon OS=Homo sapiens GN=CALD1 PE=1 SV=3 - [CALD1_HUMAN] | 12 | 11 | 11 |
| Q00341 | Vigilin OS=Homo sapiens GN=HDLBP PE=1 SV=2 - [VIGLN_HUMAN] | 13 | 11 | 10 |
| Q08257 | Quinone oxidoreductase OS=Homo sapiens GN=CRYZ PE=1 SV=1 - [QOR_HUMAN] | 11 | 11 | 10 |
| P55786 | Puromycin-sensitive aminopeptidase OS=Homo sapiens GN=NPEPPS PE=1 SV=2 - [PSA_HUMAN] | 13 | 11 | 8 |
| P28288 | ATP-binding cassette sub-family D member 3 OS=Homo sapiens GN=ABCD3 PE=1 SV=1 - [ABCD3_HUMAN] | 11 | 11 | 8 |
| O43390 | Heterogeneous nuclear ribonucleoprotein R OS=Homo sapiens GN=HNRNPR PE=1 SV=1 - [HNRPR_HUMAN] | 8 | 11 | 8 |
| O60664 | Perilipin-3 OS=Homo sapiens GN=PLIN3 PE=1 SV=3 - [PLIN3_HUMAN] | 9 | 11 | 7 |
| Q13620 | Cullin-4B OS=Homo sapiens GN=CUL4B PE=1 SV=4 - [CUL4B_HUMAN] | 7 | 11 | 7 |
| P41250 | Glycine--tRNA ligase OS=Homo sapiens GN=GARS PE=1 SV=3 - [SYG_HUMAN] | 11 | 11 | 6 |
| P52907 | F-actin-capping protein subunit alpha-1 OS=Homo sapiens GN=CAPZA1 PE=1 SV=3 - [CAZA1_HUMAN] | 8 | 11 | 6 |
| P04899 | Guanine nucleotide-binding protein G(i) subunit alpha-2 OS=Homo sapiens GN=GNAI2 PE=1 SV=3 - [GNAI2_HUMAN] | 4 | 11 | 3 |
| Q09666 | Neuroblast differentiation-associated protein AHNAK OS=Homo sapiens GN=AHNAK PE=1 SV=2 - [AHNK_HUMAN] | 11 | 11 | 0 |
| P09382 | Galectin-1 OS=Homo sapiens GN=LGALS1 PE=1 SV=2 - [LEG1_HUMAN] | 8 | 11 | 0 |
| P36871 | Phosphoglucomutase-1 OS=Homo sapiens GN=PGM1 PE=1 SV=3 - [PGM1_HUMAN] | 19 | 10 | 24 |
| P13804 | Electron transfer flavoprotein subunit alpha. mitochondrial OS=Homo sapiens GN=ETFA PE=1 SV=1 - [ETFA_HUMAN] | 12 | 10 | 19 |
| O75643 | U5 small nuclear ribonucleoprotein 200 kDa helicase OS=Homo sapiens GN=SNRNP200 PE=1 SV=2 - [U520_HUMAN] | 19 | 10 | 18 |
| P06744 | Glucose-6-phosphate isomerase OS=Homo sapiens GN=GPI PE=1 SV=4 - [G6PI_HUMAN] | 14 | 10 | 18 |
| Q12905 | Interleukin enhancer-binding factor 2 OS=Homo sapiens GN=ILF2 PE=1 SV=2 - [ILF2_HUMAN] | 13 | 10 | 18 |
| P61981 | 14-3-3 protein gamma OS=Homo sapiens GN=YWHAG PE=1 SV=2 - [1433G_HUMAN] | 7 | 10 | 17 |
| Q9UQ80 | Proliferation-associated protein 2G4 OS=Homo sapiens GN=PA2G4 PE=1 SV=3 - [PA2G4_HUMAN] | 14 | 10 | 16 |
| Q99497 | Protein DJ-1 OS=Homo sapiens GN=PARK7 PE=1 SV=2 - [PARK7_HUMAN] | 10 | 10 | 14 |
| P12236 | ADP/ATP translocase 3 OS=Homo sapiens GN=SLC25A6 PE=1 SV=4 - [ADT3_HUMAN] | 2 | 10 | 13 |
| Q99873 | Protein arginine N-methyltransferase 1 OS=Homo sapiens OX=9606 GN=PRMT1 PE=1 SV=3 - [ANM1_HUMAN] | 12 | 10 | 12 |
| P04792 | Heat shock protein beta-1 OS=Homo sapiens GN=HSPB1 PE=1 SV=2 - [HSPB1_HUMAN] | 11 | 10 | 12 |
| P14868 | Aspartyl-tRNA synthetase. cytoplasmic OS=Homo sapiens GN=DARS PE=1 SV=2 - [SYDC_HUMAN] | 14 | 10 | 11 |
| P62701 | 40S ribosomal protein S4. X isoform OS=Homo sapiens GN=RPS4X PE=1 SV=2 - [RS4X_HUMAN] | 10 | 10 | 11 |
| Q03252 | Lamin-B2 OS=Homo sapiens GN=LMNB2 PE=1 SV=4 - [LMNB2_HUMAN] | 9 | 10 | 11 |
| P15880 | 40S ribosomal protein S2 OS=Homo sapiens GN=RPS2 PE=1 SV=2 - [RS2_HUMAN] | 10 | 10 | 10 |
| Q13310 | Polyadenylate-binding protein 4 OS=Homo sapiens GN=PABPC4 PE=1 SV=1 - [PABP4_HUMAN] | 4 | 10 | 10 |
| Q14914 | Prostaglandin reductase 1 OS=Homo sapiens GN=PTGR1 PE=1 SV=2 - [PTGR1_HUMAN] | 9 | 10 | 9 |
| Q9NR30 | Nucleolar RNA helicase 2 OS=Homo sapiens GN=DDX21 PE=1 SV=5 - [DDX21_HUMAN] | 9 | 10 | 9 |
| P46783 | 40S ribosomal protein S10 OS=Homo sapiens GN=RPS10 PE=1 SV=1 - [RS10_HUMAN] | 5 | 10 | 9 |
| Q99714 | 3-hydroxyacyl-CoA dehydrogenase type-2 OS=Homo sapiens GN=HSD17B10 PE=1 SV=3 - [HCD2_HUMAN] | 8 | 10 | 8 |
| P61026 | Ras-related protein Rab-10 OS=Homo sapiens GN=RAB10 PE=1 SV=1 - [RAB10_HUMAN] | 4 | 10 | 8 |
| P08754 | Guanine nucleotide-binding protein G(k) subunit alpha OS=Homo sapiens GN=GNAI3 PE=1 SV=3 - [GNAI3_HUMAN] | 4 | 10 | 7 |
| O14745 | Na(+)/H(+) exchange regulatory cofactor NHE-RF1 OS=Homo sapiens GN=SLC9A3R1 PE=1 SV=4 - [NHRF1_HUMAN] | 10 | 10 | 6 |
| Q02218 | 2-oxoglutarate dehydrogenase. mitochondrial OS=Homo sapiens GN=OGDH PE=1 SV=3 - [ODO1_HUMAN] | 10 | 10 | 3 |
| P07686 | Beta-hexosaminidase subunit beta OS=Homo sapiens GN=HEXB PE=1 SV=3 - [HEXB_HUMAN] | 9 | 10 | 3 |
| Q9NPH2 | Inositol-3-phosphate synthase 1 OS=Homo sapiens GN=ISYNA1 PE=1 SV=1 - [INO1_HUMAN] | 7 | 10 | 1 |
| P17655 | Calpain-2 catalytic subunit OS=Homo sapiens GN=CAPN2 PE=1 SV=6 - [CAN2_HUMAN] | 9 | 10 | 0 |
| P02786 | Transferrin receptor protein 1 OS=Homo sapiens GN=TFRC PE=1 SV=2 - [TFR1_HUMAN] | 18 | 9 | 20 |
| P52209 | 6-phosphogluconate dehydrogenase. decarboxylating OS=Homo sapiens GN=PGD PE=1 SV=3 - [6PGD_HUMAN] | 14 | 9 | 20 |
| P02768 | Serum albumin OS=Homo sapiens GN=ALB PE=1 SV=2 - [ALBU_HUMAN] | 11 | 9 | 19 |
| P17844 | Probable ATP-dependent RNA helicase DDX5 OS=Homo sapiens GN=DDX5 PE=1 SV=1 - [DDX5_HUMAN] | 12 | 9 | 16 |
| P35222 | Catenin beta-1 OS=Homo sapiens GN=CTNNB1 PE=1 SV=1 - [CTNB1_HUMAN] | 9 | 9 | 16 |
| P62081 | 40S ribosomal protein S7 OS=Homo sapiens GN=RPS7 PE=1 SV=1 - [RS7_HUMAN] | 10 | 9 | 15 |
| P31943 | Heterogeneous nuclear ribonucleoprotein H OS=Homo sapiens GN=HNRNPH1 PE=1 SV=4 - [HNRH1_HUMAN] | 9 | 9 | 15 |
| P12268 | Inosine-5'-monophosphate dehydrogenase 2 OS=Homo sapiens GN=IMPDH2 PE=1 SV=2 - [IMDH2_HUMAN] | 14 | 9 | 14 |
| P27695 | DNA-(apurinic or apyrimidinic site) lyase OS=Homo sapiens GN=APEX1 PE=1 SV=2 - [APEX1_HUMAN] | 11 | 9 | 13 |
| Q15436 | Protein transport protein Sec23A OS=Homo sapiens GN=SEC23A PE=1 SV=2 - [SC23A_HUMAN] | 12 | 9 | 12 |
| O43175 | D-3-phosphoglycerate dehydrogenase OS=Homo sapiens GN=PHGDH PE=1 SV=4 - [SERA_HUMAN] | 11 | 9 | 12 |
| P30086 | Phosphatidylethanolamine-binding protein 1 OS=Homo sapiens GN=PEBP1 PE=1 SV=3 - [PEBP1_HUMAN] | 10 | 9 | 12 |
| O43143 | Putative pre-mRNA-splicing factor ATP-dependent RNA helicase DHX15 OS=Homo sapiens GN=DHX15 PE=1 SV=2 - [DHX15_HUMAN] | 9 | 9 | 12 |
| P54578 | Ubiquitin carboxyl-terminal hydrolase 14 OS=Homo sapiens GN=USP14 PE=1 SV=3 - [UBP14_HUMAN] | 9 | 9 | 11 |
| P28074 | Proteasome subunit beta type-5 OS=Homo sapiens GN=PSMB5 PE=1 SV=3 - [PSB5_HUMAN] | 9 | 9 | 10 |
| P62269 | 40S ribosomal protein S18 OS=Homo sapiens GN=RPS18 PE=1 SV=3 - [RS18_HUMAN] | 8 | 9 | 10 |
| P36873 | Serine/threonine-protein phosphatase PP1-gamma catalytic subunit OS=Homo sapiens GN=PPP1CC PE=1 SV=1 - [PP1G_HUMAN] | 2 | 9 | 10 |
| Q92499 | ATP-dependent RNA helicase DDX1 OS=Homo sapiens GN=DDX1 PE=1 SV=2 - [DDX1_HUMAN] | 11 | 9 | 9 |
| P06748 | Nucleophosmin OS=Homo sapiens GN=NPM1 PE=1 SV=2 - [NPM_HUMAN] | 5 | 9 | 9 |
| P61006 | Ras-related protein Rab-8A OS=Homo sapiens GN=RAB8A PE=1 SV=1 - [RAB8A_HUMAN] | 3 | 9 | 9 |
| Q13011 | Delta(3.5)-Delta(2.4)-dienoyl-CoA isomerase. mitochondrial OS=Homo sapiens GN=ECH1 PE=1 SV=2 - [ECH1_HUMAN] | 8 | 9 | 8 |
| P56537 | Eukaryotic translation initiation factor 6 OS=Homo sapiens GN=EIF6 PE=1 SV=1 - [IF6_HUMAN] | 7 | 9 | 8 |
| P62140 | Serine/threonine-protein phosphatase PP1-beta catalytic subunit OS=Homo sapiens GN=PPP1CB PE=1 SV=3 - [PP1B_HUMAN] | 2 | 9 | 8 |
| P38159 | Heterogeneous nuclear ribonucleoprotein G OS=Homo sapiens GN=RBMX PE=1 SV=3 - [HNRPG_HUMAN] | 2 | 9 | 8 |
| P30566 | Adenylosuccinate lyase OS=Homo sapiens GN=ADSL PE=1 SV=2 - [PUR8_HUMAN] | 8 | 9 | 7 |
| P50552 | Vasodilator-stimulated phosphoprotein OS=Homo sapiens GN=VASP PE=1 SV=3 - [VASP_HUMAN] | 10 | 9 | 6 |
| Q8NBS9 | Thioredoxin domain-containing protein 5 OS=Homo sapiens GN=TXNDC5 PE=1 SV=2 - [TXND5_HUMAN] | 8 | 9 | 6 |
| P62241 | 40S ribosomal protein S8 OS=Homo sapiens GN=RPS8 PE=1 SV=2 - [RS8_HUMAN] | 7 | 9 | 6 |
| P55084 | Trifunctional enzyme subunit beta. mitochondrial OS=Homo sapiens GN=HADHB PE=1 SV=3 - [ECHB_HUMAN] | 7 | 9 | 6 |
| P11216 | Glycogen phosphorylase. brain form OS=Homo sapiens GN=PYGB PE=1 SV=5 - [PYGB_HUMAN] | 7 | 9 | 6 |
| P46777 | 60S ribosomal protein L5 OS=Homo sapiens GN=RPL5 PE=1 SV=3 - [RL5_HUMAN] | 5 | 9 | 6 |
| P48637 | Glutathione synthetase OS=Homo sapiens GN=GSS PE=1 SV=1 - [GSHB_HUMAN] | 10 | 9 | 5 |
| P30837 | Aldehyde dehydrogenase X. mitochondrial OS=Homo sapiens GN=ALDH1B1 PE=1 SV=3 - [AL1B1_HUMAN] | 7 | 9 | 5 |
| P05556 | Integrin beta-1 OS=Homo sapiens GN=ITGB1 PE=1 SV=2 - [ITB1_HUMAN] | 9 | 9 | 4 |
| Q16881 | Thioredoxin reductase 1. cytoplasmic OS=Homo sapiens GN=TXNRD1 PE=1 SV=3 - [TRXR1_HUMAN] | 9 | 9 | 3 |
| P16152 | Carbonyl reductase [NADPH] 1 OS=Homo sapiens GN=CBR1 PE=1 SV=3 - [CBR1_HUMAN] | 7 | 9 | 3 |
| Q9NZN4 | EH domain-containing protein 2 OS=Homo sapiens GN=EHD2 PE=1 SV=2 - [EHD2_HUMAN] | 8 | 9 | 0 |
| P49748 | Very long-chain specific acyl-CoA dehydrogenase. mitochondrial OS=Homo sapiens GN=ACADVL PE=1 SV=1 - [ACADV_HUMAN] | 16 | 8 | 19 |
| P08183 | Multidrug resistance protein 1 OS=Homo sapiens GN=ABCB1 PE=1 SV=3 - [MDR1_HUMAN] | 13 | 8 | 19 |
| Q06323 | Proteasome activator complex subunit 1 OS=Homo sapiens GN=PSME1 PE=1 SV=1 - [PSME1_HUMAN] | 10 | 8 | 16 |
| O75390 | Citrate synthase. mitochondrial OS=Homo sapiens GN=CS PE=1 SV=2 - [CISY_HUMAN] | 11 | 8 | 15 |
| P51991 | Heterogeneous nuclear ribonucleoprotein A3 OS=Homo sapiens GN=HNRNPA3 PE=1 SV=2 - [ROA3_HUMAN] | 11 | 8 | 14 |
| O95394 | Phosphoacetylglucosamine mutase OS=Homo sapiens GN=PGM3 PE=1 SV=1 - [AGM1_HUMAN] | 10 | 8 | 14 |
| Q16851 | UTP--glucose-1-phosphate uridylyltransferase OS=Homo sapiens GN=UGP2 PE=1 SV=5 - [UGPA_HUMAN] | 12 | 8 | 13 |
| P33993 | DNA replication licensing factor MCM7 OS=Homo sapiens GN=MCM7 PE=1 SV=4 - [MCM7_HUMAN] | 12 | 8 | 13 |
| P39656 | Dolichyl-diphosphooligosaccharide--protein glycosyltransferase 48 kDa subunit OS=Homo sapiens GN=DDOST PE=1 SV=4 - [OST48_HUMAN] | 11 | 8 | 13 |
| P07910 | Heterogeneous nuclear ribonucleoproteins C1/C2 OS=Homo sapiens GN=HNRNPC PE=1 SV=4 - [HNRPC_HUMAN] | 9 | 8 | 13 |
| P46782 | 40S ribosomal protein S5 OS=Homo sapiens GN=RPS5 PE=1 SV=4 - [RS5_HUMAN] | 7 | 8 | 13 |
| Q12906 | Interleukin enhancer-binding factor 3 OS=Homo sapiens GN=ILF3 PE=1 SV=3 - [ILF3_HUMAN] | 12 | 8 | 12 |
| P23246 | Splicing factor. proline- and glutamine-rich OS=Homo sapiens GN=SFPQ PE=1 SV=2 - [SFPQ_HUMAN] | 10 | 8 | 12 |
| P05141 | ADP/ATP translocase 2 OS=Homo sapiens GN=SLC25A5 PE=1 SV=7 - [ADT2_HUMAN] | 4 | 8 | 12 |
| P09874 | Poly [ADP-ribose] polymerase 1 OS=Homo sapiens GN=PARP1 PE=1 SV=4 - [PARP1_HUMAN] | 12 | 8 | 11 |
| P14866 | Heterogeneous nuclear ribonucleoprotein L OS=Homo sapiens GN=HNRNPL PE=1 SV=2 - [HNRPL_HUMAN] | 11 | 8 | 11 |
| P21399 | Cytoplasmic aconitate hydratase OS=Homo sapiens GN=ACO1 PE=1 SV=3 - [ACOC_HUMAN] | 10 | 8 | 11 |
| Q9UNM6 | 26S proteasome non-ATPase regulatory subunit 13 OS=Homo sapiens GN=PSMD13 PE=1 SV=2 - [PSD13_HUMAN] | 9 | 8 | 11 |
| Q15365 | Poly(rC)-binding protein 1 OS=Homo sapiens GN=PCBP1 PE=1 SV=2 - [PCBP1_HUMAN] | 7 | 8 | 11 |
| P28482 | Mitogen-activated protein kinase 1 OS=Homo sapiens GN=MAPK1 PE=1 SV=3 - [MK01_HUMAN] | 6 | 8 | 11 |
| P31150 | Rab GDP dissociation inhibitor alpha OS=Homo sapiens GN=GDI1 PE=1 SV=2 - [GDIA_HUMAN] | 2 | 8 | 11 |
| Q92616 | eIF-2-alpha kinase activator GCN1 OS=Homo sapiens GN=GCN1 PE=1 SV=6 - [GCN1_HUMAN] | 11 | 8 | 10 |
| P13489 | Ribonuclease inhibitor OS=Homo sapiens GN=RNH1 PE=1 SV=2 - [RINI_HUMAN] | 10 | 8 | 10 |
| Q96AG4 | Leucine-rich repeat-containing protein 59 OS=Homo sapiens GN=LRRC59 PE=1 SV=1 - [LRC59_HUMAN] | 9 | 8 | 10 |
| P06753 | Tropomyosin alpha-3 chain OS=Homo sapiens GN=TPM3 PE=1 SV=2 - [TPM3_HUMAN] | 4 | 8 | 10 |
| P62136 | Serine/threonine-protein phosphatase PP1-alpha catalytic subunit OS=Homo sapiens GN=PPP1CA PE=1 SV=1 - [PP1A_HUMAN] | 2 | 8 | 10 |
| P43686 | 26S protease regulatory subunit 6B OS=Homo sapiens GN=PSMC4 PE=1 SV=2 - [PRS6B_HUMAN] | 11 | 8 | 9 |
| Q99460 | 26S proteasome non-ATPase regulatory subunit 1 OS=Homo sapiens GN=PSMD1 PE=1 SV=2 - [PSMD1_HUMAN] | 10 | 8 | 9 |
| P38117 | Electron transfer flavoprotein subunit beta OS=Homo sapiens GN=ETFB PE=1 SV=3 - [ETFB_HUMAN] | 9 | 8 | 9 |
| Q96FW1 | Ubiquitin thioesterase OTUB1 OS=Homo sapiens GN=OTUB1 PE=1 SV=2 - [OTUB1_HUMAN] | 9 | 8 | 9 |
| P61158 | Actin-related protein 3 OS=Homo sapiens GN=ACTR3 PE=1 SV=3 - [ARP3_HUMAN] | 9 | 8 | 9 |
| P00533 | Epidermal growth factor receptor OS=Homo sapiens GN=EGFR PE=1 SV=2 - [EGFR_HUMAN] | 9 | 8 | 9 |
| P20618 | Proteasome subunit beta type-1 OS=Homo sapiens GN=PSMB1 PE=1 SV=2 - [PSB1_HUMAN] | 8 | 8 | 9 |
| P00505 | Aspartate aminotransferase. mitochondrial OS=Homo sapiens GN=GOT2 PE=1 SV=3 - [AATM_HUMAN] | 8 | 8 | 9 |
| P60228 | Eukaryotic translation initiation factor 3 subunit E OS=Homo sapiens GN=EIF3E PE=1 SV=1 - [EIF3E_HUMAN] | 8 | 8 | 9 |
| Q9Y281 | Cofilin-2 OS=Homo sapiens GN=CFL2 PE=1 SV=1 - [COF2_HUMAN] | 2 | 8 | 9 |
| O15460 | Prolyl 4-hydroxylase subunit alpha-2 OS=Homo sapiens GN=P4HA2 PE=1 SV=1 - [P4HA2_HUMAN] | 11 | 8 | 8 |
| Q9H9B4 | Sideroflexin-1 OS=Homo sapiens GN=SFXN1 PE=1 SV=4 - [SFXN1_HUMAN] | 9 | 8 | 8 |
| P62495 | Eukaryotic peptide chain release factor subunit 1 OS=Homo sapiens GN=ETF1 PE=1 SV=3 - [ERF1_HUMAN] | 7 | 8 | 8 |
| P29966 | Myristoylated alanine-rich C-kinase substrate OS=Homo sapiens GN=MARCKS PE=1 SV=4 - [MARCS_HUMAN] | 6 | 8 | 8 |
| P52597 | Heterogeneous nuclear ribonucleoprotein F OS=Homo sapiens GN=HNRNPF PE=1 SV=3 - [HNRPF_HUMAN] | 4 | 8 | 8 |
| P48444 | Coatomer subunit delta OS=Homo sapiens GN=ARCN1 PE=1 SV=1 - [COPD_HUMAN] | 10 | 8 | 7 |
| Q9NTK5 | Obg-like ATPase 1 OS=Homo sapiens GN=OLA1 PE=1 SV=2 - [OLA1_HUMAN] | 8 | 8 | 7 |
| P62249 | 40S ribosomal protein S16 OS=Homo sapiens GN=RPS16 PE=1 SV=2 - [RS16_HUMAN] | 7 | 8 | 7 |
| P05198 | Eukaryotic translation initiation factor 2 subunit 1 OS=Homo sapiens GN=EIF2S1 PE=1 SV=3 - [IF2A_HUMAN] | 7 | 8 | 7 |
| P07339 | Cathepsin D OS=Homo sapiens GN=CTSD PE=1 SV=1 - [CATD_HUMAN] | 6 | 8 | 7 |
| P62873 | Guanine nucleotide-binding protein G(I)/G(S)/G(T) subunit beta-1 OS=Homo sapiens GN=GNB1 PE=1 SV=3 - [GBB1_HUMAN] | 4 | 8 | 7 |
| P46781 | 40S ribosomal protein S9 OS=Homo sapiens GN=RPS9 PE=1 SV=3 - [RS9_HUMAN] | 10 | 8 | 6 |
| Q9UHD8 | Septin-9 OS=Homo sapiens GN=SEPT9 PE=1 SV=2 - [SEPT9_HUMAN] | 9 | 8 | 6 |
| Q15019 | Septin-2 OS=Homo sapiens GN=SEPT2 PE=1 SV=1 - [SEPT2_HUMAN] | 8 | 8 | 6 |
| Q14847 | LIM and SH3 domain protein 1 OS=Homo sapiens GN=LASP1 PE=1 SV=2 - [LASP1_HUMAN] | 6 | 8 | 6 |
| P52565 | Rho GDP-dissociation inhibitor 1 OS=Homo sapiens GN=ARHGDIA PE=1 SV=3 - [GDIR1_HUMAN] | 5 | 8 | 6 |
| P51148 | Ras-related protein Rab-5C OS=Homo sapiens GN=RAB5C PE=1 SV=2 - [RAB5C_HUMAN] | 4 | 8 | 6 |
| P62333 | 26S protease regulatory subunit S10B OS=Homo sapiens GN=PSMC6 PE=1 SV=1 - [PRS10_HUMAN] | 7 | 8 | 5 |
| Q9Y696 | Chloride intracellular channel protein 4 OS=Homo sapiens GN=CLIC4 PE=1 SV=4 - [CLIC4_HUMAN] | 6 | 8 | 5 |
| P43304 | Glycerol-3-phosphate dehydrogenase. mitochondrial OS=Homo sapiens GN=GPD2 PE=1 SV=3 - [GPDM_HUMAN] | 7 | 8 | 4 |
| P26639 | Threonyl-tRNA synthetase. cytoplasmic OS=Homo sapiens GN=TARS PE=1 SV=3 - [SYTC_HUMAN] | 9 | 8 | 3 |
| P39023 | 60S ribosomal protein L3 OS=Homo sapiens GN=RPL3 PE=1 SV=2 - [RL3_HUMAN] | 7 | 8 | 3 |
| P23921 | Ribonucleoside-diphosphate reductase large subunit OS=Homo sapiens GN=RRM1 PE=1 SV=1 - [RIR1_HUMAN] | 7 | 8 | 3 |
| Q96AE4 | Far upstream element-binding protein 1 OS=Homo sapiens GN=FUBP1 PE=1 SV=3 - [FUBP1_HUMAN] | 7 | 8 | 3 |
| Q8N766 | Uncharacterized protein KIAA0090 OS=Homo sapiens GN=KIAA0090 PE=1 SV=1 - [K0090_HUMAN] | 8 | 8 | 2 |
| Q9UHN6 | Transmembrane protein 2 OS=Homo sapiens GN=TMEM2 PE=1 SV=1 - [TMEM2_HUMAN] | 8 | 8 | 1 |
| P43358 | Melanoma-associated antigen 4 OS=Homo sapiens GN=MAGEA4 PE=1 SV=2 - [MAGA4_HUMAN] | 6 | 8 | 0 |
| P19367 | Hexokinase-1 OS=Homo sapiens GN=HK1 PE=1 SV=3 - [HXK1_HUMAN] | 6 | 8 | 0 |
| Q9HAW8 | UDP-glucuronosyltransferase 1-10 OS=Homo sapiens GN=UGT1A10 PE=1 SV=1 - [UD110_HUMAN] | 4 | 8 | 0 |
| O75874 | Isocitrate dehydrogenase [NADP] cytoplasmic OS=Homo sapiens GN=IDH1 PE=1 SV=2 - [IDHC_HUMAN] | 13 | 7 | 27 |
| P16435 | NADPH--cytochrome P450 reductase OS=Homo sapiens GN=POR PE=1 SV=2 - [NCPR_HUMAN] | 20 | 7 | 22 |
| P48735 | Isocitrate dehydrogenase [NADP]. mitochondrial OS=Homo sapiens GN=IDH2 PE=1 SV=2 - [IDHP_HUMAN] | 17 | 7 | 20 |
| Q8WVM8 | Sec1 family domain-containing protein 1 OS=Homo sapiens GN=SCFD1 PE=1 SV=4 - [SCFD1_HUMAN] | 13 | 7 | 16 |
| Q99541 | Perilipin-2 OS=Homo sapiens GN=PLIN2 PE=1 SV=2 - [PLIN2_HUMAN] | 12 | 7 | 15 |
| P32119 | Peroxiredoxin-2 OS=Homo sapiens GN=PRDX2 PE=1 SV=5 - [PRDX2_HUMAN] | 9 | 7 | 15 |
| Q9UL46 | Proteasome activator complex subunit 2 OS=Homo sapiens GN=PSME2 PE=1 SV=4 - [PSME2_HUMAN] | 7 | 7 | 14 |
| P84077 | ADP-ribosylation factor 1 OS=Homo sapiens GN=ARF1 PE=1 SV=2 - [ARF1_HUMAN] | 5 | 7 | 14 |
| P30740 | Leukocyte elastase inhibitor OS=Homo sapiens GN=SERPINB1 PE=1 SV=1 - [ILEU_HUMAN] | 12 | 7 | 13 |
| Q99613 | Eukaryotic translation initiation factor 3 subunit C OS=Homo sapiens GN=EIF3C PE=1 SV=1 - [EIF3C_HUMAN] | 10 | 7 | 13 |
| P34897 | Serine hydroxymethyltransferase. mitochondrial OS=Homo sapiens GN=SHMT2 PE=1 SV=3 - [GLYM_HUMAN] | 11 | 7 | 12 |
| O95831 | Apoptosis-inducing factor 1. mitochondrial OS=Homo sapiens GN=AIFM1 PE=1 SV=1 - [AIFM1_HUMAN] | 11 | 7 | 12 |
| Q04637 | Eukaryotic translation initiation factor 4 gamma 1 OS=Homo sapiens GN=EIF4G1 PE=1 SV=4 - [IF4G1_HUMAN] | 11 | 7 | 12 |
| Q15366 | Poly(rC)-binding protein 2 OS=Homo sapiens GN=PCBP2 PE=1 SV=1 - [PCBP2_HUMAN] | 7 | 7 | 12 |
| P25787 | Proteasome subunit alpha type-2 OS=Homo sapiens GN=PSMA2 PE=1 SV=2 - [PSA2_HUMAN] | 6 | 7 | 12 |
| Q15233 | Non-POU domain-containing octamer-binding protein OS=Homo sapiens GN=NONO PE=1 SV=4 - [NONO_HUMAN] | 6 | 7 | 12 |
| Q9Y617 | Phosphoserine aminotransferase OS=Homo sapiens GN=PSAT1 PE=1 SV=2 - [SERC_HUMAN] | 12 | 7 | 11 |
| Q8TEX9 | Importin-4 OS=Homo sapiens GN=IPO4 PE=1 SV=2 - [IPO4_HUMAN] | 10 | 7 | 11 |
| P32969 | 60S ribosomal protein L9 OS=Homo sapiens GN=RPL9 PE=1 SV=1 - [RL9_HUMAN] | 7 | 7 | 11 |
| O00303 | Eukaryotic translation initiation factor 3 subunit F OS=Homo sapiens GN=EIF3F PE=1 SV=1 - [EIF3F_HUMAN] | 6 | 7 | 11 |
| P31948 | Stress-induced-phosphoprotein 1 OS=Homo sapiens GN=STIP1 PE=1 SV=1 - [STIP1_HUMAN] | 10 | 7 | 10 |
| P30048 | Thioredoxin-dependent peroxide reductase. mitochondrial OS=Homo sapiens GN=PRDX3 PE=1 SV=3 - [PRDX3_HUMAN] | 7 | 7 | 10 |
| Q9Y230 | RuvB-like 2 OS=Homo sapiens GN=RUVBL2 PE=1 SV=3 - [RUVB2_HUMAN] | 10 | 7 | 9 |
| O60716 | Catenin delta-1 OS=Homo sapiens GN=CTNND1 PE=1 SV=1 - [CTND1_HUMAN] | 10 | 7 | 9 |
| P25786 | Proteasome subunit alpha type-1 OS=Homo sapiens GN=PSMA1 PE=1 SV=1 - [PSA1_HUMAN] | 9 | 7 | 9 |
| P30044 | Peroxiredoxin-5. mitochondrial OS=Homo sapiens GN=PRDX5 PE=1 SV=4 - [PRDX5_HUMAN] | 9 | 7 | 9 |
| P08195 | 4F2 cell-surface antigen heavy chain OS=Homo sapiens GN=SLC3A2 PE=1 SV=3 - [4F2_HUMAN] | 8 | 7 | 9 |
| Q15029 | 116 kDa U5 small nuclear ribonucleoprotein component OS=Homo sapiens GN=EFTUD2 PE=1 SV=1 - [U5S1_HUMAN] | 8 | 7 | 9 |
| Q92930 | Ras-related protein Rab-8B OS=Homo sapiens GN=RAB8B PE=1 SV=2 - [RAB8B_HUMAN] | 2 | 7 | 9 |
| O00151 | PDZ and LIM domain protein 1 OS=Homo sapiens GN=PDLIM1 PE=1 SV=4 - [PDLI1_HUMAN] | 9 | 7 | 8 |
| P29692 | Elongation factor 1-delta OS=Homo sapiens GN=EEF1D PE=1 SV=5 - [EF1D_HUMAN] | 8 | 7 | 8 |
| Q12931 | Heat shock protein 75 kDa. mitochondrial OS=Homo sapiens GN=TRAP1 PE=1 SV=3 - [TRAP1_HUMAN] | 7 | 7 | 8 |
| P16615 | Sarcoplasmic/endoplasmic reticulum calcium ATPase 2 OS=Homo sapiens GN=ATP2A2 PE=1 SV=1 - [AT2A2_HUMAN] | 11 | 7 | 7 |
| Q96PK6 | RNA-binding protein 14 OS=Homo sapiens GN=RBM14 PE=1 SV=2 - [RBM14_HUMAN] | 9 | 7 | 7 |
| Q07020 | 60S ribosomal protein L18 OS=Homo sapiens GN=RPL18 PE=1 SV=2 - [RL18_HUMAN] | 7 | 7 | 7 |
| P16403 | Histone H1.2 OS=Homo sapiens GN=HIST1H1C PE=1 SV=2 - [H12_HUMAN] | 5 | 7 | 7 |
| P30084 | Enoyl-CoA hydratase. mitochondrial OS=Homo sapiens GN=ECHS1 PE=1 SV=4 - [ECHM_HUMAN] | 4 | 7 | 7 |
| P42224 | Signal transducer and activator of transcription 1-alpha/beta OS=Homo sapiens GN=STAT1 PE=1 SV=2 - [STAT1_HUMAN] | 9 | 7 | 6 |
| P45880 | Voltage-dependent anion-selective channel protein 2 OS=Homo sapiens GN=VDAC2 PE=1 SV=2 - [VDAC2_HUMAN] | 8 | 7 | 6 |
| P62277 | 40S ribosomal protein S13 OS=Homo sapiens GN=RPS13 PE=1 SV=2 - [RS13_HUMAN] | 7 | 7 | 6 |
| Q16181 | Septin-7 OS=Homo sapiens GN=SEPT7 PE=1 SV=2 - [SEPT7_HUMAN] | 7 | 7 | 6 |
| O00425 | Insulin-like growth factor 2 mRNA-binding protein 3 OS=Homo sapiens GN=IGF2BP3 PE=1 SV=2 - [IF2B3_HUMAN] | 6 | 7 | 6 |
| P62879 | Guanine nucleotide-binding protein G(I)/G(S)/G(T) subunit beta-2 OS=Homo sapiens GN=GNB2 PE=1 SV=3 - [GBB2_HUMAN] | 4 | 7 | 6 |
| Q14651 | Plastin-1 OS=Homo sapiens GN=PLS1 PE=1 SV=2 - [PLSI_HUMAN] | 3 | 7 | 6 |
| Q13492 | Phosphatidylinositol-binding clathrin assembly protein OS=Homo sapiens GN=PICALM PE=1 SV=2 - [PICAL_HUMAN] | 9 | 7 | 5 |
| P31930 | Cytochrome b-c1 complex subunit 1. mitochondrial OS=Homo sapiens GN=UQCRC1 PE=1 SV=3 - [QCR1_HUMAN] | 8 | 7 | 5 |
| Q9Y266 | Nuclear migration protein nudC OS=Homo sapiens GN=NUDC PE=1 SV=1 - [NUDC_HUMAN] | 8 | 7 | 5 |
| Q8NBQ5 | Estradiol 17-beta-dehydrogenase 11 OS=Homo sapiens GN=HSD17B11 PE=1 SV=3 - [DHB11_HUMAN] | 7 | 7 | 5 |
| Q9NSD9 | Phenylalanine--tRNA ligase beta subunit OS=Homo sapiens GN=FARSB PE=1 SV=3 - [SYFB_HUMAN] | 7 | 7 | 5 |
| P04632 | Calpain small subunit 1 OS=Homo sapiens GN=CAPNS1 PE=1 SV=1 - [CPNS1_HUMAN] | 5 | 7 | 5 |
| P62195 | 26S protease regulatory subunit 8 OS=Homo sapiens GN=PSMC5 PE=1 SV=1 - [PRS8_HUMAN] | 9 | 7 | 4 |
| Q13724 | Mannosyl-oligosaccharide glucosidase OS=Homo sapiens GN=MOGS PE=1 SV=5 - [MOGS_HUMAN] | 7 | 7 | 4 |
| O94973 | AP-2 complex subunit alpha-2 OS=Homo sapiens GN=AP2A2 PE=1 SV=2 - [AP2A2_HUMAN] | 4 | 7 | 4 |
| P49591 | Seryl-tRNA synthetase. cytoplasmic OS=Homo sapiens GN=SARS PE=1 SV=3 - [SYSC_HUMAN] | 7 | 7 | 3 |
| Q9ULV4 | Coronin-1C OS=Homo sapiens GN=CORO1C PE=1 SV=1 - [COR1C_HUMAN] | 7 | 7 | 3 |
| Q15008 | 26S proteasome non-ATPase regulatory subunit 6 OS=Homo sapiens GN=PSMD6 PE=1 SV=1 - [PSMD6_HUMAN] | 7 | 7 | 3 |
| P50995 | Annexin A11 OS=Homo sapiens GN=ANXA11 PE=1 SV=1 - [ANX11_HUMAN] | 7 | 7 | 3 |
| Q96AC1 | Fermitin family homolog 2 OS=Homo sapiens GN=FERMT2 PE=1 SV=1 - [FERM2_HUMAN] | 7 | 7 | 3 |
| P30040 | Endoplasmic reticulum resident protein 29 OS=Homo sapiens GN=ERP29 PE=1 SV=4 - [ERP29_HUMAN] | 6 | 7 | 3 |
| Q15181 | Inorganic pyrophosphatase OS=Homo sapiens GN=PPA1 PE=1 SV=2 - [IPYR_HUMAN] | 5 | 7 | 3 |
| Q7L1Q6 | Basic leucine zipper and W2 domain-containing protein 1 OS=Homo sapiens GN=BZW1 PE=1 SV=1 - [BZW1_HUMAN] | 5 | 7 | 3 |
| P62917 | 60S ribosomal protein L8 OS=Homo sapiens GN=RPL8 PE=1 SV=2 - [RL8_HUMAN] | 5 | 7 | 3 |
| Q9NPQ8 | Synembryn-A OS=Homo sapiens GN=RIC8A PE=1 SV=3 - [RIC8A_HUMAN] | 7 | 7 | 2 |
| O43237 | Cytoplasmic dynein 1 light intermediate chain 2 OS=Homo sapiens GN=DYNC1LI2 PE=1 SV=1 - [DC1L2_HUMAN] | 6 | 7 | 2 |
| O60443 | Non-syndromic hearing impairment protein 5 OS=Homo sapiens GN=DFNA5 PE=1 SV=2 - [DFNA5_HUMAN] | 7 | 7 | 1 |
| P23381 | Tryptophanyl-tRNA synthetase. cytoplasmic OS=Homo sapiens GN=WARS PE=1 SV=2 - [SYWC_HUMAN] | 5 | 7 | 1 |
| P80723 | Brain acid soluble protein 1 OS=Homo sapiens GN=BASP1 PE=1 SV=2 - [BASP1_HUMAN] | 7 | 7 | 0 |
| Q6YHK3 | CD109 antigen OS=Homo sapiens GN=CD109 PE=1 SV=2 - [CD109_HUMAN] | 7 | 7 | 0 |
| Q9NZM1 | Myoferlin OS=Homo sapiens GN=MYOF PE=1 SV=1 - [MYOF_HUMAN] | 7 | 7 | 0 |
| P19224 | UDP-glucuronosyltransferase 1-6 OS=Homo sapiens GN=UGT1A6 PE=1 SV=2 - [UD16_HUMAN] | 4 | 7 | 0 |
| Q01581 | Hydroxymethylglutaryl-CoA synthase. cytoplasmic OS=Homo sapiens GN=HMGCS1 PE=1 SV=2 - [HMCS1_HUMAN] | 12 | 6 | 16 |
| P00390 | Glutathione reductase. mitochondrial OS=Homo sapiens GN=GSR PE=1 SV=2 - [GSHR_HUMAN] | 11 | 6 | 16 |
| P00367 | Glutamate dehydrogenase 1. mitochondrial OS=Homo sapiens GN=GLUD1 PE=1 SV=2 - [DHE3_HUMAN] | 12 | 6 | 15 |
| P30154 | Serine/threonine-protein phosphatase 2A 65 kDa regulatory subunit A beta isoform OS=Homo sapiens GN=PPP2R1B PE=1 SV=3 - [2AAB_HUMAN] | 5 | 6 | 15 |
| Q14566 | DNA replication licensing factor MCM6 OS=Homo sapiens GN=MCM6 PE=1 SV=1 - [MCM6_HUMAN] | 12 | 6 | 14 |
| P38919 | Eukaryotic initiation factor 4A-III OS=Homo sapiens GN=EIF4A3 PE=1 SV=4 - [IF4A3_HUMAN] | 8 | 6 | 13 |
| Q13162 | Peroxiredoxin-4 OS=Homo sapiens GN=PRDX4 PE=1 SV=1 - [PRDX4_HUMAN] | 6 | 6 | 11 |
| O94979 | Protein transport protein Sec31A OS=Homo sapiens GN=SEC31A PE=1 SV=3 - [SC31A_HUMAN] | 12 | 6 | 10 |
| P42167 | Lamina-associated polypeptide 2. isoforms beta/gamma OS=Homo sapiens GN=TMPO PE=1 SV=2 - [LAP2B_HUMAN] | 2 | 6 | 10 |
| Q15046 | Lysyl-tRNA synthetase OS=Homo sapiens GN=KARS PE=1 SV=3 - [SYK_HUMAN] | 11 | 6 | 9 |
| P46977 | Dolichyl-diphosphooligosaccharide--protein glycosyltransferase subunit STT3A OS=Homo sapiens GN=STT3A PE=1 SV=2 - [STT3A_HUMAN] | 10 | 6 | 9 |
| P36776 | Lon protease homolog. mitochondrial OS=Homo sapiens GN=LONP1 PE=1 SV=2 - [LONM_HUMAN] | 10 | 6 | 9 |
| O43776 | Asparaginyl-tRNA synthetase. cytoplasmic OS=Homo sapiens GN=NARS PE=1 SV=1 - [SYNC_HUMAN] | 9 | 6 | 9 |
| P53992 | Protein transport protein Sec24C OS=Homo sapiens GN=SEC24C PE=1 SV=3 - [SC24C_HUMAN] | 9 | 6 | 9 |
| P23284 | Peptidyl-prolyl cis-trans isomerase B OS=Homo sapiens GN=PPIB PE=1 SV=2 - [PPIB_HUMAN] | 8 | 6 | 9 |
| O76003 | Glutaredoxin-3 OS=Homo sapiens GN=GLRX3 PE=1 SV=2 - [GLRX3_HUMAN] | 8 | 6 | 9 |
| Q9Y265 | RuvB-like 1 OS=Homo sapiens GN=RUVBL1 PE=1 SV=1 - [RUVB1_HUMAN] | 8 | 6 | 9 |
| O95486 | Protein transport protein Sec24A OS=Homo sapiens GN=SEC24A PE=1 SV=2 - [SC24A_HUMAN] | 8 | 6 | 9 |
| P14314 | Glucosidase 2 subunit beta OS=Homo sapiens GN=PRKCSH PE=1 SV=2 - [GLU2B_HUMAN] | 7 | 6 | 9 |
| P62805 | Histone H4 OS=Homo sapiens GN=HIST1H4A PE=1 SV=2 - [H4_HUMAN] | 6 | 6 | 9 |
| P62753 | 40S ribosomal protein S6 OS=Homo sapiens GN=RPS6 PE=1 SV=1 - [RS6_HUMAN] | 6 | 6 | 9 |
| P00387 | NADH-cytochrome b5 reductase 3 OS=Homo sapiens GN=CYB5R3 PE=1 SV=3 - [NB5R3_HUMAN] | 7 | 6 | 8 |
| P63092 | Guanine nucleotide-binding protein G(s) subunit alpha isoforms short OS=Homo sapiens GN=GNAS PE=1 SV=1 - [GNAS2_HUMAN] | 7 | 6 | 8 |
| Q9Y224 | UPF0568 protein C14orf166 OS=Homo sapiens GN=C14orf166 PE=1 SV=1 - [CN166_HUMAN] | 7 | 6 | 8 |
| P38606 | V-type proton ATPase catalytic subunit A OS=Homo sapiens GN=ATP6V1A PE=1 SV=2 - [VATA_HUMAN] | 7 | 6 | 8 |
| Q92841 | Probable ATP-dependent RNA helicase DDX17 OS=Homo sapiens GN=DDX17 PE=1 SV=2 - [DDX17_HUMAN] | 7 | 6 | 8 |
| P61224 | Ras-related protein Rap-1b OS=Homo sapiens GN=RAP1B PE=1 SV=1 - [RAP1B_HUMAN] | 6 | 6 | 8 |
| Q9BZZ5 | Apoptosis inhibitor 5 OS=Homo sapiens GN=API5 PE=1 SV=3 - [API5_HUMAN] | 6 | 6 | 8 |
| Q9Y277 | Voltage-dependent anion-selective channel protein 3 OS=Homo sapiens GN=VDAC3 PE=1 SV=1 - [VDAC3_HUMAN] | 4 | 6 | 8 |
| P04908 | Histone H2A type 1-B/E OS=Homo sapiens GN=HIST1H2AB PE=1 SV=2 - [H2A1B_HUMAN] | 2 | 6 | 8 |
| P15144 | Aminopeptidase N OS=Homo sapiens GN=ANPEP PE=1 SV=4 - [AMPN_HUMAN] | 10 | 6 | 7 |
| P46379 | Large proline-rich protein BAT3 OS=Homo sapiens GN=BAT3 PE=1 SV=2 - [BAT3_HUMAN] | 8 | 6 | 7 |
| P35579 | Myosin-9 OS=Homo sapiens GN=MYH9 PE=1 SV=4 - [MYH9_HUMAN] | 8 | 6 | 7 |
| P51149 | Ras-related protein Rab-7a OS=Homo sapiens GN=RAB7A PE=1 SV=1 - [RAB7A_HUMAN] | 7 | 6 | 7 |
| Q13347 | Eukaryotic translation initiation factor 3 subunit I OS=Homo sapiens GN=EIF3I PE=1 SV=1 - [EIF3I_HUMAN] | 7 | 6 | 7 |
| Q00325 | Phosphate carrier protein. mitochondrial OS=Homo sapiens GN=SLC25A3 PE=1 SV=2 - [MPCP_HUMAN] | 7 | 6 | 7 |
| P17858 | 6-phosphofructokinase. liver type OS=Homo sapiens GN=PFKL PE=1 SV=6 - [K6PL_HUMAN] | 7 | 6 | 7 |
| Q15631 | Translin OS=Homo sapiens GN=TSN PE=1 SV=1 - [TSN_HUMAN] | 4 | 6 | 7 |
| P40763 | Signal transducer and activator of transcription 3 OS=Homo sapiens GN=STAT3 PE=1 SV=2 - [STAT3_HUMAN] | 10 | 6 | 6 |
| O00429 | Dynamin-1-like protein OS=Homo sapiens GN=DNM1L PE=1 SV=2 - [DNM1L_HUMAN] | 9 | 6 | 6 |
| Q02978 | Mitochondrial 2-oxoglutarate/malate carrier protein OS=Homo sapiens GN=SLC25A11 PE=1 SV=3 - [M2OM_HUMAN] | 8 | 6 | 6 |
| P78344 | Eukaryotic translation initiation factor 4 gamma 2 OS=Homo sapiens GN=EIF4G2 PE=1 SV=1 - [IF4G2_HUMAN] | 8 | 6 | 6 |
| P48147 | Prolyl endopeptidase OS=Homo sapiens GN=PREP PE=1 SV=2 - [PPCE_HUMAN] | 7 | 6 | 6 |
| P48163 | NADP-dependent malic enzyme OS=Homo sapiens GN=ME1 PE=1 SV=1 - [MAOX_HUMAN] | 6 | 6 | 6 |
| Q96P70 | Importin-9 OS=Homo sapiens GN=IPO9 PE=1 SV=3 - [IPO9_HUMAN] | 6 | 6 | 6 |
| P30050 | 60S ribosomal protein L12 OS=Homo sapiens GN=RPL12 PE=1 SV=1 - [RL12_HUMAN] | 5 | 6 | 6 |
| P60953 | Cell division control protein 42 homolog OS=Homo sapiens GN=CDC42 PE=1 SV=2 - [CDC42_HUMAN] | 3 | 6 | 6 |
| Q9HB71 | Calcyclin-binding protein OS=Homo sapiens GN=CACYBP PE=1 SV=2 - [CYBP_HUMAN] | 7 | 6 | 5 |
| Q9Y3F4 | Serine-threonine kinase receptor-associated protein OS=Homo sapiens GN=STRAP PE=1 SV=1 - [STRAP_HUMAN] | 7 | 6 | 5 |
| Q27J81 | Inverted formin-2 OS=Homo sapiens GN=INF2 PE=1 SV=2 - [INF2_HUMAN] | 7 | 6 | 5 |
| P26373 | 60S ribosomal protein L13 OS=Homo sapiens GN=RPL13 PE=1 SV=4 - [RL13_HUMAN] | 6 | 6 | 5 |
| Q7L2H7 | Eukaryotic translation initiation factor 3 subunit M OS=Homo sapiens GN=EIF3M PE=1 SV=1 - [EIF3M_HUMAN] | 6 | 6 | 5 |
| P46060 | Ran GTPase-activating protein 1 OS=Homo sapiens GN=RANGAP1 PE=1 SV=1 - [RAGP1_HUMAN] | 6 | 6 | 5 |
| P49321 | Nuclear autoantigenic sperm protein OS=Homo sapiens GN=NASP PE=1 SV=2 - [NASP_HUMAN] | 6 | 6 | 5 |
| O43765 | Small glutamine-rich tetratricopeptide repeat-containing protein alpha OS=Homo sapiens GN=SGTA PE=1 SV=1 - [SGTA_HUMAN] | 5 | 6 | 5 |
| Q9UHG3 | Prenylcysteine oxidase 1 OS=Homo sapiens GN=PCYOX1 PE=1 SV=3 - [PCYOX_HUMAN] | 5 | 6 | 5 |
| Q9NQC3 | Reticulon-4 OS=Homo sapiens GN=RTN4 PE=1 SV=2 - [RTN4_HUMAN] | 4 | 6 | 5 |
| O43847 | Nardilysin OS=Homo sapiens OX=9606 GN=NRDC PE=1 SV=3 - [NRDC_HUMAN] | 7 | 6 | 4 |
| P62906 | 60S ribosomal protein L10a OS=Homo sapiens GN=RPL10A PE=1 SV=2 - [RL10A_HUMAN] | 6 | 6 | 4 |
| Q8N1G4 | Leucine-rich repeat-containing protein 47 OS=Homo sapiens GN=LRRC47 PE=1 SV=1 - [LRC47_HUMAN] | 6 | 6 | 4 |
| P49841 | Glycogen synthase kinase-3 beta OS=Homo sapiens GN=GSK3B PE=1 SV=2 - [GSK3B_HUMAN] | 5 | 6 | 4 |
| Q13177 | Serine/threonine-protein kinase PAK 2 OS=Homo sapiens GN=PAK2 PE=1 SV=3 - [PAK2_HUMAN] | 5 | 6 | 4 |
| P55209 | Nucleosome assembly protein 1-like 1 OS=Homo sapiens GN=NAP1L1 PE=1 SV=1 - [NP1L1_HUMAN] | 4 | 6 | 4 |
| O95433 | Activator of 90 kDa heat shock protein ATPase homolog 1 OS=Homo sapiens GN=AHSA1 PE=1 SV=1 - [AHSA1_HUMAN] | 7 | 6 | 3 |
| P51648 | Fatty aldehyde dehydrogenase OS=Homo sapiens GN=ALDH3A2 PE=1 SV=1 - [AL3A2_HUMAN] | 7 | 6 | 3 |
| Q07866 | Kinesin light chain 1 OS=Homo sapiens GN=KLC1 PE=1 SV=2 - [KLC1_HUMAN] | 6 | 6 | 3 |
| Q08945 | FACT complex subunit SSRP1 OS=Homo sapiens GN=SSRP1 PE=1 SV=1 - [SSRP1_HUMAN] | 6 | 6 | 3 |
| P50416 | Carnitine O-palmitoyltransferase 1. liver isoform OS=Homo sapiens GN=CPT1A PE=1 SV=2 - [CPT1A_HUMAN] | 6 | 6 | 3 |
| P60981 | Destrin OS=Homo sapiens GN=DSTN PE=1 SV=3 - [DEST_HUMAN] | 4 | 6 | 3 |
| Q9H3N1 | Thioredoxin-related transmembrane protein 1 OS=Homo sapiens GN=TMX1 PE=1 SV=1 - [TMX1_HUMAN] | 6 | 6 | 2 |
| P46087 | Putative ribosomal RNA methyltransferase NOP2 OS=Homo sapiens GN=NOP2 PE=1 SV=2 - [NOP2_HUMAN] | 6 | 6 | 2 |
| Q96TA1 | Niban-like protein 1 OS=Homo sapiens GN=FAM129B PE=1 SV=3 - [NIBL1_HUMAN] | 5 | 6 | 2 |
| O14974 | Protein phosphatase 1 regulatory subunit 12A OS=Homo sapiens GN=PPP1R12A PE=1 SV=1 - [MYPT1_HUMAN] | 4 | 6 | 2 |
| Q32MZ4 | Leucine-rich repeat flightless-interacting protein 1 OS=Homo sapiens GN=LRRFIP1 PE=1 SV=2 - [LRRF1_HUMAN] | 6 | 6 | 1 |
| P54868 | Hydroxymethylglutaryl-CoA synthase. mitochondrial OS=Homo sapiens GN=HMGCS2 PE=1 SV=1 - [HMCS2_HUMAN] | 5 | 6 | 1 |
| Q15758 | Neutral amino acid transporter B(0) OS=Homo sapiens GN=SLC1A5 PE=1 SV=2 - [AAAT_HUMAN] | 5 | 6 | 0 |
| Q14019 | Coactosin-like protein OS=Homo sapiens GN=COTL1 PE=1 SV=3 - [COTL1_HUMAN] | 4 | 6 | 0 |
| P21964 | Catechol O-methyltransferase OS=Homo sapiens GN=COMT PE=1 SV=2 - [COMT_HUMAN] | 4 | 6 | 0 |
| Q06210 | Glucosamine--fructose-6-phosphate aminotransferase [isomerizing] 1 OS=Homo sapiens GN=GFPT1 PE=1 SV=3 - [GFPT1_HUMAN] | 15 | 5 | 18 |
| P78417 | Glutathione S-transferase omega-1 OS=Homo sapiens GN=GSTO1 PE=1 SV=2 - [GSTO1_HUMAN] | 12 | 5 | 15 |
| Q9NYU2 | UDP-glucose:glycoprotein glucosyltransferase 1 OS=Homo sapiens GN=UGGT1 PE=1 SV=3 - [UGGG1_HUMAN] | 11 | 5 | 12 |
| Q9UNZ2 | NSFL1 cofactor p47 OS=Homo sapiens GN=NSFL1C PE=1 SV=2 - [NSF1C_HUMAN] | 10 | 5 | 12 |
| P52292 | Importin subunit alpha-2 OS=Homo sapiens GN=KPNA2 PE=1 SV=1 - [IMA2_HUMAN] | 8 | 5 | 11 |
| O00469 | Procollagen-lysine.2-oxoglutarate 5-dioxygenase 2 OS=Homo sapiens GN=PLOD2 PE=1 SV=2 - [PLOD2_HUMAN] | 10 | 5 | 10 |
| P07384 | Calpain-1 catalytic subunit OS=Homo sapiens GN=CAPN1 PE=1 SV=1 - [CAN1_HUMAN] | 10 | 5 | 10 |
| Q8N163 | Protein KIAA1967 OS=Homo sapiens GN=KIAA1967 PE=1 SV=2 - [K1967_HUMAN] | 10 | 5 | 10 |
| P60900 | Proteasome subunit alpha type-6 OS=Homo sapiens GN=PSMA6 PE=1 SV=1 - [PSA6_HUMAN] | 8 | 5 | 10 |
| P12955 | Xaa-Pro dipeptidase OS=Homo sapiens GN=PEPD PE=1 SV=3 - [PEPD_HUMAN] | 8 | 5 | 10 |
| Q92973 | Transportin-1 OS=Homo sapiens GN=TNPO1 PE=1 SV=2 - [TNPO1_HUMAN] | 8 | 5 | 10 |
| P31040 | Succinate dehydrogenase [ubiquinone] flavoprotein subunit. mitochondrial OS=Homo sapiens GN=SDHA PE=1 SV=2 - [DHSA_HUMAN] | 9 | 5 | 9 |
| P17174 | Aspartate aminotransferase. cytoplasmic OS=Homo sapiens GN=GOT1 PE=1 SV=3 - [AATC_HUMAN] | 7 | 5 | 9 |
| Q15274 | Nicotinate-nucleotide pyrophosphorylase [carboxylating] OS=Homo sapiens GN=QPRT PE=1 SV=3 - [NADC_HUMAN] | 7 | 5 | 9 |
| Q9BWD1 | Acetyl-CoA acetyltransferase. cytosolic OS=Homo sapiens GN=ACAT2 PE=1 SV=2 - [THIC_HUMAN] | 6 | 5 | 9 |
| P51665 | 26S proteasome non-ATPase regulatory subunit 7 OS=Homo sapiens GN=PSMD7 PE=1 SV=2 - [PSD7_HUMAN] | 6 | 5 | 9 |
| O15260 | Surfeit locus protein 4 OS=Homo sapiens GN=SURF4 PE=1 SV=3 - [SURF4_HUMAN] | 3 | 5 | 9 |
| P25205 | DNA replication licensing factor MCM3 OS=Homo sapiens GN=MCM3 PE=1 SV=3 - [MCM3_HUMAN] | 9 | 5 | 8 |
| P00491 | Purine nucleoside phosphorylase OS=Homo sapiens GN=NP PE=1 SV=2 - [PNPH_HUMAN] | 7 | 5 | 8 |
| Q9NVA2 | Septin-11 OS=Homo sapiens GN=SEPT11 PE=1 SV=3 - [SEP11_HUMAN] | 6 | 5 | 8 |
| P62314 | Small nuclear ribonucleoprotein Sm D1 OS=Homo sapiens GN=SNRPD1 PE=1 SV=1 - [SMD1_HUMAN] | 3 | 5 | 8 |
| P07951 | Tropomyosin beta chain OS=Homo sapiens GN=TPM2 PE=1 SV=1 - [TPM2_HUMAN] | 2 | 5 | 8 |
| Q9UMS4 | Pre-mRNA-processing factor 19 OS=Homo sapiens GN=PRPF19 PE=1 SV=1 - [PRP19_HUMAN] | 8 | 5 | 7 |
| P35998 | 26S protease regulatory subunit 7 OS=Homo sapiens GN=PSMC2 PE=1 SV=3 - [PRS7_HUMAN] | 8 | 5 | 7 |
| Q13561 | Dynactin subunit 2 OS=Homo sapiens GN=DCTN2 PE=1 SV=4 - [DCTN2_HUMAN] | 8 | 5 | 7 |
| Q16891 | Mitochondrial inner membrane protein OS=Homo sapiens GN=IMMT PE=1 SV=1 - [IMMT_HUMAN] | 8 | 5 | 7 |
| Q15691 | Microtubule-associated protein RP/EB family member 1 OS=Homo sapiens GN=MAPRE1 PE=1 SV=3 - [MARE1_HUMAN] | 7 | 5 | 7 |
| P09661 | U2 small nuclear ribonucleoprotein A' OS=Homo sapiens GN=SNRPA1 PE=1 SV=2 - [RU2A_HUMAN] | 7 | 5 | 7 |
| Q15907 | Ras-related protein Rab-11B OS=Homo sapiens GN=RAB11B PE=1 SV=4 - [RB11B_HUMAN] | 7 | 5 | 7 |
| Q9HDC9 | Adipocyte plasma membrane-associated protein OS=Homo sapiens GN=APMAP PE=1 SV=2 - [APMAP_HUMAN] | 7 | 5 | 7 |
| Q9H0D6 | 5'-3' exoribonuclease 2 OS=Homo sapiens GN=XRN2 PE=1 SV=1 - [XRN2_HUMAN] | 7 | 5 | 7 |
| Q01105 | Protein SET OS=Homo sapiens GN=SET PE=1 SV=3 - [SET_HUMAN] | 6 | 5 | 7 |
| Q07955 | Splicing factor. arginine/serine-rich 1 OS=Homo sapiens GN=SFRS1 PE=1 SV=2 - [SFRS1_HUMAN] | 6 | 5 | 7 |
| P17980 | 26S protease regulatory subunit 6A OS=Homo sapiens GN=PSMC3 PE=1 SV=3 - [PRS6A_HUMAN] | 6 | 5 | 7 |
| P26368 | Splicing factor U2AF 65 kDa subunit OS=Homo sapiens GN=U2AF2 PE=1 SV=4 - [U2AF2_HUMAN] | 6 | 5 | 7 |
| P54727 | UV excision repair protein RAD23 homolog B OS=Homo sapiens GN=RAD23B PE=1 SV=1 - [RD23B_HUMAN] | 5 | 5 | 7 |
| Q14103 | Heterogeneous nuclear ribonucleoprotein D0 OS=Homo sapiens GN=HNRNPD PE=1 SV=1 - [HNRPD_HUMAN] | 4 | 5 | 7 |
| P0C0S5 | Histone H2A.Z OS=Homo sapiens GN=H2AFZ PE=1 SV=2 - [H2AZ_HUMAN] | 3 | 5 | 7 |
| P06899 | Histone H2B type 1-J OS=Homo sapiens GN=HIST1H2BJ PE=1 SV=3 - [H2B1J_HUMAN] | 2 | 5 | 7 |
| P20340 | Ras-related protein Rab-6A OS=Homo sapiens GN=RAB6A PE=1 SV=3 - [RAB6A_HUMAN] | 2 | 5 | 7 |
| Q14247 | Src substrate cortactin OS=Homo sapiens GN=CTTN PE=1 SV=2 - [SRC8_HUMAN] | 8 | 5 | 6 |
| Q8TCJ2 | Dolichyl-diphosphooligosaccharide--protein glycosyltransferase subunit STT3B OS=Homo sapiens GN=STT3B PE=1 SV=1 - [STT3B_HUMAN] | 8 | 5 | 6 |
| Q15155 | Nodal modulator 1 OS=Homo sapiens GN=NOMO1 PE=1 SV=5 - [NOMO1_HUMAN] | 8 | 5 | 6 |
| P56192 | Methionyl-tRNA synthetase. cytoplasmic OS=Homo sapiens GN=MARS PE=1 SV=2 - [SYMC_HUMAN] | 7 | 5 | 6 |
| Q13148 | TAR DNA-binding protein 43 OS=Homo sapiens GN=TARDBP PE=1 SV=1 - [TADBP_HUMAN] | 6 | 5 | 6 |
| P61163 | Alpha-centractin OS=Homo sapiens GN=ACTR1A PE=1 SV=1 - [ACTZ_HUMAN] | 6 | 5 | 6 |
| Q7Z434 | Mitochondrial antiviral-signaling protein OS=Homo sapiens GN=MAVS PE=1 SV=2 - [MAVS_HUMAN] | 6 | 5 | 6 |
| P49720 | Proteasome subunit beta type-3 OS=Homo sapiens GN=PSMB3 PE=1 SV=2 - [PSB3_HUMAN] | 5 | 5 | 6 |
| Q96QV6 | Histone H2A type 1-A OS=Homo sapiens GN=HIST1H2AA PE=1 SV=3 - [H2A1A_HUMAN] | 2 | 5 | 6 |
| P41252 | Isoleucyl-tRNA synthetase. cytoplasmic OS=Homo sapiens GN=IARS PE=1 SV=2 - [SYIC_HUMAN] | 7 | 5 | 5 |
| P54819 | Adenylate kinase 2. mitochondrial OS=Homo sapiens GN=AK2 PE=1 SV=2 - [KAD2_HUMAN] | 6 | 5 | 5 |
| O75821 | Eukaryotic translation initiation factor 3 subunit G OS=Homo sapiens GN=EIF3G PE=1 SV=2 - [EIF3G_HUMAN] | 6 | 5 | 5 |
| P48556 | 26S proteasome non-ATPase regulatory subunit 8 OS=Homo sapiens GN=PSMD8 PE=1 SV=2 - [PSMD8_HUMAN] | 6 | 5 | 5 |
| Q09161 | Nuclear cap-binding protein subunit 1 OS=Homo sapiens GN=NCBP1 PE=1 SV=1 - [NCBP1_HUMAN] | 6 | 5 | 5 |
| O15371 | Eukaryotic translation initiation factor 3 subunit D OS=Homo sapiens GN=EIF3D PE=1 SV=1 - [EIF3D_HUMAN] | 6 | 5 | 5 |
| P59998 | Actin-related protein 2/3 complex subunit 4 OS=Homo sapiens GN=ARPC4 PE=1 SV=3 - [ARPC4_HUMAN] | 5 | 5 | 5 |
| Q14165 | Malectin OS=Homo sapiens GN=MLEC PE=1 SV=1 - [MLEC_HUMAN] | 5 | 5 | 5 |
| Q07021 | Complement component 1 Q subcomponent-binding protein. mitochondrial OS=Homo sapiens GN=C1QBP PE=1 SV=1 - [C1QBP_HUMAN] | 5 | 5 | 5 |
| P61313 | 60S ribosomal protein L15 OS=Homo sapiens GN=RPL15 PE=1 SV=2 - [RL15_HUMAN] | 5 | 5 | 5 |
| Q8WXF1 | Paraspeckle component 1 OS=Homo sapiens GN=PSPC1 PE=1 SV=1 - [PSPC1_HUMAN] | 5 | 5 | 5 |
| P11279 | Lysosome-associated membrane glycoprotein 1 OS=Homo sapiens GN=LAMP1 PE=1 SV=3 - [LAMP1_HUMAN] | 5 | 5 | 5 |
| O60841 | Eukaryotic translation initiation factor 5B OS=Homo sapiens GN=EIF5B PE=1 SV=4 - [IF2P_HUMAN] | 5 | 5 | 5 |
| P62191 | 26S protease regulatory subunit 4 OS=Homo sapiens GN=PSMC1 PE=1 SV=1 - [PRS4_HUMAN] | 4 | 5 | 5 |
| Q13409 | Cytoplasmic dynein 1 intermediate chain 2 OS=Homo sapiens GN=DYNC1I2 PE=1 SV=3 - [DC1I2_HUMAN] | 4 | 5 | 5 |
| P16989 | DNA-binding protein A OS=Homo sapiens GN=CSDA PE=1 SV=4 - [DBPA_HUMAN] | 2 | 5 | 5 |
| Q9Y4W6 | AFG3-like protein 2 OS=Homo sapiens GN=AFG3L2 PE=1 SV=2 - [AFG32_HUMAN] | 7 | 5 | 4 |
| Q9NVI7 | ATPase family AAA domain-containing protein 3A OS=Homo sapiens GN=ATAD3A PE=1 SV=2 - [ATD3A_HUMAN] | 6 | 5 | 4 |
| P62280 | 40S ribosomal protein S11 OS=Homo sapiens GN=RPS11 PE=1 SV=3 - [RS11_HUMAN] | 5 | 5 | 4 |
| P13861 | cAMP-dependent protein kinase type II-alpha regulatory subunit OS=Homo sapiens GN=PRKAR2A PE=1 SV=2 - [KAP2_HUMAN] | 5 | 5 | 4 |
| O75436 | Vacuolar protein sorting-associated protein 26A OS=Homo sapiens GN=VPS26A PE=1 SV=2 - [VP26A_HUMAN] | 5 | 5 | 4 |
| Q9NUU7 | ATP-dependent RNA helicase DDX19A OS=Homo sapiens GN=DDX19A PE=1 SV=1 - [DD19A_HUMAN] | 5 | 5 | 4 |
| P62888 | 60S ribosomal protein L30 OS=Homo sapiens GN=RPL30 PE=1 SV=2 - [RL30_HUMAN] | 4 | 5 | 4 |
| Q14108 | Lysosome membrane protein 2 OS=Homo sapiens GN=SCARB2 PE=1 SV=2 - [SCRB2_HUMAN] | 4 | 5 | 4 |
| Q13765 | Nascent polypeptide-associated complex subunit alpha OS=Homo sapiens GN=NACA PE=1 SV=1 - [NACA_HUMAN] | 3 | 5 | 4 |
| O95782 | AP-2 complex subunit alpha-1 OS=Homo sapiens GN=AP2A1 PE=1 SV=3 - [AP2A1_HUMAN] | 3 | 5 | 4 |
| P14174 | Macrophage migration inhibitory factor OS=Homo sapiens GN=MIF PE=1 SV=4 - [MIF_HUMAN] | 2 | 5 | 4 |
| P20042 | Eukaryotic translation initiation factor 2 subunit 2 OS=Homo sapiens GN=EIF2S2 PE=1 SV=2 - [IF2B_HUMAN] | 6 | 5 | 3 |
| Q96HE7 | ERO1-like protein alpha OS=Homo sapiens GN=ERO1L PE=1 SV=2 - [ERO1A_HUMAN] | 6 | 5 | 3 |
| P62316 | Small nuclear ribonucleoprotein Sm D2 OS=Homo sapiens GN=SNRPD2 PE=1 SV=1 - [SMD2_HUMAN] | 5 | 5 | 3 |
| O43399 | Tumor protein D54 OS=Homo sapiens GN=TPD52L2 PE=1 SV=2 - [TPD54_HUMAN] | 5 | 5 | 3 |
| Q9UKK9 | ADP-sugar pyrophosphatase OS=Homo sapiens GN=NUDT5 PE=1 SV=1 - [NUDT5_HUMAN] | 5 | 5 | 3 |
| P68036 | Ubiquitin-conjugating enzyme E2 L3 OS=Homo sapiens GN=UBE2L3 PE=1 SV=1 - [UB2L3_HUMAN] | 4 | 5 | 3 |
| Q99439 | Calponin-2 OS=Homo sapiens GN=CNN2 PE=1 SV=4 - [CNN2_HUMAN] | 4 | 5 | 3 |
| Q9NVD7 | Alpha-parvin OS=Homo sapiens GN=PARVA PE=1 SV=1 - [PARVA_HUMAN] | 4 | 5 | 3 |
| Q15427 | Splicing factor 3B subunit 4 OS=Homo sapiens GN=SF3B4 PE=1 SV=1 - [SF3B4_HUMAN] | 4 | 5 | 3 |
| Q9NR31 | GTP-binding protein SAR1a OS=Homo sapiens GN=SAR1A PE=1 SV=1 - [SAR1A_HUMAN] | 2 | 5 | 3 |
| Q13283 | Ras GTPase-activating protein-binding protein 1 OS=Homo sapiens GN=G3BP1 PE=1 SV=1 - [G3BP1_HUMAN] | 5 | 5 | 2 |
| Q96CV9 | Optineurin OS=Homo sapiens GN=OPTN PE=1 SV=1 - [OPTN_HUMAN] | 4 | 5 | 2 |
| Q9NUQ9 | Protein FAM49B OS=Homo sapiens GN=FAM49B PE=1 SV=1 - [FA49B_HUMAN] | 5 | 5 | 1 |
| O76021 | Ribosomal L1 domain-containing protein 1 OS=Homo sapiens GN=RSL1D1 PE=1 SV=3 - [RL1D1_HUMAN] | 5 | 5 | 1 |
| P42892 | Endothelin-converting enzyme 1 OS=Homo sapiens GN=ECE1 PE=1 SV=2 - [ECE1_HUMAN] | 5 | 5 | 1 |
| Q9Y263 | Phospholipase A-2-activating protein OS=Homo sapiens GN=PLAA PE=1 SV=2 - [PLAP_HUMAN] | 5 | 5 | 1 |
| Q13596 | Sorting nexin-1 OS=Homo sapiens GN=SNX1 PE=1 SV=3 - [SNX1_HUMAN] | 3 | 5 | 1 |
| Q9UL42 | Paraneoplastic antigen Ma2 OS=Homo sapiens GN=PNMA2 PE=1 SV=2 - [PNMA2_HUMAN] | 5 | 5 | 0 |
| P53004 | Biliverdin reductase A OS=Homo sapiens GN=BLVRA PE=1 SV=2 - [BIEA_HUMAN] | 5 | 5 | 0 |
| Q96RS6 | NudC domain-containing protein 1 OS=Homo sapiens GN=NUDCD1 PE=1 SV=2 - [NUDC1_HUMAN] | 5 | 5 | 0 |
| Q7Z2K6 | Endoplasmic reticulum metallopeptidase 1 OS=Homo sapiens GN=ERMP1 PE=1 SV=2 - [ERMP1_HUMAN] | 5 | 5 | 0 |
| Q9UNN8 | Endothelial protein C receptor OS=Homo sapiens GN=PROCR PE=1 SV=1 - [EPCR_HUMAN] | 4 | 5 | 0 |
| P49915 | GMP synthase [glutamine-hydrolyzing] OS=Homo sapiens GN=GMPS PE=1 SV=1 - [GUAA_HUMAN] | 4 | 5 | 0 |
| Q9NY33 | Dipeptidyl peptidase 3 OS=Homo sapiens GN=DPP3 PE=1 SV=2 - [DPP3_HUMAN] | 21 | 4 | 28 |
| P13674 | Prolyl 4-hydroxylase subunit alpha-1 OS=Homo sapiens GN=P4HA1 PE=1 SV=2 - [P4HA1_HUMAN] | 15 | 4 | 19 |
| P20020 | Plasma membrane calcium-transporting ATPase 1 OS=Homo sapiens OX=9606 GN=ATP2B1 PE=1 SV=4 - [AT2B1_HUMAN] | 12 | 4 | 14 |
| P04040 | Catalase OS=Homo sapiens GN=CAT PE=1 SV=3 - [CATA_HUMAN] | 12 | 4 | 13 |
| P28838 | Cytosol aminopeptidase OS=Homo sapiens GN=LAP3 PE=1 SV=3 - [AMPL_HUMAN] | 12 | 4 | 13 |
| O60763 | General vesicular transport factor p115 OS=Homo sapiens GN=USO1 PE=1 SV=2 - [USO1_HUMAN] | 12 | 4 | 13 |
| P22392 | Nucleoside diphosphate kinase B OS=Homo sapiens GN=NME2 PE=1 SV=1 - [NDKB_HUMAN] | 2 | 4 | 12 |
| Q9BPW8 | Protein NipSnap homolog 1 OS=Homo sapiens GN=NIPSNAP1 PE=1 SV=1 - [NIPS1_HUMAN] | 9 | 4 | 11 |
| P47756 | F-actin-capping protein subunit beta OS=Homo sapiens GN=CAPZB PE=1 SV=4 - [CAPZB_HUMAN] | 7 | 4 | 11 |
| Q9BR76 | Coronin-1B OS=Homo sapiens GN=CORO1B PE=1 SV=1 - [COR1B_HUMAN] | 9 | 4 | 10 |
| Q16401 | 26S proteasome non-ATPase regulatory subunit 5 OS=Homo sapiens GN=PSMD5 PE=1 SV=3 - [PSMD5_HUMAN] | 8 | 4 | 10 |
| P15531 | Nucleoside diphosphate kinase A OS=Homo sapiens GN=NME1 PE=1 SV=1 - [NDKA_HUMAN] | 2 | 4 | 10 |
| Q99798 | Aconitate hydratase. mitochondrial OS=Homo sapiens GN=ACO2 PE=1 SV=2 - [ACON_HUMAN] | 10 | 4 | 9 |
| O14880 | Microsomal glutathione S-transferase 3 OS=Homo sapiens GN=MGST3 PE=1 SV=1 - [MGST3_HUMAN] | 5 | 4 | 9 |
| P31947 | 14-3-3 protein sigma OS=Homo sapiens GN=SFN PE=1 SV=1 - [1433S_HUMAN] | 4 | 4 | 9 |
| P22695 | Cytochrome b-c1 complex subunit 2. mitochondrial OS=Homo sapiens GN=UQCRC2 PE=1 SV=3 - [QCR2_HUMAN] | 8 | 4 | 8 |
| O00116 | Alkyldihydroxyacetonephosphate synthase. peroxisomal OS=Homo sapiens GN=AGPS PE=1 SV=1 - [ADAS_HUMAN] | 8 | 4 | 8 |
| Q16822 | Phosphoenolpyruvate carboxykinase [GTP]. mitochondrial OS=Homo sapiens OX=9606 GN=PCK2 PE=1 SV=4 - [PCKGM_HUMAN] | 8 | 4 | 8 |
| P33991 | DNA replication licensing factor MCM4 OS=Homo sapiens GN=MCM4 PE=1 SV=5 - [MCM4_HUMAN] | 8 | 4 | 8 |
| P27144 | Adenylate kinase isoenzyme 4. mitochondrial OS=Homo sapiens GN=AK3L1 PE=1 SV=1 - [KAD4_HUMAN] | 7 | 4 | 8 |
| O95865 | N(G).N(G)-dimethylarginine dimethylaminohydrolase 2 OS=Homo sapiens GN=DDAH2 PE=1 SV=1 - [DDAH2_HUMAN] | 7 | 4 | 8 |
| P36542 | ATP synthase subunit gamma. mitochondrial OS=Homo sapiens GN=ATP5C1 PE=1 SV=1 - [ATPG_HUMAN] | 7 | 4 | 8 |
| P24752 | Acetyl-CoA acetyltransferase. mitochondrial OS=Homo sapiens GN=ACAT1 PE=1 SV=1 - [THIL_HUMAN] | 7 | 4 | 8 |
| O00232 | 26S proteasome non-ATPase regulatory subunit 12 OS=Homo sapiens GN=PSMD12 PE=1 SV=3 - [PSD12_HUMAN] | 7 | 4 | 8 |
| P61088 | Ubiquitin-conjugating enzyme E2 N OS=Homo sapiens GN=UBE2N PE=1 SV=1 - [UBE2N_HUMAN] | 6 | 4 | 8 |
| P27338 | Amine oxidase [flavin-containing] B OS=Homo sapiens GN=MAOB PE=1 SV=3 - [AOFB_HUMAN] | 6 | 4 | 8 |
| Q9Y6C9 | Mitochondrial carrier homolog 2 OS=Homo sapiens GN=MTCH2 PE=1 SV=1 - [MTCH2_HUMAN] | 5 | 4 | 8 |
| P25398 | 40S ribosomal protein S12 OS=Homo sapiens GN=RPS12 PE=1 SV=3 - [RS12_HUMAN] | 3 | 4 | 8 |
| P35611 | Alpha-adducin OS=Homo sapiens GN=ADD1 PE=1 SV=2 - [ADDA_HUMAN] | 8 | 4 | 7 |
| O76094 | Signal recognition particle 72 kDa protein OS=Homo sapiens GN=SRP72 PE=1 SV=3 - [SRP72_HUMAN] | 7 | 4 | 7 |
| P61604 | 10 kDa heat shock protein. mitochondrial OS=Homo sapiens GN=HSPE1 PE=1 SV=2 - [CH10_HUMAN] | 6 | 4 | 7 |
| P27105 | Erythrocyte band 7 integral membrane protein OS=Homo sapiens GN=STOM PE=1 SV=3 - [STOM_HUMAN] | 6 | 4 | 7 |
| P12004 | Proliferating cell nuclear antigen OS=Homo sapiens GN=PCNA PE=1 SV=1 - [PCNA_HUMAN] | 6 | 4 | 7 |
| P54619 | 5'-AMP-activated protein kinase subunit gamma-1 OS=Homo sapiens GN=PRKAG1 PE=1 SV=1 - [AAKG1_HUMAN] | 5 | 4 | 7 |
| P61106 | Ras-related protein Rab-14 OS=Homo sapiens GN=RAB14 PE=1 SV=4 - [RAB14_HUMAN] | 4 | 4 | 7 |
| P08708 | 40S ribosomal protein S17 OS=Homo sapiens GN=RPS17 PE=1 SV=2 - [RS17_HUMAN] | 3 | 4 | 7 |
| O60814 | Histone H2B type 1-K OS=Homo sapiens GN=HIST1H2BK PE=1 SV=3 - [H2B1K_HUMAN] | 2 | 4 | 7 |
| Q07960 | Rho GTPase-activating protein 1 OS=Homo sapiens GN=ARHGAP1 PE=1 SV=1 - [RHG01_HUMAN] | 7 | 4 | 6 |
| Q13057 | Bifunctional coenzyme A synthase OS=Homo sapiens GN=COASY PE=1 SV=4 - [COASY_HUMAN] | 7 | 4 | 6 |
| P61221 | ATP-binding cassette sub-family E member 1 OS=Homo sapiens GN=ABCE1 PE=1 SV=1 - [ABCE1_HUMAN] | 7 | 4 | 6 |
| P23588 | Eukaryotic translation initiation factor 4B OS=Homo sapiens GN=EIF4B PE=1 SV=2 - [IF4B_HUMAN] | 7 | 4 | 6 |
| O15067 | Phosphoribosylformylglycinamidine synthase OS=Homo sapiens GN=PFAS PE=1 SV=4 - [PUR4_HUMAN] | 7 | 4 | 6 |
| P60891 | Ribose-phosphate pyrophosphokinase 1 OS=Homo sapiens GN=PRPS1 PE=1 SV=2 - [PRPS1_HUMAN] | 5 | 4 | 6 |
| Q15942 | Zyxin OS=Homo sapiens GN=ZYX PE=1 SV=1 - [ZYX_HUMAN] | 5 | 4 | 6 |
| O95573 | Long-chain-fatty-acid--CoA ligase 3 OS=Homo sapiens GN=ACSL3 PE=1 SV=3 - [ACSL3_HUMAN] | 5 | 4 | 6 |
| Q9P0L0 | Vesicle-associated membrane protein-associated protein A OS=Homo sapiens GN=VAPA PE=1 SV=3 - [VAPA_HUMAN] | 4 | 4 | 6 |
| P40429 | 60S ribosomal protein L13a OS=Homo sapiens GN=RPL13A PE=1 SV=2 - [RL13A_HUMAN] | 4 | 4 | 6 |
| Q8NC51 | Plasminogen activator inhibitor 1 RNA-binding protein OS=Homo sapiens GN=SERBP1 PE=1 SV=2 - [PAIRB_HUMAN] | 4 | 4 | 6 |
| Q9UBF2 | Coatomer subunit gamma-2 OS=Homo sapiens GN=COPG2 PE=1 SV=1 - [COPG2_HUMAN] | 2 | 4 | 6 |
| O60884 | DnaJ homolog subfamily A member 2 OS=Homo sapiens GN=DNAJA2 PE=1 SV=1 - [DNJA2_HUMAN] | 6 | 4 | 5 |
| P07954 | Fumarate hydratase. mitochondrial OS=Homo sapiens GN=FH PE=1 SV=3 - [FUMH_HUMAN] | 6 | 4 | 5 |
| O95747 | Serine/threonine-protein kinase OSR1 OS=Homo sapiens GN=OXSR1 PE=1 SV=1 - [OXSR1_HUMAN] | 6 | 4 | 5 |
| Q15392 | 24-dehydrocholesterol reductase OS=Homo sapiens GN=DHCR24 PE=1 SV=2 - [DHC24_HUMAN] | 6 | 4 | 5 |
| Q15056 | Eukaryotic translation initiation factor 4H OS=Homo sapiens GN=EIF4H PE=1 SV=5 - [IF4H_HUMAN] | 5 | 4 | 5 |
| P35270 | Sepiapterin reductase OS=Homo sapiens GN=SPR PE=1 SV=1 - [SPRE_HUMAN] | 5 | 4 | 5 |
| Q04760 | Lactoylglutathione lyase OS=Homo sapiens GN=GLO1 PE=1 SV=4 - [LGUL_HUMAN] | 5 | 4 | 5 |
| Q99729 | Heterogeneous nuclear ribonucleoprotein A/B OS=Homo sapiens GN=HNRNPAB PE=1 SV=2 - [ROAA_HUMAN] | 5 | 4 | 5 |
| Q14444 | Caprin-1 OS=Homo sapiens GN=CAPRIN1 PE=1 SV=2 - [CAPR1_HUMAN] | 5 | 4 | 5 |
| O95202 | LETM1 and EF-hand domain-containing protein 1. mitochondrial OS=Homo sapiens GN=LETM1 PE=1 SV=1 - [LETM1_HUMAN] | 5 | 4 | 5 |
| Q9H2U2 | Inorganic pyrophosphatase 2. mitochondrial OS=Homo sapiens GN=PPA2 PE=1 SV=2 - [IPYR2_HUMAN] | 4 | 4 | 5 |
| P43034 | Platelet-activating factor acetylhydrolase IB subunit alpha OS=Homo sapiens GN=PAFAH1B1 PE=1 SV=2 - [LIS1_HUMAN] | 4 | 4 | 5 |
| Q07666 | KH domain-containing. RNA-binding. signal transduction-associated protein 1 OS=Homo sapiens GN=KHDRBS1 PE=1 SV=1 - [KHDR1_HUMAN] | 4 | 4 | 5 |
| P62913 | 60S ribosomal protein L11 OS=Homo sapiens GN=RPL11 PE=1 SV=2 - [RL11_HUMAN] | 3 | 4 | 5 |
| P02765 | Alpha-2-HS-glycoprotein OS=Homo sapiens OX=9606 GN=AHSG PE=1 SV=2 - [FETUA_HUMAN] | 3 | 4 | 5 |
| Q9UIA9 | Exportin-7 OS=Homo sapiens GN=XPO7 PE=1 SV=3 - [XPO7_HUMAN] | 7 | 4 | 4 |
| Q9Y2Z0 | Suppressor of G2 allele of SKP1 homolog OS=Homo sapiens GN=SUGT1 PE=1 SV=3 - [SUGT1_HUMAN] | 6 | 4 | 4 |
| Q9NVJ2 | ADP-ribosylation factor-like protein 8B OS=Homo sapiens GN=ARL8B PE=1 SV=1 - [ARL8B_HUMAN] | 5 | 4 | 4 |
| P62750 | 60S ribosomal protein L23a OS=Homo sapiens GN=RPL23A PE=1 SV=1 - [RL23A_HUMAN] | 5 | 4 | 4 |
| P18754 | Regulator of chromosome condensation OS=Homo sapiens GN=RCC1 PE=1 SV=1 - [RCC1_HUMAN] | 5 | 4 | 4 |
| O94925 | Glutaminase kidney isoform. mitochondrial OS=Homo sapiens GN=GLS PE=1 SV=1 - [GLSK_HUMAN] | 5 | 4 | 4 |
| Q9HCE1 | Putative helicase MOV-10 OS=Homo sapiens GN=MOV10 PE=1 SV=2 - [MOV10_HUMAN] | 5 | 4 | 4 |
| Q9BRA2 | Thioredoxin domain-containing protein 17 OS=Homo sapiens GN=TXNDC17 PE=1 SV=1 - [TXD17_HUMAN] | 4 | 4 | 4 |
| P60660 | Myosin light polypeptide 6 OS=Homo sapiens GN=MYL6 PE=1 SV=2 - [MYL6_HUMAN] | 4 | 4 | 4 |
| P39019 | 40S ribosomal protein S19 OS=Homo sapiens GN=RPS19 PE=1 SV=2 - [RS19_HUMAN] | 4 | 4 | 4 |
| P47755 | F-actin-capping protein subunit alpha-2 OS=Homo sapiens GN=CAPZA2 PE=1 SV=3 - [CAZA2_HUMAN] | 4 | 4 | 4 |
| P50914 | 60S ribosomal protein L14 OS=Homo sapiens GN=RPL14 PE=1 SV=4 - [RL14_HUMAN] | 4 | 4 | 4 |
| P61160 | Actin-related protein 2 OS=Homo sapiens GN=ACTR2 PE=1 SV=1 - [ARP2_HUMAN] | 4 | 4 | 4 |
| Q99733 | Nucleosome assembly protein 1-like 4 OS=Homo sapiens GN=NAP1L4 PE=1 SV=1 - [NP1L4_HUMAN] | 4 | 4 | 4 |
| Q9H3U1 | Protein unc-45 homolog A OS=Homo sapiens GN=UNC45A PE=1 SV=1 - [UN45A_HUMAN] | 4 | 4 | 4 |
| P11586 | C-1-tetrahydrofolate synthase. cytoplasmic OS=Homo sapiens GN=MTHFD1 PE=1 SV=3 - [C1TC_HUMAN] | 4 | 4 | 4 |
| Q9NQW7 | Xaa-Pro aminopeptidase 1 OS=Homo sapiens GN=XPNPEP1 PE=1 SV=3 - [XPP1_HUMAN] | 6 | 4 | 3 |
| Q9Y5B9 | FACT complex subunit SPT16 OS=Homo sapiens GN=SUPT16H PE=1 SV=1 - [SP16H_HUMAN] | 6 | 4 | 3 |
| Q9UHB9 | Signal recognition particle 68 kDa protein OS=Homo sapiens GN=SRP68 PE=1 SV=2 - [SRP68_HUMAN] | 5 | 4 | 3 |
| P12931 | Proto-oncogene tyrosine-protein kinase Src OS=Homo sapiens GN=SRC PE=1 SV=3 - [SRC_HUMAN] | 5 | 4 | 3 |
| P50570 | Dynamin-2 OS=Homo sapiens GN=DNM2 PE=1 SV=2 - [DYN2_HUMAN] | 5 | 4 | 3 |
| Q13435 | Splicing factor 3B subunit 2 OS=Homo sapiens GN=SF3B2 PE=1 SV=2 - [SF3B2_HUMAN] | 5 | 4 | 3 |
| Q13045 | Protein flightless-1 homolog OS=Homo sapiens GN=FLII PE=1 SV=2 - [FLII_HUMAN] | 5 | 4 | 3 |
| P61803 | Dolichyl-diphosphooligosaccharide--protein glycosyltransferase subunit DAD1 OS=Homo sapiens GN=DAD1 PE=1 SV=3 - [DAD1_HUMAN] | 4 | 4 | 3 |
| Q9NRV9 | Heme-binding protein 1 OS=Homo sapiens GN=HEBP1 PE=1 SV=1 - [HEBP1_HUMAN] | 4 | 4 | 3 |
| P16401 | Histone H1.5 OS=Homo sapiens GN=HIST1H1B PE=1 SV=3 - [H15_HUMAN] | 4 | 4 | 3 |
| Q6ZN17 | Protein lin-28 homolog B OS=Homo sapiens GN=LIN28B PE=1 SV=1 - [LN28B_HUMAN] | 4 | 4 | 3 |
| Q9NZT2 | Opioid growth factor receptor OS=Homo sapiens GN=OGFR PE=1 SV=3 - [OGFR_HUMAN] | 4 | 4 | 3 |
| Q16643 | Drebrin OS=Homo sapiens GN=DBN1 PE=1 SV=4 - [DREB_HUMAN] | 4 | 4 | 3 |
| Q9BS26 | Endoplasmic reticulum resident protein 44 OS=Homo sapiens GN=ERP44 PE=1 SV=1 - [ERP44_HUMAN] | 4 | 4 | 3 |
| Q7L014 | Probable ATP-dependent RNA helicase DDX46 OS=Homo sapiens GN=DDX46 PE=1 SV=2 - [DDX46_HUMAN] | 4 | 4 | 3 |
| P00441 | Superoxide dismutase [Cu-Zn] OS=Homo sapiens GN=SOD1 PE=1 SV=2 - [SODC_HUMAN] | 3 | 4 | 3 |
| P60866 | 40S ribosomal protein S20 OS=Homo sapiens GN=RPS20 PE=1 SV=1 - [RS20_HUMAN] | 3 | 4 | 3 |
| P02792 | Ferritin light chain OS=Homo sapiens GN=FTL PE=1 SV=2 - [FRIL_HUMAN] | 3 | 4 | 3 |
| P31942 | Heterogeneous nuclear ribonucleoprotein H3 OS=Homo sapiens GN=HNRNPH3 PE=1 SV=2 - [HNRH3_HUMAN] | 3 | 4 | 3 |
| P21291 | Cysteine and glycine-rich protein 1 OS=Homo sapiens GN=CSRP1 PE=1 SV=3 - [CSRP1_HUMAN] | 5 | 4 | 2 |
| P54920 | Alpha-soluble NSF attachment protein OS=Homo sapiens GN=NAPA PE=1 SV=3 - [SNAA_HUMAN] | 5 | 4 | 2 |
| P27635 | 60S ribosomal protein L10 OS=Homo sapiens GN=RPL10 PE=1 SV=4 - [RL10_HUMAN] | 5 | 4 | 2 |
| Q6DKJ4 | Nucleoredoxin OS=Homo sapiens GN=NXN PE=1 SV=2 - [NXN_HUMAN] | 5 | 4 | 2 |
| P49189 | 4-trimethylaminobutyraldehyde dehydrogenase OS=Homo sapiens GN=ALDH9A1 PE=1 SV=3 - [AL9A1_HUMAN] | 5 | 4 | 2 |
| Q08J23 | tRNA (cytosine-5-)-methyltransferase NSUN2 OS=Homo sapiens GN=NSUN2 PE=1 SV=2 - [NSUN2_HUMAN] | 5 | 4 | 2 |
| Q6XQN6 | Nicotinate phosphoribosyltransferase OS=Homo sapiens GN=NAPRT1 PE=1 SV=2 - [PNCB_HUMAN] | 5 | 4 | 2 |
| P62244 | 40S ribosomal protein S15a OS=Homo sapiens GN=RPS15A PE=1 SV=2 - [RS15A_HUMAN] | 4 | 4 | 2 |
| O15173 | Membrane-associated progesterone receptor component 2 OS=Homo sapiens GN=PGRMC2 PE=1 SV=1 - [PGRC2_HUMAN] | 4 | 4 | 2 |
| Q96IJ6 | Mannose-1-phosphate guanyltransferase alpha OS=Homo sapiens GN=GMPPA PE=1 SV=1 - [GMPPA_HUMAN] | 4 | 4 | 2 |
| Q9H3P7 | Golgi resident protein GCP60 OS=Homo sapiens GN=ACBD3 PE=1 SV=4 - [GCP60_HUMAN] | 4 | 4 | 2 |
| Q9UNF1 | Melanoma-associated antigen D2 OS=Homo sapiens GN=MAGED2 PE=1 SV=2 - [MAGD2_HUMAN] | 4 | 4 | 2 |
| A6NGU5 | Putative gamma-glutamyltranspeptidase 3 OS=Homo sapiens GN=GGT3P PE=5 SV=2 - [GGT3_HUMAN] | 4 | 4 | 2 |
| Q10471 | Polypeptide N-acetylgalactosaminyltransferase 2 OS=Homo sapiens GN=GALNT2 PE=1 SV=1 - [GALT2_HUMAN] | 4 | 4 | 2 |
| Q9NTJ5 | Phosphatidylinositide phosphatase SAC1 OS=Homo sapiens GN=SACM1L PE=1 SV=2 - [SAC1_HUMAN] | 4 | 4 | 2 |
| Q9BUJ2 | Heterogeneous nuclear ribonucleoprotein U-like protein 1 OS=Homo sapiens GN=HNRNPUL1 PE=1 SV=2 - [HNRL1_HUMAN] | 4 | 4 | 2 |
| Q9Y5K6 | CD2-associated protein OS=Homo sapiens GN=CD2AP PE=1 SV=1 - [CD2AP_HUMAN] | 4 | 4 | 2 |
| Q12996 | Cleavage stimulation factor subunit 3 OS=Homo sapiens GN=CSTF3 PE=1 SV=1 - [CSTF3_HUMAN] | 4 | 4 | 2 |
| Q15121 | Astrocytic phosphoprotein PEA-15 OS=Homo sapiens GN=PEA15 PE=1 SV=2 - [PEA15_HUMAN] | 4 | 4 | 1 |
| O15400 | Syntaxin-7 OS=Homo sapiens GN=STX7 PE=1 SV=4 - [STX7_HUMAN] | 4 | 4 | 1 |
| Q9UIJ7 | GTP:AMP phosphotransferase mitochondrial OS=Homo sapiens GN=AK3 PE=1 SV=4 - [KAD3_HUMAN] | 4 | 4 | 1 |
| P55036 | 26S proteasome non-ATPase regulatory subunit 4 OS=Homo sapiens GN=PSMD4 PE=1 SV=1 - [PSMD4_HUMAN] | 4 | 4 | 1 |
| Q5JTV8 | Torsin-1A-interacting protein 1 OS=Homo sapiens GN=TOR1AIP1 PE=1 SV=2 - [TOIP1_HUMAN] | 4 | 4 | 1 |
| P15170 | Eukaryotic peptide chain release factor GTP-binding subunit ERF3A OS=Homo sapiens GN=GSPT1 PE=1 SV=1 - [ERF3A_HUMAN] | 4 | 4 | 1 |
| P09960 | Leukotriene A-4 hydrolase OS=Homo sapiens GN=LTA4H PE=1 SV=2 - [LKHA4_HUMAN] | 4 | 4 | 1 |
| P13798 | Acylamino-acid-releasing enzyme OS=Homo sapiens GN=APEH PE=1 SV=4 - [ACPH_HUMAN] | 4 | 4 | 1 |
| Q86UP2 | Kinectin OS=Homo sapiens GN=KTN1 PE=1 SV=1 - [KTN1_HUMAN] | 4 | 4 | 1 |
| P31949 | Protein S100-A11 OS=Homo sapiens GN=S100A11 PE=1 SV=2 - [S10AB_HUMAN] | 3 | 4 | 1 |
| Q8N8S7 | Protein enabled homolog OS=Homo sapiens GN=ENAH PE=1 SV=2 - [ENAH_HUMAN] | 3 | 4 | 1 |
| P08237 | 6-phosphofructokinase. muscle type OS=Homo sapiens GN=PFKM PE=1 SV=2 - [K6PF_HUMAN] | 3 | 4 | 1 |
| O75223 | Gamma-glutamylcyclotransferase OS=Homo sapiens GN=GGCT PE=1 SV=1 - [GGCT_HUMAN] | 4 | 4 | 0 |
| P11117 | Lysosomal acid phosphatase OS=Homo sapiens GN=ACP2 PE=1 SV=3 - [PPAL_HUMAN] | 4 | 4 | 0 |
| Q14738 | Serine/threonine-protein phosphatase 2A 56 kDa regulatory subunit delta isoform OS=Homo sapiens GN=PPP2R5D PE=1 SV=1 - [2A5D_HUMAN] | 4 | 4 | 0 |
| P08648 | Integrin alpha-5 OS=Homo sapiens GN=ITGA5 PE=1 SV=2 - [ITA5_HUMAN] | 4 | 4 | 0 |
| O14964 | Hepatocyte growth factor-regulated tyrosine kinase substrate OS=Homo sapiens GN=HGS PE=1 SV=1 - [HGS_HUMAN] | 4 | 4 | 0 |
| P55285 | Cadherin-6 OS=Homo sapiens GN=CDH6 PE=1 SV=1 - [CADH6_HUMAN] | 4 | 4 | 0 |
| Q9H993 | UPF0364 protein C6orf211 OS=Homo sapiens GN=C6orf211 PE=1 SV=1 - [CF211_HUMAN] | 3 | 4 | 0 |
| Q6NZI2 | Polymerase I and transcript release factor OS=Homo sapiens GN=PTRF PE=1 SV=1 - [PTRF_HUMAN] | 3 | 4 | 0 |
| Q9UJS0 | Calcium-binding mitochondrial carrier protein Aralar2 OS=Homo sapiens GN=SLC25A13 PE=1 SV=2 - [CMC2_HUMAN] | 12 | 3 | 14 |
| Q6DD88 | Atlastin-3 OS=Homo sapiens GN=ATL3 PE=1 SV=1 - [ATLA3_HUMAN] | 11 | 3 | 13 |
| O60488 | Long-chain-fatty-acid--CoA ligase 4 OS=Homo sapiens GN=ACSL4 PE=1 SV=2 - [ACSL4_HUMAN] | 9 | 3 | 12 |
| P61019 | Ras-related protein Rab-2A OS=Homo sapiens GN=RAB2A PE=1 SV=1 - [RAB2A_HUMAN] | 10 | 3 | 10 |
| Q12792 | Twinfilin-1 OS=Homo sapiens GN=TWF1 PE=1 SV=3 - [TWF1_HUMAN] | 9 | 3 | 10 |
| P78347 | General transcription factor II-I OS=Homo sapiens GN=GTF2I PE=1 SV=2 - [GTF2I_HUMAN] | 10 | 3 | 9 |
| P25789 | Proteasome subunit alpha type-4 OS=Homo sapiens GN=PSMA4 PE=1 SV=1 - [PSA4_HUMAN] | 8 | 3 | 9 |
| Q96G03 | Phosphoglucomutase-2 OS=Homo sapiens GN=PGM2 PE=1 SV=4 - [PGM2_HUMAN] | 8 | 3 | 9 |
| Q9NSE4 | Isoleucyl-tRNA synthetase. mitochondrial OS=Homo sapiens GN=IARS2 PE=1 SV=2 - [SYIM_HUMAN] | 8 | 3 | 9 |
| O43809 | Cleavage and polyadenylation specificity factor subunit 5 OS=Homo sapiens GN=NUDT21 PE=1 SV=1 - [CPSF5_HUMAN] | 6 | 3 | 9 |
| P68400 | Casein kinase II subunit alpha OS=Homo sapiens GN=CSNK2A1 PE=1 SV=1 - [CSK21_HUMAN] | 8 | 3 | 8 |
| Q9Y394 | Dehydrogenase/reductase SDR family member 7 OS=Homo sapiens GN=DHRS7 PE=1 SV=1 - [DHRS7_HUMAN] | 6 | 3 | 8 |
| P26196 | Probable ATP-dependent RNA helicase DDX6 OS=Homo sapiens GN=DDX6 PE=1 SV=2 - [DDX6_HUMAN] | 6 | 3 | 8 |
| P14923 | Junction plakoglobin OS=Homo sapiens GN=JUP PE=1 SV=3 - [PLAK_HUMAN] | 5 | 3 | 8 |
| O00629 | Importin subunit alpha-4 OS=Homo sapiens GN=KPNA4 PE=1 SV=1 - [IMA4_HUMAN] | 4 | 3 | 8 |
| Q9BXP5 | Serrate RNA effector molecule homolog OS=Homo sapiens GN=SRRT PE=1 SV=1 - [SRRT_HUMAN] | 7 | 3 | 7 |
| P62263 | 40S ribosomal protein S14 OS=Homo sapiens GN=RPS14 PE=1 SV=3 - [RS14_HUMAN] | 6 | 3 | 7 |
| Q5VYK3 | Proteasome-associated protein ECM29 homolog OS=Homo sapiens GN=ECM29 PE=1 SV=2 - [ECM29_HUMAN] | 6 | 3 | 7 |
| O95340 | Bifunctional 3'-phosphoadenosine 5'-phosphosulfate synthase 2 OS=Homo sapiens GN=PAPSS2 PE=1 SV=2 - [PAPS2_HUMAN] | 4 | 3 | 7 |
| P13796 | Plastin-2 OS=Homo sapiens GN=LCP1 PE=1 SV=6 - [PLSL_HUMAN] | 3 | 3 | 7 |
| Q96HR9 | Receptor expression-enhancing protein 6 OS=Homo sapiens OX=9606 GN=REEP6 PE=1 SV=2 - [REEP6_HUMAN] | 2 | 3 | 7 |
| P30419 | Glycylpeptide N-tetradecanoyltransferase 1 OS=Homo sapiens GN=NMT1 PE=1 SV=2 - [NMT1_HUMAN] | 8 | 3 | 6 |
| P35237 | Serpin B6 OS=Homo sapiens GN=SERPINB6 PE=1 SV=3 - [SPB6_HUMAN] | 6 | 3 | 6 |
| Q96CS3 | FAS-associated factor 2 OS=Homo sapiens GN=FAF2 PE=1 SV=2 - [FAF2_HUMAN] | 6 | 3 | 6 |
| P50453 | Serpin B9 OS=Homo sapiens GN=SERPINB9 PE=1 SV=1 - [SPB9_HUMAN] | 6 | 3 | 6 |
| P09622 | Dihydrolipoyl dehydrogenase. mitochondrial OS=Homo sapiens GN=DLD PE=1 SV=2 - [DLDH_HUMAN] | 6 | 3 | 6 |
| Q15185 | Prostaglandin E synthase 3 OS=Homo sapiens GN=PTGES3 PE=1 SV=1 - [TEBP_HUMAN] | 5 | 3 | 6 |
| Q13155 | Aminoacyl tRNA synthetase complex-interacting multifunctional protein 2 OS=Homo sapiens GN=AIMP2 PE=1 SV=2 - [AIMP2_HUMAN] | 5 | 3 | 6 |
| P61289 | Proteasome activator complex subunit 3 OS=Homo sapiens GN=PSME3 PE=1 SV=1 - [PSME3_HUMAN] | 5 | 3 | 6 |
| Q96HY6 | DDRGK domain-containing protein 1 OS=Homo sapiens GN=DDRGK1 PE=1 SV=2 - [DDRGK_HUMAN] | 5 | 3 | 6 |
| Q9UBE0 | SUMO-activating enzyme subunit 1 OS=Homo sapiens GN=SAE1 PE=1 SV=1 - [SAE1_HUMAN] | 5 | 3 | 6 |
| Q13247 | Splicing factor. arginine/serine-rich 6 OS=Homo sapiens GN=SFRS6 PE=1 SV=2 - [SFRS6_HUMAN] | 5 | 3 | 6 |
| P35613 | Basigin OS=Homo sapiens GN=BSG PE=1 SV=2 - [BASI_HUMAN] | 4 | 3 | 6 |
| Q16630 | Cleavage and polyadenylation specificity factor subunit 6 OS=Homo sapiens GN=CPSF6 PE=1 SV=2 - [CPSF6_HUMAN] | 4 | 3 | 6 |
| Q92820 | Gamma-glutamyl hydrolase OS=Homo sapiens GN=GGH PE=1 SV=2 - [GGH_HUMAN] | 4 | 3 | 6 |
| O14979 | Heterogeneous nuclear ribonucleoprotein D-like OS=Homo sapiens GN=HNRPDL PE=1 SV=3 - [HNRDL_HUMAN] | 4 | 3 | 6 |
| P17812 | CTP synthase 1 OS=Homo sapiens GN=CTPS PE=1 SV=2 - [PYRG1_HUMAN] | 4 | 3 | 6 |
| P05386 | 60S acidic ribosomal protein P1 OS=Homo sapiens GN=RPLP1 PE=1 SV=1 - [RLA1_HUMAN] | 3 | 3 | 6 |
| P39687 | Acidic leucine-rich nuclear phosphoprotein 32 family member A OS=Homo sapiens GN=ANP32A PE=1 SV=1 - [AN32A_HUMAN] | 3 | 3 | 6 |
| Q15459 | Splicing factor 3A subunit 1 OS=Homo sapiens GN=SF3A1 PE=1 SV=1 - [SF3A1_HUMAN] | 7 | 3 | 5 |
| Q9UHX1 | Poly(U)-binding-splicing factor PUF60 OS=Homo sapiens GN=PUF60 PE=1 SV=1 - [PUF60_HUMAN] | 6 | 3 | 5 |
| Q9GZS3 | WD repeat-containing protein 61 OS=Homo sapiens GN=WDR61 PE=1 SV=1 - [WDR61_HUMAN] | 5 | 3 | 5 |
| Q8TF05 | Serine/threonine-protein phosphatase 4 regulatory subunit 1 OS=Homo sapiens GN=PPP4R1 PE=1 SV=1 - [PP4R1_HUMAN] | 5 | 3 | 5 |
| P05387 | 60S acidic ribosomal protein P2 OS=Homo sapiens GN=RPLP2 PE=1 SV=1 - [RLA2_HUMAN] | 4 | 3 | 5 |
| P40616 | ADP-ribosylation factor-like protein 1 OS=Homo sapiens GN=ARL1 PE=1 SV=1 - [ARL1_HUMAN] | 4 | 3 | 5 |
| Q96A72 | Protein mago nashi homolog 2 OS=Homo sapiens GN=MAGOHB PE=1 SV=1 - [MGN2_HUMAN] | 4 | 3 | 5 |
| P02794 | Ferritin heavy chain OS=Homo sapiens GN=FTH1 PE=1 SV=2 - [FRIH_HUMAN] | 4 | 3 | 5 |
| O75844 | CAAX prenyl protease 1 homolog OS=Homo sapiens GN=ZMPSTE24 PE=1 SV=2 - [FACE1_HUMAN] | 4 | 3 | 5 |
| P11310 | Medium-chain specific acyl-CoA dehydrogenase. mitochondrial OS=Homo sapiens GN=ACADM PE=1 SV=1 - [ACADM_HUMAN] | 4 | 3 | 5 |
| P08621 | U1 small nuclear ribonucleoprotein 70 kDa OS=Homo sapiens GN=SNRNP70 PE=1 SV=2 - [RU17_HUMAN] | 4 | 3 | 5 |
| O60762 | Dolichol-phosphate mannosyltransferase OS=Homo sapiens GN=DPM1 PE=1 SV=1 - [DPM1_HUMAN] | 3 | 3 | 5 |
| Q9Y3E0 | Vesicle transport protein GOT1B OS=Homo sapiens GN=GOLT1B PE=1 SV=1 - [GOT1B_HUMAN] | 3 | 3 | 5 |
| P53007 | Tricarboxylate transport protein. mitochondrial OS=Homo sapiens GN=SLC25A1 PE=1 SV=2 - [TXTP_HUMAN] | 3 | 3 | 5 |
| P27361 | Mitogen-activated protein kinase 3 OS=Homo sapiens GN=MAPK3 PE=1 SV=4 - [MK03_HUMAN] | 2 | 3 | 5 |
| P49902 | Cytosolic purine 5'-nucleotidase OS=Homo sapiens GN=NT5C2 PE=1 SV=1 - [5NTC_HUMAN] | 6 | 3 | 4 |
| P42765 | 3-ketoacyl-CoA thiolase. mitochondrial OS=Homo sapiens GN=ACAA2 PE=1 SV=2 - [THIM_HUMAN] | 6 | 3 | 4 |
| Q9UBT2 | SUMO-activating enzyme subunit 2 OS=Homo sapiens GN=UBA2 PE=1 SV=2 - [SAE2_HUMAN] | 6 | 3 | 4 |
| Q92990 | Glomulin OS=Homo sapiens GN=GLMN PE=1 SV=2 - [GLMN_HUMAN] | 6 | 3 | 4 |
| Q53GQ0 | Estradiol 17-beta-dehydrogenase 12 OS=Homo sapiens GN=HSD17B12 PE=1 SV=2 - [DHB12_HUMAN] | 5 | 3 | 4 |
| O15144 | Actin-related protein 2/3 complex subunit 2 OS=Homo sapiens GN=ARPC2 PE=1 SV=1 - [ARPC2_HUMAN] | 5 | 3 | 4 |
| Q16795 | NADH dehydrogenase [ubiquinone] 1 alpha subcomplex subunit 9. mitochondrial OS=Homo sapiens GN=NDUFA9 PE=1 SV=2 - [NDUA9_HUMAN] | 5 | 3 | 4 |
| A1L0T0 | Acetolactate synthase-like protein OS=Homo sapiens GN=ILVBL PE=1 SV=2 - [ILVBL_HUMAN] | 5 | 3 | 4 |
| Q96HC4 | PDZ and LIM domain protein 5 OS=Homo sapiens GN=PDLIM5 PE=1 SV=5 - [PDLI5_HUMAN] | 5 | 3 | 4 |
| Q9H9A6 | Leucine-rich repeat-containing protein 40 OS=Homo sapiens GN=LRRC40 PE=1 SV=1 - [LRC40_HUMAN] | 5 | 3 | 4 |
| Q9NZ08 | Endoplasmic reticulum aminopeptidase 1 OS=Homo sapiens GN=ERAP1 PE=1 SV=3 - [ERAP1_HUMAN] | 5 | 3 | 4 |
| P46776 | 60S ribosomal protein L27a OS=Homo sapiens GN=RPL27A PE=1 SV=2 - [RL27A_HUMAN] | 4 | 3 | 4 |
| P83731 | 60S ribosomal protein L24 OS=Homo sapiens GN=RPL24 PE=1 SV=1 - [RL24_HUMAN] | 4 | 3 | 4 |
| Q8IZP2 | Protein FAM10A4 OS=Homo sapiens GN=FAM10A4 PE=1 SV=1 - [F10A4_HUMAN] | 4 | 3 | 4 |
| Q9BTV4 | Transmembrane protein 43 OS=Homo sapiens GN=TMEM43 PE=1 SV=1 - [TMM43_HUMAN] | 4 | 3 | 4 |
| P28072 | Proteasome subunit beta type-6 OS=Homo sapiens GN=PSMB6 PE=1 SV=4 - [PSB6_HUMAN] | 4 | 3 | 4 |
| Q9BT78 | COP9 signalosome complex subunit 4 OS=Homo sapiens GN=COPS4 PE=1 SV=1 - [CSN4_HUMAN] | 4 | 3 | 4 |
| P51570 | Galactokinase OS=Homo sapiens GN=GALK1 PE=1 SV=1 - [GALK1_HUMAN] | 4 | 3 | 4 |
| P14324 | Farnesyl pyrophosphate synthase OS=Homo sapiens GN=FDPS PE=1 SV=4 - [FPPS_HUMAN] | 4 | 3 | 4 |
| P11387 | DNA topoisomerase 1 OS=Homo sapiens GN=TOP1 PE=1 SV=2 - [TOP1_HUMAN] | 4 | 3 | 4 |
| Q92598 | Heat shock protein 105 kDa OS=Homo sapiens GN=HSPH1 PE=1 SV=1 - [HS105_HUMAN] | 4 | 3 | 4 |
| Q92974 | Rho guanine nucleotide exchange factor 2 OS=Homo sapiens GN=ARHGEF2 PE=1 SV=4 - [ARHG2_HUMAN] | 4 | 3 | 4 |
| Q14157 | Ubiquitin-associated protein 2-like OS=Homo sapiens GN=UBAP2L PE=1 SV=2 - [UBP2L_HUMAN] | 4 | 3 | 4 |
| P62851 | 40S ribosomal protein S25 OS=Homo sapiens GN=RPS25 PE=1 SV=1 - [RS25_HUMAN] | 3 | 3 | 4 |
| P61086 | Ubiquitin-conjugating enzyme E2 K OS=Homo sapiens GN=UBE2K PE=1 SV=3 - [UBE2K_HUMAN] | 3 | 3 | 4 |
| P62829 | 60S ribosomal protein L23 OS=Homo sapiens GN=RPL23 PE=1 SV=1 - [RL23_HUMAN] | 3 | 3 | 4 |
| Q13151 | Heterogeneous nuclear ribonucleoprotein A0 OS=Homo sapiens GN=HNRNPA0 PE=1 SV=1 - [ROA0_HUMAN] | 3 | 3 | 4 |
| Q01081 | Splicing factor U2AF 35 kDa subunit OS=Homo sapiens GN=U2AF1 PE=1 SV=3 - [U2AF1_HUMAN] | 3 | 3 | 4 |
| O94826 | Mitochondrial import receptor subunit TOM70 OS=Homo sapiens GN=TOMM70A PE=1 SV=1 - [TOM70_HUMAN] | 3 | 3 | 4 |
| Q02750 | Dual specificity mitogen-activated protein kinase kinase 1 OS=Homo sapiens GN=MAP2K1 PE=1 SV=2 - [MP2K1_HUMAN] | 2 | 3 | 4 |
| Q7L576 | Cytoplasmic FMR1-interacting protein 1 OS=Homo sapiens GN=CYFIP1 PE=1 SV=1 - [CYFP1_HUMAN] | 6 | 3 | 3 |
| P25788 | Proteasome subunit alpha type-3 OS=Homo sapiens GN=PSMA3 PE=1 SV=2 - [PSA3_HUMAN] | 5 | 3 | 3 |
| Q99536 | Synaptic vesicle membrane protein VAT-1 homolog OS=Homo sapiens GN=VAT1 PE=1 SV=2 - [VAT1_HUMAN] | 5 | 3 | 3 |
| Q14166 | Tubulin--tyrosine ligase-like protein 12 OS=Homo sapiens GN=TTLL12 PE=1 SV=2 - [TTL12_HUMAN] | 5 | 3 | 3 |
| O43490 | Prominin-1 OS=Homo sapiens GN=PROM1 PE=1 SV=1 - [PROM1_HUMAN] | 5 | 3 | 3 |
| P49589 | Cysteinyl-tRNA synthetase. cytoplasmic OS=Homo sapiens GN=CARS PE=1 SV=3 - [SYCC_HUMAN] | 5 | 3 | 3 |
| Q02543 | 60S ribosomal protein L18a OS=Homo sapiens GN=RPL18A PE=1 SV=2 - [RL18A_HUMAN] | 4 | 3 | 3 |
| P62995 | Transformer-2 protein homolog beta OS=Homo sapiens GN=TRA2B PE=1 SV=1 - [TRA2B_HUMAN] | 4 | 3 | 3 |
| Q3ZCQ8 | Mitochondrial import inner membrane translocase subunit TIM50 OS=Homo sapiens GN=TIMM50 PE=1 SV=2 - [TIM50_HUMAN] | 4 | 3 | 3 |
| O43684 | Mitotic checkpoint protein BUB3 OS=Homo sapiens GN=BUB3 PE=1 SV=1 - [BUB3_HUMAN] | 4 | 3 | 3 |
| Q14498 | RNA-binding protein 39 OS=Homo sapiens GN=RBM39 PE=1 SV=2 - [RBM39_HUMAN] | 4 | 3 | 3 |
| Q9UJU6 | Drebrin-like protein OS=Homo sapiens GN=DBNL PE=1 SV=1 - [DBNL_HUMAN] | 4 | 3 | 3 |
| P04062 | Glucosylceramidase OS=Homo sapiens GN=GBA PE=1 SV=3 - [GLCM_HUMAN] | 4 | 3 | 3 |
| O60568 | Procollagen-lysine.2-oxoglutarate 5-dioxygenase 3 OS=Homo sapiens GN=PLOD3 PE=1 SV=1 - [PLOD3_HUMAN] | 4 | 3 | 3 |
| Q02809 | Procollagen-lysine.2-oxoglutarate 5-dioxygenase 1 OS=Homo sapiens GN=PLOD1 PE=1 SV=2 - [PLOD1_HUMAN] | 4 | 3 | 3 |
| Q32P28 | Prolyl 3-hydroxylase 1 OS=Homo sapiens GN=LEPRE1 PE=1 SV=2 - [P3H1_HUMAN] | 4 | 3 | 3 |
| Q9UBC2 | Epidermal growth factor receptor substrate 15-like 1 OS=Homo sapiens GN=EPS15L1 PE=1 SV=1 - [EP15R_HUMAN] | 4 | 3 | 3 |
| O96005 | Cleft lip and palate transmembrane protein 1 OS=Homo sapiens GN=CLPTM1 PE=1 SV=1 - [CLPT1_HUMAN] | 4 | 3 | 3 |
| Q9Y2W1 | Thyroid hormone receptor-associated protein 3 OS=Homo sapiens GN=THRAP3 PE=1 SV=2 - [TR150_HUMAN] | 4 | 3 | 3 |
| P63173 | 60S ribosomal protein L38 OS=Homo sapiens GN=RPL38 PE=1 SV=2 - [RL38_HUMAN] | 3 | 3 | 3 |
| P20290 | Transcription factor BTF3 OS=Homo sapiens GN=BTF3 PE=1 SV=1 - [BTF3_HUMAN] | 3 | 3 | 3 |
| Q15102 | Platelet-activating factor acetylhydrolase IB subunit gamma OS=Homo sapiens GN=PAFAH1B3 PE=1 SV=1 - [PA1B3_HUMAN] | 3 | 3 | 3 |
| P37108 | Signal recognition particle 14 kDa protein OS=Homo sapiens GN=SRP14 PE=1 SV=2 - [SRP14_HUMAN] | 3 | 3 | 3 |
| P49721 | Proteasome subunit beta type-2 OS=Homo sapiens GN=PSMB2 PE=1 SV=1 - [PSB2_HUMAN] | 3 | 3 | 3 |
| P28066 | Proteasome subunit alpha type-5 OS=Homo sapiens GN=PSMA5 PE=1 SV=3 - [PSA5_HUMAN] | 3 | 3 | 3 |
| Q13185 | Chromobox protein homolog 3 OS=Homo sapiens GN=CBX3 PE=1 SV=4 - [CBX3_HUMAN] | 3 | 3 | 3 |
| Q9NP79 | Vacuolar protein sorting-associated protein VTA1 homolog OS=Homo sapiens GN=VTA1 PE=1 SV=1 - [VTA1_HUMAN] | 3 | 3 | 3 |
| Q9BRF8 | Serine/threonine-protein phosphatase CPPED1 OS=Homo sapiens GN=CPPED1 PE=1 SV=3 - [CPPED_HUMAN] | 3 | 3 | 3 |
| P39748 | Flap endonuclease 1 OS=Homo sapiens GN=FEN1 PE=1 SV=1 - [FEN1_HUMAN] | 3 | 3 | 3 |
| P50224 | Sulfotransferase 1A3/1A4 OS=Homo sapiens GN=SULT1A3 PE=1 SV=1 - [ST1A3_HUMAN] | 3 | 3 | 3 |
| P11177 | Pyruvate dehydrogenase E1 component subunit beta. mitochondrial OS=Homo sapiens GN=PDHB PE=1 SV=3 - [ODPB_HUMAN] | 3 | 3 | 3 |
| Q9Y6E2 | Basic leucine zipper and W2 domain-containing protein 2 OS=Homo sapiens GN=BZW2 PE=1 SV=1 - [BZW2_HUMAN] | 3 | 3 | 3 |
| O43493 | Trans-Golgi network integral membrane protein 2 OS=Homo sapiens OX=9606 GN=TGOLN2 PE=1 SV=3 - [TGON2_HUMAN] | 3 | 3 | 3 |
| Q6NUK1 | Calcium-binding mitochondrial carrier protein SCaMC-1 OS=Homo sapiens GN=SLC25A24 PE=1 SV=2 - [SCMC1_HUMAN] | 3 | 3 | 3 |
| Q92504 | Zinc transporter SLC39A7 OS=Homo sapiens GN=SLC39A7 PE=1 SV=2 - [S39A7_HUMAN] | 3 | 3 | 3 |
| Q9NUQ6 | SPATS2-like protein OS=Homo sapiens GN=SPATS2L PE=1 SV=2 - [SPS2L_HUMAN] | 3 | 3 | 3 |
| P15586 | N-acetylglucosamine-6-sulfatase OS=Homo sapiens GN=GNS PE=1 SV=3 - [GNS_HUMAN] | 3 | 3 | 3 |
| Q9H074 | Polyadenylate-binding protein-interacting protein 1 OS=Homo sapiens GN=PAIP1 PE=1 SV=1 - [PAIP1_HUMAN] | 3 | 3 | 3 |
| Q9UEY8 | Gamma-adducin OS=Homo sapiens GN=ADD3 PE=1 SV=1 - [ADDG_HUMAN] | 3 | 3 | 3 |
| Q969N2 | GPI transamidase component PIG-T OS=Homo sapiens GN=PIGT PE=1 SV=1 - [PIGT_HUMAN] | 3 | 3 | 3 |
| Q9UN86 | Ras GTPase-activating protein-binding protein 2 OS=Homo sapiens GN=G3BP2 PE=1 SV=2 - [G3BP2_HUMAN] | 3 | 3 | 3 |
| O00505 | Importin subunit alpha-3 OS=Homo sapiens GN=KPNA3 PE=1 SV=2 - [IMA3_HUMAN] | 3 | 3 | 3 |
| P51114 | Fragile X mental retardation syndrome-related protein 1 OS=Homo sapiens GN=FXR1 PE=1 SV=3 - [FXR1_HUMAN] | 3 | 3 | 3 |
| P22307 | Non-specific lipid-transfer protein OS=Homo sapiens GN=SCP2 PE=1 SV=2 - [NLTP_HUMAN] | 3 | 3 | 3 |
| P27694 | Replication protein A 70 kDa DNA-binding subunit OS=Homo sapiens GN=RPA1 PE=1 SV=2 - [RFA1_HUMAN] | 3 | 3 | 3 |
| P84103 | Splicing factor. arginine/serine-rich 3 OS=Homo sapiens GN=SFRS3 PE=1 SV=1 - [SFRS3_HUMAN] | 2 | 3 | 3 |
| P20339 | Ras-related protein Rab-5A OS=Homo sapiens GN=RAB5A PE=1 SV=2 - [RAB5A_HUMAN] | 2 | 3 | 3 |
| P08579 | U2 small nuclear ribonucleoprotein B'' OS=Homo sapiens GN=SNRPB2 PE=1 SV=1 - [RU2B_HUMAN] | 2 | 3 | 3 |
| O60493 | Sorting nexin-3 OS=Homo sapiens GN=SNX3 PE=1 SV=3 - [SNX3_HUMAN] | 2 | 3 | 3 |
| Q16629 | Splicing factor. arginine/serine-rich 7 OS=Homo sapiens GN=SFRS7 PE=1 SV=1 - [SFRS7_HUMAN] | 2 | 3 | 3 |
| P43307 | Translocon-associated protein subunit alpha OS=Homo sapiens GN=SSR1 PE=1 SV=3 - [SSRA_HUMAN] | 2 | 3 | 3 |
| Q14978 | Nucleolar and coiled-body phosphoprotein 1 OS=Homo sapiens GN=NOLC1 PE=1 SV=2 - [NOLC1_HUMAN] | 2 | 3 | 3 |
| P52788 | Spermine synthase OS=Homo sapiens GN=SMS PE=1 SV=2 - [SPSY_HUMAN] | 4 | 3 | 2 |
| Q96N66 | Lysophospholipid acyltransferase 7 OS=Homo sapiens GN=MBOAT7 PE=1 SV=2 - [MBOA7_HUMAN] | 4 | 3 | 2 |
| Q969Z0 | Protein TBRG4 OS=Homo sapiens GN=TBRG4 PE=1 SV=1 - [TBRG4_HUMAN] | 4 | 3 | 2 |
| Q9P035 | Protein tyrosine phosphatase-like protein PTPLAD1 OS=Homo sapiens GN=PTPLAD1 PE=1 SV=2 - [PTAD1_HUMAN] | 4 | 3 | 2 |
| Q12797 | Aspartyl/asparaginyl beta-hydroxylase OS=Homo sapiens GN=ASPH PE=1 SV=3 - [ASPH_HUMAN] | 4 | 3 | 2 |
| Q8TAT6 | Nuclear protein localization protein 4 homolog OS=Homo sapiens GN=NPLOC4 PE=1 SV=3 - [NPL4_HUMAN] | 4 | 3 | 2 |
| Q07157 | Tight junction protein ZO-1 OS=Homo sapiens GN=TJP1 PE=1 SV=3 - [ZO1_HUMAN] | 4 | 3 | 2 |
| Q92538 | Golgi-specific brefeldin A-resistance guanine nucleotide exchange factor 1 OS=Homo sapiens GN=GBF1 PE=1 SV=2 - [GBF1_HUMAN] | 4 | 3 | 2 |
| P60983 | Glia maturation factor beta OS=Homo sapiens GN=GMFB PE=1 SV=2 - [GMFB_HUMAN] | 3 | 3 | 2 |
| P53999 | Activated RNA polymerase II transcriptional coactivator p15 OS=Homo sapiens GN=SUB1 PE=1 SV=3 - [TCP4_HUMAN] | 3 | 3 | 2 |
| O75396 | Vesicle-trafficking protein SEC22b OS=Homo sapiens GN=SEC22B PE=1 SV=4 - [SC22B_HUMAN] | 3 | 3 | 2 |
| P24534 | Elongation factor 1-beta OS=Homo sapiens GN=EEF1B2 PE=1 SV=3 - [EF1B_HUMAN] | 3 | 3 | 2 |
| P00492 | Hypoxanthine-guanine phosphoribosyltransferase OS=Homo sapiens GN=HPRT1 PE=1 SV=2 - [HPRT_HUMAN] | 3 | 3 | 2 |
| Q9UM00 | Calcium load-activated calcium channel OS=Homo sapiens OX=9606 GN=TMCO1 PE=1 SV=2 - [TMCO1_HUMAN] | 3 | 3 | 2 |
| P06730 | Eukaryotic translation initiation factor 4E OS=Homo sapiens GN=EIF4E PE=1 SV=2 - [IF4E_HUMAN] | 3 | 3 | 2 |
| P84098 | 60S ribosomal protein L19 OS=Homo sapiens GN=RPL19 PE=1 SV=1 - [RL19_HUMAN] | 3 | 3 | 2 |
| O15145 | Actin-related protein 2/3 complex subunit 3 OS=Homo sapiens GN=ARPC3 PE=1 SV=3 - [ARPC3_HUMAN] | 3 | 3 | 2 |
| Q6PIU2 | Neutral cholesterol ester hydrolase 1 OS=Homo sapiens GN=NCEH1 PE=1 SV=3 - [NCEH1_HUMAN] | 3 | 3 | 2 |
| Q8TC12 | Retinol dehydrogenase 11 OS=Homo sapiens GN=RDH11 PE=1 SV=2 - [RDH11_HUMAN] | 3 | 3 | 2 |
| P31153 | S-adenosylmethionine synthetase isoform type-2 OS=Homo sapiens GN=MAT2A PE=1 SV=1 - [METK2_HUMAN] | 3 | 3 | 2 |
| P11766 | Alcohol dehydrogenase class-3 OS=Homo sapiens GN=ADH5 PE=1 SV=4 - [ADHX_HUMAN] | 3 | 3 | 2 |
| Q16186 | Proteasomal ubiquitin receptor ADRM1 OS=Homo sapiens GN=ADRM1 PE=1 SV=2 - [ADRM1_HUMAN] | 3 | 3 | 2 |
| Q15717 | ELAV-like protein 1 OS=Homo sapiens GN=ELAVL1 PE=1 SV=2 - [ELAV1_HUMAN] | 3 | 3 | 2 |
| P10155 | 60 kDa SS-A/Ro ribonucleoprotein OS=Homo sapiens GN=TROVE2 PE=1 SV=2 - [RO60_HUMAN] | 3 | 3 | 2 |
| Q13501 | Sequestosome-1 OS=Homo sapiens GN=SQSTM1 PE=1 SV=1 - [SQSTM_HUMAN] | 3 | 3 | 2 |
| Q12907 | Vesicular integral-membrane protein VIP36 OS=Homo sapiens GN=LMAN2 PE=1 SV=1 - [LMAN2_HUMAN] | 3 | 3 | 2 |
| Q12765 | Secernin-1 OS=Homo sapiens GN=SCRN1 PE=1 SV=2 - [SCRN1_HUMAN] | 3 | 3 | 2 |
| Q92905 | COP9 signalosome complex subunit 5 OS=Homo sapiens GN=COPS5 PE=1 SV=4 - [CSN5_HUMAN] | 3 | 3 | 2 |
| Q13895 | Bystin OS=Homo sapiens GN=BYSL PE=1 SV=3 - [BYST_HUMAN] | 3 | 3 | 2 |
| P06865 | Beta-hexosaminidase subunit alpha OS=Homo sapiens GN=HEXA PE=1 SV=2 - [HEXA_HUMAN] | 3 | 3 | 2 |
| P58107 | Epiplakin OS=Homo sapiens OX=9606 GN=EPPK1 PE=1 SV=3 - [EPIPL_HUMAN] | 3 | 3 | 2 |
| O95487 | Protein transport protein Sec24B OS=Homo sapiens GN=SEC24B PE=1 SV=2 - [SC24B_HUMAN] | 3 | 3 | 2 |
| P42766 | 60S ribosomal protein L35 OS=Homo sapiens GN=RPL35 PE=1 SV=2 - [RL35_HUMAN] | 2 | 3 | 2 |
| Q15738 | Sterol-4-alpha-carboxylate 3-dehydrogenase. decarboxylating OS=Homo sapiens GN=NSDHL PE=1 SV=2 - [NSDHL_HUMAN] | 2 | 3 | 2 |
| Q92599 | Septin-8 OS=Homo sapiens GN=SEPT8 PE=1 SV=4 - [SEPT8_HUMAN] | 2 | 3 | 2 |
| Q7Z4V5 | Hepatoma-derived growth factor-related protein 2 OS=Homo sapiens GN=HDGFRP2 PE=1 SV=1 - [HDGR2_HUMAN] | 2 | 3 | 2 |
| Q9Y6E0 | Serine/threonine-protein kinase 24 OS=Homo sapiens GN=STK24 PE=1 SV=1 - [STK24_HUMAN] | 2 | 3 | 2 |
| Q14739 | Lamin-B receptor OS=Homo sapiens GN=LBR PE=1 SV=2 - [LBR_HUMAN] | 4 | 3 | 1 |
| P43487 | Ran-specific GTPase-activating protein OS=Homo sapiens GN=RANBP1 PE=1 SV=1 - [RANG_HUMAN] | 3 | 3 | 1 |
| Q16543 | Hsp90 co-chaperone Cdc37 OS=Homo sapiens GN=CDC37 PE=1 SV=1 - [CDC37_HUMAN] | 3 | 3 | 1 |
| O14828 | Secretory carrier-associated membrane protein 3 OS=Homo sapiens GN=SCAMP3 PE=1 SV=3 - [SCAM3_HUMAN] | 3 | 3 | 1 |
| O15479 | Melanoma-associated antigen B2 OS=Homo sapiens GN=MAGEB2 PE=1 SV=3 - [MAGB2_HUMAN] | 3 | 3 | 1 |
| Q92747 | Actin-related protein 2/3 complex subunit 1A OS=Homo sapiens GN=ARPC1A PE=1 SV=2 - [ARC1A_HUMAN] | 3 | 3 | 1 |
| Q5TFE4 | 5'-nucleotidase domain-containing protein 1 OS=Homo sapiens GN=NT5DC1 PE=1 SV=1 - [NT5D1_HUMAN] | 3 | 3 | 1 |
| Q96S52 | GPI transamidase component PIG-S OS=Homo sapiens GN=PIGS PE=1 SV=3 - [PIGS_HUMAN] | 3 | 3 | 1 |
| Q9UHD1 | Cysteine and histidine-rich domain-containing protein 1 OS=Homo sapiens GN=CHORDC1 PE=1 SV=2 - [CHRD1_HUMAN] | 3 | 3 | 1 |
| Q9UNH7 | Sorting nexin-6 OS=Homo sapiens GN=SNX6 PE=1 SV=1 - [SNX6_HUMAN] | 3 | 3 | 1 |
| P10515 | Dihydrolipoyllysine-residue acetyltransferase component of pyruvate dehydrogenase complex. mitochondrial OS=Homo sapiens GN=DLAT PE=1 SV=3 - [ODP2_HUMAN] | 3 | 3 | 1 |
| Q9H6S3 | Epidermal growth factor receptor kinase substrate 8-like protein 2 OS=Homo sapiens GN=EPS8L2 PE=1 SV=2 - [ES8L2_HUMAN] | 3 | 3 | 1 |
| Q9BZE4 | Nucleolar GTP-binding protein 1 OS=Homo sapiens GN=GTPBP4 PE=1 SV=3 - [NOG1_HUMAN] | 3 | 3 | 1 |
| P98082 | Disabled homolog 2 OS=Homo sapiens GN=DAB2 PE=1 SV=3 - [DAB2_HUMAN] | 3 | 3 | 1 |
| Q9NZB2 | Constitutive coactivator of PPAR-gamma-like protein 1 OS=Homo sapiens GN=FAM120A PE=1 SV=2 - [F120A_HUMAN] | 3 | 3 | 1 |
| P02751 | Fibronectin OS=Homo sapiens GN=FN1 PE=1 SV=4 - [FINC_HUMAN] | 3 | 3 | 1 |
| P62847 | 40S ribosomal protein S24 OS=Homo sapiens GN=RPS24 PE=1 SV=1 - [RS24_HUMAN] | 2 | 3 | 1 |
| O95292 | Vesicle-associated membrane protein-associated protein B/C OS=Homo sapiens GN=VAPB PE=1 SV=3 - [VAPB_HUMAN] | 2 | 3 | 1 |
[truncated: 96,211 more chars]
